# Stereoselective Cyclopropanation of 1,1-Diborylalkenes via Palladium-Catalyzed (Trimethylsilyl)diazomethane Insertion

**DOI:** 10.1021/acs.orglett.2c01885

**Published:** 2022-07-07

**Authors:** Oriol Salvado, Paula Dominguez-Molano, Elena Fernández

**Affiliations:** Departament de Química Física i Inorgànica, University Rovira i Virgili, 43007 Tarragona, Spain

## Abstract

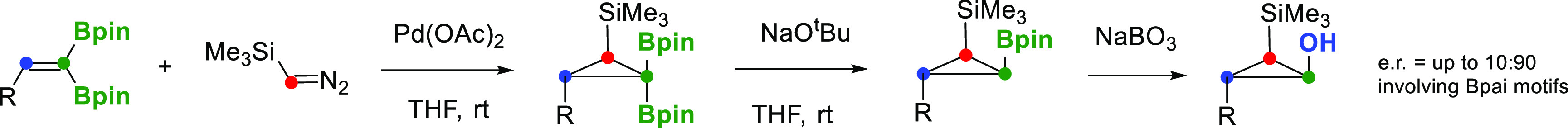

Palladium catalyzes the cyclopropanation of 2-substituted 1,1-diborylalkenes with (trimethylsilyl)diazomethane. The relative stereoselectivity is controlled via a carbene insertion sequence generating an exclusive *anti* conformation between the R and SiMe_3_ substituents. Mixed 1,1-diborylalkenes also contributed to the formation of stereoselective B, B, Si-cyclopropanes. Orthogonal activation with NaO^*t*^Bu gives protodeborylation preferentially on the boron moiety *syn* to the aryl group. Further oxidation gives access to polyfunctional cyclopropyl alcohols with controlled enantioselectivity when chiral boryl motifs are involved.

Polyborylated carbon frameworks act as valuable building blocks to be transformed into biologically active substances and functional organic materials.^1^ In particular, *gem*-bis(boryl)cyclopropanes represent an important structural motif to be sequentially functionalized into target cyclopropane frameworks involved in the pharmaceutical industry.^2^ An early approach to synthesizing *gem*-bis(boryl)cyclopropane was developed on the basis of the efficient reactivity between 1,1-dibromocyclopropanes and bis(pinacolato)diboron (B_2_pin_2_) at low temperatures.^3^ The protocol implied the *in situ* formation of cyclopropylidene lithium carbenoids that interact with B_2_pin_2_ to form the boronate intermediate that evolves, through 1,2-migration, toward the corresponding 1,1-diborylated cyclopropane. The relative *syn* or *anti* conformation of the substituents is fixed along the 1,1-dibromocyclopropane formation by choosing the appropriate *Z*- or *E*-alkene, respectively (Scheme 1).^3^

**Scheme 1:**
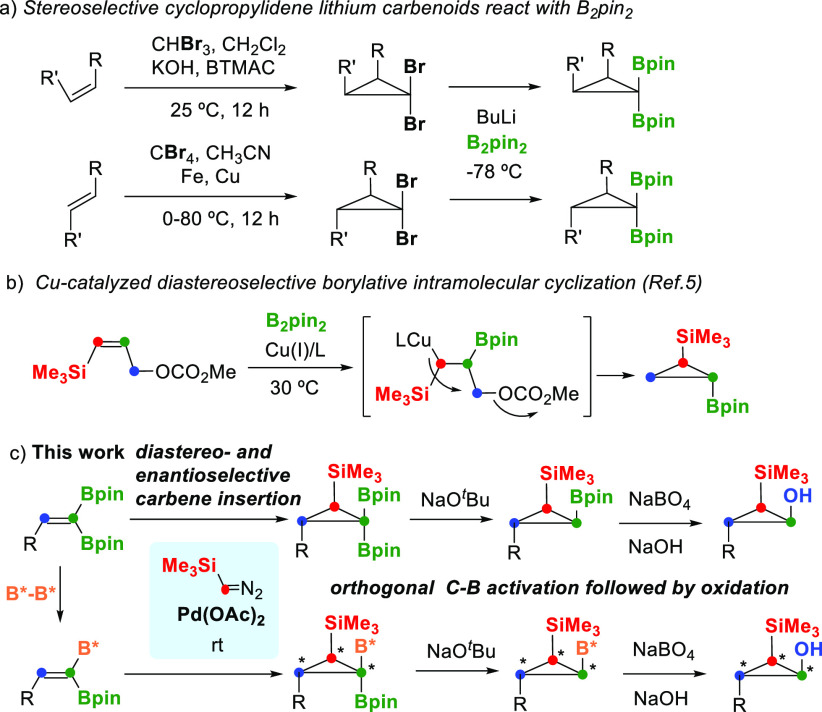
Synthetic Approaches to 1,1-Diborylcyclopropanes via (a) Cyclopropylidene Lithium Carbenoids, (b) Cu-Catalyzed Borylative Intramolecular Cyclization, and (c) Pd-Catalyzed TMSDM Insertion on 1,1-Diborylalkenes

With the aim of contributing to the modulated construction of polyfunctionalized *gem*-bis(boryl)cyclopropanes, we describe here a direct cyclopropanation process via palladium-catalyzed addition of (trimethylsilyl)diazomethane (TMSDM) to 2-substituted 1,1-diborylalkenes (Scheme 1c). The relative stereoselectivity can be controlled throughout the carbene insertion step, showing an exclusive *anti* conformation of the vicinal R and SiMe_3_ substituents on the new B, B, Si-cyclopropanes. This methodology avoids the use of brominated cyclopropanes^3,4^ and seems to be highly tolerant of the nature of the substituents on the alkene. To the best of our knowledge, only one precedent has reported the preparation of *anti*-B, Si bifunctional cyclopropanes, through copper-catalyzed intramolecular borylative cyclization of γ-silylated allylic carbonates with B_2_pin_2_ (Scheme 1b).^5^ We have also explored the orthogonal functionalization of the tetrasubstituted carbon atom as a key connective unit for selective B activation. In the presence of NaO^*t*^Bu, the Bpin moiety *syn* to R suffers protodeborylation, suggesting that SiMe_3_ might protect the *syn* boryl unit. Subsequent oxidation gives access to the stereoselective *syn*-2-(trimethylsilyl)cyclopropan-1-ols (Scheme 1b). Our method contributes to the preparation of stereoselective *gem*-bis(boryl)cyclopropanes containing different boryl moieties, as an alternative to the reported method based on the diastereotopic pinacolboryl desymmetrization via trifluorination.^6^

The preparation of 1,1-bis(pinacolboryl)alkenes^7^ can be performed by alkylidene-type lithium carbenoids that react with B_2_pin_2_.^8^ However, to avoid the use of halogenated reagents in our new synthetic strategy, we faced the condensation of tris(boryl)methane with aldehydes followed by B–O elimination (Scheme 2, method A). Matteson originally described that tris(boryl)methide ions could be formed by treatment of tetra(boryl)methane with methyllithium to eventually react with formaldehyde and benzaldehyde to undergo the expected condensation.^9^ Here, we have adapted the boron-Wittig reaction^10^ synthesizing tris(pinacolboryl)methane (**1**) that forms *in situ* the corresponding salt Li[C(Bpin)_3_], after treatment with LiTMP. The organolithium Li[C(Bpin)_3_] reacts with a variety of aldehydes to perform the condensation/B–O elimination, with the subsequent formation of the trisubstituted *gem*-diborylalkenes. Scheme 2 shows that benzyl, alkyl, and aryl aldehydes can be efficiently transformed into 2-substituted 1,1-diborylalkenes **2a**, **2c**, **2f**, and **2h** through method A.

**Scheme 2:**
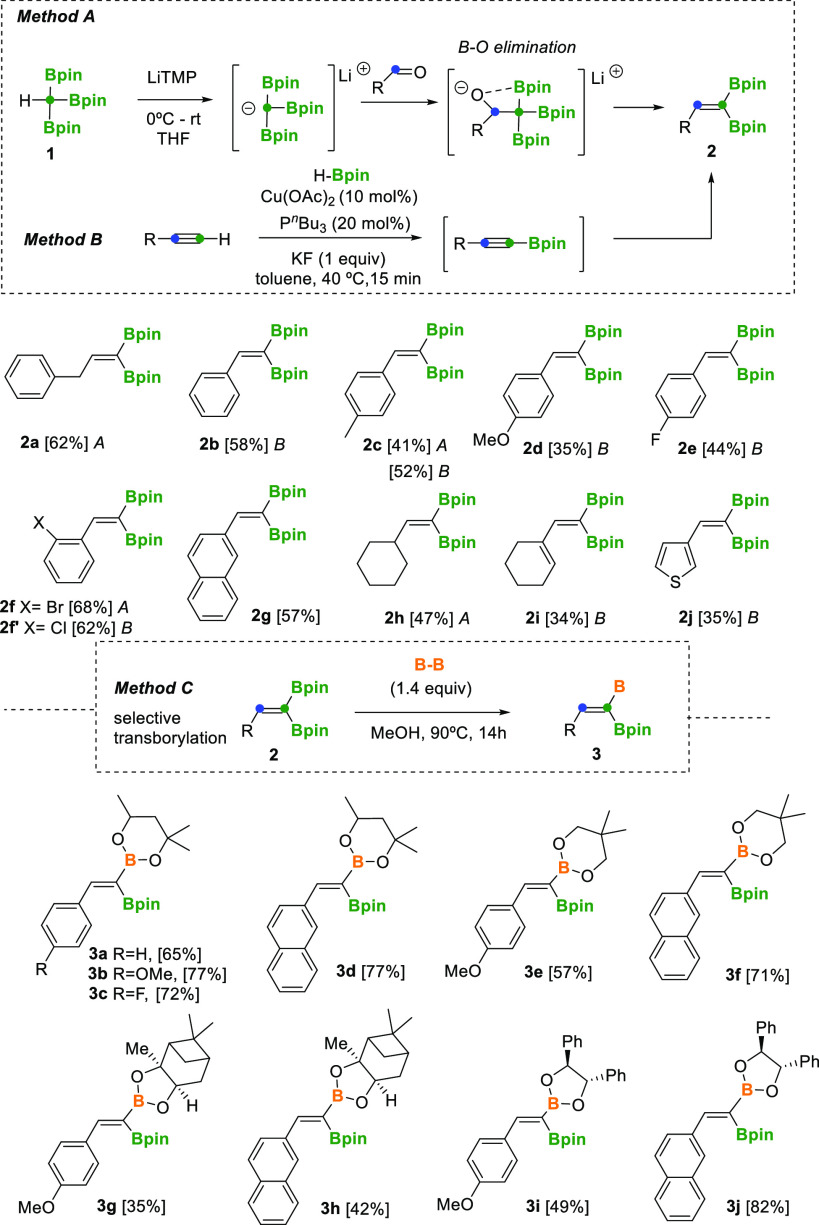
Synthesis of 2-Substituted 1,1-Diborylalkenes through Condensation of Lithium Tris(pinacolboryl)methide and Aldehydes (Method A), Copper-Catalyzed Dehydrogenative Borylation/Hydroboration of Alkynes (Method B), and Transborylation Reaction (Method C)

We also developed an alternative method B to generate 2-substituted 1,1-diborylalkenes from accessible alkynes via copper-catalyzed dehydrogenative borylation/hydroboration with pinacolborane (HBpin) (Scheme 2, method B). Recently, Marder and co-workers described a related protocol for preparing triborylalkanes from alkynes,^11^ whereas Miura, Murakami, and co-workers developed a cobalt(II)-catalyzed 1,1-diboration of alkynes with B_2_pin_2_ to gain access to 1,1-diborylalkenes.^12^ 2-Naphthyl-substituted *gem*-diborylalkene **2g** has been prepared by a boryl-Heck reaction reported previously.^13^

We next explored the preparation of valuable mixed 1,1-diborylalkenes, which have been prepared only via hydroboration of alkynyl boronic esters^14,15^ or Co-catalyzed 1,1-diboration of terminal alkynes with nonsymmetrical diboron reagents.^16^ Here, we adapted the protocol for the B–C(sp^2^)–B/B′–B′ cross metathesis reaction based on our recently developed transborylation sequence.^17^ Consequently, 2-substituted 1,1-bis(pinacolboryl)alkenes reacted with bis(hexylene glycolato) diboron (B_2_hex_2_) or bis(neopenthyl glycolato) diboron (B_2_neo_2_), in MeOH at 90 °C, to generate the mixed 2-aryl 1,1-diborylalkenes **3a–3f** (Scheme 2, method C). The transborylation took place stereoselectively on the less sterically hindered position, as we unambiguously proved by one-dimensional (1D) NMR NOE experiments. Similarly, the transborylation between 1,1-bis(pinacolboryl)alkenes and bis(+)-pinanediolato diboron (B_2_pai_2_) or (4*S*,4′*S*,5*S*,5′*S*)-4,4′,5,5′-tetraphenyl-2,2′-bi(1,3,2-dioxaborolane) (*S,S*)-B_2_(O-CHPh-CHPh-O)_2_ was conducted to isolate the chiral mixed 2-aryl 1,1-diborylalkenes **3g–3j** (Scheme 2, method C).

For the cyclopropanation of 1,1-diborylalkenes, we became inspired by the previous studies of Carboni and co-workers concerned with the palladium-catalyzed addition of diazomethanes to 1-alkenylboronates.^18^ We selected (trimethylsilyl)diazomethane (TMSDM), as the carbene source, to be added on the 2-substituted 1,1-diborylalkenes with the aim of generating polyfunctionalized B, B, Si-cyclopropanes. To the best of our knowledge, cyclopropanation with TMSDM was achieved only through copper-catalyzed addition to vinylarenes.^19^ The proof of concept was conducted on 1,1-diborylalkene **2a** in the presence of Pd(OAc)_2_ (15 mol %) and TMSDM, in hexane at rt. To our delight, the reaction was completed in 16 h with total control of the stereoselectivity, placing the trimethylsilyl and benzyl groups with *anti* conformation in the new product **4** (Scheme 3a). A similar reaction outcome was observed for cyclopropanation of 2-aryl-substituted 1,1-diborylalkenes **2b–2g** independent of the electron rich or electron poor aryl substituents involved. The diastereoisomer with *anti* conformation between the SiMe_3_ and the aryl groups were also exclusively formed in products **5–10** (Scheme 3a). The suggested model for the diastereoselectivity observed on the Pd-catalyzed cyclopropanation of 2-aryl 1,1-diborylalkenes with TMSDM might involve migratory insertion of Pd=CH-TMS into the trisubstituted alkenes (Scheme 3b). The observed preferred *anti* diastereoselection contrasts with the favored *syn* diastereoselection in the synthesis of 1-boryl 2,3-disubstituted cyclopropanes through cyclopropanation of alkenylboronates with ethyl diazoacetate in the presence of catalytic amounts of a copper(I) complex.^20^

**Scheme 3:**
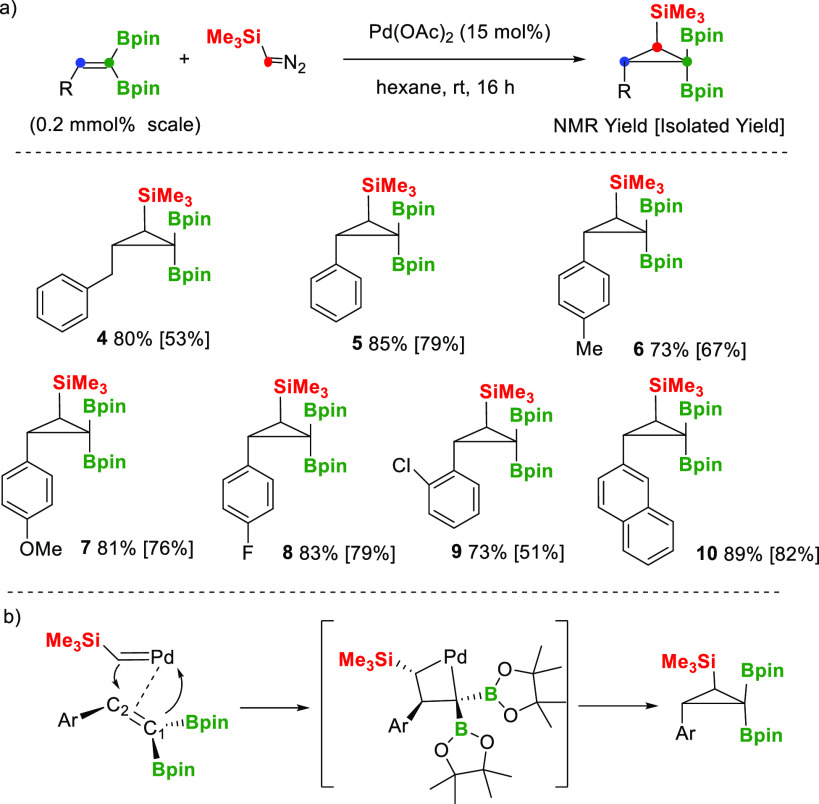
Pd-Catalyzed Stereoselective Cyclopropanation of 2-Substituted 1,1-Diborylakenes with (Trimethylsilyl)diazomethane [(a) substrate scope and (b) suggested mechanistic model]

Surprisingly, when 2-aryl-substituted 1,1-diborylalkene **2f** reacted with TMSDM, in the presence of Pd(OAc)_2_, the cyclopropanation did not occur and (*E*)-vinyl silane product **A** was isolated instead (Scheme 4). The formation of this product could be explained by the oxidative addition of Ar–Br to Pd, followed by a double-palladium carbene migratory insertion process (Scheme 4). Similar direct olefination of aryl/alkyl halides with (trimethylsilyl)methylene was observed by Chen and Xu to occur via carbene migratory insertion in the presence of palladium complexes.^21^ The cyclopropanation of 2-cyclohexyl 1,1-diborylalkene (**2h**), 2-cyclohexenyl 1,1-diborylalkene (**2i**), and 2-(3-thiophenyl) 1,1-diborylalkene (**2j**) did not progress toward the desired product, suggesting an inhibited migratory insertion of the alkene into the Pd=CH-TMS intermediate, as a consequence of the lower electrophilic character of C_2_.

**Scheme 4:**
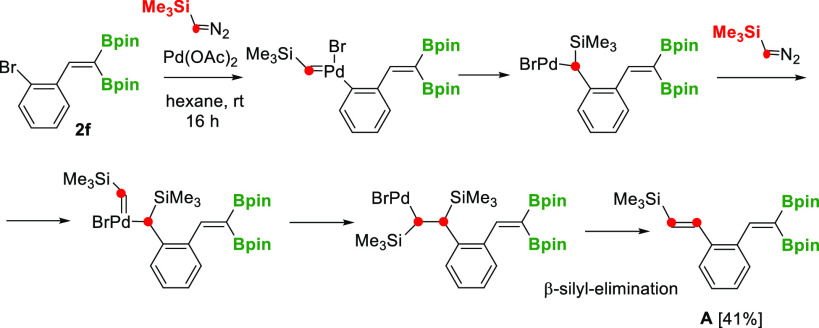
Pd-Catalyzed Olefination of the 2-Br Aryl Group with (Trimethylsilyl)diazomethane

The Pd-catalyzed cyclopropanation of the mixed 1,1-(BpinBhex)alkenes **3a–3d** with TMSDM resulted in high stereoselectivity, providing one exclusive conformer in which the Bhex moiety appears *syn* to the SiMe_3_ group, whereas the Bpin fragment is placed *syn* to the aryl group, for compounds **11–14** (Scheme 5). The diastereoselection has been unambiguously determined by 1D NMR NOE experiments, and in product **11**, we have been able to isolate the two isomers with regard to the Me conformation on the Bhex group.^22^ Interestingly, when we conducted the Pd-catalyzed cyclopropanation of the mixed chiral 1,1-BpinB* alkenes **3g–3j** with TMSDM [B* = Bpai = (+)-pinanediolboryl or (*S,S*)-B_2_(O-CHPh-CHPh-O)_2_], the corresponding B*, Bpin, Si-cyclopropanes **15–18** were isolated as unique isomers, in contrast to the reported Pd-catalyzed cyclopropanation of alkenylmonoboronates, containing Bpai motifs, using CH_2_N_2_ as the carbene source, providing a modest diastereselection of ≤63:37.^23^ It is worth mentioning, for comparison, that Masarwa and co-workers suggested a complementary diastereoselective model for the desymmetrization of *gem*-diborylcyclopropanes via nucleophilic “trifluorination” of the Bpin group, taking place on the less sterically hindered face of the cyclopropane (Scheme 5b).^6^

**Scheme 5:**
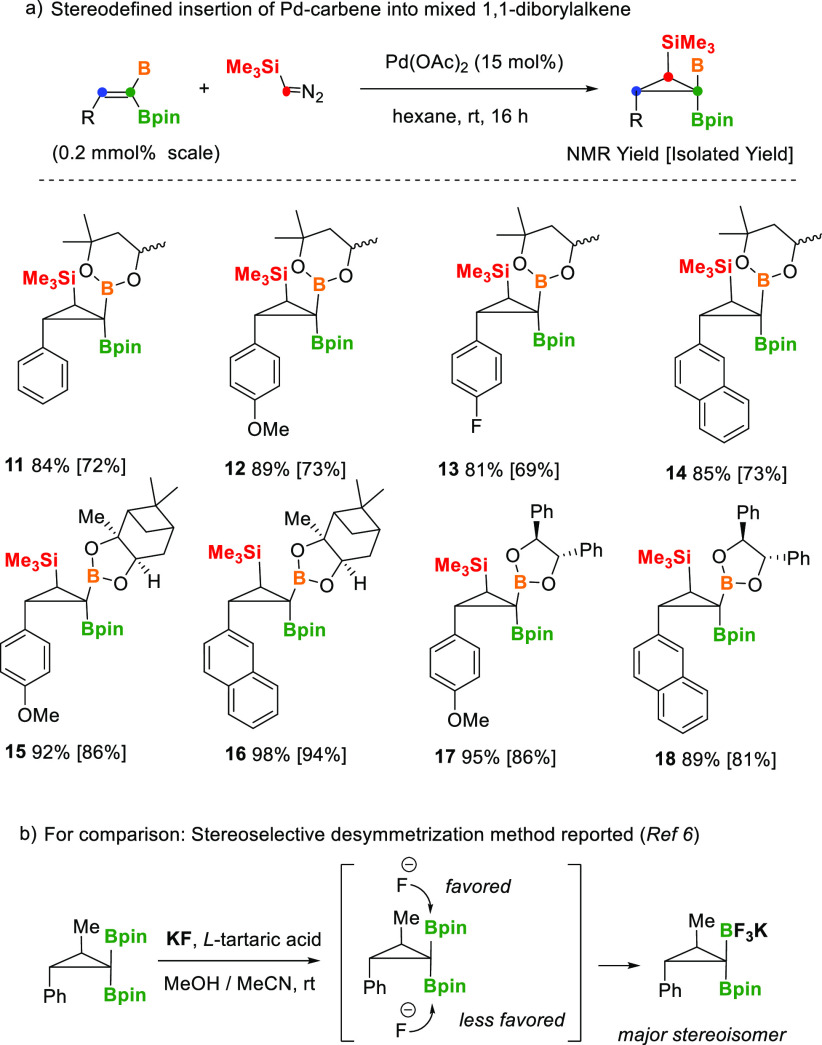
Stereoselective Pd-Catalyzed Cyclopropanation of Mixed 1,1-Diborylakenes with TMSDM and Comparison with Desymmetrization Pathways

Taking advantage of the stereoselective formation of the B, B, Si-cyclopropanes prepared in this work, we next conducted the orthogonal functionalization of the *gem*-bis(boryl)cyclopropanes. When we applied the protodeborylation protocol with NaO^*t*^Bu (3 equiv) at 60 °C on B, B, Si-cyclopropane **7**, we observed a preferred activation of the Bpin unit *syn* to the aryl group to form **19a** in 86% yield, instead of the activation of the Bpin unit *syn* to the SiMe_3_, which generates **19b** in 14% yield (Scheme 6a). A similar preferred reaction outcome was observed for the protodeborylation of B, B, Si-cyclopropanes **9** and **10**, toward products **20a** and **21a**, respectively (Scheme 6a). The steric hindrance associated with the SiMe_3_ group might justify the selective protodeborylation. This hypothesis is in contrast to the selective alkoxide-assisted protodeborylation of *gem*-BpinBdan-cyclohexanes, based on the different electronic properties of the boryl moieties and the enhanced stabilization of the carbanion p-type electron density into the π-channel of Bdan units (Scheme 6b).^24^ The resulting *syn*-B, Si bifunctional cyclopropanes are complementary to the *anti*-B, Si bifunctional cyclopropanes synthesized by Sawamura and Ito through the copper-catalyzed intramolecular borylative cyclization of γ-silylated allylic carbonates with B_2_pin_2_ (Scheme 1b).^5^ Subsequent oxidation of **19a**, **20a**, and **21a** produced the corresponding (aryl)-3-(trimethylsilyl)cyclopropan-1-ol (**22–24**) in quantitative yields (Scheme 6a).

**Scheme 6:**
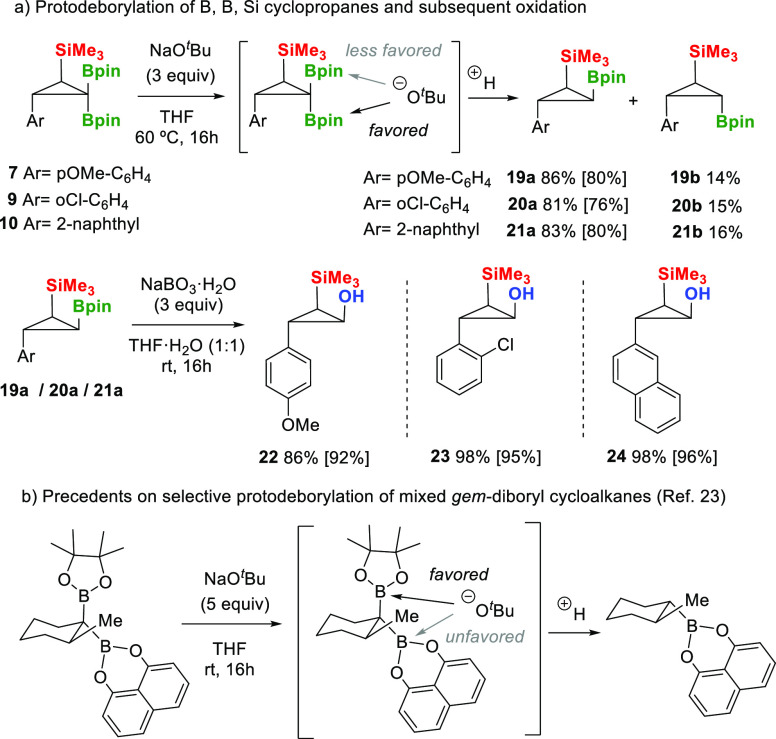
Site-Selective Protodeborylation of *gem*-Bis(boryl)cyclopropanes and *gem*-Bis(boryl)cyclohexanes

B*, B, Si-cyclopropanes **15–18**, containing the chiral boryl units B* = (+)-pinanediolboryl (Bpai) or (*S,S*)-B_2_(O-CHPh-CHPh-O)_2_, also reacted with NaO^*t*^Bu (3 equiv) at 60 °C to protodeborylate exclusively the Bpin unit (Scheme 7). The X-ray single-crystal diffraction analysis of compound **25** projected the absolute configuration of the three new stereocenters formed on the major enantiomer (Figure 1). The enantiomeric ratio was determined from the corresponding alcohol derivatives, after oxidation of B*, Si-cyclopropanes **25–28** with NaBO^3^, in comparison with racemic samples **22** and **24**. The enantiomeric ratio seems to be slightly higher when B* = (+)-pinanediolboryl (Bpai) is involved rather than (*S,S*)-B_2_(O-CHPh-CHPh-O)_2_, independent of the aryl group present in the compounds (Scheme 7). This is presumably a result of an efficient asymmetric induction during the palladium insertion of TMSDM into chiral mixed 2-aryl 1,1-diborylalkenes **3g** and **3h** versus **3i** and **3j**.

**Scheme 7:**
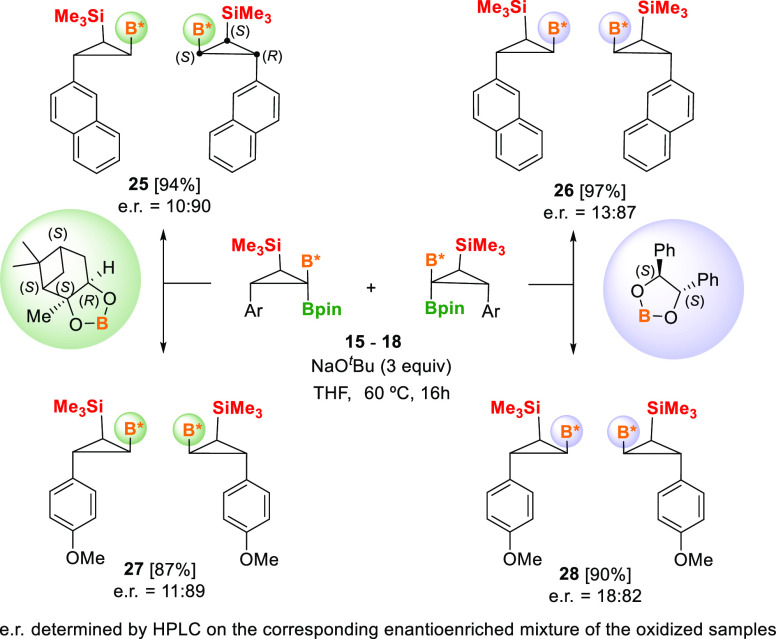
Enantioenriched Synthesis of B*, Si-Cyclopropane Compounds

**Figure 1:**
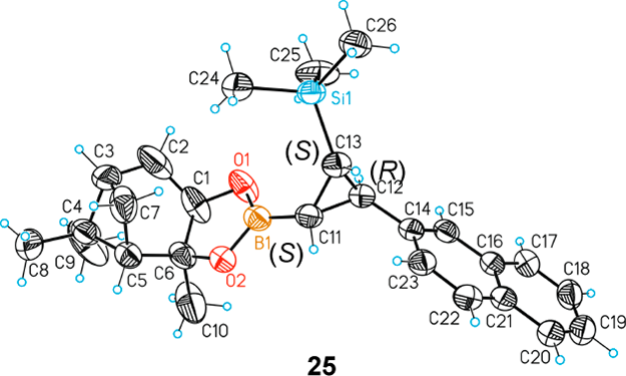
X-ray single-crystal diffraction analysis of the major enantiomer of compound **25**. Thermal ellipsoids draw at the 50% level.

In conclusion, we have described a palladium-catalyzed cyclopropanation of 2-substituted 1,1-diborylalkenes with (trimethylsilyl)diazomethane. The relative stereoselectivity is controlled via a carbene insertion sequence generating an exclusive *anti* conformation between R and SiMe_3_ substituents and an enantiomeric ratio of ≤10:90 when B* = (+)-pinanediolboryl (Bpai) is involved. The new B, B, Si-cyclopropanes can be activated by NaO^*t*^Bu, via protodeborylation preferentially on the boron moiety *syn* to the aryl group. Further oxidation enabled the formation of polyfunctional cyclopropyl alcohols with controlled stereoselectivity and enantioselectivity.

## Supporting Information Available

Experimental procedures, characterization data, ^1^H, ^13^C, and ^11^B NMR spectra for new compounds, and enantiomeric ratios determined by HPLC (PDF)

## References

[ref1] Wang X.; Wang Y.; Huang W.; Xia Ch.; Wu L. Direct Synthesis of Multi(boronate) Esters from Alkenes and Alkynes via Hydroboration and Boration Reactions. ACS Catal. 2021, 11, 1 10.1021/acscatal.0c03418.

[ref2] Harris M. R.; Wisniewska H. M.; Jiao W.; Wang X.; Bradow J. N. A Modular Approach to the Synthesis of gem-Disubstituted Cyclopropanes. Org. Lett. 2018, 20, 2867 10.1021/acs.orglett.8b00899. 29707948

[ref3] Shimizu M.; Schelper M.; Nagao I.; Shimono K.; Kurahashi T.; Hiyama T. Synthesis and Applications of 1,1-Diborylated Cyclopropanes: Facile Route to 1,2-Diboryl-3-methylenecyclopentenes. Chem. Lett. 2006, 35, 1222 10.1246/cl.2006.1222.

[ref4] Hong K.; Liu X.; Morken J. P. Simple Access to Elusive α-Boryl Carbanions and Their Alkylation: An Umpolung Construction for Organic Synthesis. J. Am. Chem. Soc. 2014, 136, 10581 10.1021/ja505455z. 25019925 PMC4353009

[ref5] Ito H.; Kosaka Y.; Nonoyama K.; Sasaki Y.; Sawamura M. Synthesis of Optically Active Boron–Silicon Bifunctional Cyclopropane Derivatives through Enantioselective Copper(I)-Catalyzed Reaction of Allylic Carbonates with a Diboron Derivative. Angew. Chem., Int. Ed. 2008, 47, 7424 10.1002/anie.200802342. 18726978

[ref6] Kumar N.; Reddy R. R.; Masarwa A. Stereoselective Desymmetrization of gem-Diborylalkanes by Trifluorination. Chem.- Eur. J. 2019, 25, 8008 10.1002/chem.201901267. 30964216

[ref7] Royes J.; Cuenca A. B.; Fernández E. Access to 1,1-Diborylalkenes and Concomitant Stereoselective Reactivity. Eur. J. Org. Chem. 2018, 2018, 2728 10.1002/ejoc.201701786.

[ref8] aHata T.; Kitagawa H.; Masai H.; Kurahashi T.; Shimizu M.; Hiyama T. Geminal Difunctionalization of Alkenylidene-Type Carbenoids by Using Interelement Compounds. Angew. Chem., Int. Ed. 2001, 40, 790 10.1002/1521-3773(20010216)40:4<790::AID-ANIE7900>3.0.CO;2-T. 11241626

[ref9] Matteson D. S.; Tripathy P. B. Alkene-1,1-diboronic esters from the condensation of triborylmethide anions with ketones or aldehydes. J. Organomet. Chem. 1974, 69, 53 10.1016/S0022-328X(00)92985-1.

[ref10] Cuenca A. B.; Fernández E. Boron-Wittig olefination with *gem*-bis(boryl)alkanes. Chem. Soc. Rev. 2021, 50, 72 10.1039/D0CS00953A. 33319887

[ref11] Liu X.; Ming W.; Zhang Y.; Friedrich A.; Marder T. B. Copper-Catalyzed Triboration: Straightforward, Atom-Economical Synthesis of 1,1,1-Triborylalkanes from Terminal Alkynes and HBpin. Angew. Chem., Int. Ed. 2019, 58, 18923 10.1002/anie.201909376. PMC6972527 31490606

[ref12] Miura T.; Oku N.; Murakami M. Diastereo- and Enantioselective Synthesis of (E)-δ-Boryl-Substituted anti-Homoallylic Alcohols in Two Steps from Terminal Alkynes. Angew. Chem., Int. Ed. 2019, 58, 14620 10.1002/anie.201908299. 31392816

[ref13] Idowu O. O.; Hayes J. C.; Reid W. B.; Watson D. A. Synthesis of 1,1-Diboryl Alkenes Using the Boryl-Heck Reaction. Org. Lett. 2021, 23, 4838 10.1021/acs.orglett.1c01567. 34043367 PMC8217338

[ref14] aLi H.; Carroll P. J.; Walsh P. J. Generation and Tandem Reactions of 1-Alkenyl-1,1-Heterobimetallics: Practical and Versatile Reagents for Organic Synthesis. J. Am. Chem. Soc. 2008, 130, 3521 10.1021/ja077664u. 18302376

[ref15] Weber L.; Eickhoff D.; Halama J.; Werner S.; Kahlert J.; Stammler H.-G.; Neumann B. Hydroboration of Alkyne-Functionalized 1,3,2-Benzodiazaboroles. Eur. J. Inorg. Chem. 2013, 2013, 2608 10.1002/ejic.201201489.

[ref16] Krautwald S.; Bezdek M. J.; Chirik P. J. Cobalt-Catalyzed 1,1-Diboration of Terminal Alkynes: Scope, Mechanism, and Synthetic Applications. J. Am. Chem. Soc. 2017, 139, 3868 10.1021/jacs.7b00445. 28199104 PMC5697746

[ref17] Dominguez-Molano P.; Bru G.; Salvado O.; Maza R. J.; Carbó J. J.; Fernández E. Transborylation of alkenylboranes with diboranes. Chem. Commun. 2021, 57, 13361 10.1039/D1CC05815K. 34821229

[ref18] aFontani P.; Carboni B.; Vaultier M.; Carrie R. A convenient highly stereoselective synthesis of cyclopropylborontaes. Tetrahedron Lett. 1989, 30, 4815 10.1016/S0040-4039(01)80516-5.

[ref19] France M. B.; Milojevich A. K.; Stitt T. A.; Kim A. J. High trans selectivity in the copper bis(oxazoline)-catalyzed asymmetric cyclopropanation of olefins by (trimethylsilyl)diazomethane. Tetrahedron Lett. 2003, 44, 9287 10.1016/j.tetlet.2003.10.068.

[ref20] Carreras J.; Caballero A.; Pérez P. J. Enantio- and Diastereoselective Cyclopropanation of 1-Alkenylboronates: Synthesis of 1-boryl-2,3-disubstituted Cyclopropanes. Angew. Chem., Int. Ed. 2018, 57, 2334 10.1002/anie.201710415. 29226562

[ref21] Mu Q.-Ch.; Wang X.-B.; Ye F.; Sun Y.-L.; Bai X.-F.; Chen J.; Xia Ch.-G.; Xu L.-W. Palladium-catalyzed olefination of aryl/alkyl halides with trimethylsilyldiazomethane via carbene migratory insertion. Chem. Commun. 2018, 54, 12994 10.1039/C8CC07664B. 30387477

[ref22] See the Supporting Information for the characterization of both structural isomers (**11a** and **11b**) observed in cyclopropanation with 1,1-BpinBhex alkenes with TMSDM.

[ref23] aImai T.; Mineta H.; Nishida S. Asymmetric cyclopropanation of 1-alkenylboronic esters and its application to the synthesis of optically active cyclopropanols. J. Org. Chem. 1990, 55, 4986 10.1021/jo00304a004.

[ref24] Cuenca A. B.; Cid J.; Garcia-Lopez D.; Carbó J. J.; Fernández E. Unsymmetrical 1,1-diborated multisubstituted sp^3^-carbons formed via a metal-free concerted-asynchronous mechanism. Org. Biomol. Chem. 2015, 13, 9659 10.1039/C5OB01523E. 26264986

