# Supplementary material for: Stereoselective Cyclopropanation of 1,1-Diborylalkenes via Palladium-Catalyzed (Trimethylsilyl)diazomethane Insertion
Source: Org Lett. 2022 Jul 7;24(27):4949–53. doi: 10.1021/acs.orglett.2c01885 (PMC9348837; doi:10.1021/acs.orglett.2c01885)
Supplement: Supplementary file 1 — ol2c01885_si_001.pdf [file ol2c01885_si_001.pdf]

Stereoselective cyclopropanation of 1,1-diborylalkenes via palladium catalyzed (trimethylsilyl)diazomethane insertion

Oriol Salvado, [a] Paula Dominguez-Molano, [a] Elena Fernández[a]*

Dept. Química Física i Inorgànica, University Rovira i Virgili, 43007 Tarragona, Spain

Contents

General information

General procedure for the synthesis of tris(4,4,5,5-tetramethyl-1,3,2-dioxaborolan-2-yl)methane (1)

General procedure for the synthesis of the 1,1-diborylalkenes (METHOD A)

General procedure for the synthesis of the 1,1-diborylalkenes (METHOD B)

General procedure for the synthesis of the 1,1-diborylalkene 2g

General procedure for transborylation of 1,1-diborylalkenes with diboron reagents

General procedure for the cyclopropanation of the 1,1-diborylalkenes

General procedure for the oxidation of borylcyclopropanes

Characterization data for 1,1-diborylalkenes

Characterization data for B, B, Si cyclopropanes

Characterization data for B, Si cyclopropanes

Characterization data for cyclopropanol derivatives

Characterization data for vinyl silane

Enantiomeric ratio determined by HPLC

X-ray single-crystal diffraction analysis for 25

References Experimental Part

References for X-Ray single-crystal diffraction analysis for 25

General Information

Solvents and reagents: Solvents and reagents were obtained from commercial suppliers and dried and/or purified (if needed) by standard procedures. Tetrahydrofuran was dried by distillation from sodium benzophenone ketyl. (Trimethylsilyl)diazomethane solution 2.0 M in hexanes, lithium 2,2,6,6-tetramethylpiperidide employed was purchased from Sigma-Aldrich Inc. Bis(pinacolato)diboron was purchased from Ally Chem and used without further purification. All the alkynes were purchased from typical suppliers (Sigma Aldrich, ThermoFisher, Abcr or Apollo Scientific) and were used without further purification. Pinacolborane was purchased from Apollo Scientific. All reactions were conducted in oven and flame-dried glassware under an inert atmosphere of argon, using Schlenk-type techniques. *Flash chromatography* was performed on standard silica gel (Merck Kieselgel 60 F254 400-630 mesh). *Thin layer chromatography* was performed on Merck Kieselgel 60 F254 which was developed using standard visualizing agents: UV fluorescence (254 and 366 nm) or potassium permanganate/Δ. *NMR spectr*a were recorded at a Varian Goku 400 or a Varian Mercury 400 spectrometer. ^{1}H NMR and $^{13}C\{^{1}H\}$ NMR chemical shifts (δ) are reported in ppm with the solvent resonance as the internal standard ($CHCl_3$: 7.26 ppm (1 H)) and ($CDCl_3$: 77.16 ppm (^{13}C). $^{11}B\{^{1}H\}$ NMR chemical shifts (δ) are reported in ppm relative to $(CH_3)_2O\cdots BF_3$. Data are reported as follows: chemical shift, multiplicity (s = singlet, d = doublet, t = triplet, q = quartet, hept = heptuplet, br = broad, m = multiplet), coupling constants (Hz) and integration. *High resolution mass spectra (HRMS)* were recorded using a 6210 Time of Flight (TOF) mass spectrometer from Agilent Technologies (Waldbronn, Germany) with an ESI interface and it was performed at the Servei de Recursos Científics i Tècnics (Universitat Rovira i Virgili, Tarragona) or using a BIOTOF II Time of Flight (TOF) mass spectrometer from Bruker with an APCI interface or EI interface and it was performed at the Unidade de Espectrometría de Masas e Proteómica (Universidade de Santiago de Compostela, Santiago de Compostela). GC-MS analyses were performed on a HP6890 gas chromatograph and an Agilent Technologies 5973 Mass selective detector (Waldbronn, Germany) equipped with an achiral capillary column HP-5 (30m, 0.25mm i. d., 0.25μm thickness) using He as the carrier gas.

General procedure for the synthesis of tris(4,4,5,5-tetramethyl-1,3,2-dioxaborolan-2-yl)methane (1)[1]

Trimethylboroxine (0.42 mmol, 1 equiv) and 2-(1H-pyrazol-5-yl)aniline (1.26 mmol, 3 equiv) were mixed with toluene (30 mL) in a rounded-bottom Schlenk flask and heated (oil bath) under reflux in a Dean-Stark apparatus for 2h. The solvent was evaporated and the resulting mixture was 5-methyl-5,6-dihydrobenzo[e]pyrazolo[1,5-c][1,3,2]diazaborinine (99%, 230 mg) as a pale brownish-white solid. In case the mixture was not pure enough, it was subjected to a bulb-to-bulb distillation. Then, a Schlenk-tube equipped with a magnetic stir bar was charged with 5-methyl-5,6-dihydrobenzo[e]pyrazolo[1,5-c][1,3,2] (1,26 mmol, 1 equiv), the iridium catalyst (0.19 mmol, 0.15 equiv) and B_2pin_2 (5.04 mmol, 4 equiv) in dry THF (2 mL) and the mixture was stirred and heated at 50 °C for 24h. Then pinacol (3.78 mmol, 3 equiv) and p-toluenesulfonic acid monohydrated (3.78 mmol, 3 equiv) were added and the mixture was stirred for 5h at room temperature. The crude was filtered through a pad of celite and washed with Et_2O and purified by silica gel chromatography to afford **1** (42%, 209 mg). The characterization was in agreement with the reported product in the literature.[1]

General procedure for the synthesis of the 1,1-diborylalkenes (METHOD A)

A Schlenk-tube equipped with a magnetic stir bar was charged with triborylmethane **1** (0.3 mmol, 1 equiv) and LiTMP (0.36 mmol, 1.2 equiv) in dry THF (2 mL). The mixture was stirred for 30 minutes at 0 °C. Then the corresponding aldehyde (0.45 mmol, 1.5 equiv) was added and the reaction was stirred at 0 °C for 10 minutes, followed by 16 h at room temperature. The crude residue was purified by silica gel chromatography to afford the desired products **2a**, **2c**, **2f**, **2h**.

General procedure for the synthesis of the 1,1-diborylalkenes (METHOD B)

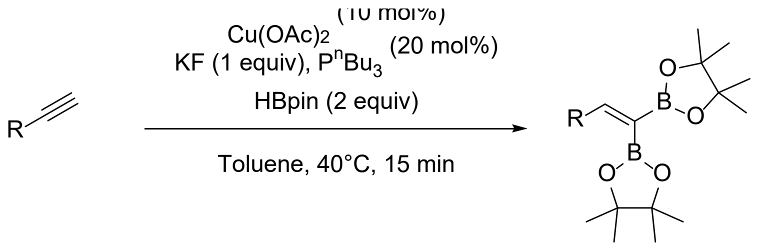

A Schlenk-tube equipped with a magnetic stir bar was charged with $Cu(OAc)_2$ (10 mol%, 36 mg, 0.2 mmol), KF (1 equiv, 116 mg, 2 mmol) in dry toluene as solvent (2.5 mL). Then, P^nBu_3 (81 mg, 99 µL, 0.4 mmol), the corresponding alkyne (204 mg, 0.22 mL, 2 mmol), and HBpin (512 mg, 0.58 mL, 4 mmol) were added in this order. The mixture was stirred for 15 min at 40 °C (oil bath). The

reaction mixture was then diluted with Et_2O and filtered through a plug of celite in the air with copious washing (Et_2O). The solvent was gently concentrated at the rotary evaporator and the crude residue was purified by silica gel flash chromatography to afford the desired products **2b**, **2c**, **2d**, **2e**, **2f'**, **2i**, **2j**.

General procedure for the synthesis of the 1,1-diborylalkene 2g[2]

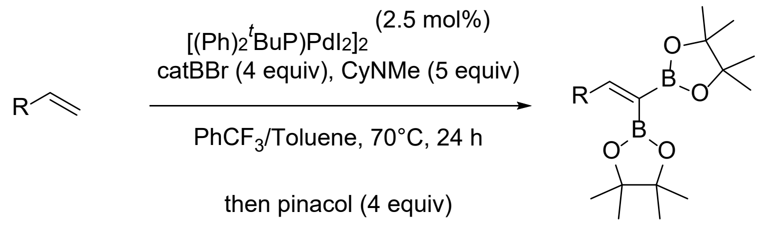

A Schlenk tube equipped with a magnetic stir bar was charged with $[(Ph)_2{}^tBuP)PdI_2]_2$[3] (41 mg, 2.5 mol%) in dry trifluorotoluene (5 mL) and the solution was stirred at rt for 10 minutes. *N*,*N*-dicyclohexylmethylamine (1.1 mL, 5 equiv), a solution of *B*-bromocatecholborane in toluene (5 mL, ~0.8 M, 4 equiv), and the alkene (1 mmol, 1 equiv) were added sequentially. The reaction was stirred in an oil bath at 70 °C (oil bath) for 24 h. Subsequently, the flask was cooled to room temperature, opened to air, and charged with pinacol (472 mg, 4 equiv), ammonium pyrrolidine-dithiocarbamate (50 mg, palladium scavenger, 6 equiv to palladium), and diluted with Et_2O (10 mL). The reaction was stirred at rt for 1 h. Then, the reaction was filtered through a pad of celite and concentrated *in vacuo* at 40 °C to remove all solvents. The resultant crude oil was diluted with Et_2O (20 mL) and washed with 1 M aqueous HCl (3 x 20mL) to remove the excess of amine. The organic layer was dried with $MgSO_4$, filtered through Celite, and concentrated *in vacuo*. The solvent was gently concentrated at the rotary evaporator and the NMR yield was calculated through comparison to an internal standard (naphthalene). The crude residue was purified by silica gel flash chromatography to afford the desired product **2g**.

General procedure for transborylation of 1,1-diborylalkenes with diboron reagents[4]

A Schlenk-tube equipped with a magnetic stir bar was charged with alkenylborane (0.3 mmol, 1 equiv), diboron reagent (0.6 mmol, 2 equiv) in dry MeOH as solvent (2 mL). The mixture was stirred during 16 h at 90 °C (oil bath). The solvent was gently concentrated at the rotary evaporator and the NMR yield was calculated by comparison to an internal standard (naphthalene). The crude residue was purified by silica gel flash chromatography to afford the desired product.

General procedure for the cyclopropanation of the 1,1-diborylalkenes (1mmol scale)

In a glove box, a Schlenk-tube equipped with a magnetic stir bar was charged with $Pd(OAc)_2$ (15 mol%). Then the 1,1-diborylakene substrate (0.2 mmol, 1 equiv) and hexane (1mL) were added. The mixture was stirred for 5 minutes and the (diazomethyl)trimethylsilane 2M (4.2 mmol, 7 equiv) was added dropwise and the reaction was stirred for 5 minutes in the glovebox. The Schlenck-tube was closed with a Teflon cap and was allowed to react for 16h, at room temperature, with agitation. The mixture was flirted through Celite and washed with Et_2O. The solvent was evaporated and the crude was purified by silica gel chromatography.

General procedure for the cyclopropanation of the 1,1-diborylalkenes towards 5 (1mmol scale)

In a glove box, a Schlenk-tube equipped with a magnetic stir bar was charged with $Pd(OAc)_2$ (15 mol%). Then the 2,2'-(2-phenylethene-1,1-diyl)bis(4,4,5,5-tetramethyl-1,3,2-dioxaborolane) (**2b**) (1 mmol, 1 equiv) and hexane (1 mL) were added. The mixture was stirred for 5 minutes and the (diazomethyl)trimethylsilane 2M (7 mmol, 7 equiv) was added dropwise and the reaction was stirred for 5 minutes in the glovebox. The Schlenck-tube was closed with a Teflon cap and was allowed to react for 16h, at room temperature, with agitation. The mixture was flirted through Celite and washed with Et_2O. The solvent was evaporated and the crude was purified by silica gel chromatography (pentane:ethyl ether = 100:3) getting **5** (79%, 350 mg) as a pale yellowish solid.

General procedure for the protodeborylation of 1,1-diborylcyclopropanes

A Schlenk-tube equipped with a magnetic stir bar was charged with NaO^tBu (0.6 mmol, 3 equiv), the corresponding B, B, Si, cyclopropane (0.2 mmol, 1 equiv) and THF (2 mL). The Schlenk-tube was closed with a Teflon cap and the reaction was stirred for 16h at 60 °C (oil bath). The solvents were evaporated at the rotatory evaporator and the crude was purified by silica gel chromatography to obtain the desired product.

General procedure for the oxidation of borylcyclopropanes

In a opened-air flask, charged with a magnetic stir bar, were added the corresponding B, Si cyclopropane (0.1 mmol, 1 equiv), $NaBO_3 \cdot H_2O$ (0.3 mmol, 3 equiv), THF (2 mL) and distilled water (1 mL). The reaction was closed with a septum with a needle to avoid over pressures and was stirred for 16h at room temperature. After this period of time, the mixture was extracted with Et_2O (3 x 15 mL), the organic layer was dried with anhydrous magnesium sulphate, filtered and the solvents were evaporated. The resulting crude was purified by silica gel chromatography to obtain the corresponding alcohol.

Characterization data for 1,1-diborylalkenes

2,2'-(3-phenylprop-1-ene-1,1-diyl)bis(4,4,5,5-tetramethyl-1,3,2-dioxaborolane) (2a)

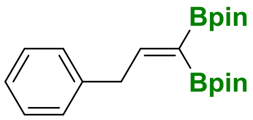

2a

Purified by flash column chromatography (pentane:ethyl ether = 100:3) yielded **2a** (Method A: 62%, 55 mg) as a colourless oil. Spectral data are in agreement with the literature[5]

2,2'-(2-phenylethene-1,1-diyl)bis(4,4,5,5-tetramethyl-1,3,2-dioxaborolane) (2b)

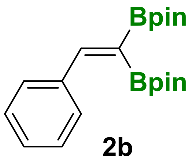

2b

Purified by flash column chromatography (pentane:ethyl ether = 100:3) yielded **2b** (Method B: 58%, 410 mg) as a pale yellowish oil.

^{1}H NMR ($CDCl_3$, 400 MHz) δ 7.71 (s, 1H), 7.51 – 7.44 (m, 2H), 7.34 – 7.27 (m, 3H), 1.31 (s, 12H), 1.28 (s, 12H).

^{13}C NMR ($CDCl_3$, 100 MHz) δ 155.1, 139.6, 128.4, 128.2, 128.1, 83.6, 83.2, 24.8, 24.6.

^{11}B NMR ($CDCl_3$, 128.3 MHz) δ 30.8.

HRMS (ESI) for **$C_{20}H_{30}B_2O_4$ [M+H]$^+$:** calculated: 371.2568, found: 371.2567.

2,2'-(2-(p-tolyl)ethene-1,1-diyl)bis(4,4,5,5-tetramethyl-1,3,2-dioxaborolane) (2c)

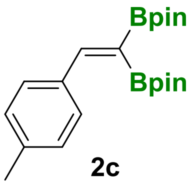

2c

Purified by flash column chromatography (pentane:ethyl ether = 100:3) yielded **2c** (Method A: 41%, 36 mg; Method B: 52%, 240 mg) as a pale yellowish viscous oil.

^{1}H NMR ($CDCl_3$, 400 MHz) δ 7.60 (s, 1H), 7.31 (d, J = 8.1 Hz, 2H), 7.02 (d, J = 8.1 Hz, 2H), 2.25 (s, 3H), 1.25 (s, 12H), 1.20 (s, 12H).

^{13}C NMR ($CDCl_3$, 100 MHz) δ 155.1, 138.4, 136.8, 128.8, 128.2, 83.5, 83.1, 24.8, 24.6, 21.3.

^{11}B NMR ($CDCl_3$, 128.3 MHz) δ 30.9.

HRMS (ESI) for **$C_{21}H_{36}NB_2O_4^+$[M+NH$_4^+$]$^+$:** calculated: 388.2830, found: 388.2832

2,2'-(2-(4-methoxyphenyl)ethene-1,1-diyl)bis(4,4,5,5-tetramethyl-1,3,2-dioxaborolane) (2d)

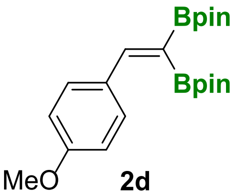

2d

Purified by flash column chromatography (pentane:ethyl ether = 100:3) yielded **2d** (Method B: 35%, 270 mg) as a pale yellowish viscous oil.

^{1}H NMR ($CDCl_3$, 400 MHz) δ 7.65 (s, 1H), 7.48 – 7.40 (m, 2H), 6.86 – 6.78 (m, 2H), 3.80 (s, 3H), 1.32 (s, 12H), 1.27 (s, 12H).

^{13}C NMR ($CDCl_3$, 100 MHz) δ 159.9, 154.8, 132.4, 129.7, 113.5, 83.5, 83.0, 55.2, 24.8, 24.7.

^{11}B NMR ($CDCl_3$, 128.3 MHz) δ 31.3.

HRMS (ESI) for **$C_{21}H_{36}NB_2O_5$ [M+NH_4^+]$^+$:** calculated: 404.2779, found: 404.2782.

2,2'-(2-(4-fluorophenyl)ethene-1,1-diyl)bis(4,4,5,5-tetramethyl-1,3,2-dioxaborolane) (2e)

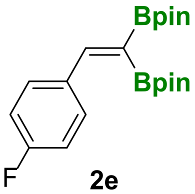

2e

Purified by flash column chromatography (pentane:ethyl ether = 100:3) yielded **2e** (Method B: 44%, 210 mg) as a pale yellowish solid.

^{1}H NMR ($CDCl_3$, 400 MHz) δ 7.66 (s, 1H), 7.49 – 7.43 (m, 2H), 6.98 (m, 2H), 1.31 (s, 12H), 1.27 (s, 12H).

^{13}C NMR ($CDCl_3$, 100 MHz) δ 153.8, 129.9, 129.8, 115.2, 114.9, 83.6, 83.2, 24.8, 24.6.

^{11}B NMR ($CDCl_3$, 128.3 MHz) δ 30.8.

HRMS (ESI) for **$C_{20}H_{29}B_2FO_4$ [M+H^+]$^+$:** calculated: 375.2210, found: 375.2215.

Melting point: 78.4 °C

2,2'-(2-(2-bromophenyl)ethene-1,1-diyl)bis(4,4,5,5-tetramethyl-1,3,2-dioxaborolane) (2f)

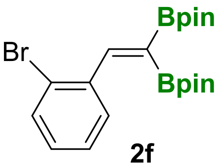

2f

Purified by flash column chromatography (pentane:ethyl ether = 100:3) yielded **2f** (Method A: 68%, 62 mg) as a pale yellowish viscous oil.

^{1}H NMR ($CDCl_3$, 400 MHz) δ 7.77 (s, 1H), 7.48 (d, J = 7.8 Hz, 1H), 7.44 (d, J = 7.8 Hz, 1H), 7.13 (t, J = 7.7 Hz, 1H), 7.03 (t, J = 7.7 Hz, 1H), 1.20 (s, 12H), 1.16 (s, 12H).

^{13}**C NMR** ($CDCl_3$, 100 MHz) δ 153.9, 139.8, 132.6, 129.5, 129.3, 126.9, 123.9, 83.6, 83.3, 24.8, 24.5.

^{11}**B NMR** ($CDCl_3$, 128.3 MHz) δ 30.2.

HRMS (ESI) for $\mathbf{C_{20}H_{35}B_2NBrO_4^+}$ **[M+NH$_4^+$]$^+$:** calculated: 454.1936, found: 454.1929.

2,2'-(2-(2-chlorophenyl)ethene-1,1-diyl)bis(4,4,5,5-tetramethyl-1,3,2-dioxaborolane) (2f')

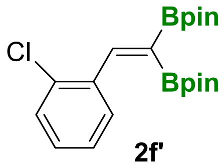

2f'

Purified by flash column chromatography (pentane:ethyl ether = 100:3) yielded **2f'** (Method B: 62%, 48 mg) as a pale yellowish oil.

^{1}**H NMR** ($CDCl_3$, 400 MHz) δ 7.92 (s, 1H), 7.60 – 7.54 (m, 1H), 7.35 – 7.29 (m, 1H), 7.23 – 7.11 (m, 2H), 1.28 (s, 12H), 1.25 (s, 12H).

^{13}**C NMR** ($CDCl_3$, 100 MHz) δ 151.7, 138.0, 133.8, 129.4, 129.4, 129.2, 126.2, 83.6, 83.3, 24.9, 24.5.

^{11}**B NMR** ($CDCl_3$, 128.3 MHz) δ 30.9.

HRMS (ESI) for $\mathbf{C_{20}H_{29}B_2ClO_4}$ **[M+H]$^+$:** calculated: 391.2017, found: 391.2021.

2,2'-(2-(Naphthalen-2-yl)ethene-1,1-diyl)bis(4,4,5,5-tetramethyl-1,3,2-dioxaborolane) (2g)

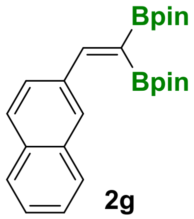

2g

Purified by flash column chromatography (pentane: diethyl ether = 10:1) yielded **2g** (57%, 47 mg) as pale yellowish oil.

^{1}**H NMR** ($CDCl_3$, 400 MHz) δ 7.95 (s, 1H), 7.87 (s, 1H), 7.84 – 7.73 (m, 3H), 7.63 (dd, J = 8.5, 1.8 Hz, 1H), 7.50 – 7.40 (m, 2H), 1.34 (s, 12H), 1.30 (s, 12H).

^{13}**C NMR** ($CDCl_3$, 100 MHz) δ 155.1, 137.2, 133.3, 133.2, 128.3, 127.7, 127.7, 127.6, 126.2, 126.1, 125.9, 83.7, 83.2, 24.9, 24.7.

^{11}**B NMR** ($CDCl_3$, 128.3 MHz) δ 31.8.

HRMS (ESI) for $\mathbf{C_{24}H_{33}B_2O_4}$ **[M+H]$^+$:** calculated: 407.2567, found: 407.2567

2,2'-(2-cyclohexylethene-1,1-diyl)bis(4,4,5,5-tetramethyl-1,3,2-dioxaborolane) (2h)

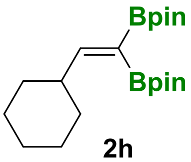

2h

Purified by flash column chromatography (pentane:ethyl ether = 100:3) yielded **2h** (Method A: 47%, 41 mg) as a pale yellowish oil.

^{1}H NMR ($CDCl_3$, 400 MHz) δ 6.73 (d, J = 8.8 Hz, 1H), 2.29 – 2.20 (m, 1H), 1.70 (dd, J = 8.8, 6.7 Hz, 4H), 1.64 – 1.57 (m, 2H), 1.28 (s, 12H), 1.21 (s, 12H), 1.18 – 1.03 (m, 4H).

^{13}C NMR ($CDCl_3$, 100 MHz) δ 166.0, 82.0, 81.7, 43.1, 31.6, 24.9, 24.8, 23.8, 23.6.

^{11}B NMR ($CDCl_3$, 128.3 MHz) δ 30.7.

HRMS (ESI) for **$C_{20}H_{40}NB_2O_4^+$[M+NH$_4^+$]$^+$:** calculated: 380.3143, found: 380.3149

2,2'-(2-(cyclohex-1-en-1-yl)ethene-1,1-diyl)bis(4,4,5,5-tetramethyl-1,3,2-dioxaborolane) (2i)

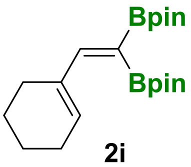

2i

Purified by flash column chromatography (pentane:ethyl ether = 100:3) yielded **2i** (Method B: 34%, 250 mg) as a pale yellowish solid.

^{1}H NMR ($CDCl_3$, 400 MHz) δ 7.18 (s, 1H), 6.06 – 5.94 (m, 1H), 2.25 – 2.16 (m, 2H), 2.18 – 2.07 (m, 2H), 1.72 – 1.50 (m, 4H), 1.30 (s, 12H), 1.22 (s, 12H).

^{13}C NMR ($CDCl_3$, 100 MHz) δ 158.1, 138.9, 135.2, 83.5, 82.9, 26.3, 25.8, 24.9, 24.8, 24.8, 22.3, 22.1.

^{11}B NMR ($CDCl_3$, 128.3 MHz) δ 30.8.

HRMS (ESI) for **$C_{20}H_{40}NB_2O_4^+$[M+NH$_4^+$]$^+$:** calculated: 380.3143, found: 380.3149

Melting point: 42.3 °C

2,2'-(2-(thiophen-3-yl)ethene-1,1-diyl)bis(4,4,5,5-tetramethyl-1,3,2-dioxaborolane) (2j)

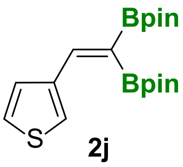

2j

Purified by flash column chromatography (pentane: diethyl ether = 100:3) yielded **2j** (35%, 26 mg) as pale yellowish oil.

^{1}H NMR ($CDCl_3$, 400 MHz) δ 7.64 (s, 1H), 7.44 (m, 1H), 7.31 (dd, *J* = 5.1, 1.3 Hz, 1H), 7.22 (ddd, *J* = 5.1, 3.0, 1.5 Hz, 1H), 1.33 (s, 12H), 1.27 (s, 12H).

^{13}C NMR ($CDCl_3$, 100 MHz) δ 148.2, 142.5, 127.2, 125.6, 125.3, 83.6, 83.1, 24.8, 24.8.

^{11}B NMR ($CDCl_3$, 128.3 MHz) δ 31.9.

HRMS (ESI) for **$C_{18}H_{29}B_2O_4S$ [M+H]$^+$:** calculated: 363.1972, found: 363.1973.

(*Z*)-4,4,6-trimethyl-2-(2-phenyl-1-(4,4,5,5-tetramethyl-1,3,2-dioxaborolan-2-yl)vinyl)-1,3,2-dioxaborinane (3a)

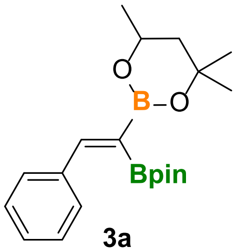

3a

Purified by flash column chromatography (hexane:diethyl ether = 100:1) yielded **3a** (65%, 47 mg) as a pale yellowish oil.

^{1}H NMR ($CDCl_3$, 400 MHz) δ 7.66 (s, 1H), 7.53 – 7.41 (m, 2H), 7.32 – 7.19 (m, 3H), 4.26 (m, 1H), 1.76 (dd, J = 13.8, 3.0 Hz, 1H), 1.53 (dd, J = 13.9, 5.6 Hz, 1H), 1.31 (s, 12H), 1.30 (s, 6H), 1.27 – 1.24 (m, 3H).

^{13}C NMR ($CDCl_3$, 100 MHz) δ 152.5, 140.1, 128.0, 128.0, 127.9, 83.3, 70.8, 64.8, 45.8, 31.2, 28.1, 24.8, 24.7, 23.1.

^{11}B NMR ($CDCl_3$, 128.3 MHz) δ 26.1.

HRMS (ESI) for **$C_{20}H_{30}B_2O_4$ [M+H]$^+$:** calculated: 357.2409, found: 357.2410.

(*Z*)-2-(2-(4-methoxyphenyl)-1-(4,4,5,5-tetramethyl-1,3,2-dioxaborolan-2-yl)vinyl)-4,4,6-trimethyl-1,3,2-dioxaborinane (3b)

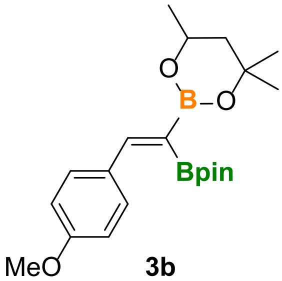

3b

Purified by flash column chromatography (hexane:diethyl ether = 100:1) yielded **3b** (77%, 60 mg) as a pale yellowish solid.

^{1}**H NMR** ($CDCl_3$, 400 MHz) δ 7.60 (s, 1H), 7.46 – 7.39 (m, 2H), 6.85 – 6.75 (m, 2H), 4.24 (m, 1H), 3.79 (s, 3H), 1.78 – 1.70 (m, 1H), 1.56 – 1.47 (m, 2H), 1.32 (s, 12H), 1.29 (s, 6H), 1.26 (s, 3H).

^{13}**C NMR** ($CDCl_3$, 100 MHz) δ 159.5, 152.1, 133.0, 129.5, 113.3, 83.3, 70.7, 64.7, 55.2, 45.8, 31.3, 28.1, 24.9, 24.7, 23.1.

^{11}**B NMR** ($CDCl_3$, 128.3 MHz) δ 26.1.

HRMS (ESI) for $\mathbf{C_{21}H_{32}B_2O_5}$ **[M+H]$^+$:** calculated: 386.0966, found: 386.0959.

Melting point: 62.4 °C

(*Z*)-2-(2-(4-Fluorophenyl)-1-(4,4,5,5-tetramethyl-1,3,2-dioxaborolan-2-yl)vinyl)-4,4,6-trimethyl-1,3,2-dioxaborinane (3c)

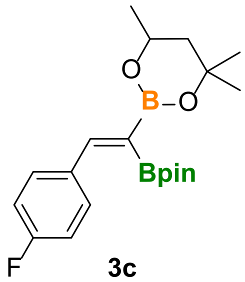

3c

Purified by flash column chromatography (hexane:diethyl ether = 100:1) yielded **3c** (72%, 54 mg) as a pale yellowish solid.

^{1}**H NMR** ($CDCl_3$, 400 MHz) δ 7.61 (s, 1H), 7.47 – 7.41 (m, 2H), 6.99 – 6.92 (m, 2H), 4.25 (m, 1H), 1.76 (dd, J = 13.8, 2.9 Hz, 1H), 1.56 – 1.47 (m, 2H), 1.31 (s, 12H), 1.30 (s, 6H), 1.26 (d, J = 6.1 Hz, 3H).

^{13}**C NMR** ($CDCl_3$, 100 MHz) δ 151.2, 129.7, 129.7, 115.0, 114.8, 83.4, 70.9, 64.8, 45.8, 31.2, 28.1, 24.8, 24.7, 23.0.

^{11}**B NMR** ($CDCl_3$, 128.3 MHz) δ 32.5, 26.7.

HRMS (ESI) for $\mathbf{C_{20}H_{30}B_2FO_4}$ **[M+H]$^+$:** calculated: 375.2314, found: 375.2311.

Melting point: 71.4°C.

(*Z*)-4,4,6-trimethyl-2-(2-(naphthalen-2-yl)-1-(4,4,5,5-tetramethyl-1,3,2-dioxaborolan-2-yl)vinyl)-1,3,2-dioxaborinane (3d)

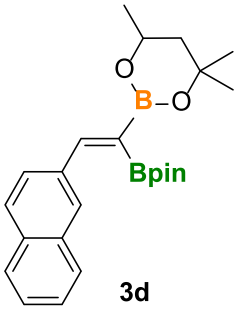

3d

Purified by flash column chromatography (hexane:diethyl ether = 100:1) yielded **3d** (77%, 63 mg) as a pale yellowish solid.

^{1}H NMR ($CDCl_3$, 400 MHz) δ 7.94 (d, J = 1.7 Hz, 1H), 7.83 – 7.72 (m, 4H), 7.63 (dd, J = 8.5, 1.8 Hz, 1H), 7.47 – 7.41 (m, 2H), 4.28 (m, 1H), 1.78 (dd, J = 13.8, 3.0 Hz, 1H), 1.55 (s, 1H), 1.33 (s, 12H), 1.32 (s, 6H), 1.28 (d, J = 6.2 Hz, 3H).

^{13}C NMR ($CDCl_3$, 100 MHz) δ 152.4, 137.8, 133.3, 133.1, 128.2, 127.6, 127.5, 127.3, 126.1, 125.9, 125.9, 83.4, 70.9, 64.9, 45.8, 31.3, 28.2, 24.9, 24.8, 23.1.

^{11}B NMR ($CDCl_3$, 128.3 MHz) δ 33.6, 27.0.

HRMS (ESI) for **$C_{24}H_{33}B_2O_4$ $[M+H]^+$:** calculated: 407.2566, found: 407.2568.

Melting point: 63.4 °C

(*Z*)-2-(2-(4-methoxyphenyl)-1-(4,4,5,5-tetramethyl-1,3,2-dioxaborolan-2-yl)vinyl)-5,5-dimethyl-1,3,2-dioxaborinane (3e)

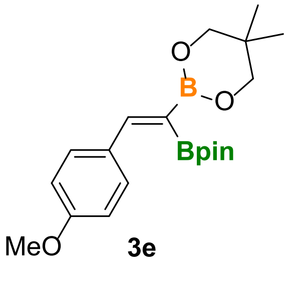

3e

Purified by flash column chromatography (hexane:diethyl ether = 100:1) yielded **3e** (57%, 42 mg) as a pale yellowish solid.

^{1}H NMR ($CDCl_3$, 400 MHz) δ 7.61 (s, 1H), 7.43 (d, J = 8.8 Hz, 2H), 6.87 – 6.78 (m, 2H), 3.80 (s, 3H), 3.66 (s, H), 1.32 (s, 12H), 0.98 (s, 6H).

^{13}C NMR ($CDCl_3$, 100 MHz) δ 159.7, 152.5, 129.6, 113.4, 83.4, 72.2, 55.2, 31.6, 24.7, 22.6, 22.0, 14.1.

^{11}B NMR ($CDCl_3$, 128.3 MHz) δ 31.7, 26.9.

HRMS (ESI) for **$C_{20}H_{31}B_2O_5$ [M+H]$^+$:** calculated: 373.2357, found: 373.2360.

Melting point: 61.2 °C

(*Z*)-5,5-Dimethyl-2-(2-(naphthalen-2-yl)-1-(4,4,5,5-tetramethyl-1,3,2-dioxaborolan-2-yl)vinyl)-1,3,2-dioxaborinane (3f)

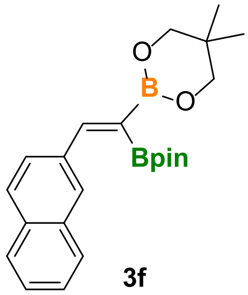

3f

Purified by flash column chromatography (hexane:diethyl ether = 100:1) yielded **3f** (71%, 55 mg) as a pale yellowish solid.

^{1}H NMR ($CDCl_3$, 400 MHz) δ 7.96 – 7.94 (m, 1H), 7.86 – 7.71 (m, 3H), 7.63 (dd, J = 8.5, 1.8 Hz, 1H), 7.48 – 7.41 (m, 2H), 3.69 (s, 4H), 1.33 (s, 12H), 1.00 (s, 6H).

^{13}C NMR ($CDCl_3$, 100 MHz) δ 152.9, 137.5, 133.3, 133.2, 128.2, 127.6, 127.4, 126.1, 126.0, 126.0, 83.5, 72.3, 31.8, 24.8, 22.0.

^{11}B NMR ($CDCl_3$, 128.3 MHz) δ 31.6, 27.1.

HRMS (ESI) for **$C_{23}H_{31}B_2O_4$ [M+H]$^+$:** calculated: 393.2408, found: 393.2409.

Melting point: 70.1 °C

(*E*)-2-(2-(4-Methoxyphenyl)-1-(4,4,5,5-tetramethyl-1,3,2-dioxaborolan-2-yl)vinyl)-3a,5,5-trimethylhexahydro-4,6-methanobenzo[d][1,3,2]dioxaborole (3g)

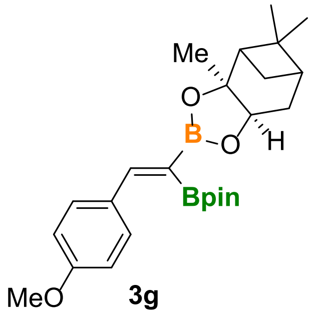

3g

Purified by flash column chromatography (pentane: diethyl ether = 10:1) yielded **3g** (35%, 32 mg) as pale yellowish solid.

^{1}H NMR ($CDCl_3$, 400 MHz) δ 7.68 (s, 1H), 7.49 – 7.41 (m, 2H), 6.86 – 6.78 (m, 2H), 4.34 (dd, J = 8.7, 1.9 Hz, 1H), 3.80 (s, 3H), 2.34 (dddd, J = 14.5, 8.7, 3.7, 2.1 Hz, 1H), 2.25 – 2.15 (m, 1H), 2.10

– 2.03 (m, 1H), 1.96 – 1.80 (m, 2H), 1.41 (s, 3H), 1.33 (s, 12H), 1.29 (s, 3H), 1.27 – 1.21 (m, 1H), 0.85 (s, 3H).

^{13}C NMR ($CDCl_3$, 100 MHz) δ 159.9, 155.0, 132.4, 129.8, 113.5, 85.4, 83.5, 77.8, 55.2, 51.4, 39.5, 38.1, 35.6, 28.6, 27.1, 26.4, 24.8, 24.6, 24.0.

^{11}B NMR ($CDCl_3$, 128.3 MHz) δ 31.4.

HRMS (ESI) for **$C_{25}H_{36}B_2O_5$ [M+H]$^+$:** calculated: 439.1726, found: 439.1724.

Melting point: 75.8°C

(*Z*)-3a,5,5-trimethyl-2-(2-(naphthalen-2-yl)-1-(4,4,5,5-tetramethyl-1,3,2-dioxaborolan-2-yl)vinyl)hexahydro-4,6-methanobenzo[d][1,3,2]dioxaborole(3h)

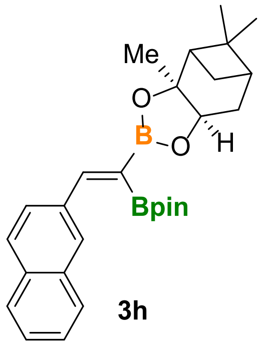

3h

Purified by flash column chromatography (hexane:diethyl ether = 100:1) yielded **3h** (42%, 39 mg) as a pale yellowish solid.

^{1}H NMR ($CDCl_3$, 400 MHz) δ 7.96 (t, J = 2.1 Hz, 1H), 7.90 (s, 1H), 7.81 – 7.74 (m, 3H), 7.64 (dt, J = 8.7, 1.9 Hz, 1H), 7.47 – 7.42 (m, 2H), 4.38 (dd, J = 8.7, 1.9 Hz, 1H), 2.33 – 2.26 (m, 1H), 2.21 – 2.16 (m, 1H), 2.10 – 2.05 (m, 1H), 1.93 – 1.88 (m, 2H), 1.44 (s, 3H), 1.34 (s, 12H), 1.12 (d, J = 10.9 Hz, 2H), 0.90 (s, 3H).

^{13}C NMR ($CDCl_3$, 100 MHz) δ 155.3, 137.2, 133.4, 133.2, 128.3, 127.7, 127.7, 127.6, 126.2, 126.1, 125.9, 85.6, 83.7, 77.9, 51.4, 38.1, 35.6, 34.1, 28.6, 27.1, 26.5, 24.8, 24.7, 24.0, 22.3, 14.0.

^{11}B NMR ($CDCl_3$, 128.3 MHz) δ 29.2.

HRMS (ESI) for **$C_{28}H_{36}B_2O_4$ [M+H]$^+$:** calculated: 459.2876, found: 459.2882.

Melting point: 83.0°C

2-((*Z*)-1-((4*S*,5*R*)-4,5-diphenyl-1,3,2-dioxaborolan-2-yl)-2-(4-methoxyphenyl)vinyl)-4,4,5,5-tetramethyl-1,3,2-dioxaborolane (3i)

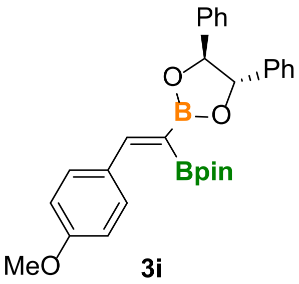

3i

Purified by flash column chromatography (pentane:ethyl ether = 10:1) yielded **3i** (49%, 47 mg) as a pale yellowish oil.

^{1}H NMR ($CDCl_3$, 400 MHz) δ 7.94 (s, 1H), 7.57 – 7.48 (m, 2H), 7.44 – 7.28 (m, 10H), 6.87 (d, J = 8.8 Hz, 2H), 5.20 (s, 2H), 3.83 (s, 3H), 1.35 (d, J = 2.3 Hz, 12H).

^{13}C NMR ($CDCl_3$, 100 MHz) δ 160.3, 157.1, 140.7, 130.0, 128.6, 128.1, 125.7, 113.6, 86.4, 83.8, 55.3, 24.9, 24.7, 24.7.

^{11}B NMR ($CDCl_3$, 128.3 MHz) δ 31.8.

HRMS (ESI) for **$C_{29}H_{33}B_2O_5^+$[M+H$^+$]$^+$:** calculated: 483.2512, found: 483.2513.

2-((*Z*)-1-((4*S*,5*R*)-4,5-diphenyl-1,3,2-dioxaborolan-2-yl)-2-(naphthalen-2-yl)vinyl)-4,4,5,5-tetramethyl-1,3,2-dioxaborolane (3j)

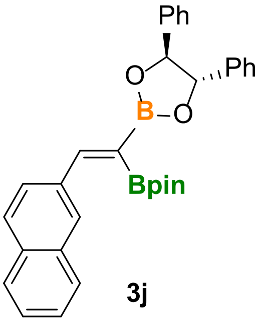

3j

Purified by flash column chromatography (pentane:ethyl ether = 10:1) yielded **3j** (82%, 82 mg) as a pale yellowish oil.

^{1}H NMR ($CDCl_3$, 400 MHz) δ 8.15 (s, 1H), 8.05 – 8.00 (m, 1H), 7.86 – 7.77 (m, 3H), 7.70 (dd, J = 8.6, 1.8 Hz, 1H), 7.51 – 7.44 (m, 2H), 7.44 – 7.32 (m, 10H), 5.25 (s, 2H), 1.37 (s, 16H), 1.36 (s, 16H).

^{13}C NMR ($CDCl_3$, 100 MHz) δ 157.4, 140.6, 128.7, 128.1, 128.1, 126.9, 125.7, 86.5, 84.0, 79.1, 25.0, 24.7.

^{11}B NMR ($CDCl_3$, 128.3 MHz) δ 33.1.

HRMS (ESI) for **$C_{32}H_{32}B_2O_4Na^+$[M+Na]$^+$:** calculated: 525.2384, found: 525.2387.

Characterization data for B, B, Si cyclopropanes

(3-benzyl-2,2-bis(4,4,5,5-tetramethyl-1,3,2-dioxaborolan-2-yl)cyclopropyl)trimethylsilane (4)

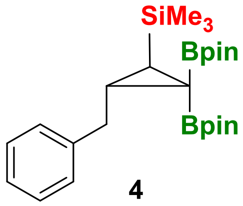

4

Purified by flash column chromatography (pentane:ethyl ether = 100:3) yielded **4** (53%, 48 mg) as a pale yellowish solid.

^{1}H NMR ($CDCl_3$, 400 MHz) δ 7.28 – 7.24 (m, 4H), 7.19 – 7.15 (m, 1H), 3.16 (dd, J = 14.3, 4.4 Hz, 1H), 2.34 (dd, J = 14.3, 9.5 Hz, 1H), 1.44 – 1.37 (m, 1H), 1.25 (s, 6H), 1.23 (s, 6H), 1.23 (s, 6H), 1.21 (s, 6H), 0.21 (d, J = 6.7 Hz, 1H), -0.07 (s, 9H).

^{13}C NMR ($CDCl_3$, 100 MHz) δ 142.9, 128.6, 128.2, 125.7, 83.2, 83.0, 40.0, 28.3, 25.5, 25.3, 25.2, 24.5, 17.3, 0.7, -0.8.

^{11}B NMR ($CDCl_3$, 128.3 MHz) δ 33.1

HRMS (ESI) for **$C_{25}H_{43}B_2O_4Si^+[M+H^+]^+$:** calculated: 457.3116, found: 457.3118.

Melting point: 73.7 °C

trimethyl(3-phenyl-2,2-bis(4,4,5,5-tetramethyl-1,3,2-dioxaborolan-2-yl)cyclopropyl)silane (5)

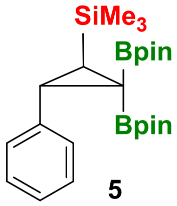

5

Purified by flash column chromatography (pentane:ethyl ether = 100:3) yielded **5** (79%, 70 mg) as a pale yellowish solid

^{1}H NMR ($CDCl_3$, 400 MHz) δ 7.31 – 7.27 (m, 2H), 7.23 – 7.15 (m, 2H), 7.13 – 7.04 (m, 1H), 2.46 (d, J = 7.2 Hz, 1H), 1.27 (s, 6H), 1.25 (s, 6H), 0.97 (s, 6H), 0.92 (d, J = 7.2 Hz, 1H), 0.81 (s, 6H), 0.10 (s, 9H).

^{13}C NMR ($CDCl_3$, 100 MHz) δ 142.2, 129.0, 127.7, 125.8, 83.1, 82.9, 30.6, 25.3, 25.1, 25.1, 24.2, 14.2, -0.6.

^{11}B NMR ($CDCl_3$, 128.3 MHz) δ 31.9.

HRMS (ESI) for **$C_{24}H_{41}B_2O_4Si^+[M+H^+]^+$:** calculated: 443.2960, found: 443.2966.

Melting point: 74.7 °C

2,2-bis(4,4,5,5-tetramethyl-1,3,2-dioxaborolan-2-yl)-3-(p-tolyl)cyclopropyl)trimethylsilane (6)

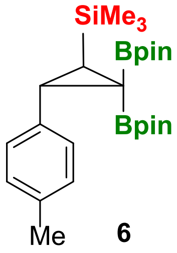

6

Purified by flash column chromatography (pentane:ethyl ether = 10:1) yielded **6** (67%, 69 mg) as a pale yellowish solid

^{1}H NMR ($CDCl_3$, 400 MHz) δ 7.17 (d, J = 8.0 Hz, 2H), 6.99 (d, J = 8.0 Hz, 2H), 2.42 (d, J = 7.2 Hz, 1H), 2.26 (s, 3H), 1.26 (s, 6H), 1.25 (s, 6H), 0.98 (s, 6H), 0.88 (d, J = 7.2 Hz, 1H), 0.83 (s, 6H), 0.10 (s, 9H).

^{13}C NMR ($CDCl_3$, 100 MHz) δ 139.1, 135.2, 128.9, 128.4, 83.1, 82.9, 30.3, 25.3, 25.2, 25.1, 24.2, 21.1, 14.3, -0.6.

^{11}B NMR ($CDCl_3$, 128.3 MHz) δ 31.9.

HRMS (ESI) for **$C_{25}H_{43}B_2O_4Si^+[M+H^+]^+$:** calculated: 457.3117, found: 457.3118.

Melting point: 75.2°C

(3-(4-methoxyphenyl)-2,2-bis(4,4,5,5-tetramethyl-1,3,2-dioxaborolan-2-yl)cyclopropyl)trimethylsilane (7)

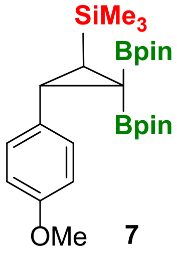

7

Purified by flash column chromatography (pentane:ethyl ether = 100:3) yielded **7** (76%, 63 mg) as a pale yellowish solid

^{1}H NMR ($CDCl_3$, 400 MHz) δ 7.23 – 7.17 (m, 2H), 6.78 – 6.70 (m, 2H), 3.75 (s, 3H), 2.40 (d, J = 7.2 Hz, 1H), 1.26 (s, 6H), 1.24 (s, 6H), 0.99 (s, 6H), 0.85 (d, J = 7.2 Hz, 1H), 0.82 (s, 6H), 0.10 (s, 9H).

^{13}C NMR ($CDCl_3$, 100 MHz) δ 158.0, 134.4, 130.0, 113.2, 83.0, 82.8, 55.5, 29.8, 25.3, 25.1, 24.2, 14.3, -0.6.

^{11}B NMR ($CDCl_3$, 128.3 MHz) δ 32.2.

HRMS (ESI) for **$C_{25}H_{43}B_2O_5Si^+[M+H^+]^+$:** calculated: 473.3065, found: 473.3064.

Melting point: 76.1°C

3-(4-fluorophenyl)-2,2-bis(4,4,5,5-tetramethyl-1,3,2-dioxaborolan-2-yl)cyclopropyl)trimethylsilane (8)

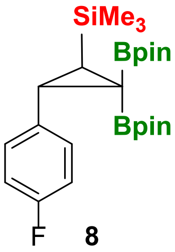

8

Purified by flash column chromatography (pentane:ethyl ether = 10:1) yielded **8** (79%, 73 mg) as a white solid

^{1}H NMR ($CDCl_3$, 400 MHz) δ 7.26 – 7.20 (m, 2H), 6.92 – 6.84 (m, 2H), 2.41 (d, J = 7.2 Hz, 1H), 1.26 (s, 6H), 1.25 (s, 6H), 0.99 (s, 6H), 0.87 – 0.84 (m, 1H), 0.82 (s, 6H), 0.10 (s, 9H).

^{13}C NMR ($CDCl_3$, 100 MHz) δ 161.5 (d, J = 243.2 Hz), 137.9 (d, J = 3.0 Hz), 130.5 (d, J = 7.9 Hz), 114.3 (d, J = 21.1 Hz), 83.1, 82.9, 29.7, 25.3, 25.1, 24.2, 14.5, -0.7.

^{11}B NMR ($CDCl_3$, 128.3 MHz) δ 32.4.

^{19}F NMR ($CDCl_3$, 376.5 MHz) δ -117.9.

HRMS (ESI) for **$C_{24}H_{40}B_2FO_4Si^+$[M+H$^+$]$^+$:** calculated: 461.2866, found: 461.2868.

Melting point: 77.7°C

(3-(2-chlorophenyl)-2,2-bis(4,4,5,5-tetramethyl-1,3,2-dioxaborolan-2-yl)cyclopropyl)trimethylsilane (9)

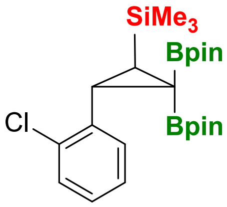

9

Purified by flash column chromatography (pentane:ethyl ether = 10:1) yielded **9** (51%, 49 mg) as a white solid.

^{1}H NMR ($CDCl_3$, 400 MHz) δ 7.30 – 7.27 (m, 1H), 7.18 – 7.13 (m, 1H), 7.12 – 7.04 (m, 2H), 2.53 (d, J = 7.5 Hz, 1H), 1.26 (s, 6H), 1.26 (s, 6H), 0.99 (d, J = 10.6 Hz, 1H), 0.93 (s, 6H), 0.78 (s, 6H), 0.13 (s, 9H).

^{13}C NMR ($CDCl_3$, 100 MHz) δ 140.7, 137.6, 130.1, 129.2, 127.7, 126.4, 83.5, 83.1, 31.2, 25.8, 25.5, 25.3, 24.9, 15.5, -0.0.

^{11}B NMR ($CDCl_3$, 128.3 MHz) δ 32.2.

HRMS (ESI) for **$C_{24}H_{40}B_2ClO_4Si$ [M+H]$^+$:** calculated: 477.2572, found: 477.2575.

Melting point: 76.7°C

Trimethyl(3-(naphthalen-2-yl)-2,2-bis(4,4,5,5-tetramethyl-1,3,2-dioxaborolan-2-yl)cyclopropyl)silane (10)

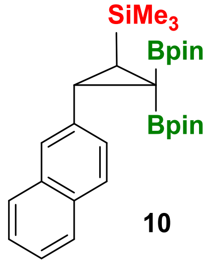

10

Purified by flash column chromatography (pentane:ethyl ether = 10:1) yielded **10** (82%, 81 mg) as a white solid

^{1}H NMR ($CDCl_3$, 400 MHz) δ 7.77 – 7.71 (m, 2H), 7.70 – 7.64 (m, 2H), 7.48 (m, 1H), 7.43 – 7.33 (m, 2H), 2.61 (d, J = 7.2 Hz, 1H), 1.29 (s, 6H), 1.27 (s, 6H), 1.07 (d, J = 7.2 Hz, 1H), 0.89 (s, 6H), 0.69 (s, 6H), 0.15 (s, 9H).

^{13}C NMR ($CDCl_3$, 100 MHz) δ 140.0, 133.4, 132.2, 128.4, 127.6, 127.5, 127.1, 126.0, 125.6, 124.9, 83.1, 82.9, 30.8, 25.3, 25.2, 25.0, 24.1, 14.6, -0.6.

^{11}B NMR ($CDCl_3$, 128.3 MHz) δ 33.3.

HRMS (ESI) for **$C_{28}H_{43}B_2O_4Si^+$[M+H$^+$]$^+$:** calculated: 493.3117, found: 493.3118.

Melting point: 80.1 °C

Cyclopropane 11 has been isolated in both diastereoisomers 11a and 11b.

(E)-(2-(2,2-bis(4,4,5,5-tetramethyl-1,3,2-dioxaborolan-2-yl)vinyl)styryl)trimethylsilane (11a)

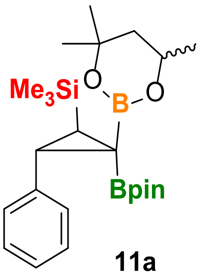

11a

Purified by flash column chromatography (pentane:ethyl ether = 10:1) yielded **11a** (54%, 48 mg) as a pale yellowish solid

^{1}H NMR ($CDCl_3$, 400 MHz) δ 7.28 – 7.24 (m, 2H), 7.21 – 7.15 (m, 2H), 7.10 – 7.05 (m, 1H), 4.26 – 4.13 (m, 1H), 2.44 (d, J = 7.1 Hz, 1H), 1.71 (dd, J = 13.8, 3.0 Hz, 1H), 1.56 – 1.45 (m, 1H), 1.27 (s,

3H), 1.25 (s, 3H), 1.22 (d, J = 7.1 Hz, 3H), 0.98 (s, 6H), 0.86 (s, 6H), 0.83 (d, J = 7.1 Hz, 1H), 0.08 (s, 9H).

^{13}C NMR ($CDCl_3$, 100 MHz) δ 143.1, 128.6, 127.7, 125.5, 82.7, 70.6, 64.8, 45.7, 31.4, 30.0, 28.3, 25.1, 24.2, 23.1, 14.2, -0.3.

^{11}B NMR ($CDCl_3$, 128.3 MHz) δ 32.0, 28.5.

HRMS (ESI) for **$C_{24}H_{41}B_2O_4Si^+[M+H^+]^+$:** calculated: 443.2960, found: 443.2969.

Melting point: 74.5°C

(E)-(2-(2,2-bis(4,4,5,5-tetramethyl-1,3,2-dioxaborolan-2-yl)vinyl)styryl)trimethylsilane (11b)

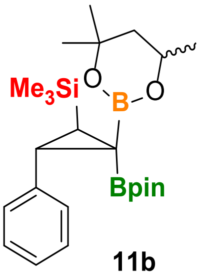

11b

Purified by flash column chromatography (pentane:ethyl ether = 10:1) yielded **11b** (18%, 16 mg) as a pale yellowish solid

^{1}H NMR ($CDCl_3$, 400 MHz) δ 7.29 – 7.21 (m, 2H), 7.22 – 7.13 (m, 2H), 7.12 – 7.05 (m, 1H), 4.14 – 4.01 (m, 1H), 2.48 (d, J = 7.2 Hz, 1H), 1.70 (dd, J = 14.0, 2.9 Hz, 1H), 1.53 (dd, J = 14.0, 11.5 Hz, 1H), 1.25 (s, 6H), 1.21 (d, J = 7.2 Hz, 3H), 0.97 (s, 6H), 0.88 (s, 6H), 0.83 (d, J = 7.2 Hz, 1H), 0.08 (s, 9H).

^{13}C NMR ($CDCl_3$, 100 MHz) δ 143.0, 128.5, 127.7, 125.5, 82.6, 70.7, 64.4, 45.7, 31.4, 30.4, 28.5, 25.0, 24.2, 23.1, 14.5, -0.20.

^{11}B NMR ($CDCl_3$, 128.3 MHz) δ 32.1, 29.2.

HRMS (ESI) for **$C_{24}H_{41}B_2O_4Si^+[M+H^+]^+$:** calculated: 443.2960, found: 443.2963.

Melting point: 74.5°C

((1S,2S,3R)-3-(4-methoxyphenyl)-2-(4,4,5,5-tetramethyl-1,3,2-dioxaborolan-2-yl)-2-(4,4,6-trimethyl-1,3,2-dioxaborinan-2-yl)cyclopropyl)trimethylsilane (12)

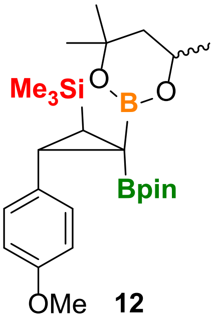

12

Purified by flash column chromatography (pentane:ethyl ether = 10:1) yielded **12** (73%, 69 mg) as a pale yellowish oil.

^{1}H NMR ($CDCl_3$, 400 MHz) δ 7.18 (d, J = 8.7 Hz, 2H), 6.73 (d, J = 6.5 Hz, 2H), 4.24 – 4.13 (m, 1H), 3.74 (s, 3H), 2.38 (d, J = 7.2 Hz, 1H), 1.70 (dt, J = 13.7, 3.1 Hz, 1H), 1.55 – 1.43 (m, 1H), 1.26 (s, 3H), 1.26 – 1.22 (m, 3H), 1.21 (t, J = 6.6 Hz, 3H), 0.99 (s, 6H), 0.86 (s, 6H), 0.75 (d, J = 6.6 Hz, 1H), 0.07 (s, 9H).

^{13}C NMR ($CDCl_3$, 100 MHz) δ 158.1, 129.9, 113.5, 83.0, 70.9, 65.1, 55.8, 46.0, 31.7, 30.7, 30.1, 29.5, 28.6, 25.5, 24.4, 23.4, 14.6, -0.0.

^{11}B NMR ($CDCl_3$, 128.3 MHz) δ 33.4, 30.7.

HRMS (ESI) for **$C_{25}H_{42}B_2O_5Si^+$[M+H$^+$]$^+$:** calculated: 473.3054, found: 473.3056.

Melting point: 76.2°C

(E)-(2-(2,2-bis(4,4,5,5-tetramethyl-1,3,2-dioxaborolan-2-yl)vinyl)styryl)trimethylsilane (13)

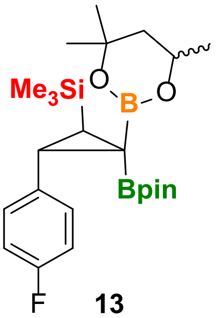

13

Purified by flash column chromatography (pentane:ethyl ether = 10:1) yielded **13** (69%, 63 mg) as a white solid

^{1}H NMR ($CDCl_3$, 400 MHz) δ 7.26 – 7.17 (m, 2H), 6.91 – 6.83 (m, 2H), 4.26 – 4.13 (m, 1H), 2.39 (d, J = 7.1 Hz, 1H), 1.71 (dd, J = 13.9, 3.0 Hz, 1H), 1.49 (m, 1H), 1.27 (s, 3H), 1.24 (s, 3H), 1.22 (d, J = 7.2 Hz, 3H), 0.99 (s, 6H), 0.86 (s, 6H), 0.76 (d, J = 7.1 Hz, 1H), 0.07 (s, 9H).

^{13}**C NMR** ($CDCl_3$, 100 MHz) δ 161.7 (d, J = 242.4 Hz), 139.1 (d, J = 2.5 Hz), 130.4 (d, J = 7.7 Hz), 114.7 (d, J = 21.1 Hz), 83.1, 71.0, 65.2, 46.1, 31.8, 29.6, 28.7, 25.5, 24.5, 23.4, 14.9, -0.0.

^{11}**B NMR** ($CDCl_3$, 128.3 MHz) δ 32.2, 28.9.

HRMS (ESI) for **$C_{24}H_{40}B_2FO_4Si^+[M+H^+]^+$:** calculated: 461.2866, found: 461.2867.

Melting point: 78.2°C

Trimethyl(3-(naphthalen-2-yl)-2-(4,4,5,5-tetramethyl-1,3,2-dioxaborolan-2-yl)-2-((S)-4,4,6-trimethyl-1,3,2-dioxaborinan-2-yl)cyclopropyl)silane (14)

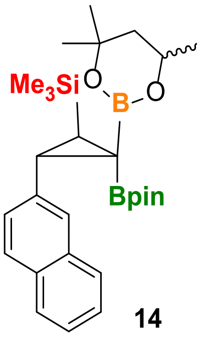

14

Purified by flash column chromatography (pentane:ethyl ether = 10:1) yielded **14** (73%, 72 mg) as a white solid

^{1}**H NMR** ($CDCl_3$, 400 MHz) δ 7.76 – 7.69 (m, 2H), 7.70 – 7.62 (m, 2H), 7.50 – 7.43 (m, 1H), 7.43 – 7.30 (m, 2H), 4.29 – 4.16 (m, 1H), 2.58 (d, J = 6.9 Hz, 1H), 1.73 (dd, J = 13.9, 3.0 Hz, 1H), 1.56 – 1.48 (m, 1H), 1.29 (s, 3H), 1.27 (s, 3H), 1.25 (d, J = 6.9 Hz, 3H), 0.98 (d, J = 7.4 Hz, 2H), 0.89 (s, 6H), 0.75 (s, 6H), 0.12 (s, 9H).

^{13}**C NMR** ($CDCl_3$, 100 MHz) δ 140.9, 133.5, 132.1, 128.2, 127.6, 127.5, 127.1, 125.6, 125.5, 124.7, 82.7, 70.7, 64.8, 45.7, 31.4, 30.3, 28.3, 25.1, 24.1, 23.1, 14.7, -0.2.

^{11}**B NMR** ($CDCl_3$, 128.3 MHz) δ 31.5, 28.6.

HRMS (ESI) for **$C_{28}H_{43}B_2O_4Si^+[M+H^+]^+$:** calculated: 493.3117, found: 493.3121.

Melting point: 80.4°C

(3-(4-methoxyphenyl)-2-(4,4,5,5-tetramethyl-1,3,2-dioxaborolan-2-yl)-2-((3aS,7aR)-3a,5,5-trimethylhexahydro-4,6-methanobenzo[d][1,3,2]dioxaborol-2-yl)cyclopropyl)trimethylsilane (15)

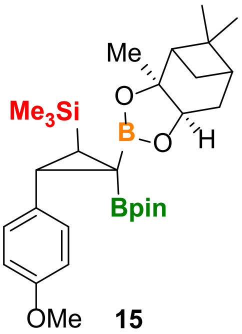

15

Purified by flash column chromatography (pentane:ethyl ether = 10:1) yielded **15** (86%, 69 mg) as a pale yellowish solid

^{1}H NMR ($CDCl_3$, 400 MHz) δ 7.20 (dd, J = 8.7, 0.7 Hz, 2H), 6.74 (d, J = 8.7 Hz, 2H), 4.26 (dd, J = 8.7, 2.0 Hz, 1H), 3.75 (s, 3H), 2.42 (d, J = 7.2 Hz, 1H), 2.36 – 2.25 (m, 1H), 2.26 – 2.16 (m, 1H), 2.11 – 2.04 (m, 1H), 1.93 – 1.85 (m, 2H), 1.51 (d, J = 10.9 Hz, 1H), 1.37 (s, 3H), 1.30 (s, 3H), 0.98 (s, 6H), 0.86 (d, J = 7.2 Hz, 1H), 0.84 (s, 3H), 0.82 (s, 6H), 0.09 (s, 9H).

^{13}C NMR ($CDCl_3$, 100 MHz) δ 158.0, 134.4, 130.1, 113.1, 85.5, 82.8, 77.9, 55.5, 51.6, 39.6, 38.4, 35.5, 29.7, 28.5, 27.3, 26.2, 25.2, 24.2, 24.2, 24.2, 14.2, -0.8.

^{11}B NMR ($CDCl_3$, 128.3 MHz) δ 31.6.

HRMS (ESI) for **$C_{29}H_{47}B_2O_5Si^+$[M+H$^+$]$^+$:** calculated: 525.3378, found: 525.3380.

Melting point: 80.3°C

Trimethyl(3-(naphthalen-2-yl)-2-(4,4,5,5-tetramethyl-1,3,2-dioxaborolan-2-yl)-2-((3aS,7aR)-3a,5,5-trimethylhexahydro-4,6-methanobenzo[d][1,3,2]dioxaborol-2-yl)cyclopropyl)silane (16)

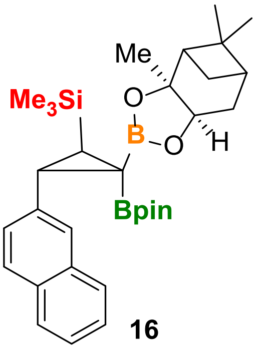

16

Purified by flash column chromatography (pentane:ethyl ether = 10:1) yielded **16** (94%, 88 mg) as a pale yellowish solid.

^{1}H NMR ($CDCl_3$, 400 MHz) δ 7.74 (m, 2H), 7.67 (d, J = 8.7 Hz, 2H), 7.53 – 7.44 (m, 1H), 7.42 – 7.33 (m, 2H), 4.29 (dd, J = 8.7, 1.9 Hz, 1H), 2.62 (d, J = 7.3 Hz, 1H), 2.38 – 2.21 (m, 2H), 2.11 (dd, J = 6.1, 4.8 Hz, 1H), 1.96 – 1.88 (m, 2H), 1.54 (d, J = 10.9 Hz, 1H), 1.40 (s, 3H), 1.31 (s, 3H), 1.08 (d, J = 7.3 Hz, 1H), 0.89 (m, 7H), 0.86 (s, 3H), 0.68 (s, 6H), 0.14 (s, 9H).

^{13}C NMR ($CDCl_3$, 100 MHz) δ 139.9, 133.4, 132.2, 128.5, 127.6, 127.5, 127.0, 126.2, 125.6, 124.9, 85.6, 82.9, 77.9, 77.4, 77.3, 77.1, 76.8, 51.6, 39.6, 38.4, 35.5, 30.7, 28.5, 27.3, 26.2, 25.1, 24.2, 14.5, -0.8.

^{11}B NMR ($CDCl_3$, 128.3 MHz) δ 31.5.

HRMS (ESI) for **$C_{32}H_{47}B_2O_4Si^+[M+H^+]^+$:** calculated: 545.3429, found: 545.3432.

Melting point: 82.6°C

(2-((4*R*,5*S*)-4,5-diphenyl-1,3,2-dioxaborolan-2-yl)-3-(4-methoxyphenyl)-2-(4,4,5,5-tetramethyl-1,3,2-dioxaborolan-2-yl)cyclopropyl)trimethylsilane (17)

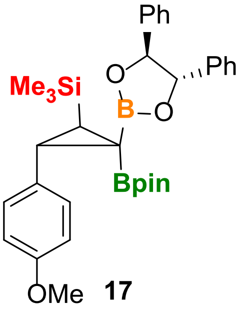

17

Purified by flash column chromatography (pentane:ethyl ether = 10:1) yielded **17** (86%, 98 mg) as a pale yellowish oil.

^{1}H NMR ($CDCl_3$, 400 MHz) δ 7.44 – 7.22 (m, 12H), 6.81 – 6.75 (m, 2H), 5.18 (s, 2H), 3.77 (s, 3H), 2.63 (d, J = 7.8 Hz, 1H), 1.04 (s, 6H), 1.03 (d, J = 7.8 Hz, 1H), 0.90 (s, 6H), 0.13 (s, 9H).

^{13}C NMR ($CDCl_3$, 100 MHz) δ 140.2, 130.0, 128.5, 128.1, 126.2, 113.1, 86.5, 83.0, 55.4, 24.9, 24.3, -1.0.

^{11}B NMR ($CDCl_3$, 128.3 MHz) δ 28.6.

HRMS (ESI) for **$C_{33}H_{43}B_2O_5Si^+[M+H^+]^+$:** calculated: 569.3065, found: 569.3065.

(2-((4S,5R)-4,5-diphenyl-1,3,2-dioxaborolan-2-yl)-3-(naphthalen-2-yl)-2-(4,4,5,5-tetramethyl-1,3,2-dioxaborolan-2-yl)cyclopropyl)trimethylsilane (18)

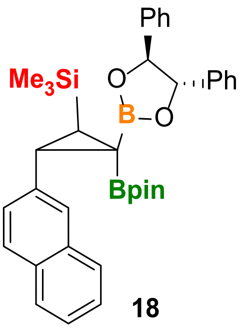

18

Purified by flash column chromatography (pentane:ethyl ether = 10:1) yielded **18** (81%, 95 mg) as a pale yellowish oil.

^{1}H NMR ($CDCl_3$, 400 MHz) δ 7.81 – 7.68 (m, 4H), 7.56 (dd, J = 8.4, 1.8 Hz, 1H), 7.41 – 7.29 (m, 11H), 6.90 (dt, J = 8.2, 7.0 Hz, 1H), 6.63 (dt, J = 8.4, 1.5 Hz, 1H), 5.23 (s, 2H), 2.90 – 2.82 (m, 1H), 0.94 (s, 6H), 0.92 – 0.81 (m, 1H), 0.76 (s, 6H), 0.20 (s, 9H).

^{13}C NMR (CDCl3, 100 MHz) δ 141.2, 129.6, 129.5, 129.3, 129.2, 129.2, 129.1, 128.5, 128.4, 128.0, 127.3, 127.2, 126.7, 126.6, 87.6, 84.0, 31.9, 25.8, 25.2, 15.6, -0.0.

^{11}B NMR ($CDCl_3$, 128.3 MHz) δ 32.5.

HRMS (ESI) for **$C_{36}H_{43}B_2O_4Si^+$[M+H$^+$]$^+$:** calculated: 588.3149, found: 588.3148.

Characterization data for B, Si cyclopropanes

(2-(4-methoxyphenyl)-3-(4,4,5,5-tetramethyl-1,3,2-dioxaborolan-2-yl)cyclopropyl)trimethylsilane (19a)

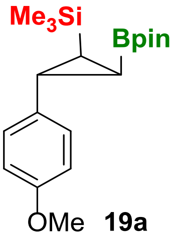

19a

Purified by flash column chromatography (pentane:ethyl ether = 10:1) yielded **19a** (80%, 55 mg) as a pale yellowish solid

^{1}H NMR ($CDCl_3$, 400 MHz) δ 7.03 (d, J = 8.7 Hz, 2H), 6.79 (d, J = 8.7 Hz, 2H), 3.77 (s, 3H), 2.07 (dd, J = 7.1, 5.9 Hz, 1H), 1.24 (s, 12H), 0.42 (dd, J = 11.5, 5.9 Hz, 1H), 0.27 (dd, J = 11.5, 7.1 Hz, 1H), 0.09 (s, 9H).

^{13}C NMR ($CDCl_3$, 100 MHz) δ 157.7, 136.8, 126.8, 113.8, 83.2, 77.4, 77.1, 76.8, 55.4, 26.5, 25.3, 24.8, 17.1, -0.5.

^{11}**B NMR** ($CDCl_3$, 128.3 MHz) δ 33.2.

HRMS (ESI) for **$C_{19}H_{32}BO_3Si^+[M+H^+]^+$:** calculated: 347.2213, found: 347.2214.

Melting point: 60.2°C

(2-(4-methoxyphenyl)-3-(4,4,5,5-tetramethyl-1,3,2-dioxaborolan-2-yl)cyclopropyl)trimethylsilane (20a)

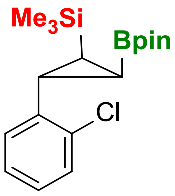

20a

Purified by flash column chromatography (pentane:ethyl ether = 10:1) yielded **20a** (76%, 53 mg) as a pale yellowish solid

^{1}**H NMR** ($CDCl_3$, 400 MHz) δ 7.32 (dd, J = 7.7, 1.5 Hz, 1H), 7.12 (m, 2H), 7.05 – 6.99 (m, 1H), 2.36 (dd, J = 7.3, 6.1 Hz, 1H), 1.26 (s, 12H), 0.53 (dd, J = 11.4, 6.1Hz 1H), 0.27 (dd, J = 11.4, 7.4 Hz, 1H), 0.14 (s, 9H).

^{13}**C NMR** ($CDCl_3$, 100 MHz) δ 142.3, 136.2, 129.6, 127.4, 127.3, 127.1, 83.9, 26.4, 25.9, 25.3, 16.1, -0.0.

^{11}**B NMR** ($CDCl_3$, 128.3 MHz) δ 33.4.

HRMS (ESI) for **$C_{18}H_{29}BClO_2Si$ [M+H]$^+$**calculated: 351.1716, found: 351.1717.

Melting point: 61.0°C

Trimethyl((1S,2S,3S)-2-(naphthalen-2-yl)-3-(4,4,5,5-tetramethyl-1,3,2-dioxaborolan-2-yl)cyclopropyl)silane (21a)

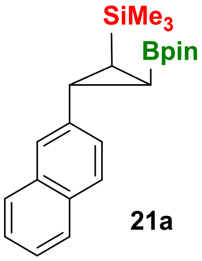

21a

Purified by flash column chromatography (pentane:ethyl ether = 10:1) yielded **21a** (80%, 58 mg) as a white solid

^{1}H NMR ($CDCl_3$, 400 MHz) δ 7.79 – 7.72 (m, 3H), 7.63 – 7.57 (m, 1H), 7.46 – 7.36 (m, 2H), 7.19 (dd, J = 8.6, 1.8 Hz, 1H), 2.30 (dd, J = 7.0, 5.9 Hz, 1H), 1.28 (s, 6H), 1.27 (s, 6H), 0.66 (dd, J = 11.6, 5.9 Hz, 1H), 0.47 (dd, J = 11.6, 7.0 Hz, 1H), 0.14 (s, 9H).

^{13}C NMR ($CDCl_3$, 100 MHz) δ 142.31, 133.65, 132.08, 127.96, 127.68, 127.32, 126.08, 124.97, 124.24, 124.13, 83.35, 27.48, 25.39, 24.90, 17.92, -0.53.

^{11}B NMR ($CDCl_3$, 128.3 MHz) δ 32.95.

HRMS (ESI) for **$C_{22}H_{32}BO_2Si^+$[M+H$^+$]$^+$:** calculated: 367.2264, found: 367.2267

Melting point: 63.5°C

Trimethyl(2-(naphthalen-2-yl)-3-((3aS,7aR)-3a,5,5-trimethylhexahydro-4,6-methanobenzo[d][1,3,2]dioxaborol-2-yl)cyclopropyl)silane (25)

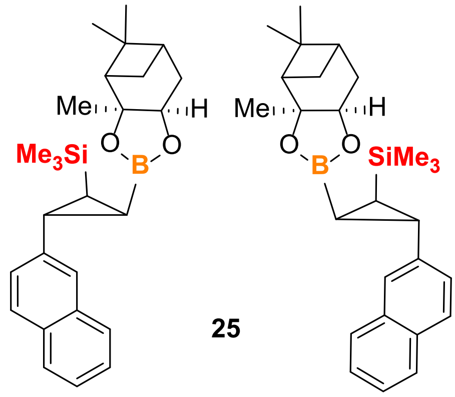

25

Purified by flash column chromatography (pentane:ethyl ether = 10:1) yielded **25** (94%, 79 mg) as a white solid.

^{1}H NMR ($CDCl_3$, 400 MHz) δ 7.82 – 7.69 (m, 3H), 7.59 (q, J = 0.9 Hz, 1H), 7.47 – 7.33 (m, 3H), 7.19 (dd, J = 8., 1.9 Hz, 1H), 4.30 (dd, J = 8.8, 1.9 Hz, 1H), 2.37 – 2.20 (m, 3H), 2.06 (dd, J = 8.3, 3.6 Hz, 1H), 1.94 – 1.77 (m, 1H), 1.39 (s, 3H), 1.30 (s, 3H), 1.27 – 1.19 (m, 2H), 0.85 (s, 4H), 0.72 – 0.62 (m, 1H), 0.54 – 0.44 (m, 1H), 0.13 (s, 9H).

^{13}C NMR ($CDCl_3$, 100 MHz) δ 142.8, 134.1, 132.5, 128.4, 128.1, 127.8, 126.6, 125.4, 124.7, 124.6, 86.3, 78.6, 52.0, 40.1, 38.8, 35.8, 29.2, 27.8, 27.7, 26.9, 24.6, 18.2, -0.0.

^{11}B NMR ($CDCl_3$, 128.3 MHz) δ 33.2.

HRMS (ESI) for **$C_{26}H_{35}BO_2Si^+$[M+H$^+$]$^+$:** calculated: 419.4413, found: 419.4412.

Melting point: 77.9°C

(2-(4,5-diphenyl-1,3,2-dioxaborolan-2-yl)-3-(naphthalen-2 yl)cyclopropyl) trimethylsilane (26)

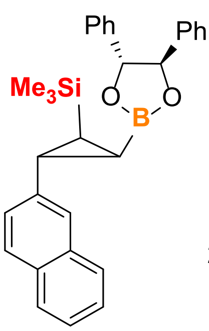

26

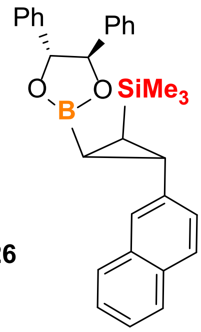

Purified by flash column chromatography (pentane:ethyl ether = 10:1) yielded **26** (97%, 73 mg) as a pale yellowish oil.

^{1}H NMR ($CDCl_3$, 400 MHz) δ 7.70 – 7.53 (m, 6H), 7.46 (d, J = 2.1 Hz, 2H), 7.39 – 7.20 (m, 6H), 7.08 – 6.97 (m, 2H), 6.86 (d, J = 0.8 Hz, 1H), 5.51 (s, 2H), 2.84 – 2.74 (m, 1H), 0.58 – 0.47 (m, 1H), 0.38 – 0.25 (m, 1H), -0.00 (s, 9H).

^{13}C NMR ($CDCl_3$, 100 MHz) δ 150.5, 137.6, 128.4, 128.2, 127.8, 126.9, 126.6, 125.5, 124.8, 124.6, 83.8, 30.9, 25.9, 25.4, -0.0.

^{11}B NMR ($CDCl_3$, 128.3 MHz) δ 33.1.

HRMS (ESI) for **$C_{30}H_{31}BO_2Si^+$[M+H$^+$]$^+$:** calculated: 463.4512, found: 463.4510.

(2-(4-methoxyphenyl)-3-((3aS,7aR)-3a,5,5-trimethylhexahydro-4,6-methanobenzo[d][1,3,2]dioxaborol-2-yl)cyclopropyl)trimethylsilane (27)

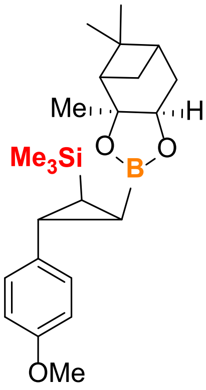

27

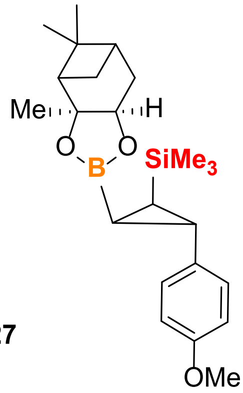

Purified by flash column chromatography (pentane:ethyl ether = 10:1) yielded **27** (87%, 69 mg) as a pale yellowish oil.

^{1}H NMR ($CDCl_3$, 400 MHz) δ 7.03 (d, J = 8.6 Hz, 2H), 6.83 – 6.77 (m, 2H), 4.27 (dd, J = 8.6, 1.9 Hz, 1H), 3.77 (d, J = 0.7 Hz, 3H), 2.36 – 2.25 (m, 1H), 2.25 – 2.17 (m, 1H), 2.05 (dt, J = 14.4, 6.0 Hz, 2H), 1.93 – 1.82 (m, 2H), 1.36 (d, J = 0.8 Hz, 3H), 1.28 (s, 3H), 1.21 – 1.17 (m, 2H), 0.83 (s, 3H), 0.51 – 0.41 (m, 1H), 0.34 – 0.24 (m, 1H), 0.09 (s, 9H).

^{13}**C NMR** (CDCl3, 100 MHz) δ 157.5, 136.6, 129.8, 126.6, 113.6, 85.5, 77.8, 55.3, 51.3, 39.4, 38.1, 35.2, 30.3, 28.5, 27.0, 26.3, 26.2, 23.9, 16.8, -0.6.

^{11}**B NMR** ($CDCl_3$, 128.3 MHz) δ 31.1.

HRMS (ESI) for $\mathbf{C_{23}H_{35}BO_3Si^+[M+H^+]^+}$: calculated: 399.4140, found: 399.4142.

(2-(4,5-diphenyl-1,3,2-dioxaborolan-2-yl)-3-(4- methoxyphenyl)cyclopropyl)trimethylsilane (28)

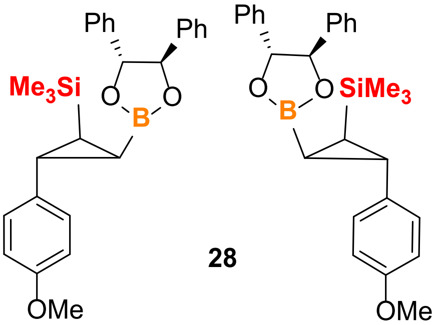

28

Purified by flash column chromatography (pentane:ethyl ether = 10:1) yielded **28** (90%, 80 mg) as a pale yellowish oil.

^{1}**H NMR** ($CDCl_3$, 400 MHz) δ 7.42 – 7.32 (m, 5H), 7.29 (dt, J = 8.0, 2.0 Hz, 5H), 7.25 – 7.21 (m, 2H), 6.84 – 6.79 (m, 2H), 5.16 (s, 2H), 3.80 (s, 3H), 2.81 – 2.72 (m, 1H), 0.73 – 0.65 (m, 1H), 0.50 (dt, J = 9.9, 7.4 Hz, 1H), -0.20 (d, J = 1.8 Hz, 9H).

^{13}**C NMR** ($CDCl_3$, 100 MHz) δ 141.6, 131.5, 129.9, 129.4, 126.8, 114.3, 87.5, 56.3, 28.3, 15.3, -0.0.

^{11}**B NMR** ($CDCl_3$, 128.3 MHz) δ 36.5.

HRMS (ESI) for $\mathbf{C_{27}H_{31}BO_3SiNa^+[M+Na]^+}$: calculated: 465.2033, found: 465.2039.

Characterization data for cyclopropanol derivatives

2-(4-methoxyphenyl)-3-(trimethylsilyl)cyclopropan-1-ol (22)

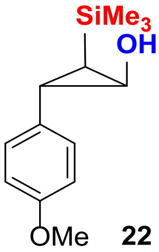

22

Purified by flash column chromatography (pentane:ethyl ether = 10:2) yielded **22** (92%, 21 mg) as a pale yellowish oil

^{1}H NMR ($CDCl_3$, 400 MHz) δ 6.96 (d, J = 8.7 Hz, 2H), 6.80 (d, J = 8.7 Hz, 2H), 3.80 (dd, J = 7.4, 2.4 Hz, 1H), 3.78 (s, 3H), 2.00 (dd, J = 8.2, 2.4 Hz, 1H), 0.24 (dd, J = 8.2, 7.4 Hz, 1H), 0.13 (s, 9H).

^{13}C NMR ($CDCl_3$, 100 MHz) δ 157.8, 134.3, 126.9, 113.9, 59.7, 55.4, 29.8, 28.5, 18.8, -0.2.

2-(4-methoxyphenyl)-3-(trimethylsilyl)cyclopropan-1-ol (23)

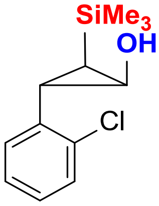

23

Purified by flash column chromatography (pentane:ethyl ether = 10:2) yielded **23** (95%, 43 mg) as a pale yellowish oil

^{1}H NMR ($CDCl_3$, 400 MHz) δ 7.35 (dd, J = 7.5, 1.8 Hz, 1H), 7.18 – 7.08 (m, 2H), 6.92 – 6.83 (m, 1H), 3.85 (dd, J = 7.6, 2.6 Hz, 1H), 2.31 (dd, J = 8.5, 2.6, 1H), 1.58 (bs, 1H), 0.30 (dd, J = 8.5, 7.6 Hz, 1H), 0.17 (s, 9H).

^{13}C NMR ($CDCl_3$, 100 MHz) δ 139.7, 135.4, 129.5, 127.4, 127.1, 127.1, 59.1, 27.7, 18.3, -0.0

(1S,2S,3R)-2-(naphthalen-2-yl)-3-(trimethylsilyl)cyclopropan-1-ol (24)

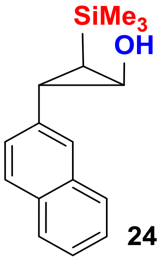

24

Purified by flash column chromatography (pentane:ethyl ether = 10:1) yielded **24** (96%, 49 mg) as a pale yellowish oil.

^{1}H NMR ($CDCl_3$, 400 MHz) δ 7.57 (ddd, J = 17.8, 8.2, 1.9 Hz, 3H), 7.28 – 7.18 (m, 3H), 6.98 (dd, J = 8.5, 1.9 Hz, 1H), 3.80 (dd, J = 7.5, 2.4 Hz, 1H), 2.02 (dd, J = 8.5, 2.4 Hz, 1H), 0.86 (m, 1H), -0.01 (s, 9H).

^{13}C NMR ($CDCl_3$, 100 MHz) δ 140.1, 133.8, 132.2, 128.2, 127.9, 127.6, 126.4, 125.3, 125.1, 123.8, 60.4, 29.8, 19.8, -0.0.

Characterization data for vinyl silane

(E)-(2-(2,2-bis(4,4,5,5-tetramethyl-1,3,2-dioxaborolan-2-yl)vinyl)styryl)trimethylsilane (A)

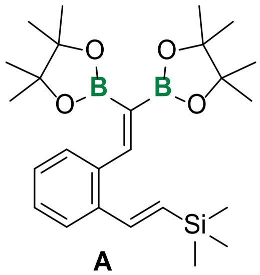

A

Purified by flash column chromatography (pentane:ethyl ether = 10:1) yielded **A** (41%, 37 mg) as a brown-yellowish solid

^{1}H NMR ($CDCl_3$, 400 MHz) δ 8.00 (s, 1H), 7.44 (td, J = 7.6, 1.5 Hz, 2H), 7.24 (td, J = 7.5, 1.3 Hz, 1H), 7.17 (td, J = 7.5, 1.4 Hz, 1H), 7.12 (d, J = 19.1 Hz, 1H), 6.32 (d, J = 19.1 Hz, 1H), 1.28 (s, 12H), 1.24 (s, 12H), 0.14 (s, 9H).

^{13}C NMR ($CDCl_3$, 100 MHz) δ 154.8, 141.5, 138.3, 137.6, 133.5, 128.4, 128.4, 127.3, 126.0, 83.7, 83.3, 25.0, 24.7, -1.1

^{11}B NMR ($CDCl_3$, 128.3 MHz) δ 29.9.

Melting point: 76.9°C

^{1}H, ^{13}C, ^{11}B, and NOE's spectra

^{1}H NMR ($CDCl_3$, 400 MHz)

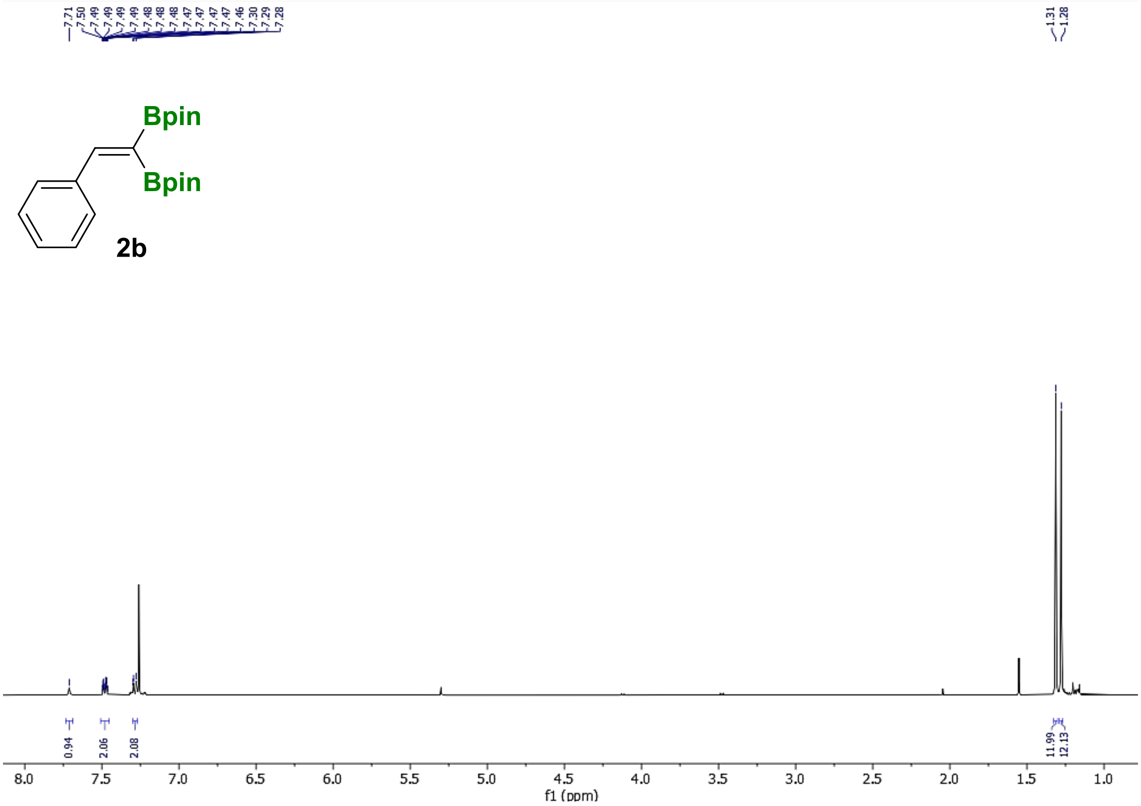

^{13}C NMR ($CDCl_3$, 100 MHz)

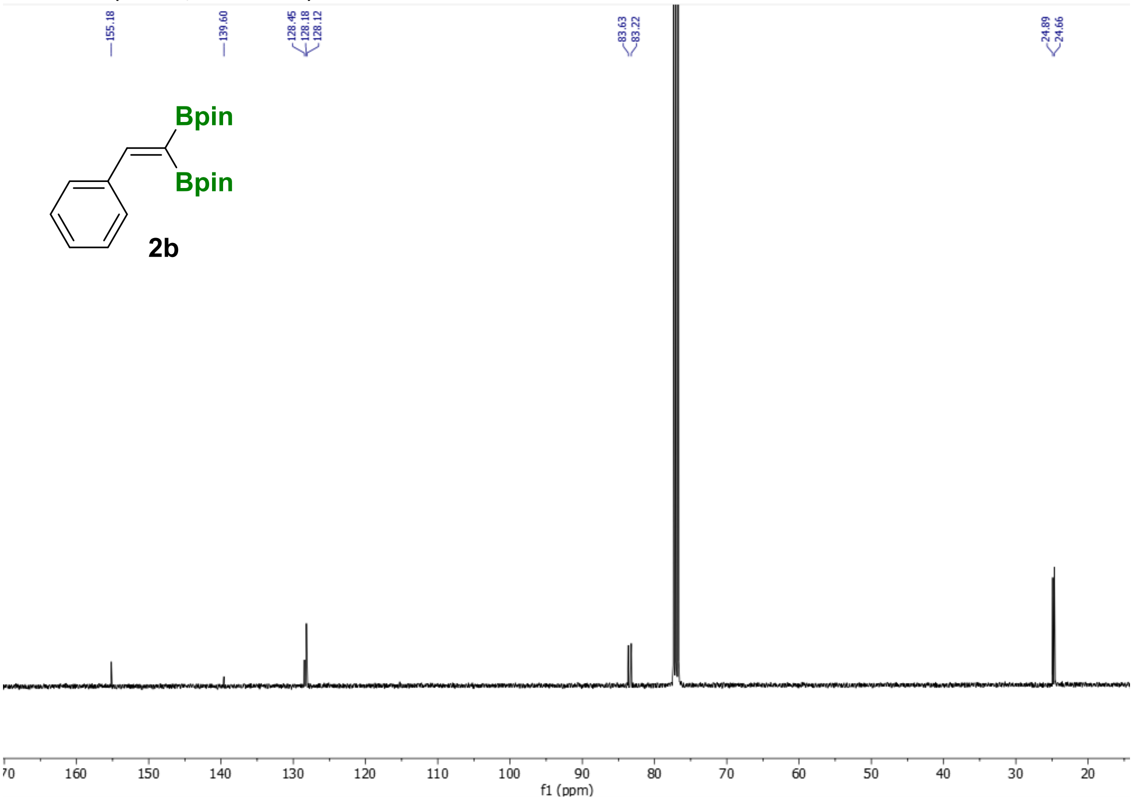

^{11}B NMR ($CDCl_3$, 128.3 MHz)

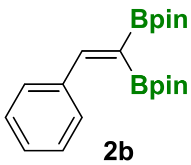

2b

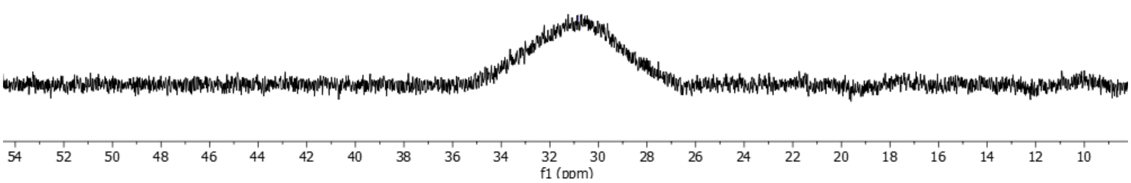

^{1}H NMR ($CDCl_3$, 400 MHz)

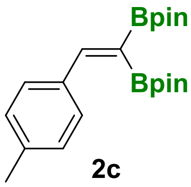

2c

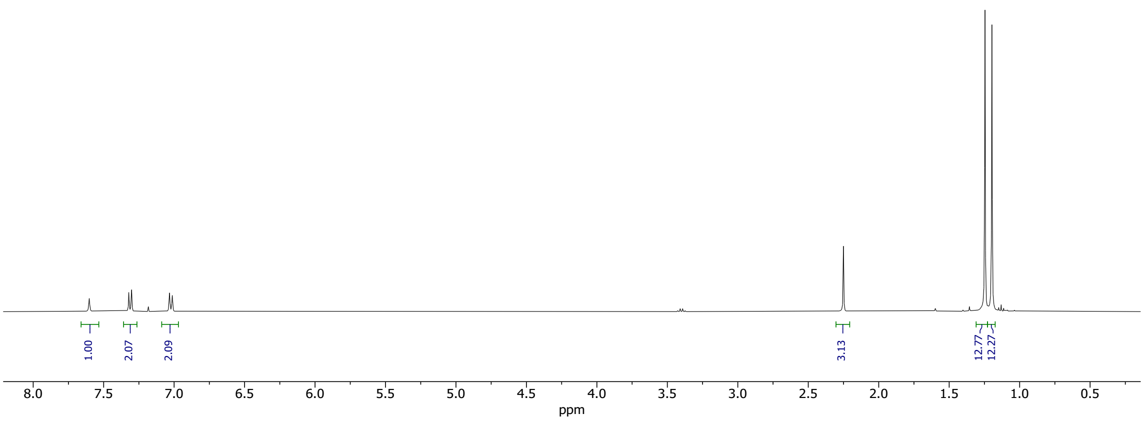

^{13}**C NMR** ($CDCl_3$, 100 MHz)

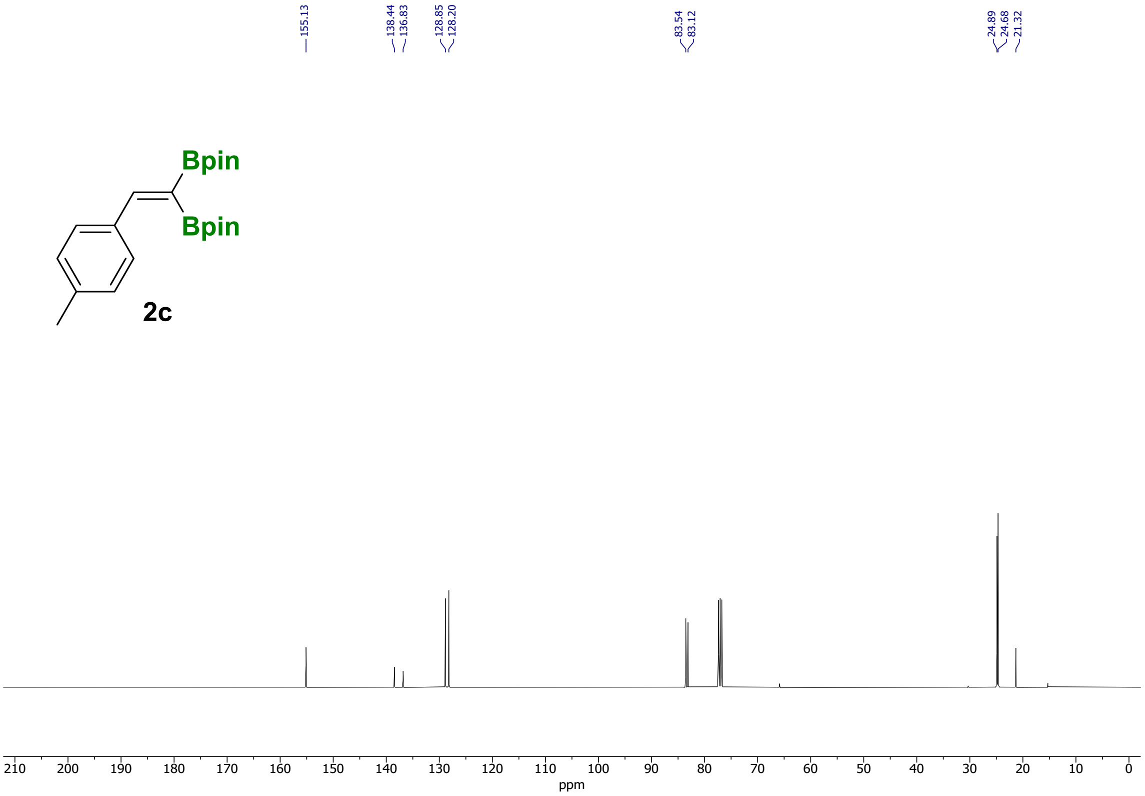

^{11}**B NMR** ($CDCl_3$, 128.3 MHz)

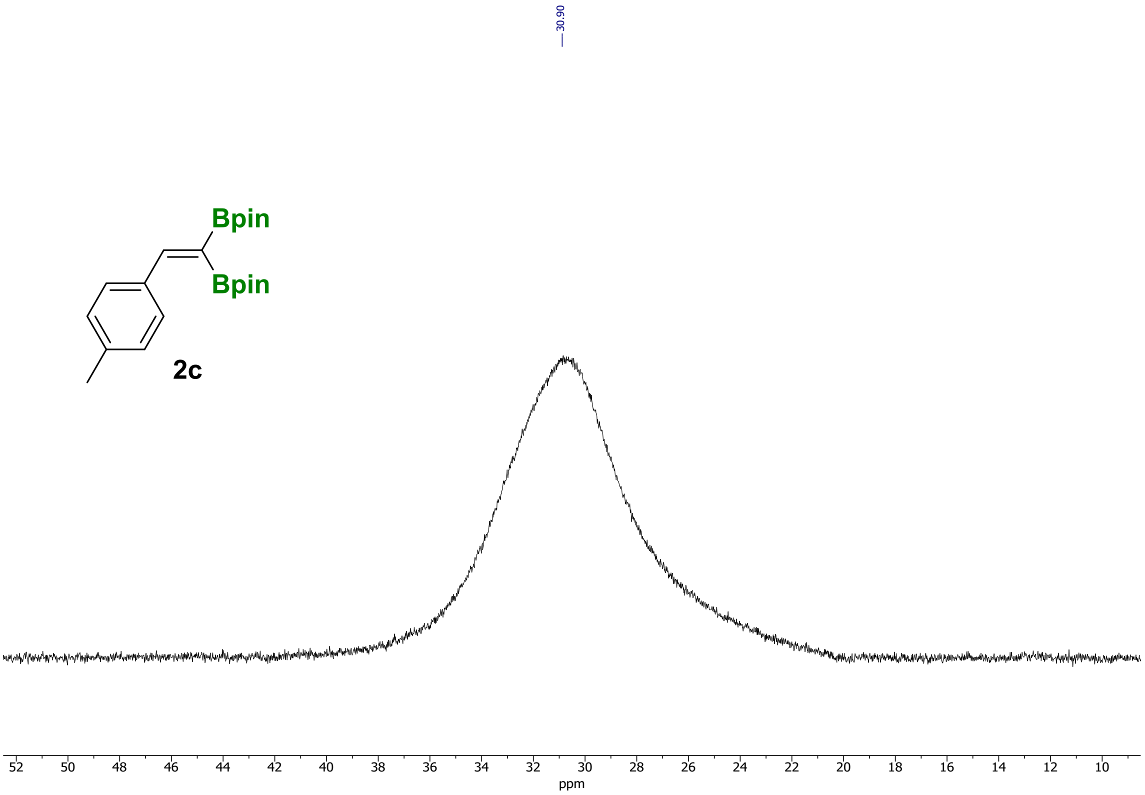

^{1}H NMR ($CDCl_3$, 400 MHz)

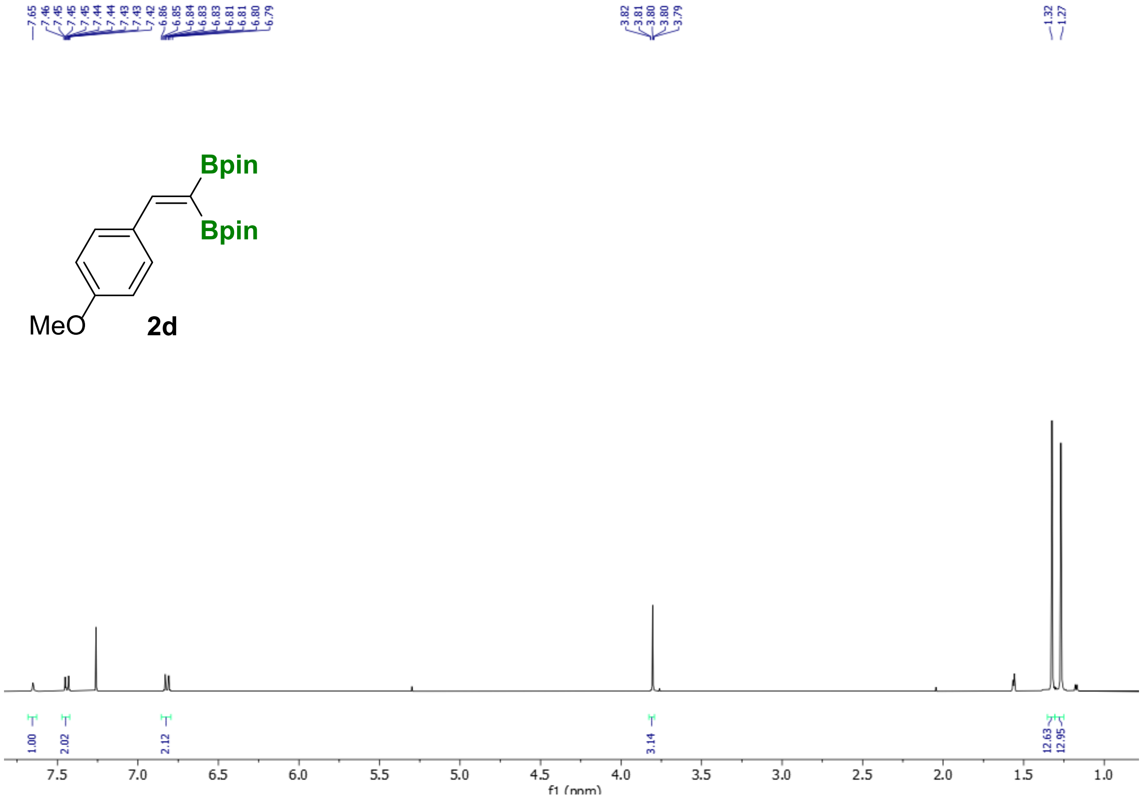

^{13}C NMR ($CDCl_3$, 100 MHz)

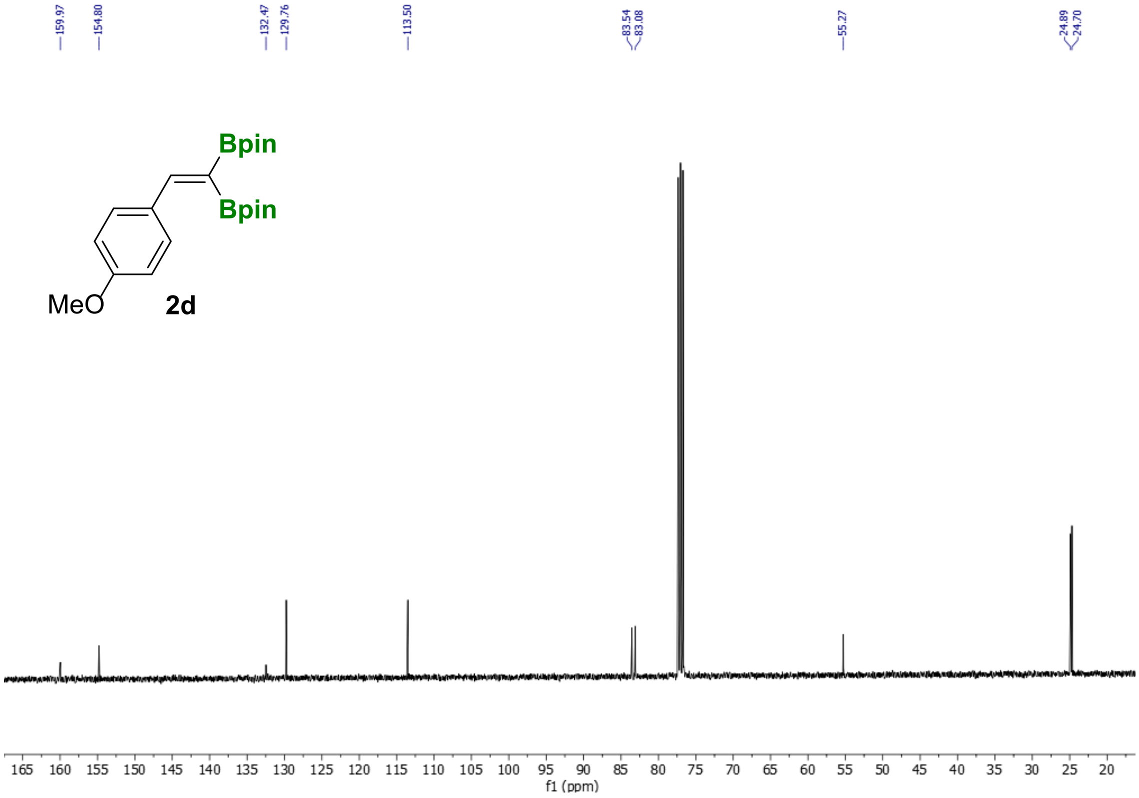

^{11}B NMR (CDCl$_3$, 128.3 MHz)

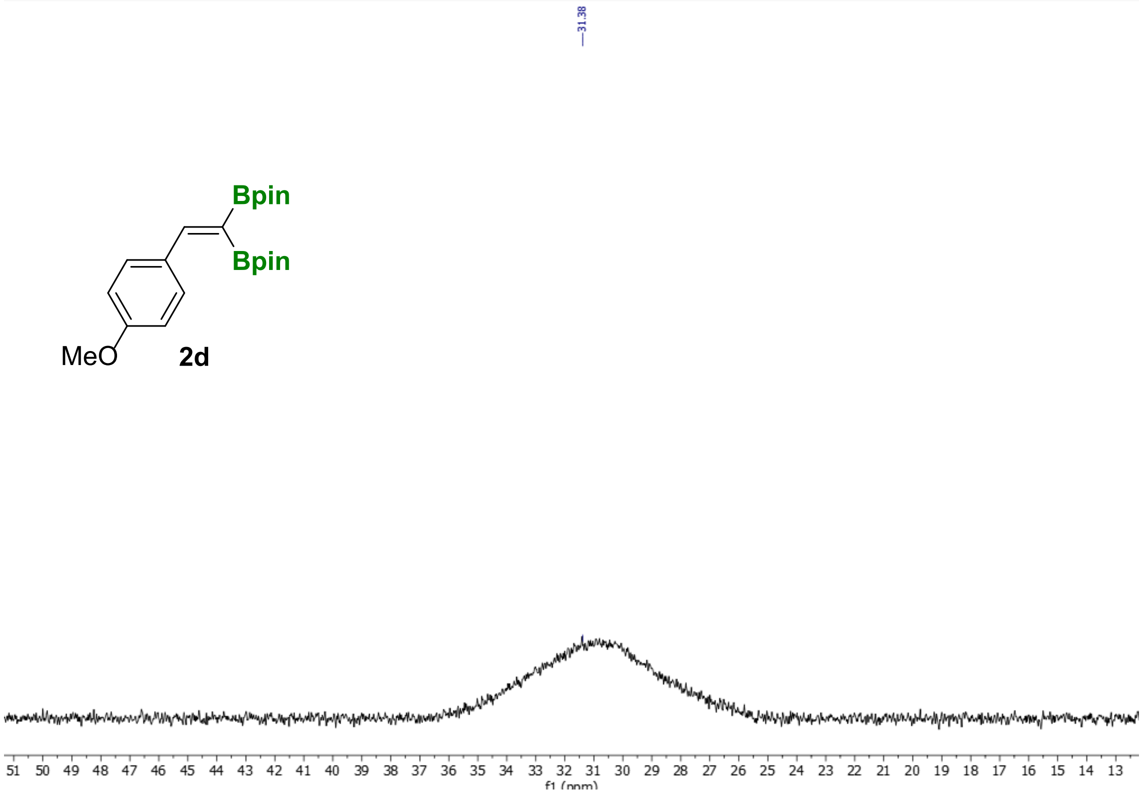

^{1}H NMR (CDCl$_3$, 400 MHz)

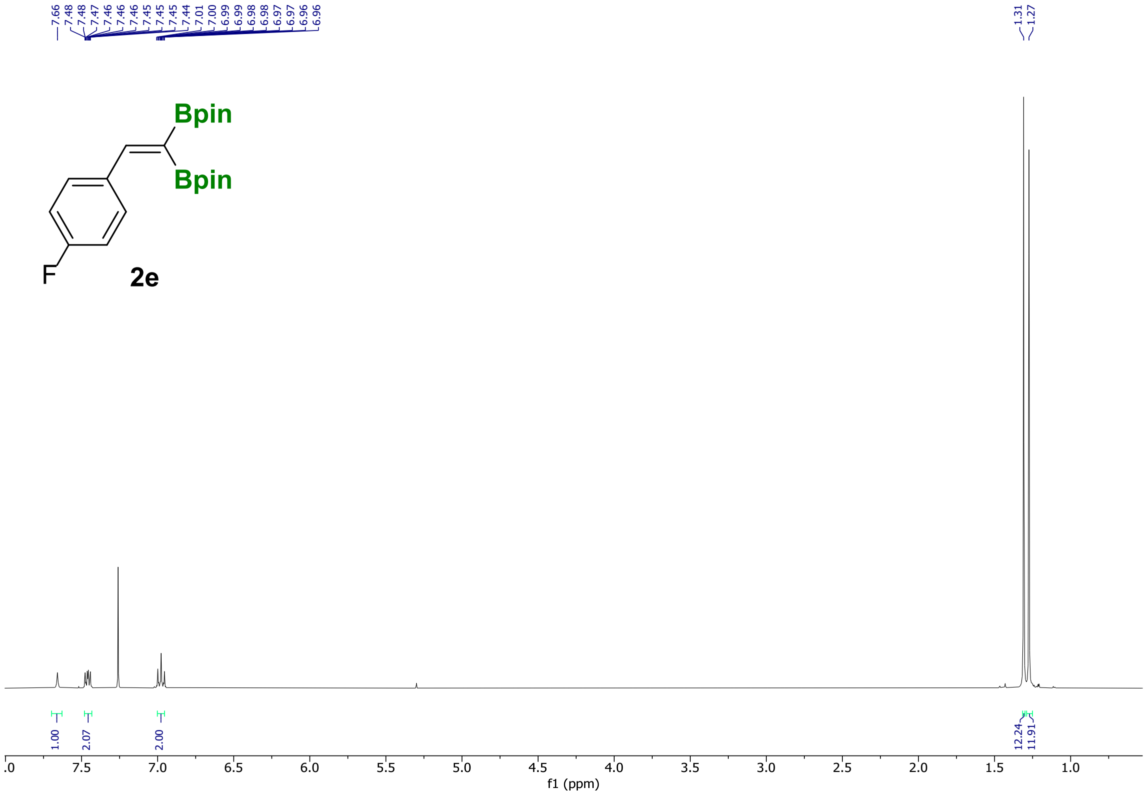

^{13}C NMR ($CDCl_3$, 100 MHz)

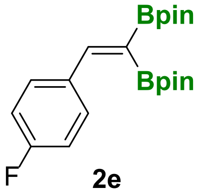

2e

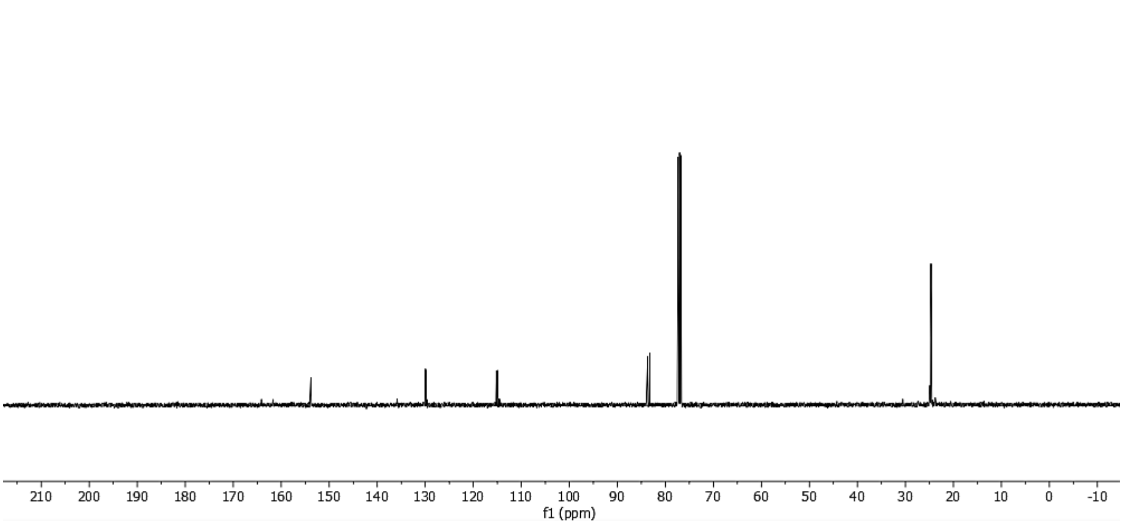

^{11}B NMR ($CDCl_3$, 128.3 MHz)

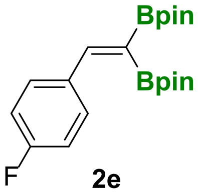

2e

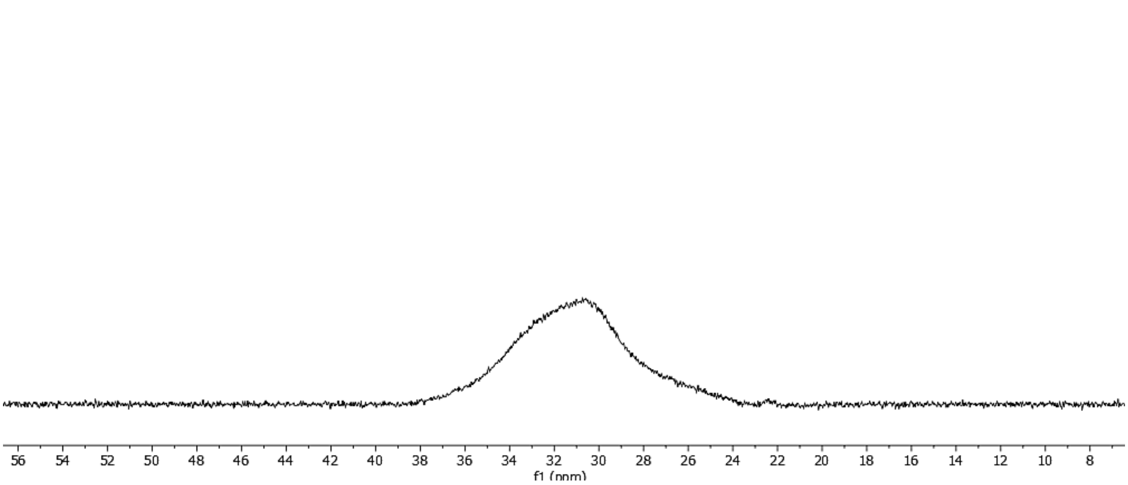

^{1}H NMR ($CDCl_3$, 400 MHz)

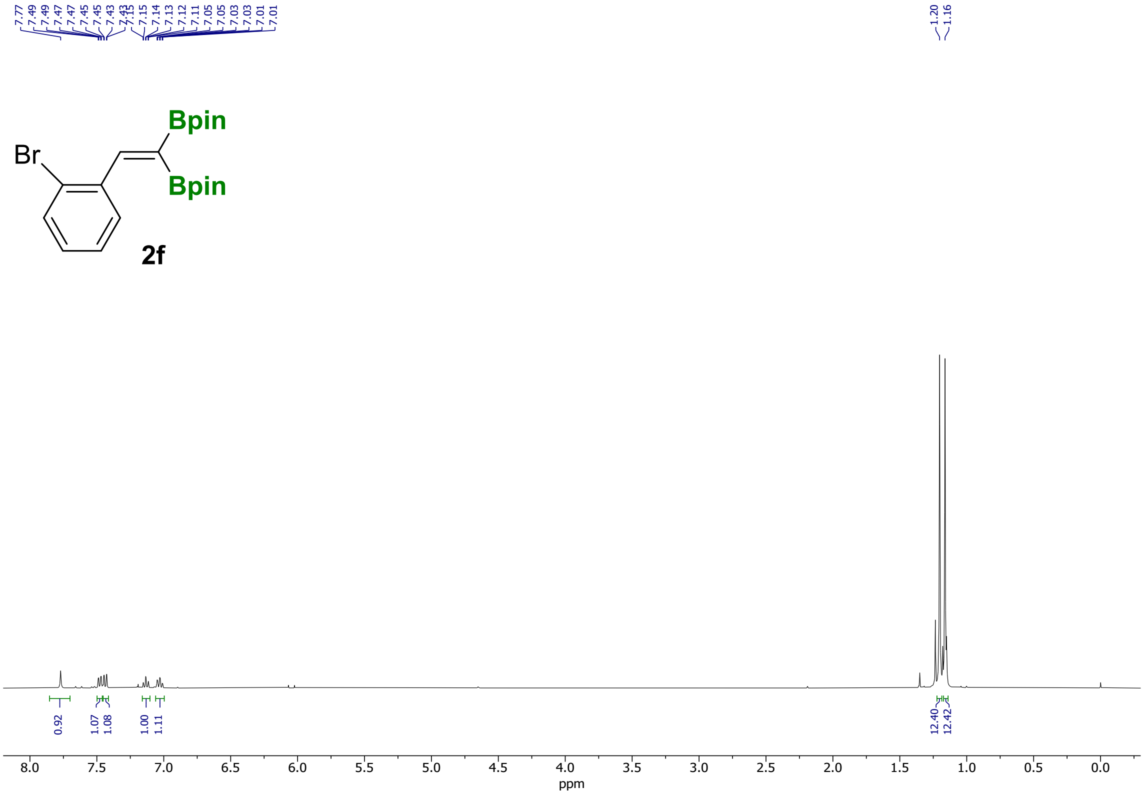

^{13}C NMR ($CDCl_3$, 100 MHz)

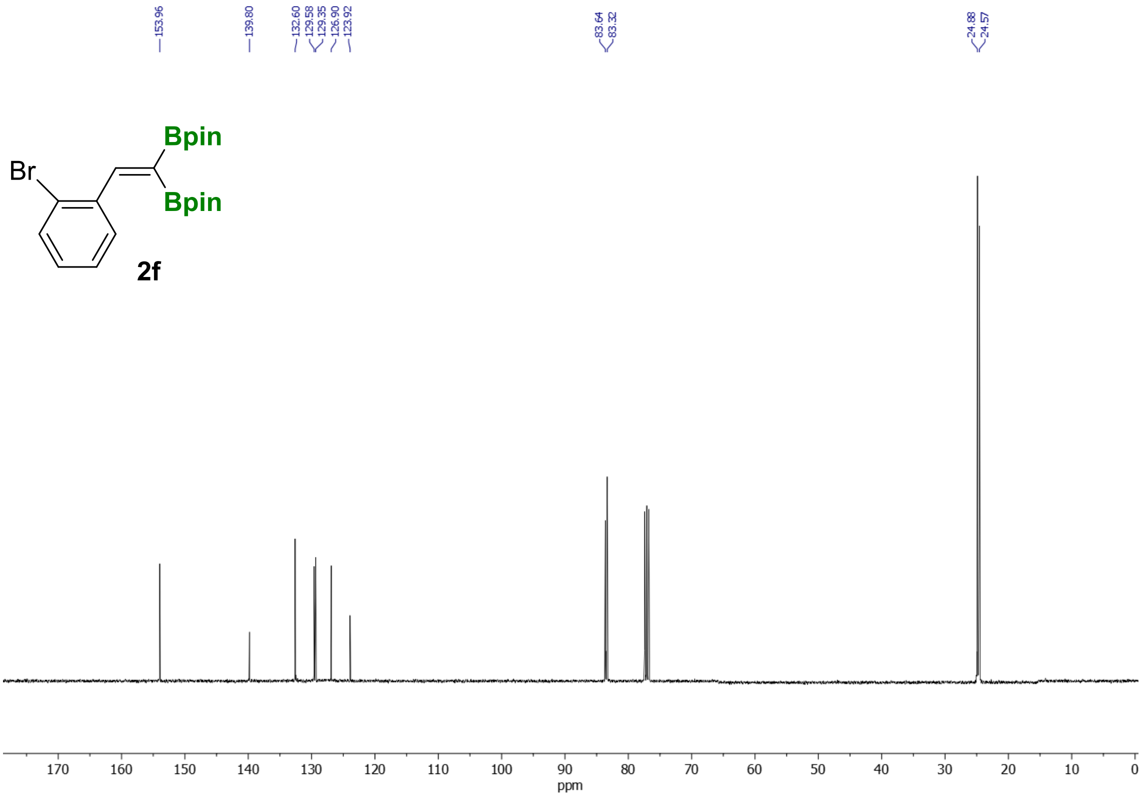

^{11}B NMR ($CDCl_3$, 128.3 MHz)

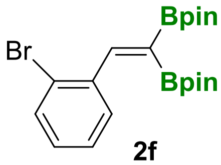

2f

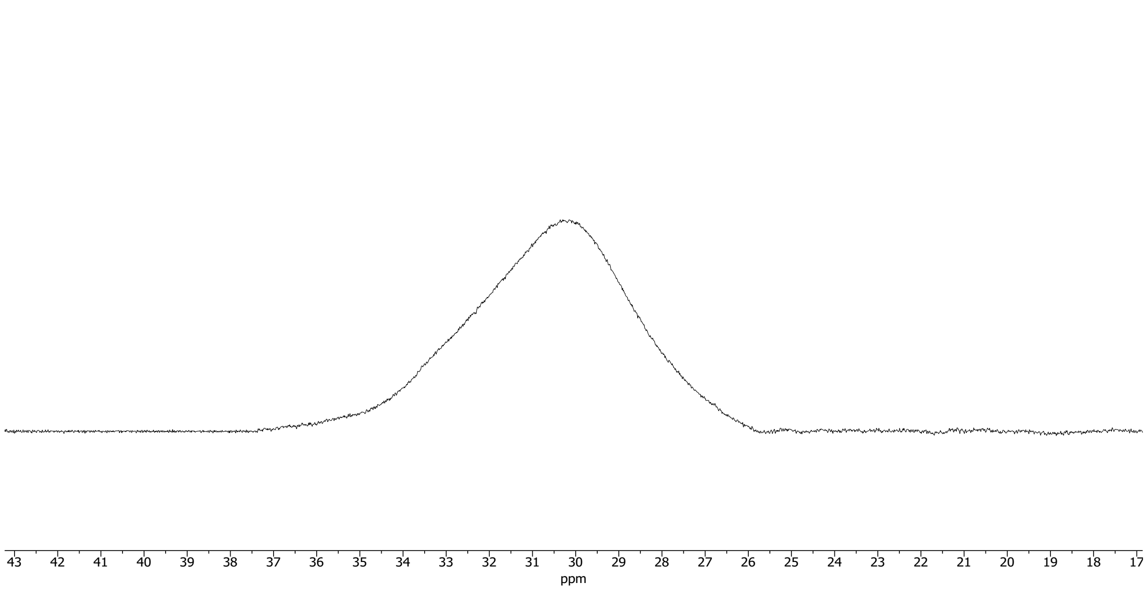

^{1}H NMR ($CDCl_3$, 400 MHz)

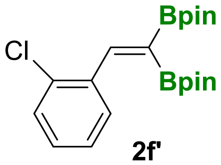

2f'

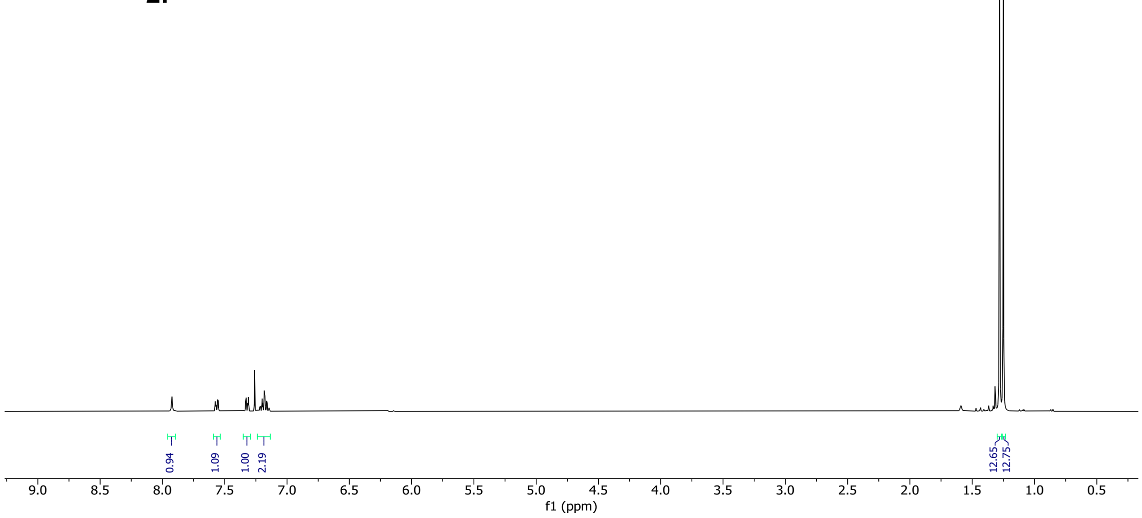

^{13}C NMR ($CDCl_3$, 100 MHz)

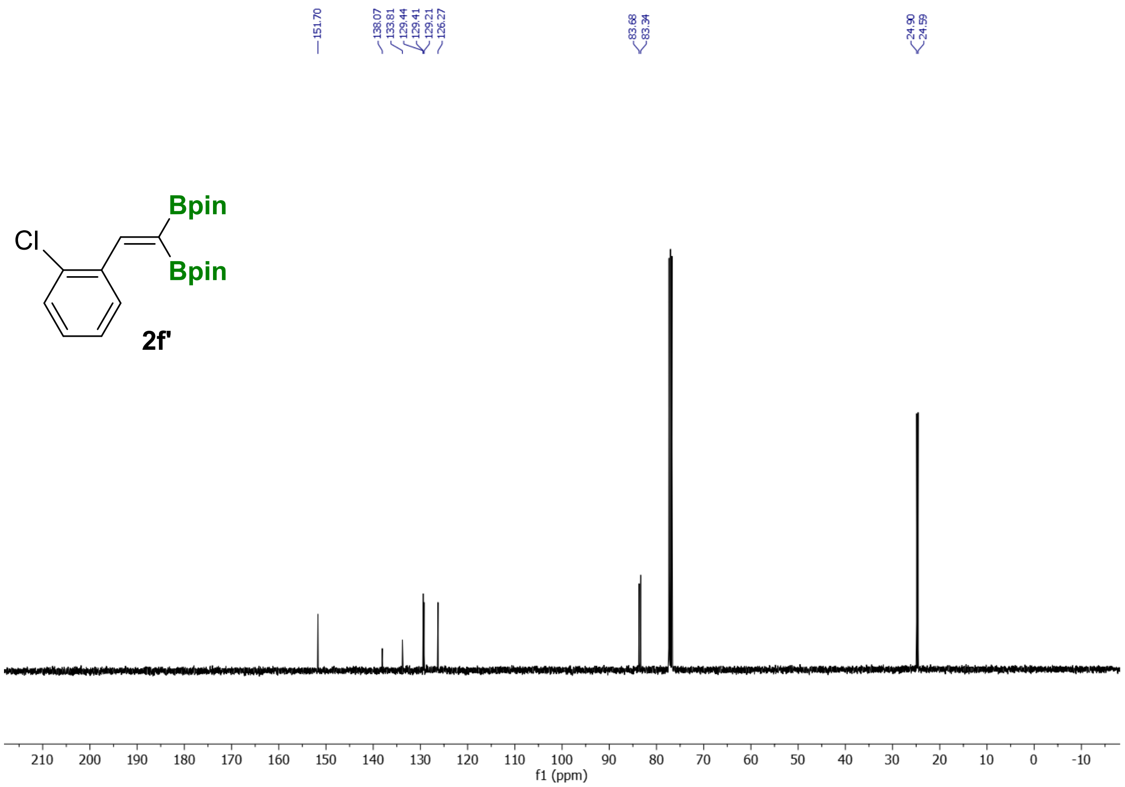

^{11}B NMR ($CDCl_3$, 128.3 MHz)

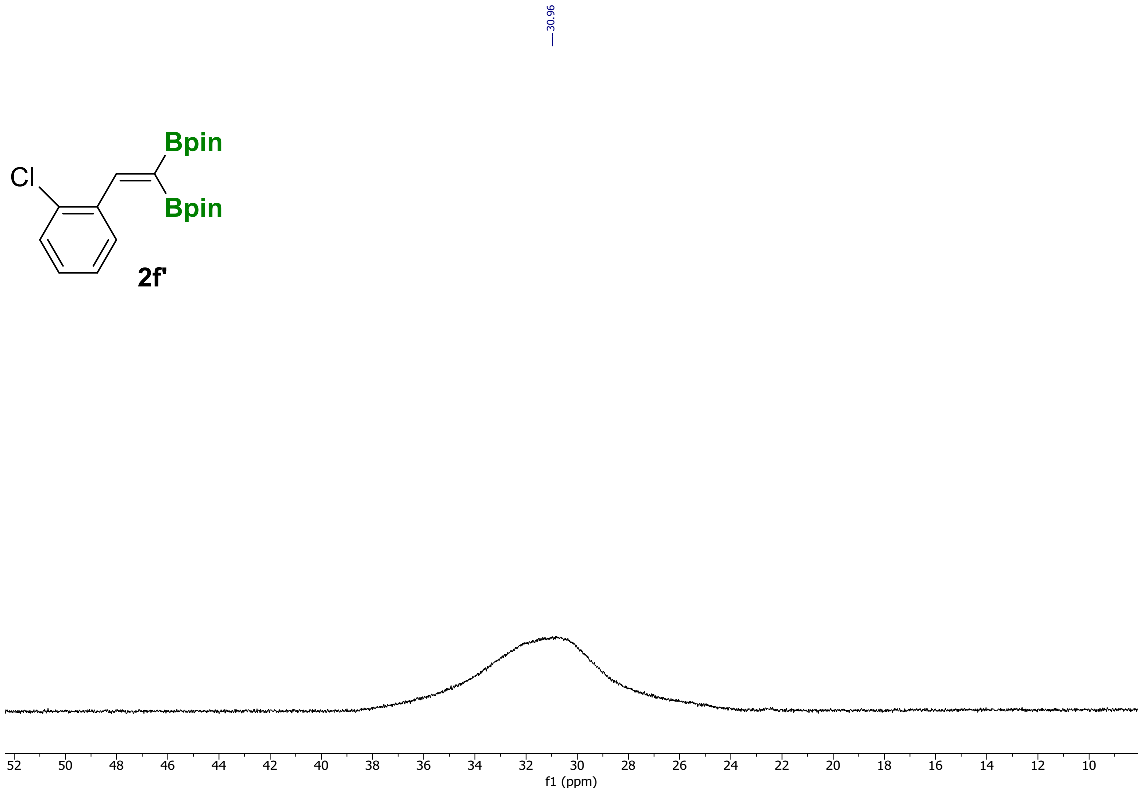

^{1}H NMR ($CDCl_3$, 400 MHz)

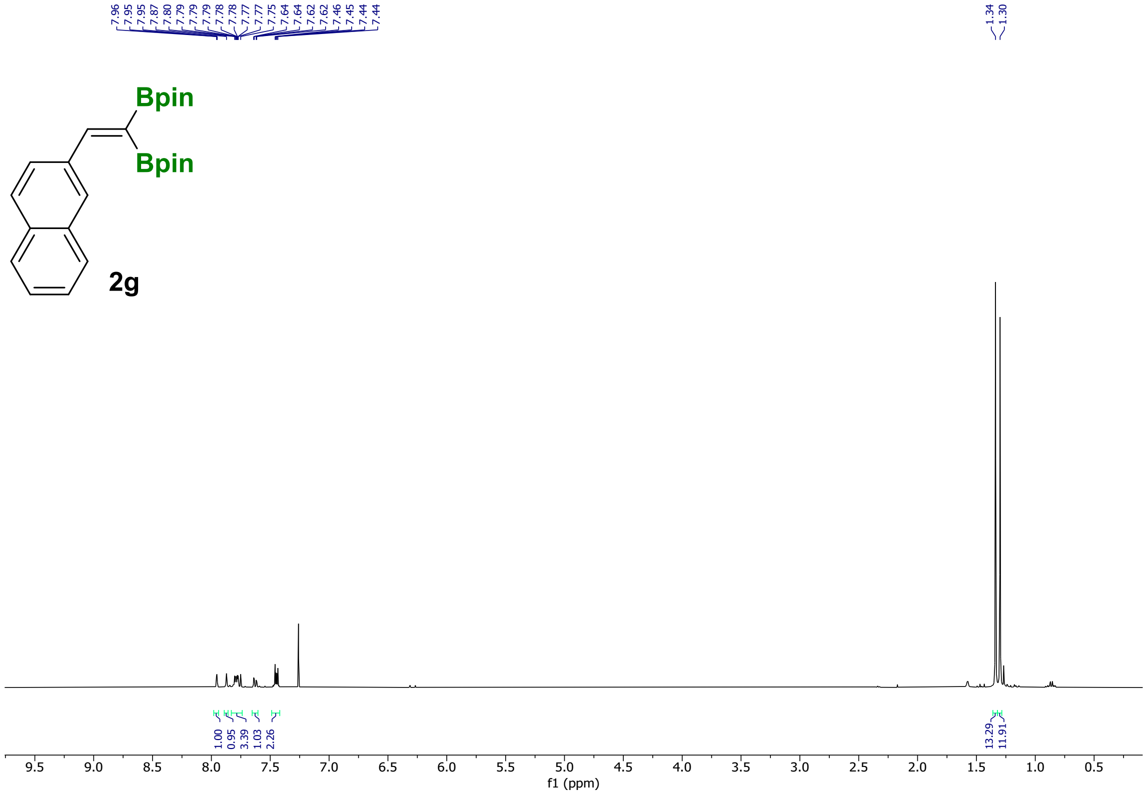

^{13}C NMR ($CDCl_3$, 100 MHz)

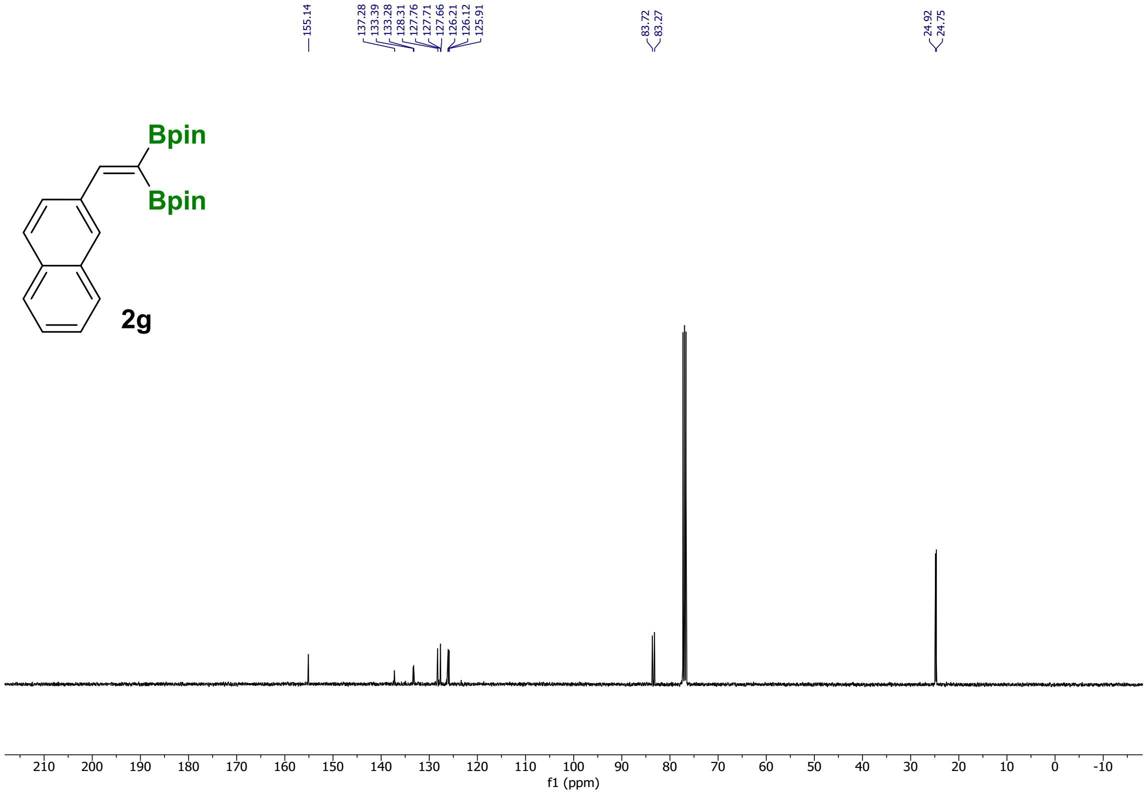

^{11}B NMR (CDCl$_3$, 128.3 MHz)

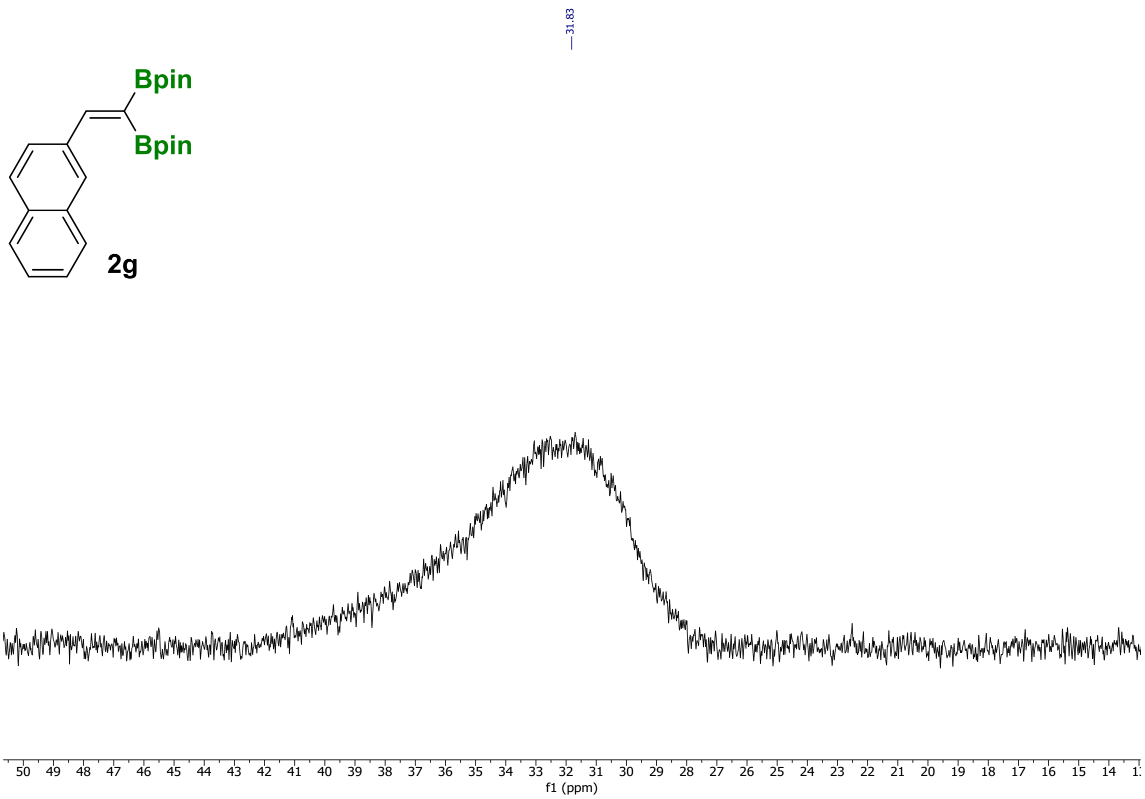

^{1}H NMR (CDCl$_3$, 400 MHz)

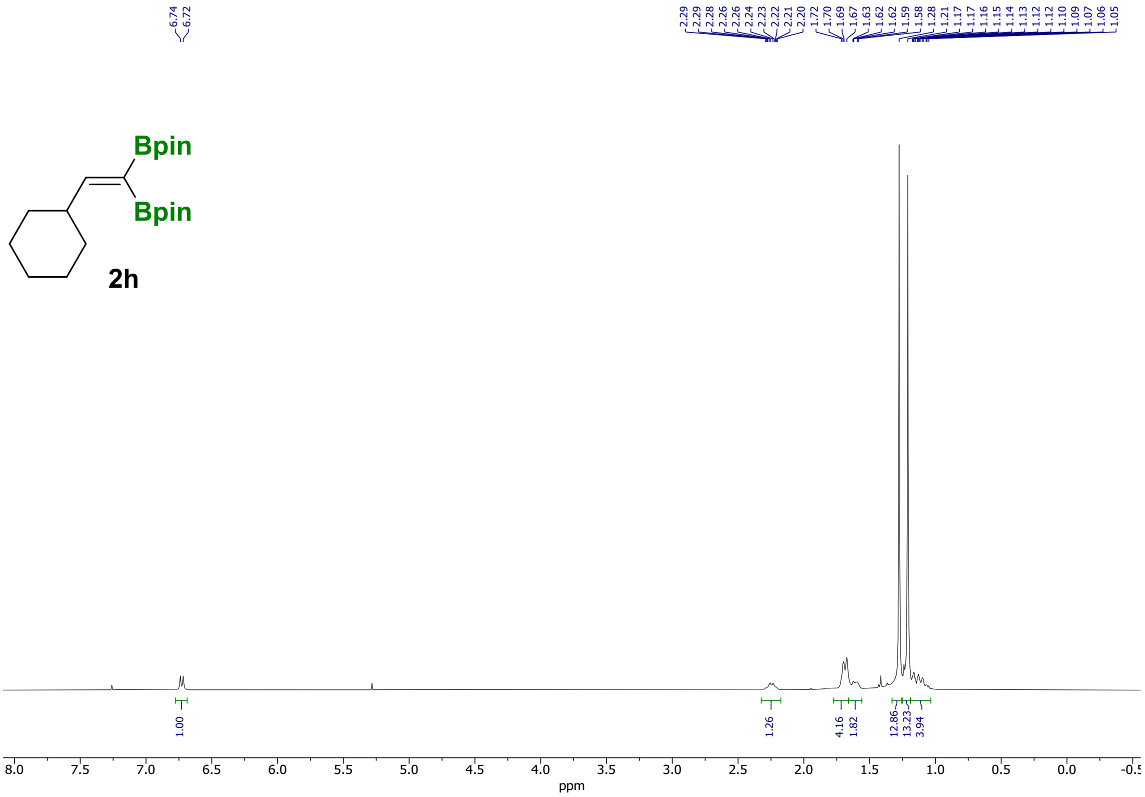

^{13}C NMR ($CDCl_3$, 100 MHz)

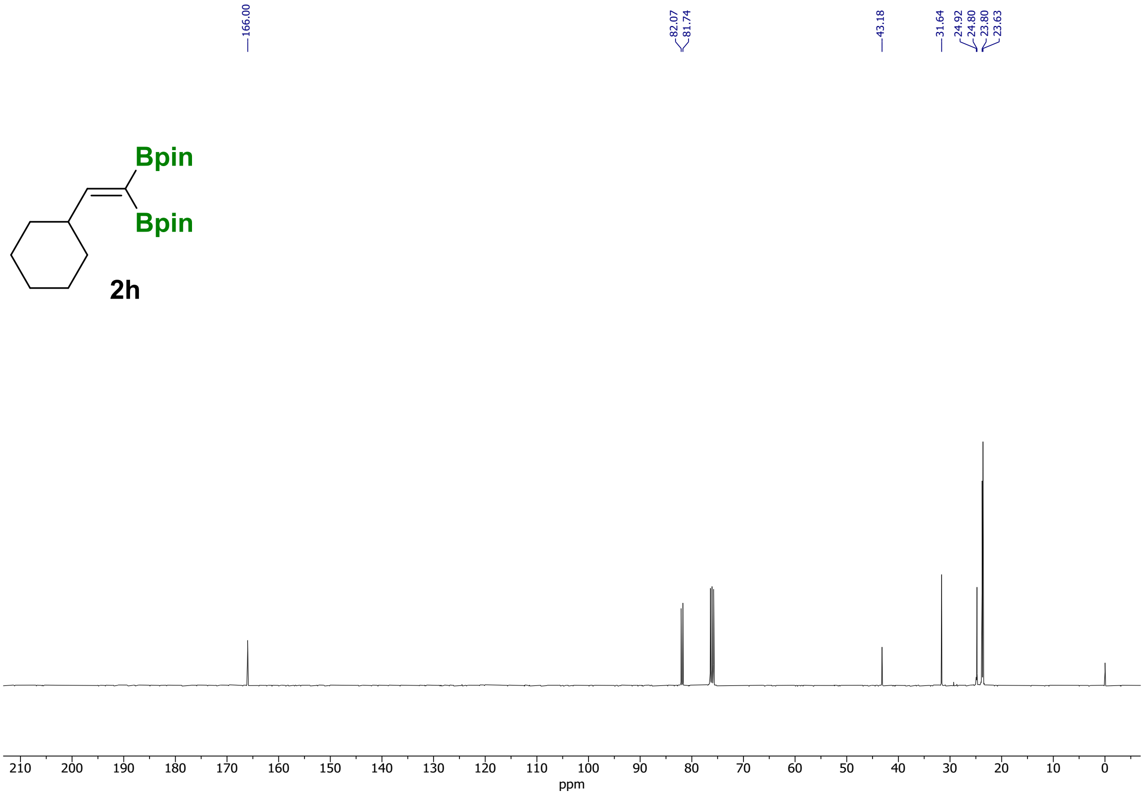

^{11}B NMR ($CDCl_3$, 128.3 MHz)

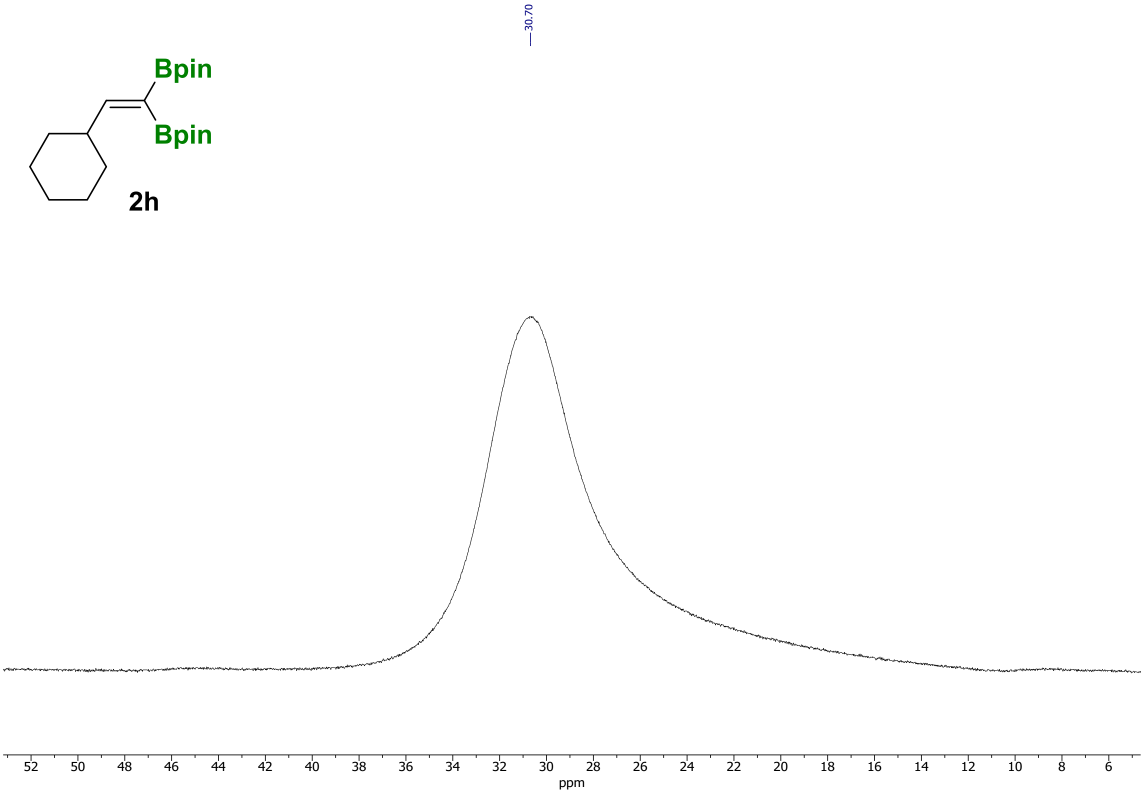

^{1}H NMR ($CDCl_3$, 400 MHz)

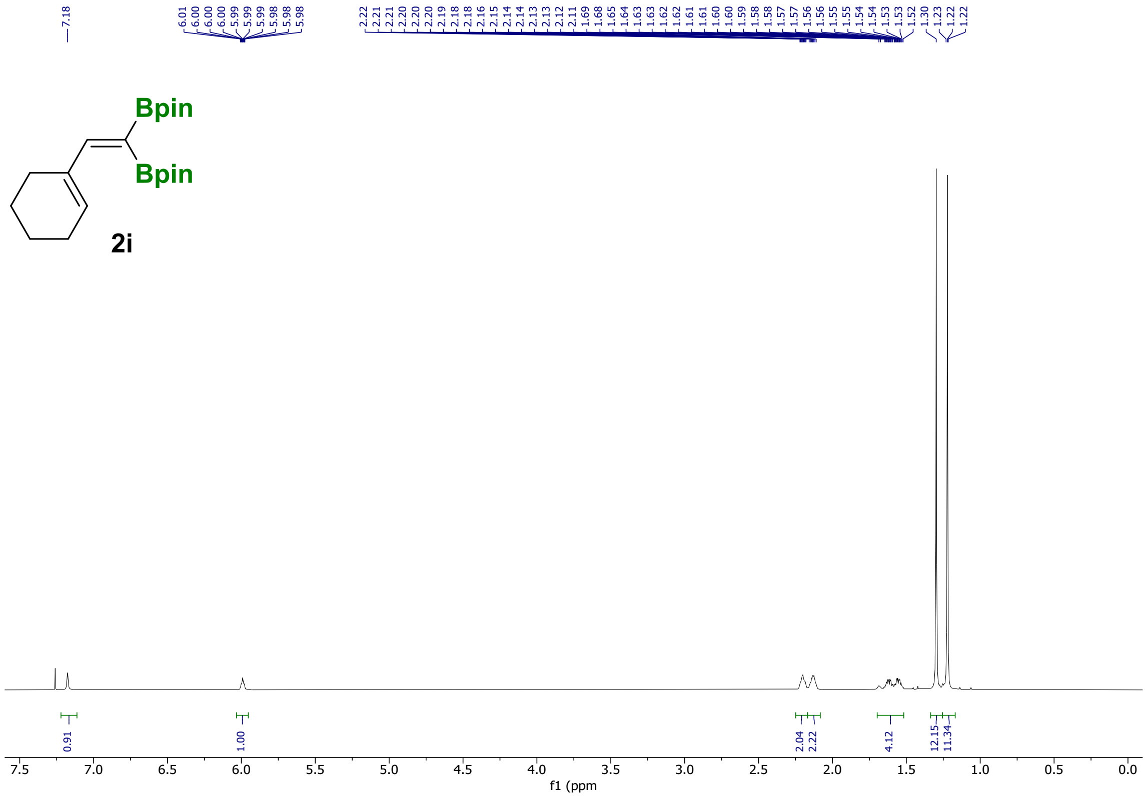

^{13}C NMR ($CDCl_3$, 100 MHz)

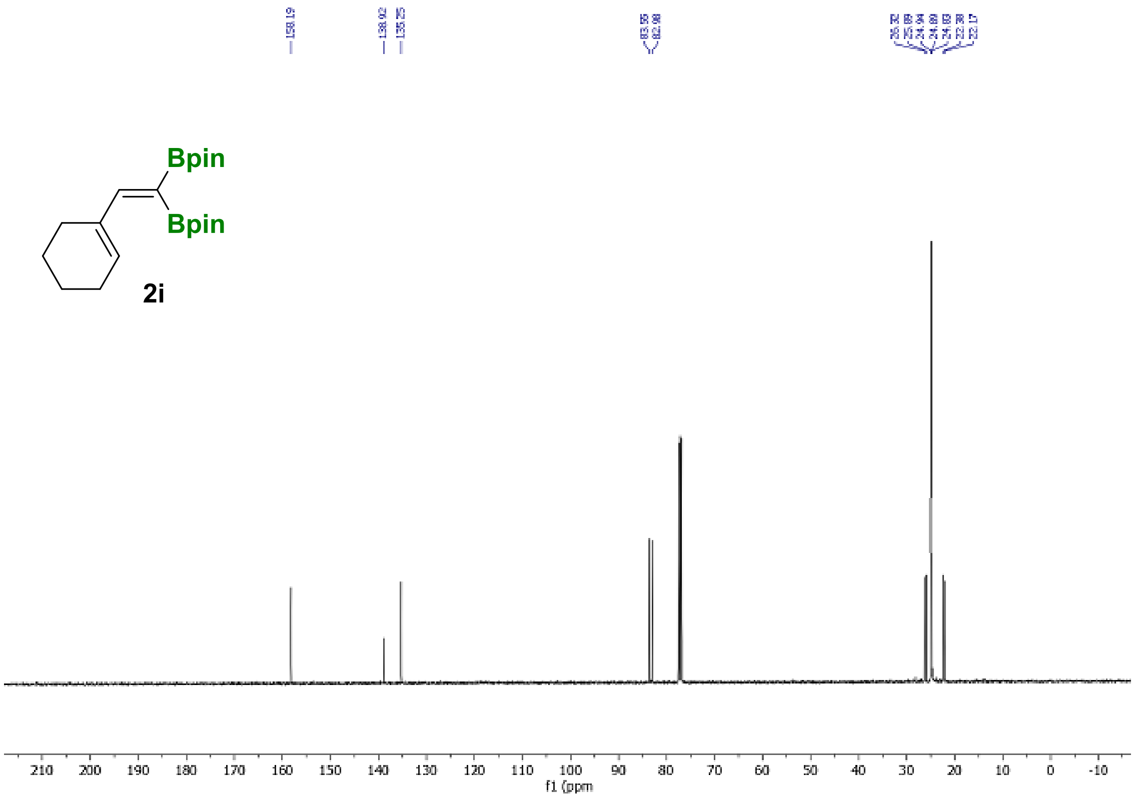

^{11}B NMR (CDCl$_3$, 128.3 MHz)

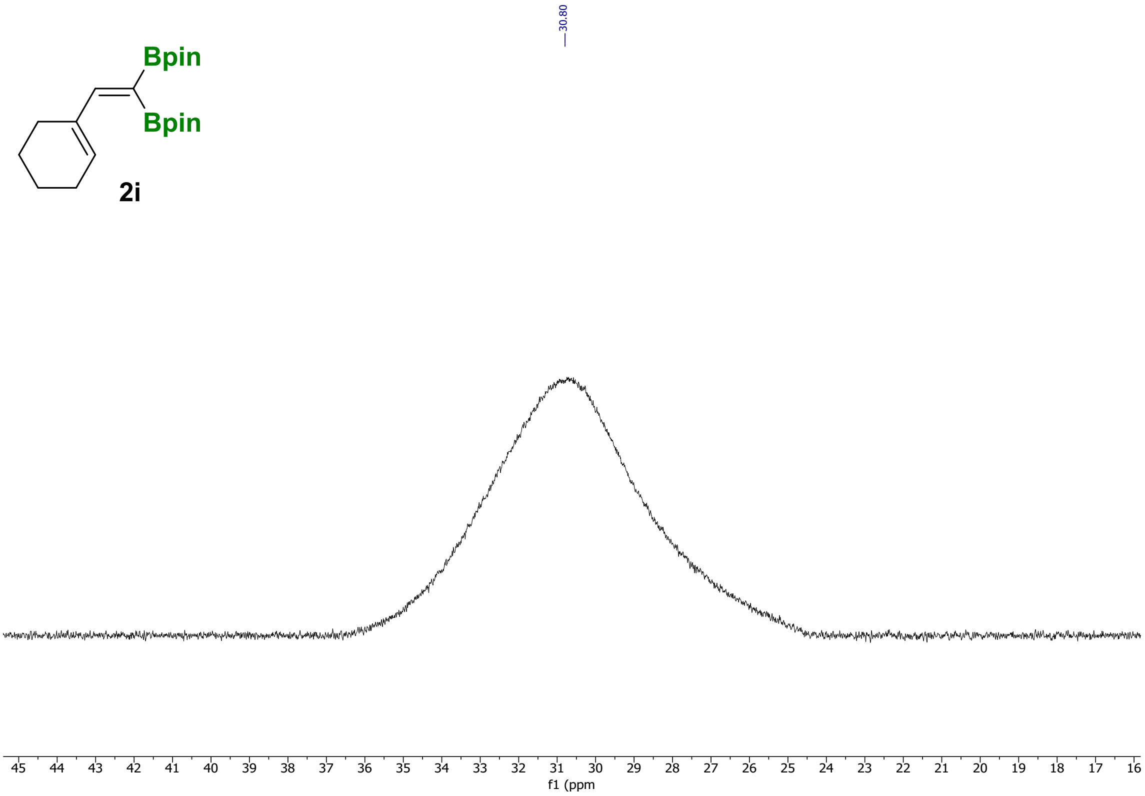

^{1}H NMR (CDCl$_3$, 400 MHz)

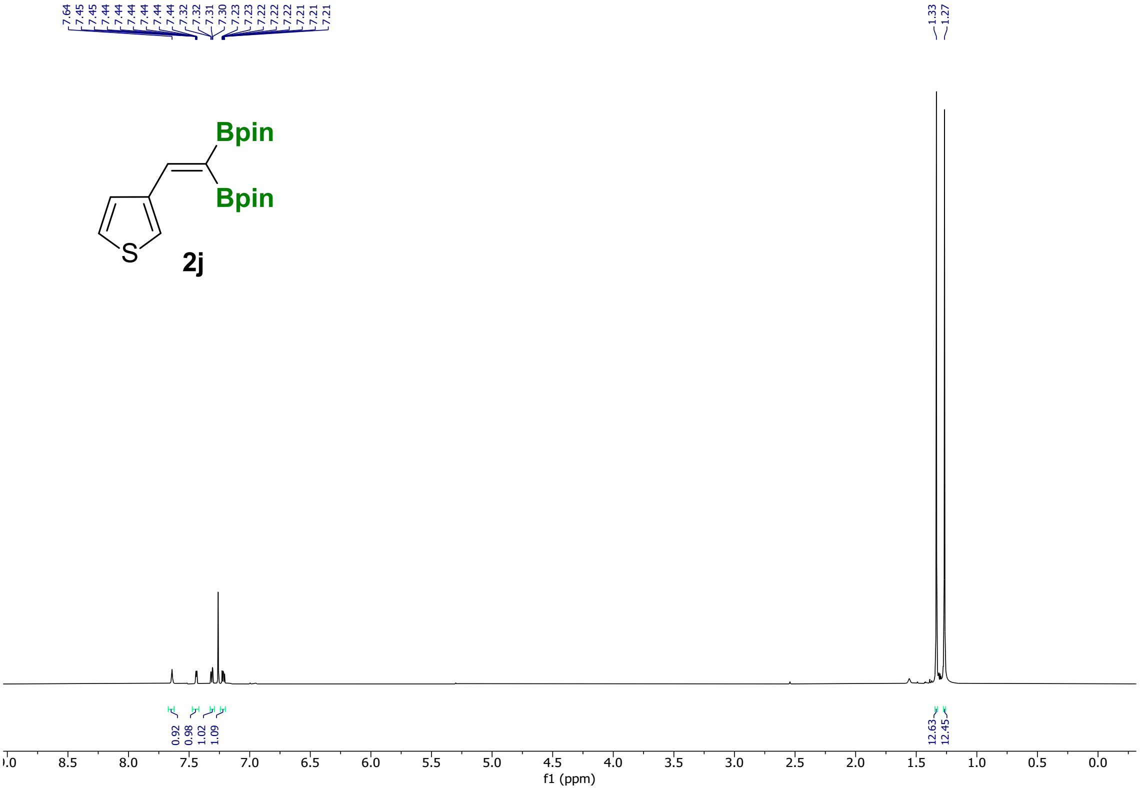

^{13}C NMR ($CDCl_3$, 100 MHz)

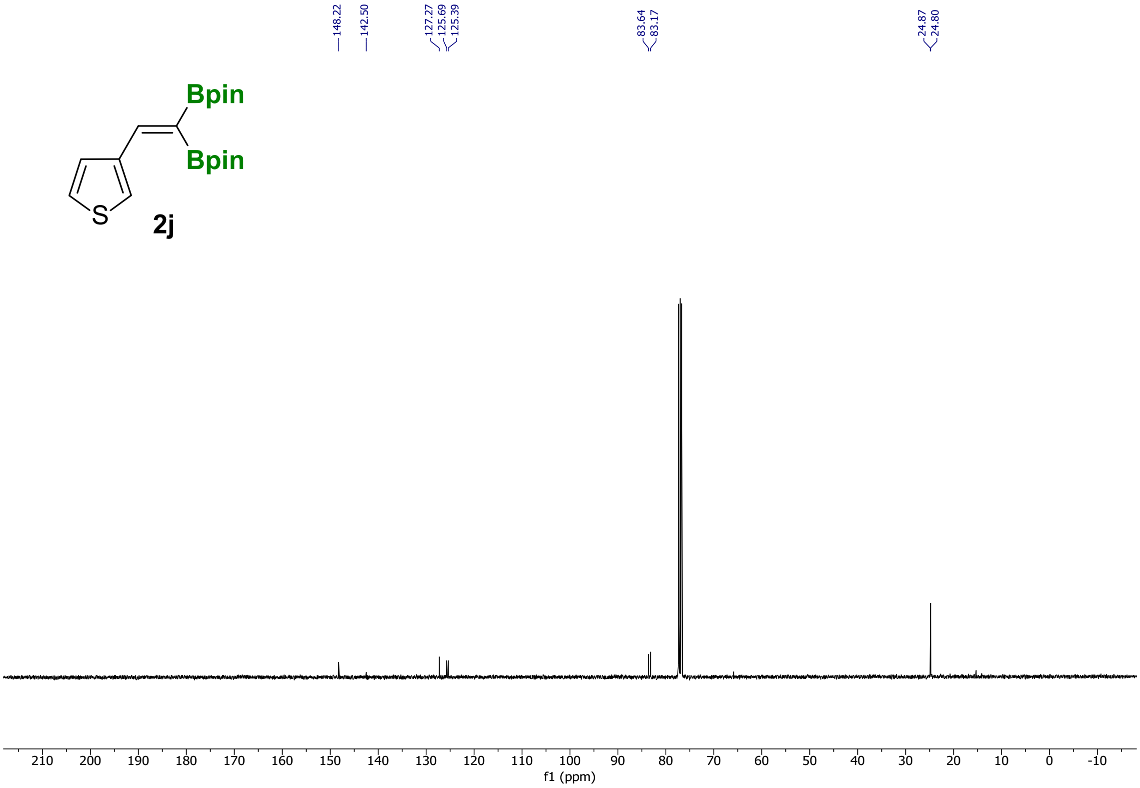

^{11}B NMR ($CDCl_3$, 128.3 MHz)

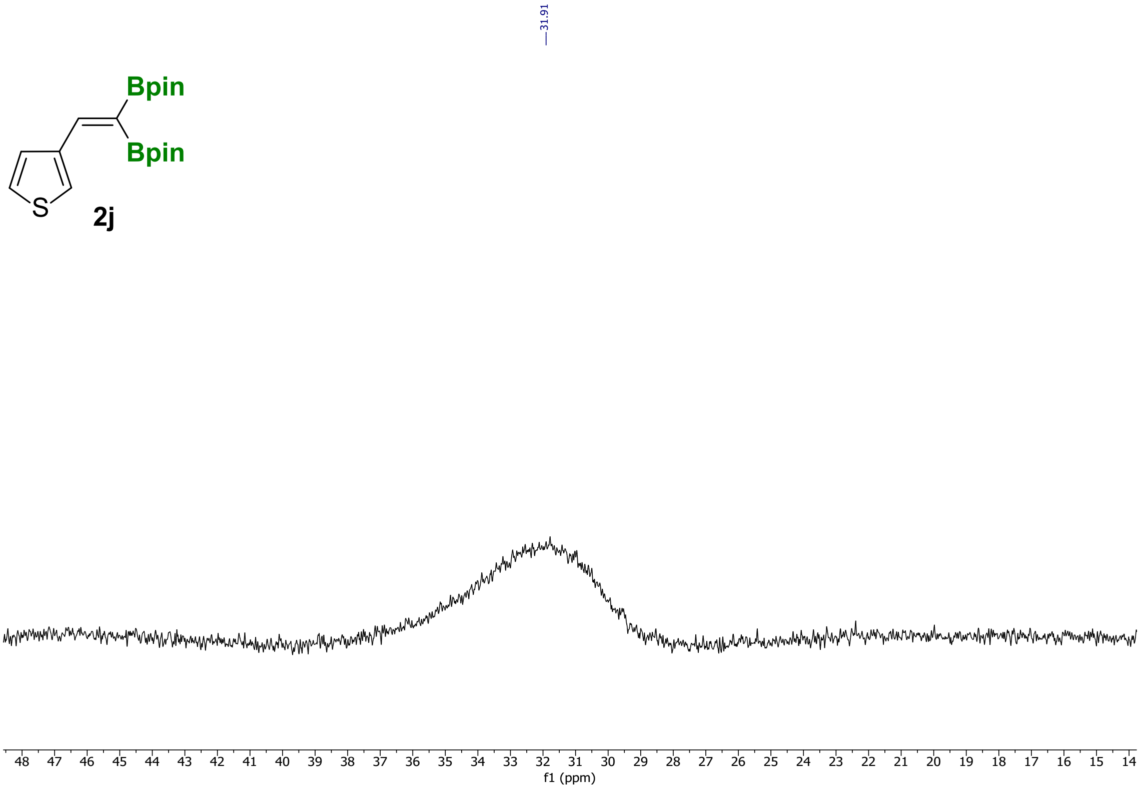

^{1}H NMR ($CDCl_3$, 400 MHz)

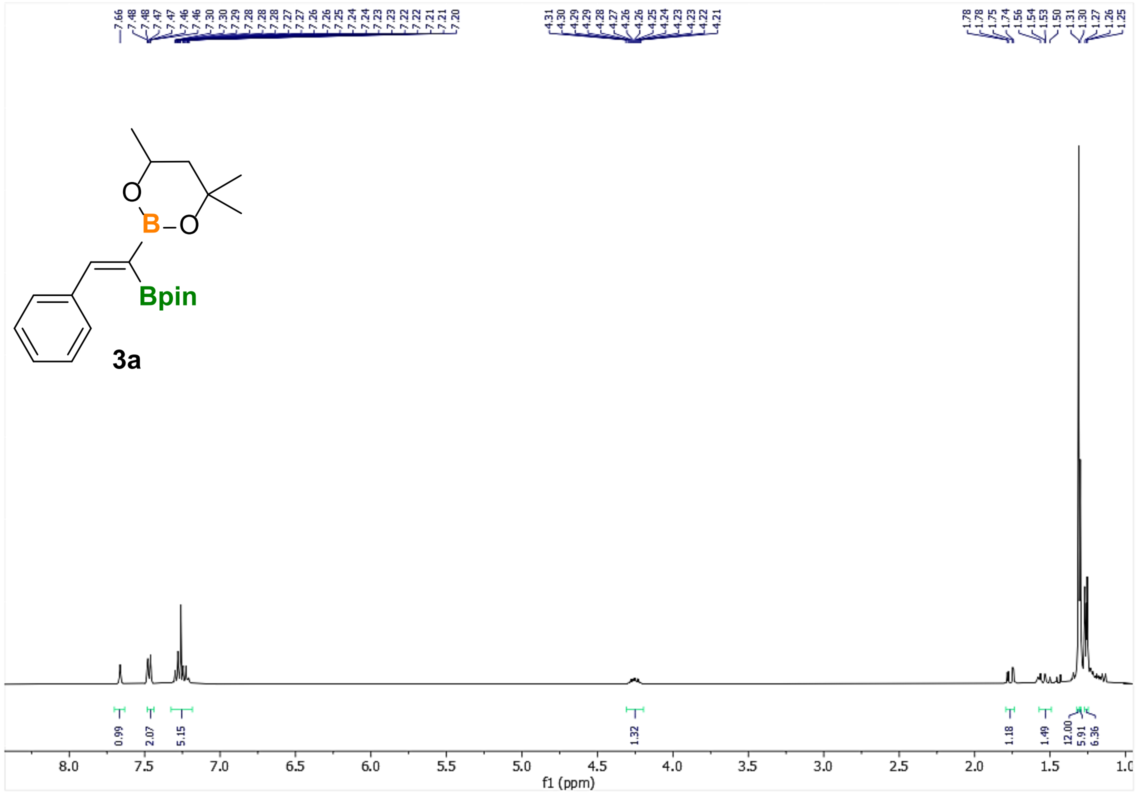

^{13}C NMR ($CDCl_3$, 100 MHz)

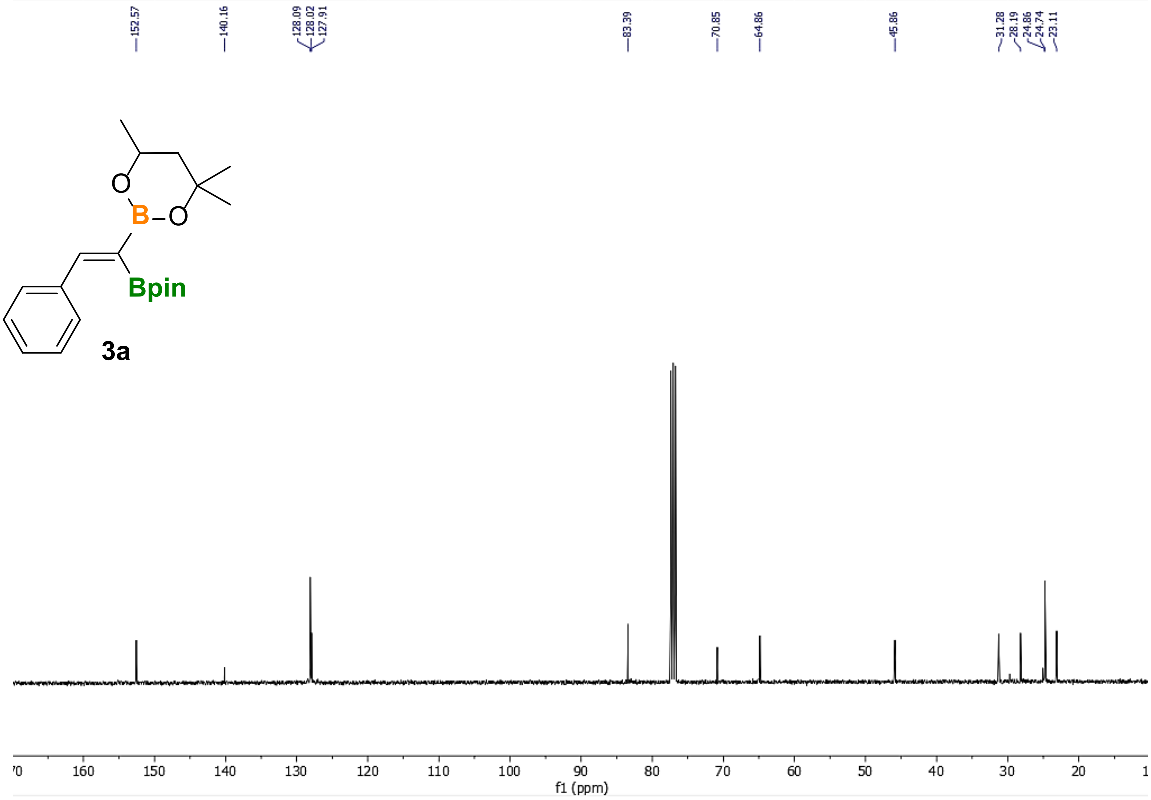

11B NMR (CDCl$_3$, 128.3 MHz)

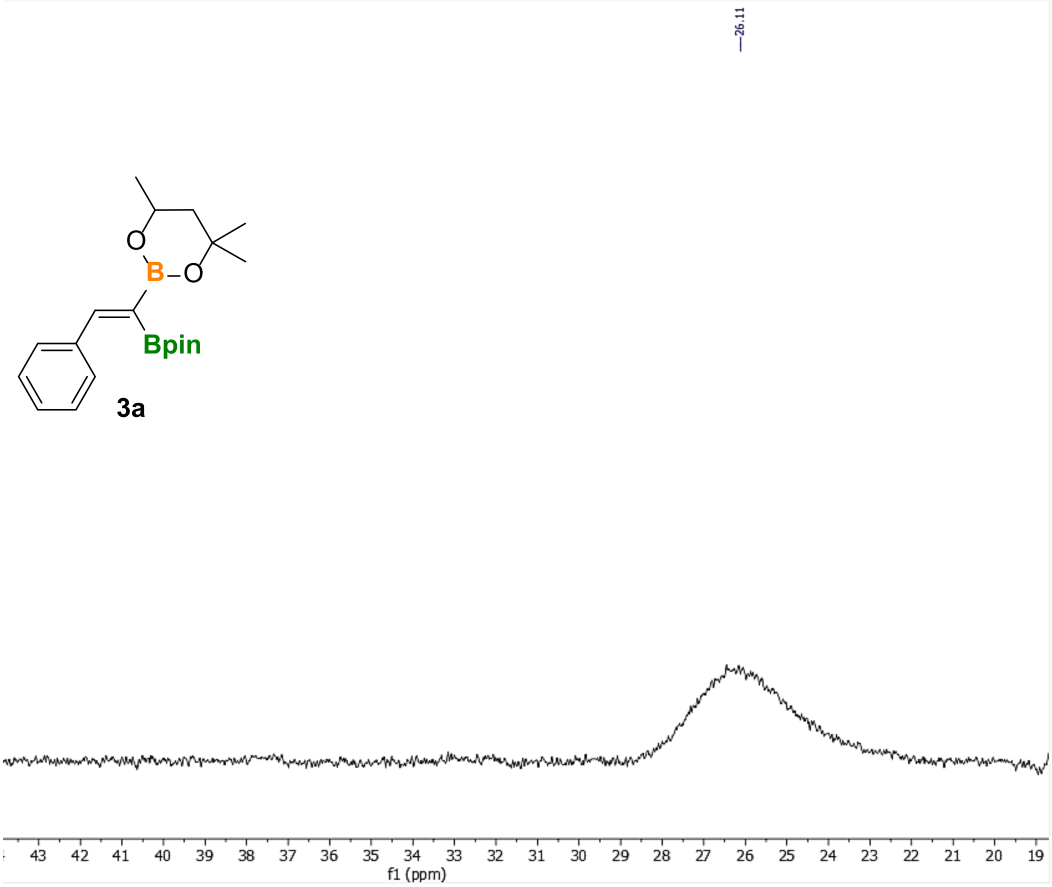

^{1}H NMR (CDCl$_3$, 400 MHz)

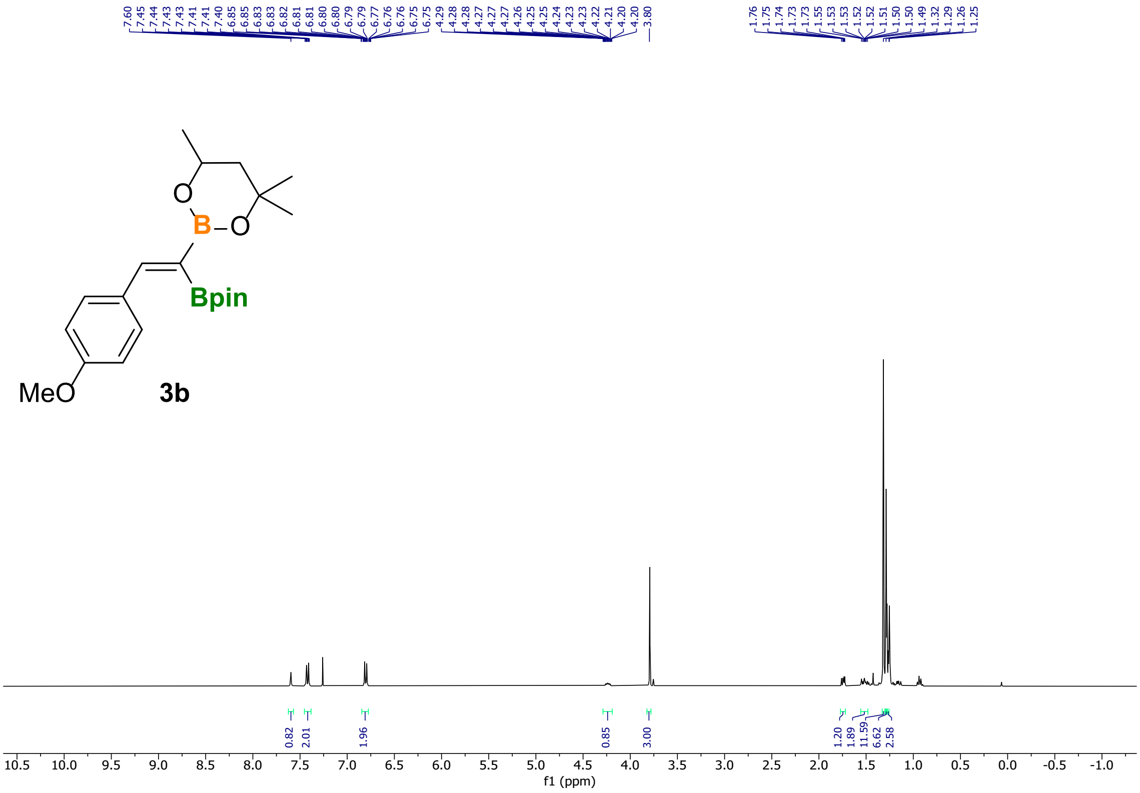

^{13}C NMR ($CDCl_3$, 100 MHz)

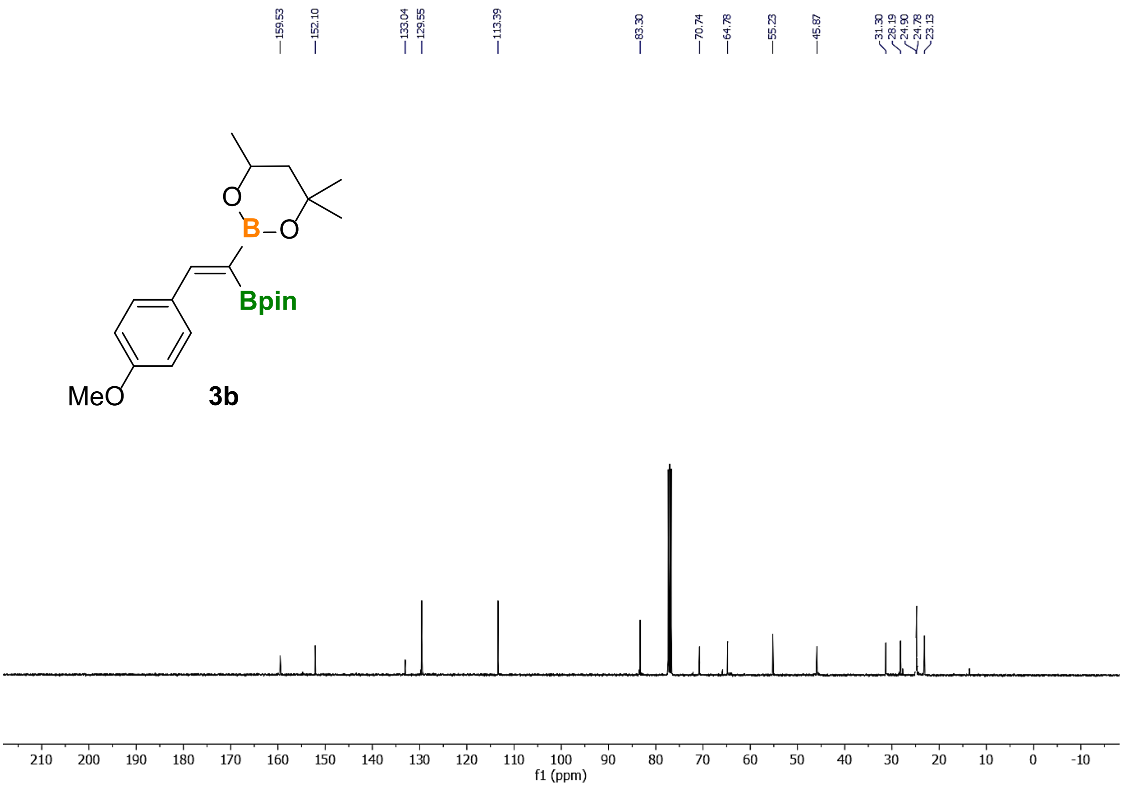

^{11}B NMR ($CDCl_3$, 128.3 MHz)

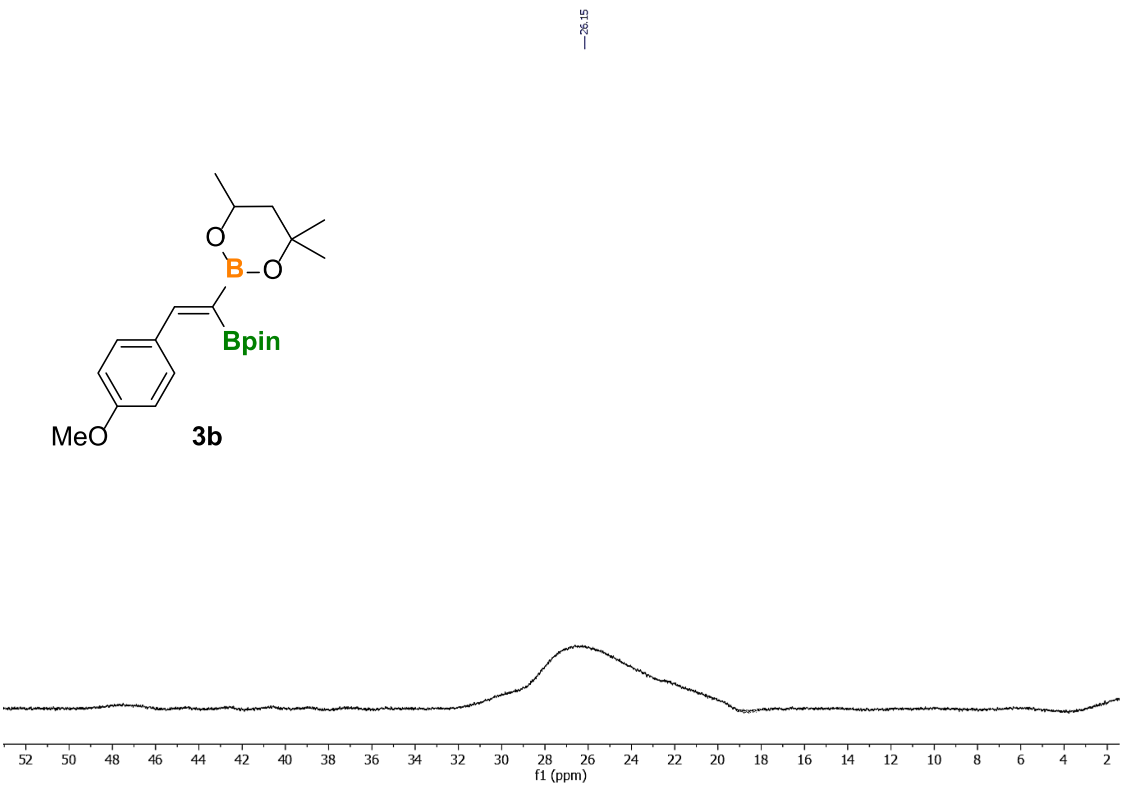

^{1}H NMR (CDCl$_3$, 400 MHz)

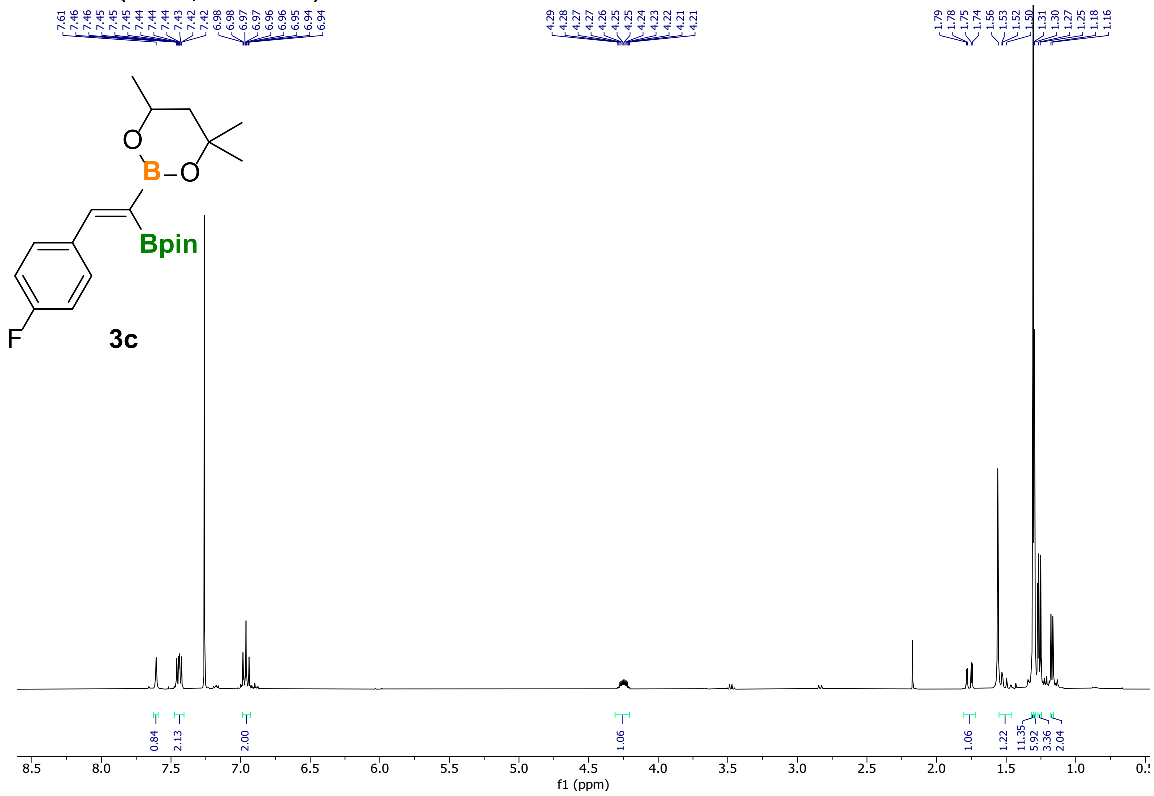

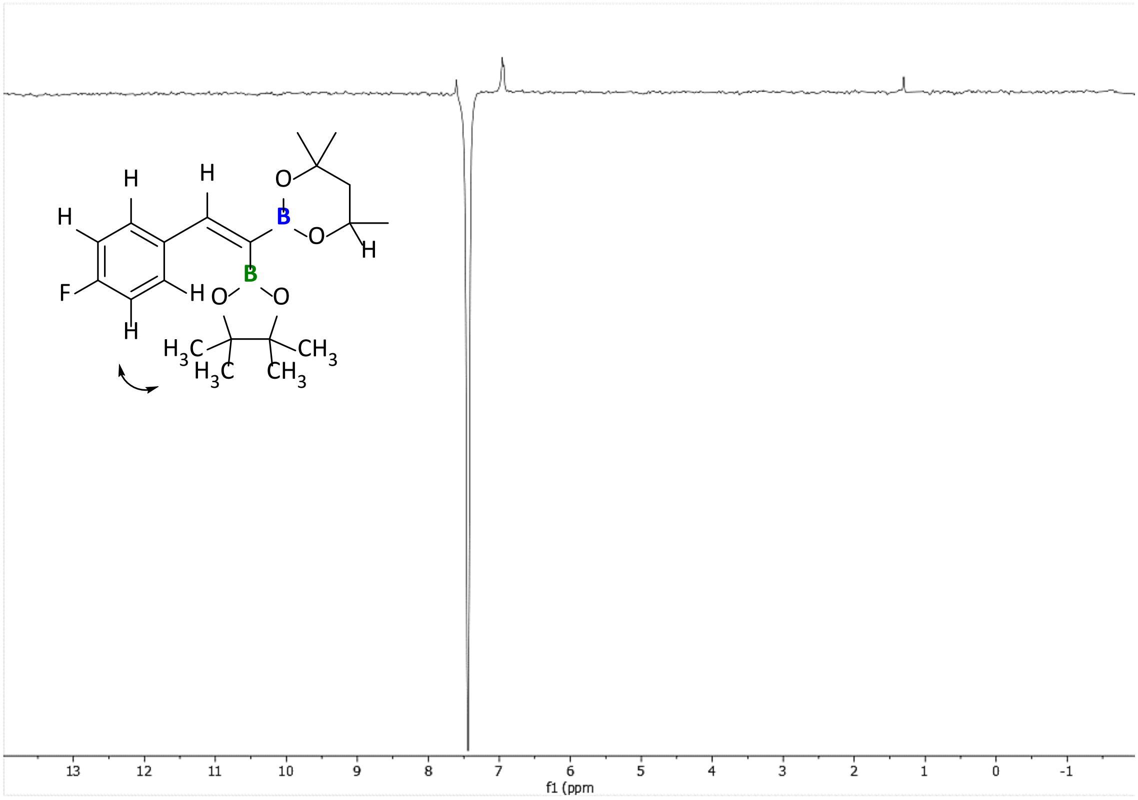

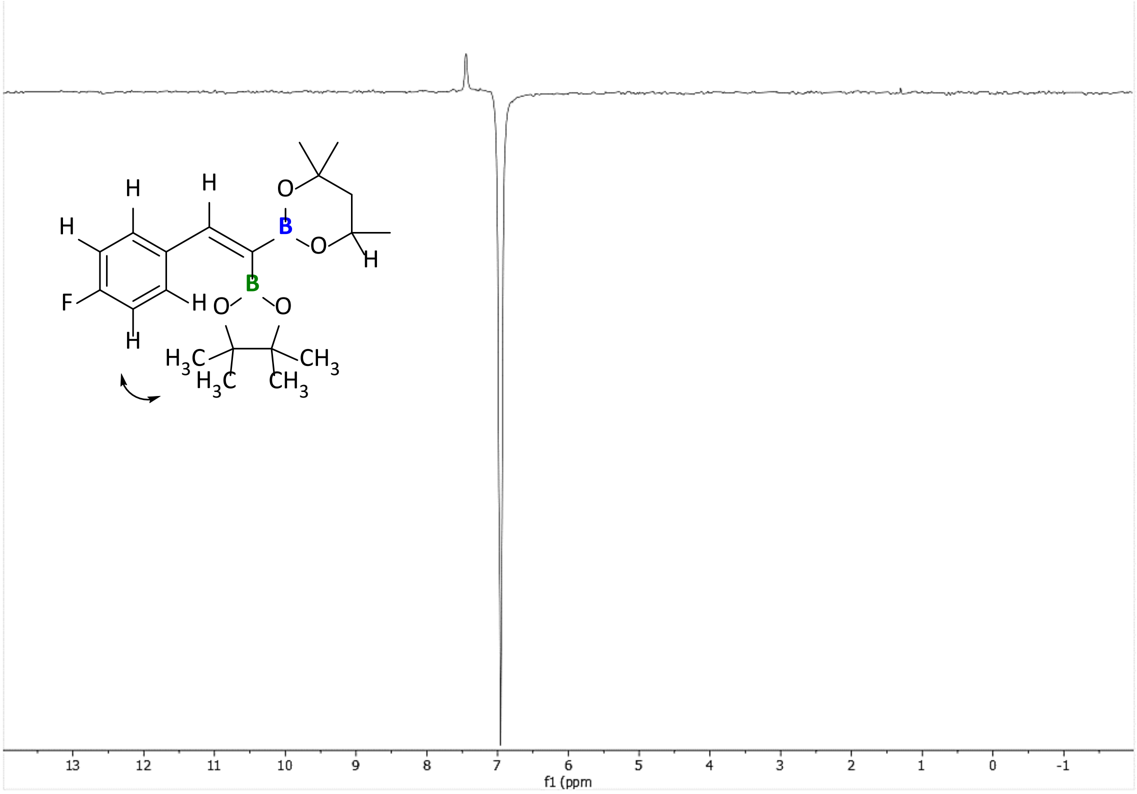
H
H
H
F
H
H
B
O
O
H
B
O
O
H3C
H3C
CH3
CH3
13
12
11
10
9
8
7
6
5
4
3
2
1
0
-1
f1 (ppm

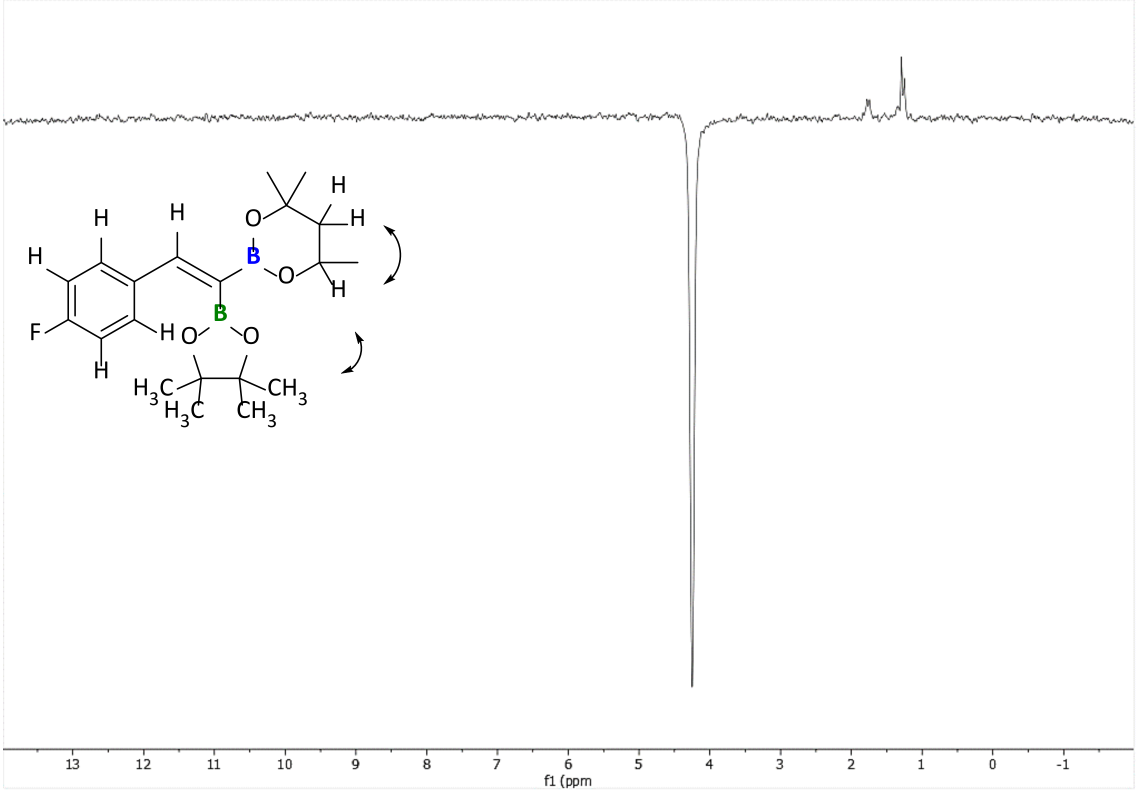
H
H
H
F
H
H
B
O
O
H
H
H
B
O
O
H3C
H3C
CH3
CH3
13
12
11
10
9
8
7
6
5
4
3
2
1
0
-1
f1 (ppm

^{13}C NMR (CDCl$_3$, 100 MHz)

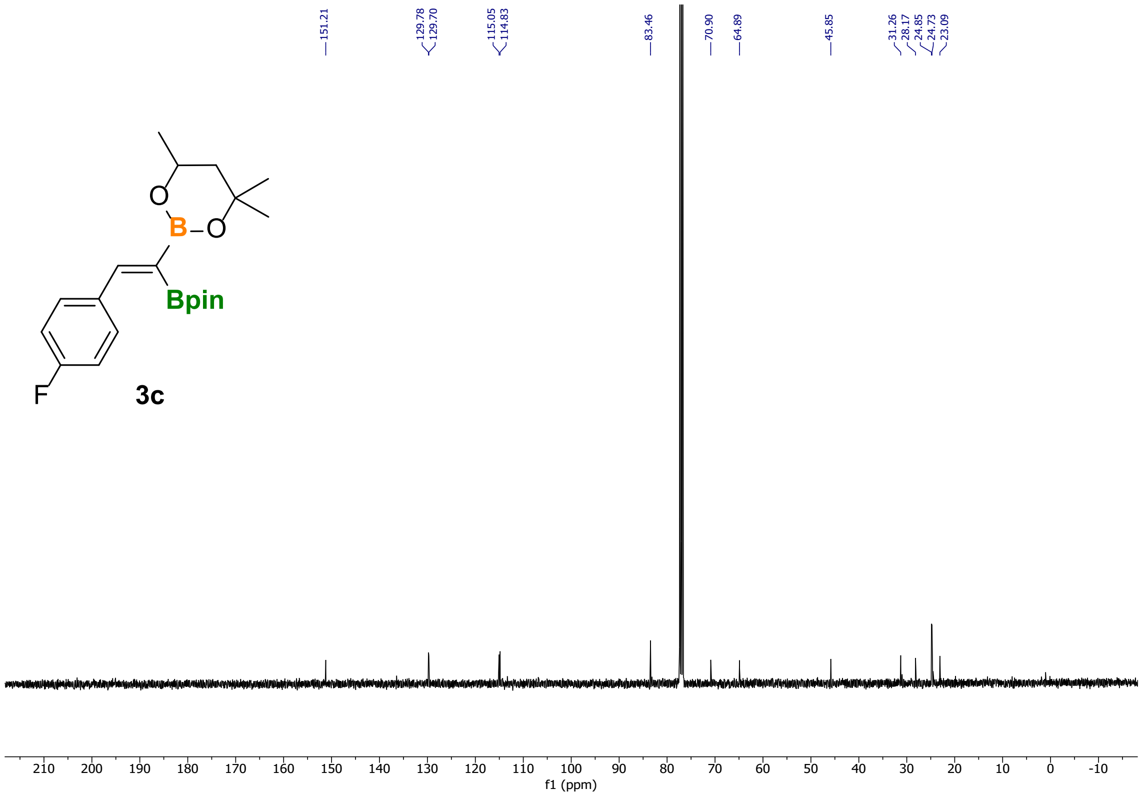

^{11}B NMR (CDCl$_3$, 128.3 MHz)

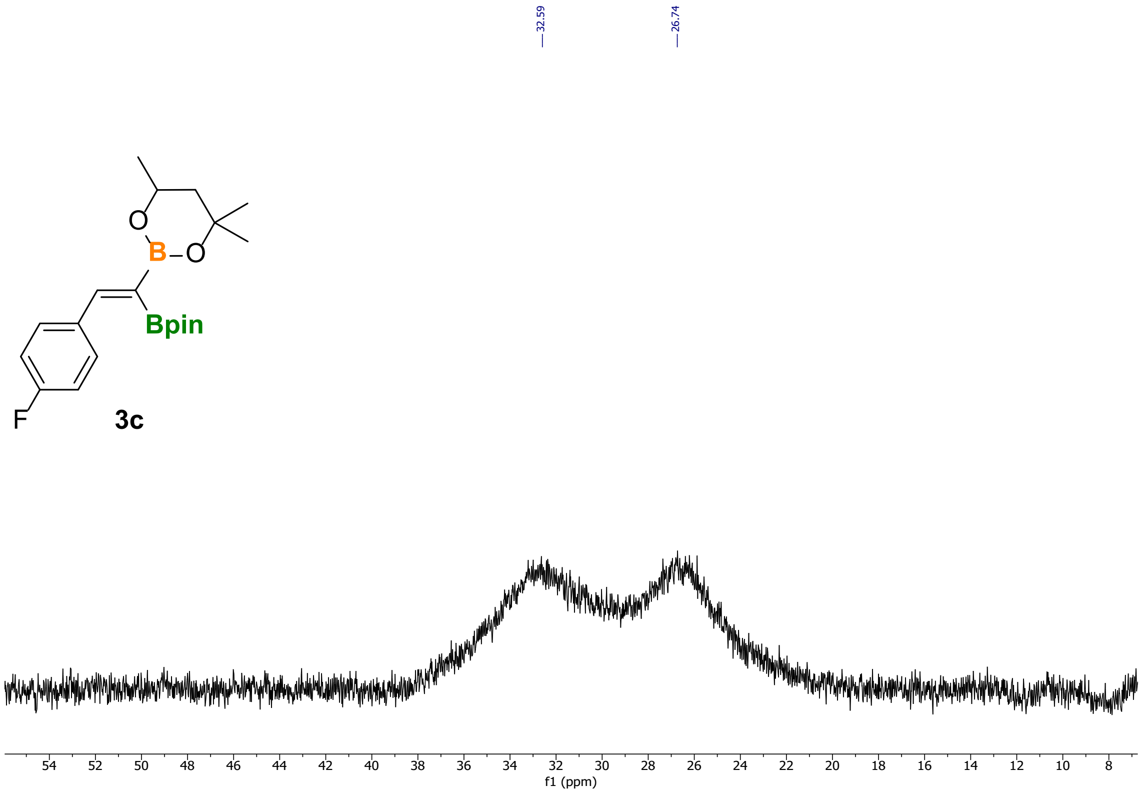

^{1}H NMR ($CDCl_3$, 400 MHz)

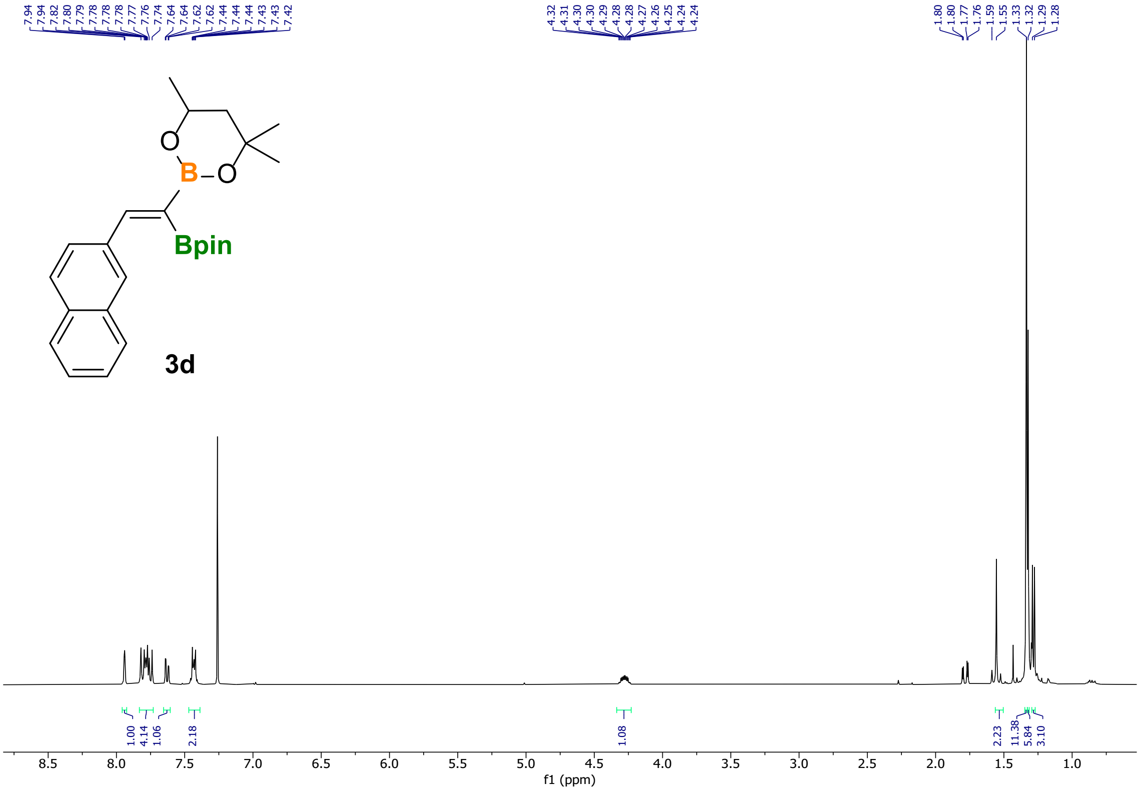

^{13}**C NMR** ($CDCl_3$, 100 MHz)

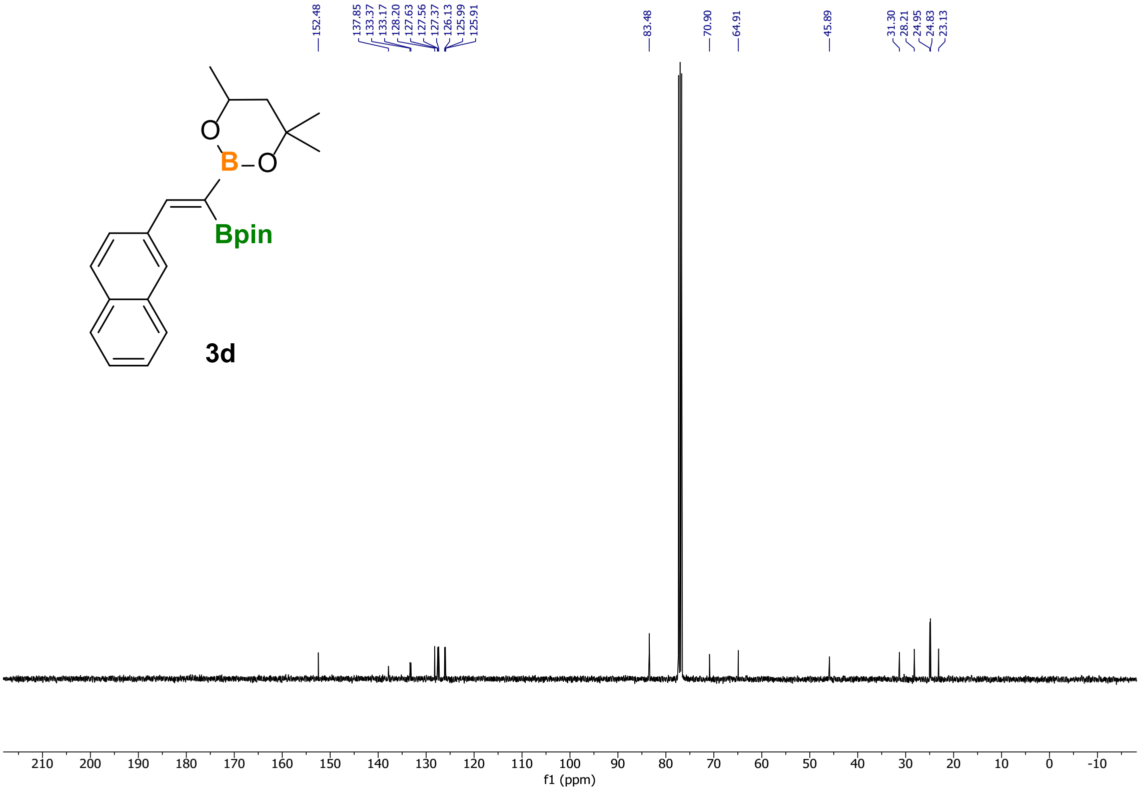

^{11}B NMR ($CDCl_3$, 128.3 MHz)

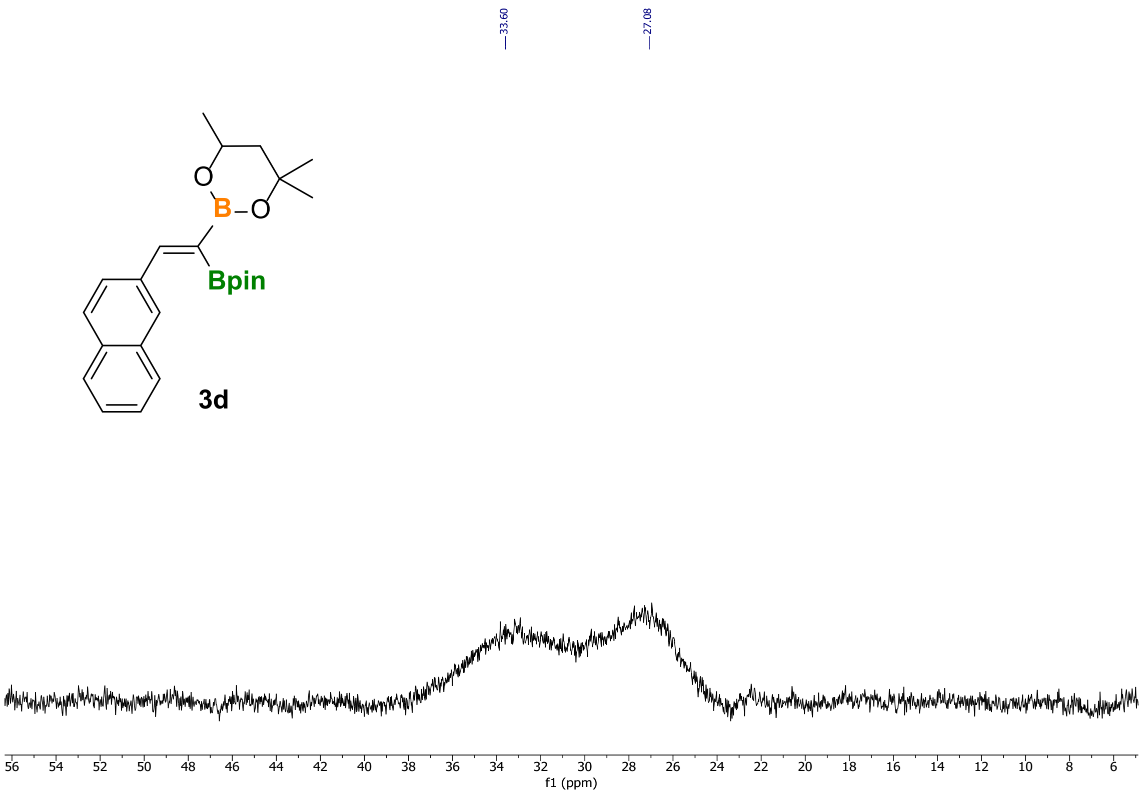

^{1}H NMR ($CDCl_3$, 400 MHz)

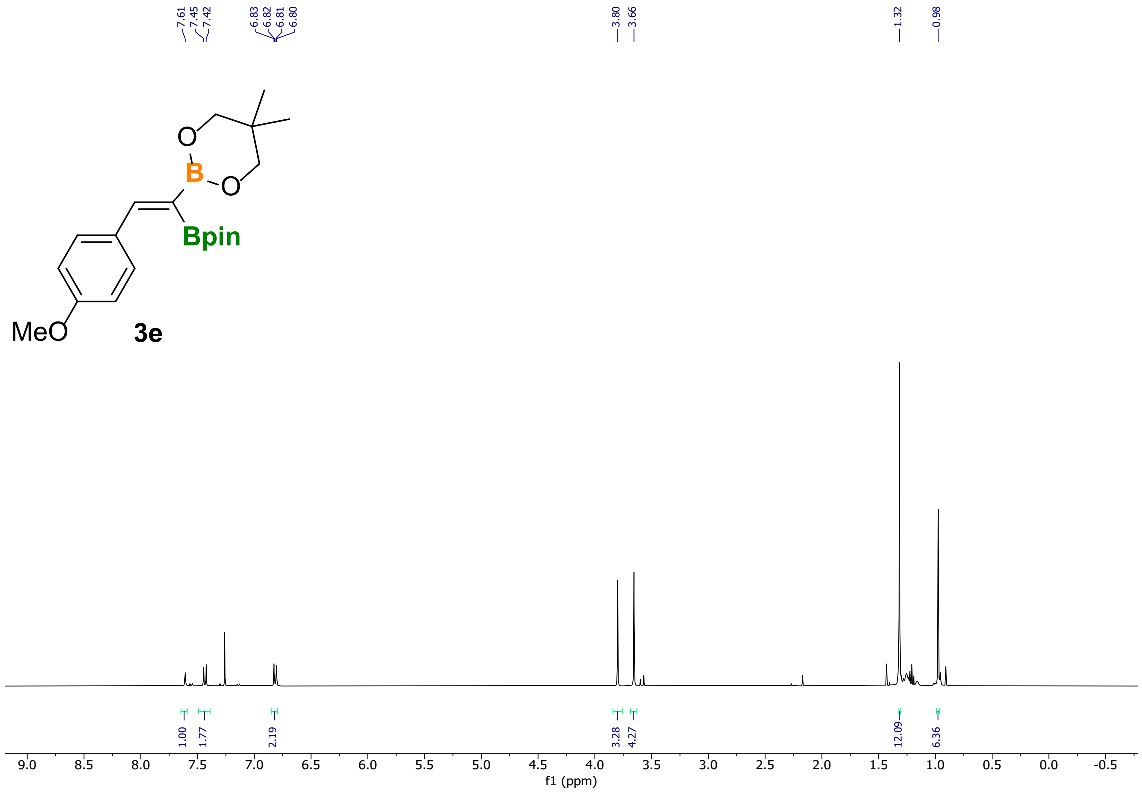

^{13}C NMR (CDCl$_3$, 100 MHz)

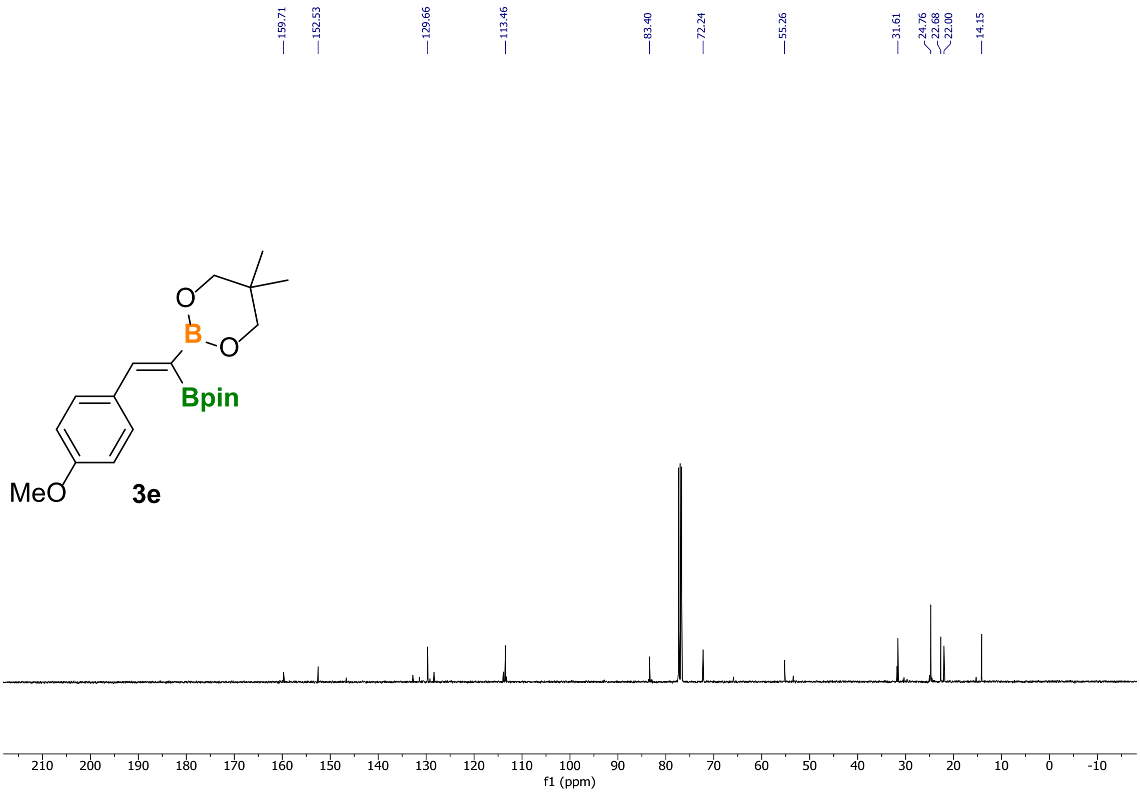

^{11}B NMR (CDCl$_3$, 128.3 MHz)

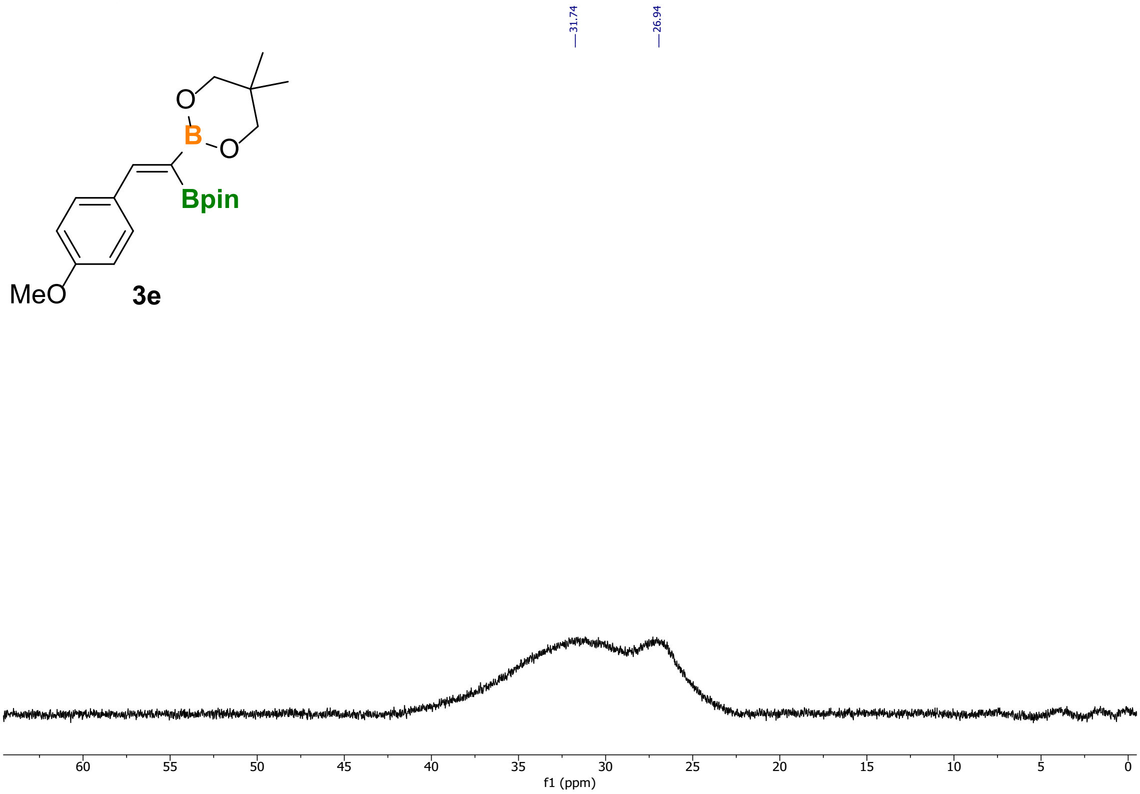

^{1}H NMR (CDCl$_3$, 400 MHz)

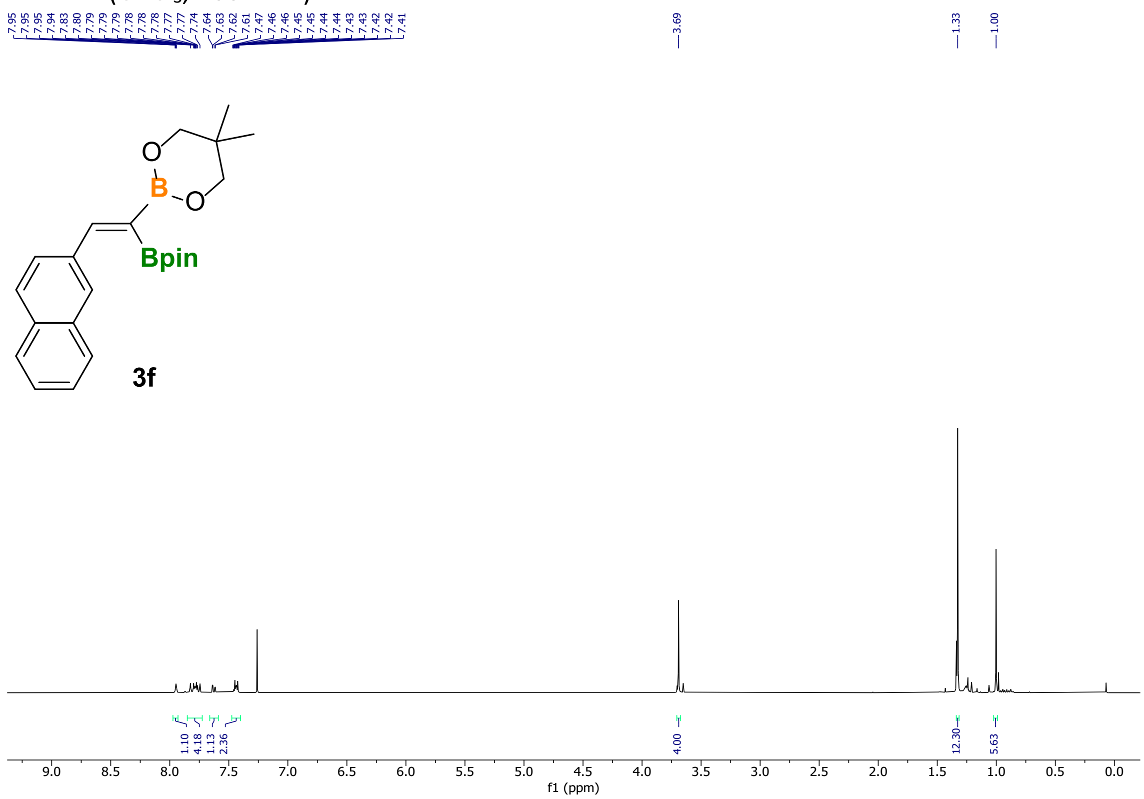

^{13}C NMR (CDCl$_3$, 100 MHz)

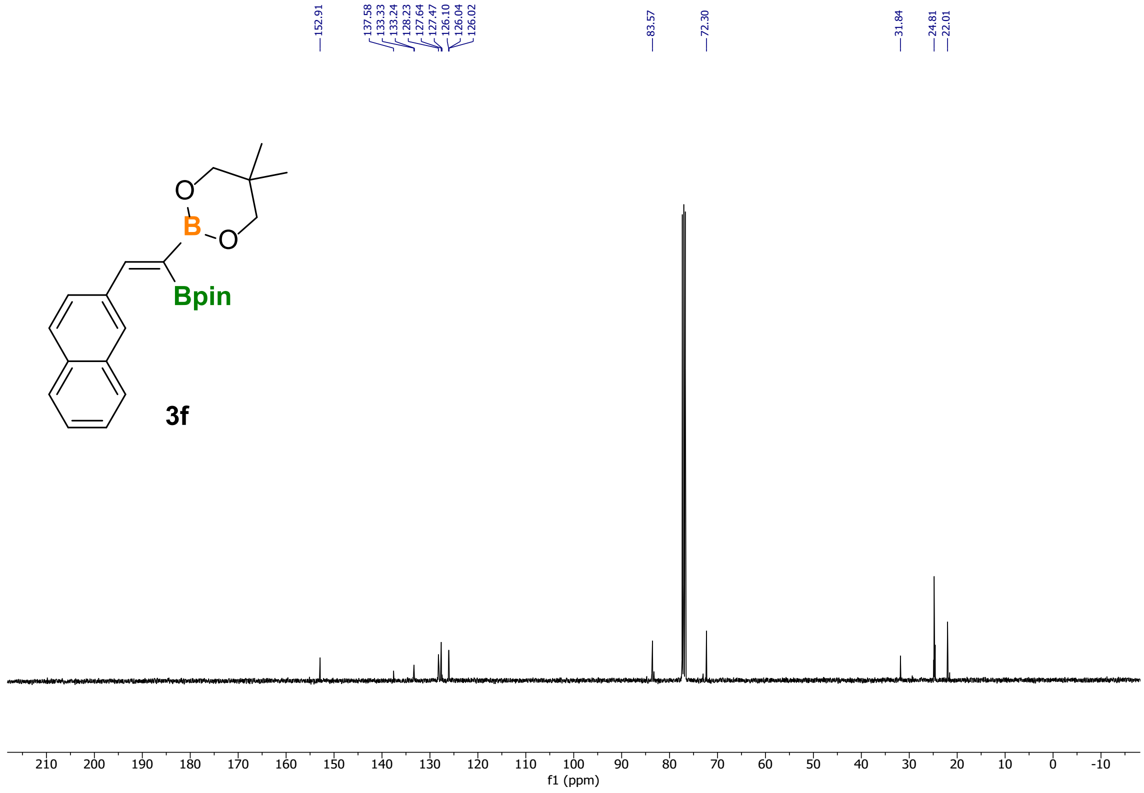

^{11}B NMR (CDCl$_3$, 128.3 MHz)

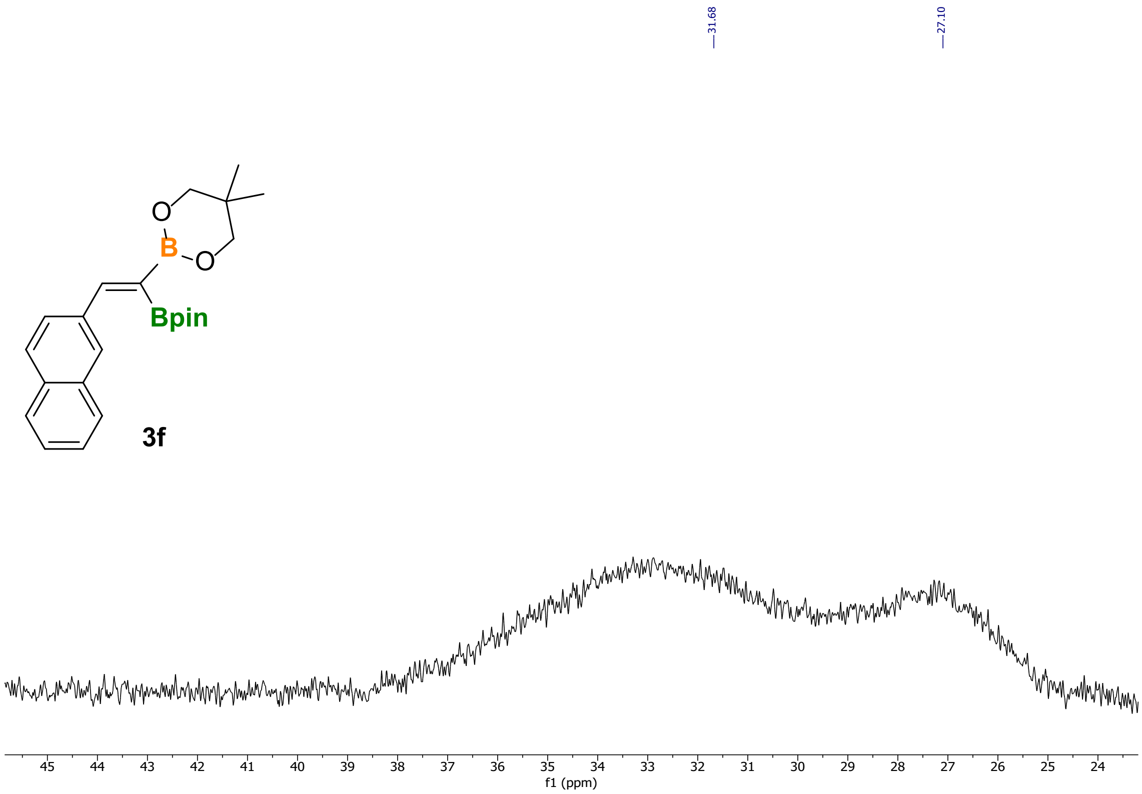

^{1}H NMR (CDCl$_3$, 400 MHz)

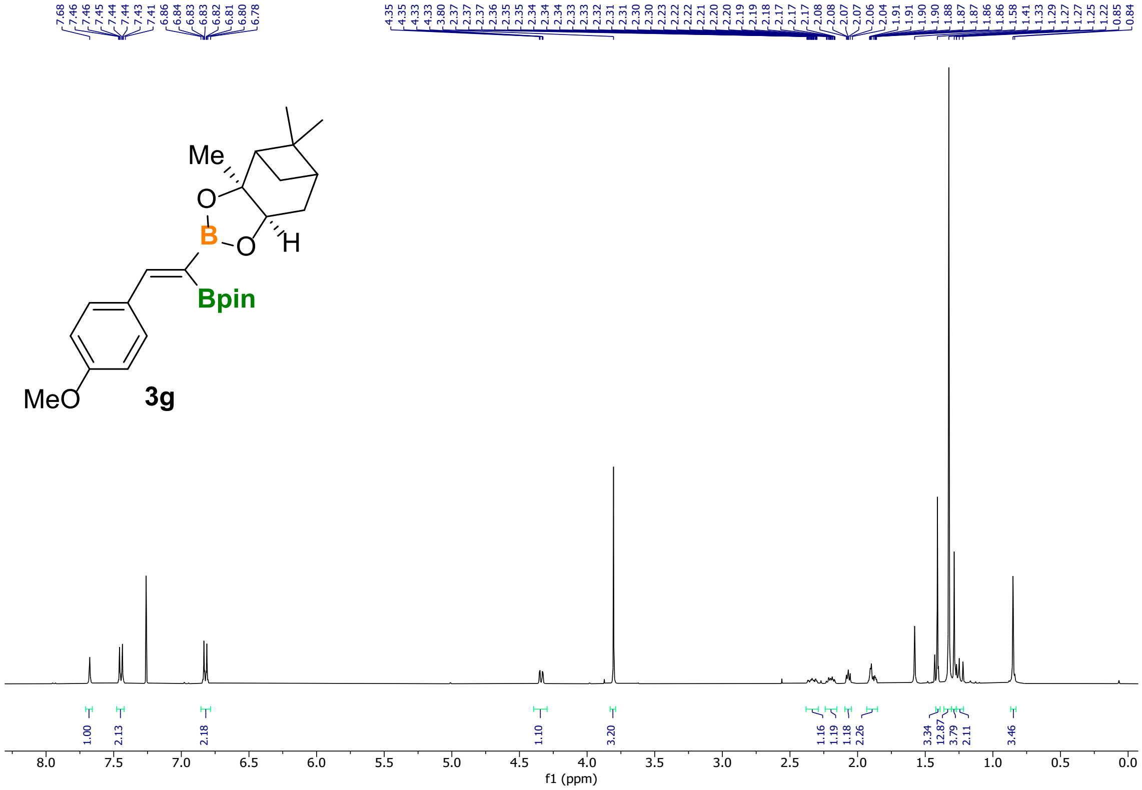

^{13}C NMR (CDCl$_3$, 100 MHz)

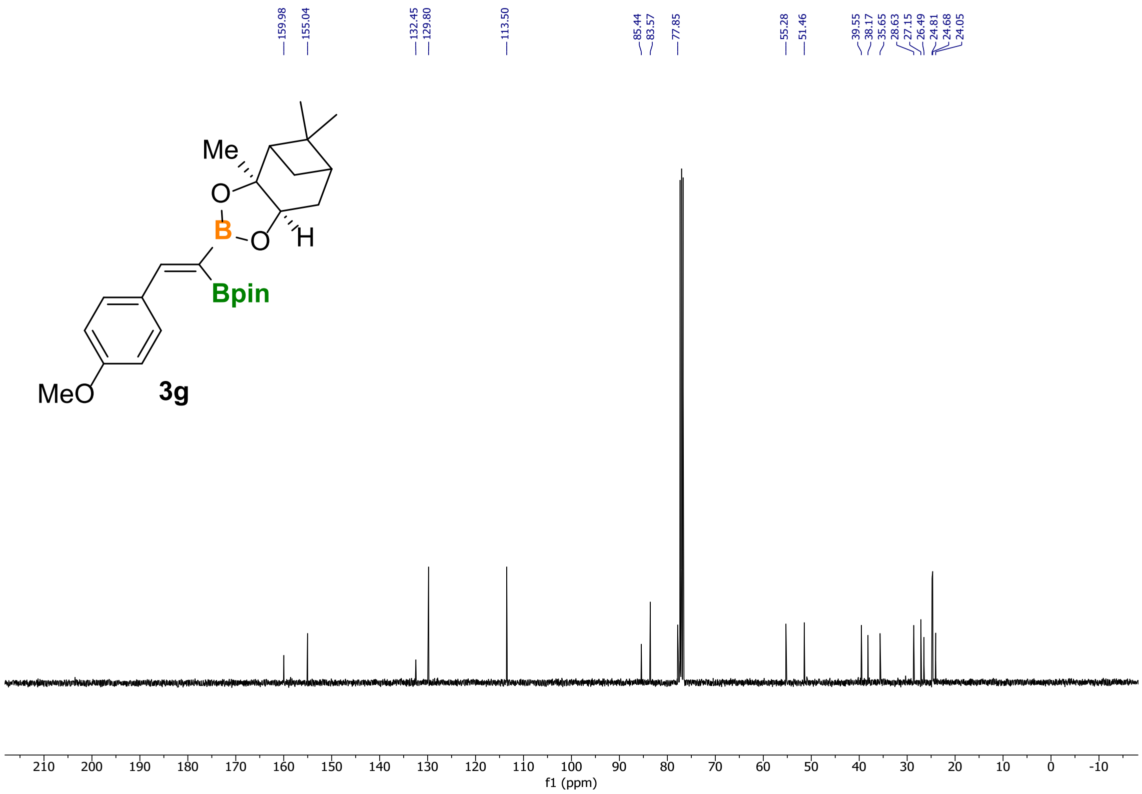

^{11}B NMR (CDCl$_3$, 128.3 MHz)

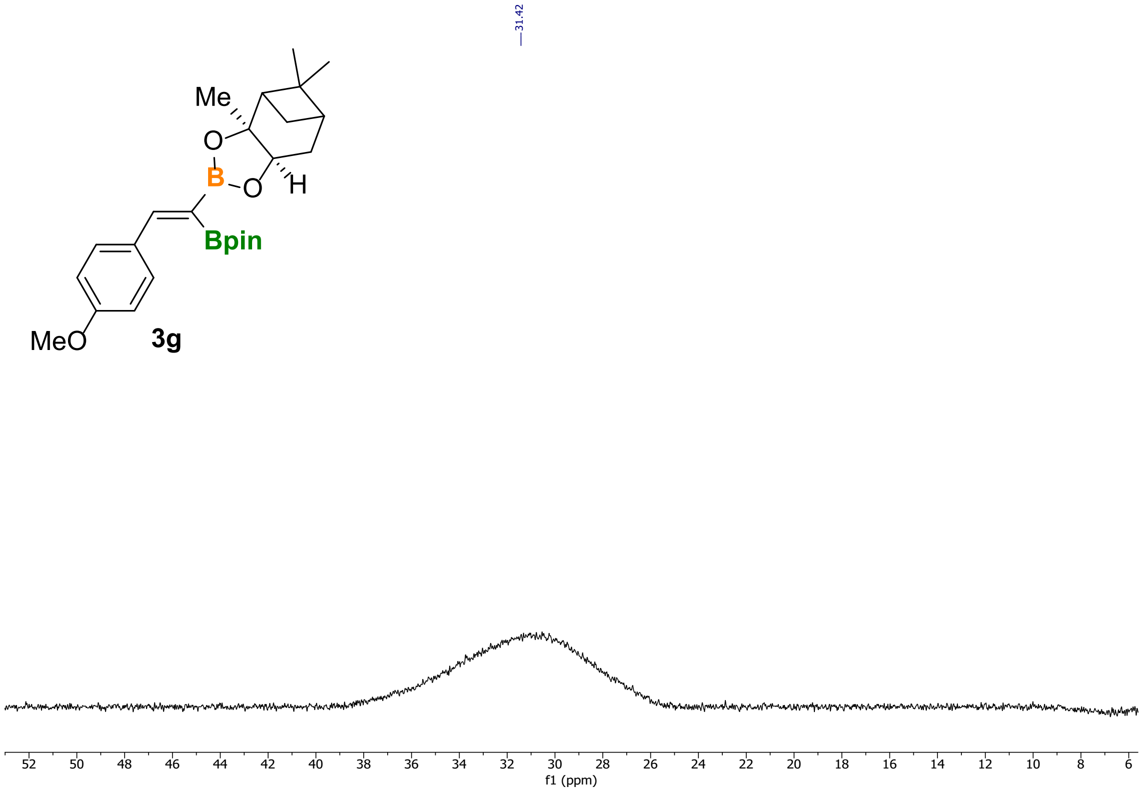

^{1}H NMR (CDCl$_3$, 400 MHz)

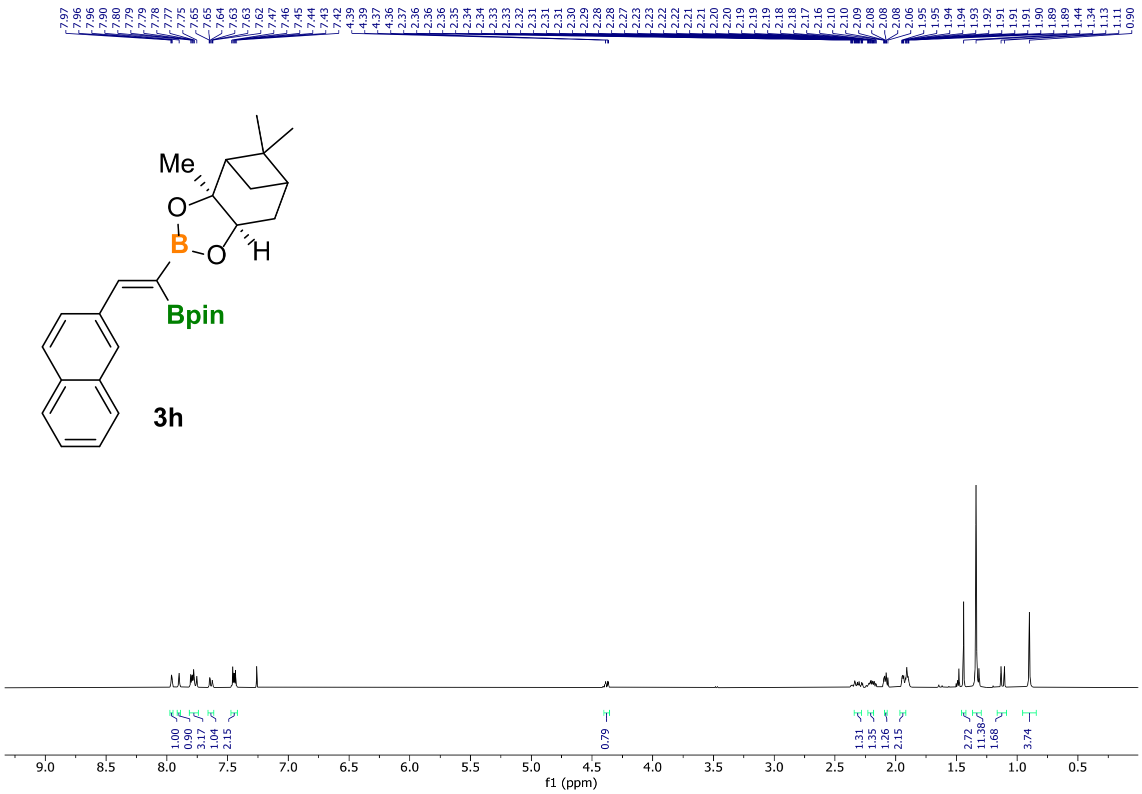

3h

^{13}C NMR ($CDCl_3$, 100 MHz)

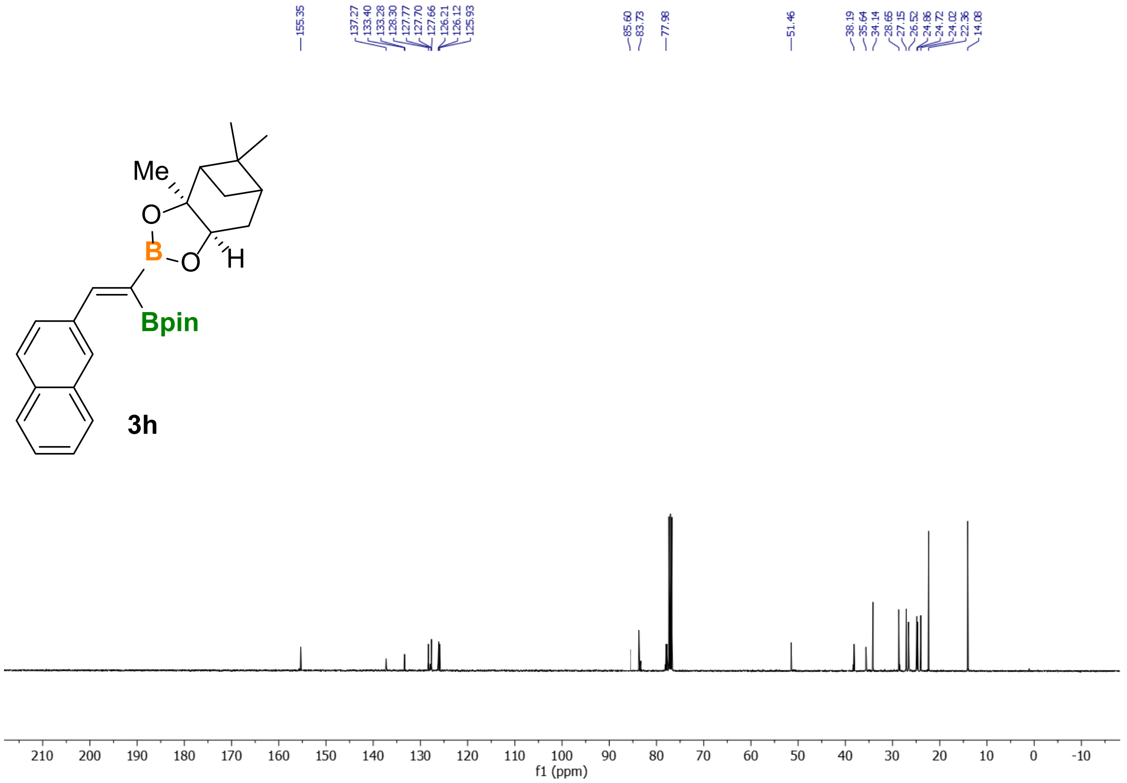

3h

^{11}B NMR (CDCl$_3$, 128.3 MHz)

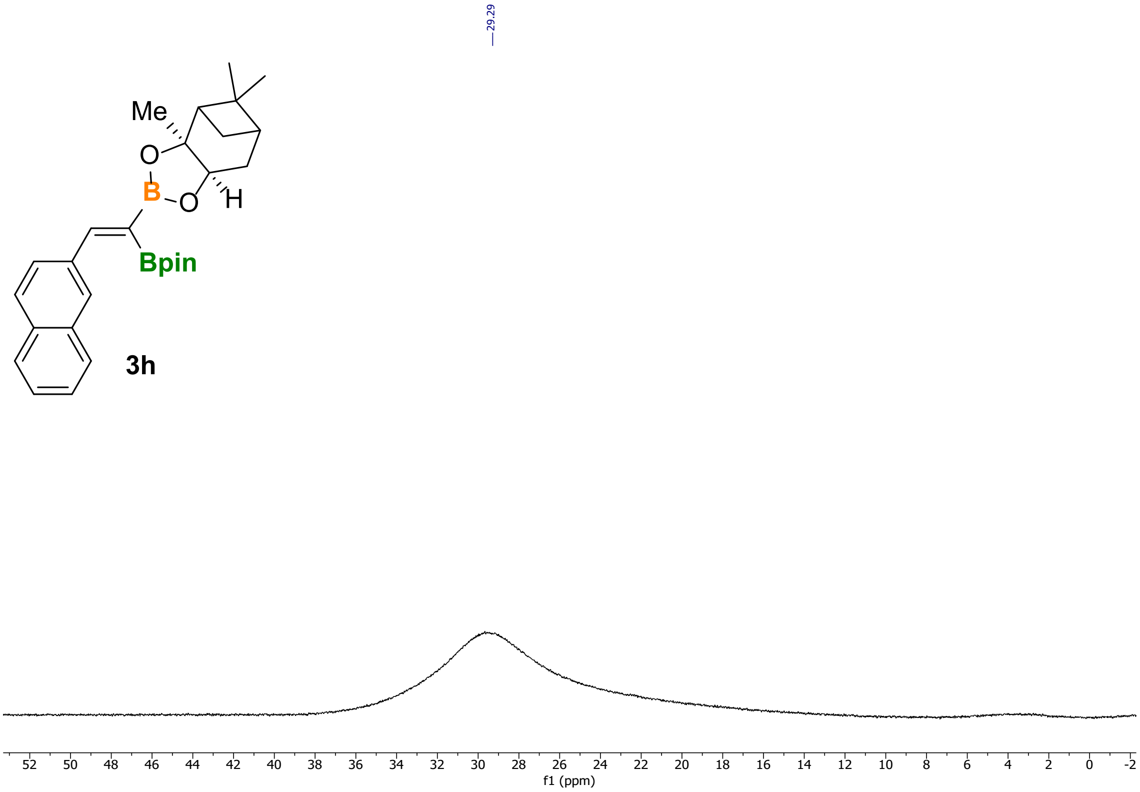

^{1}H NMR (CDCl$_3$, 400 MHz)

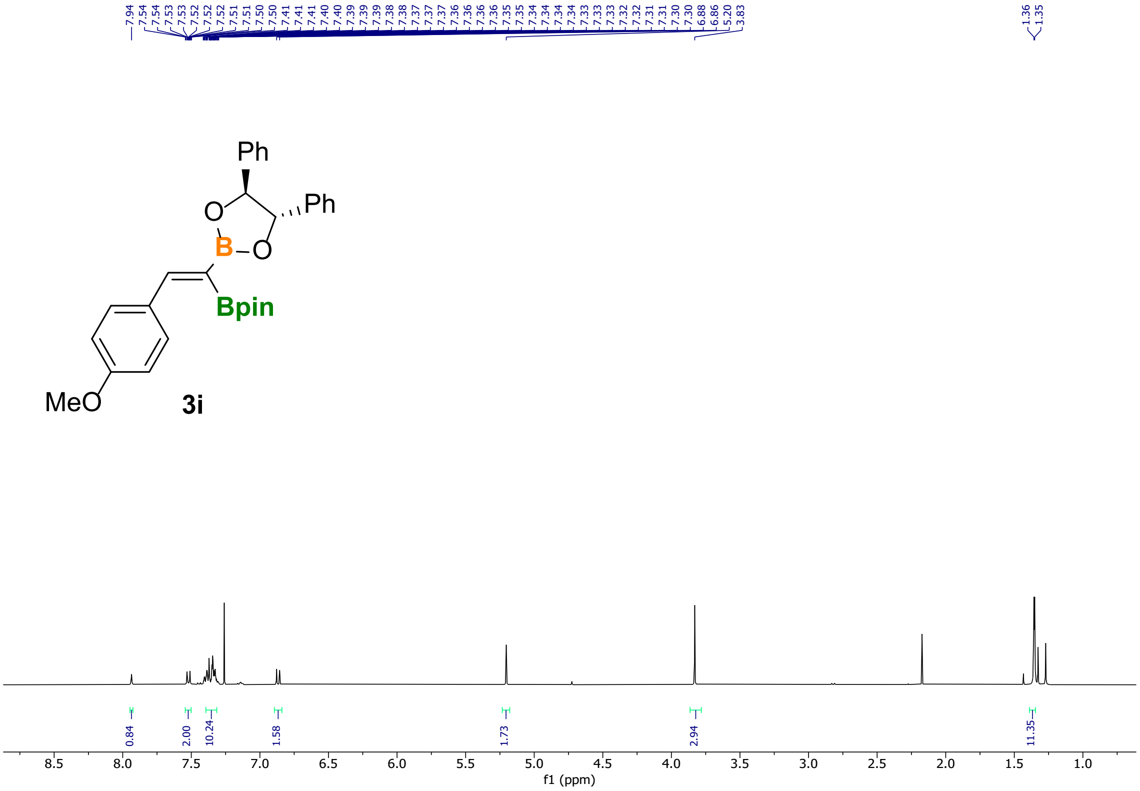

^{13}C NMR ($CDCl_3$, 100 MHz)

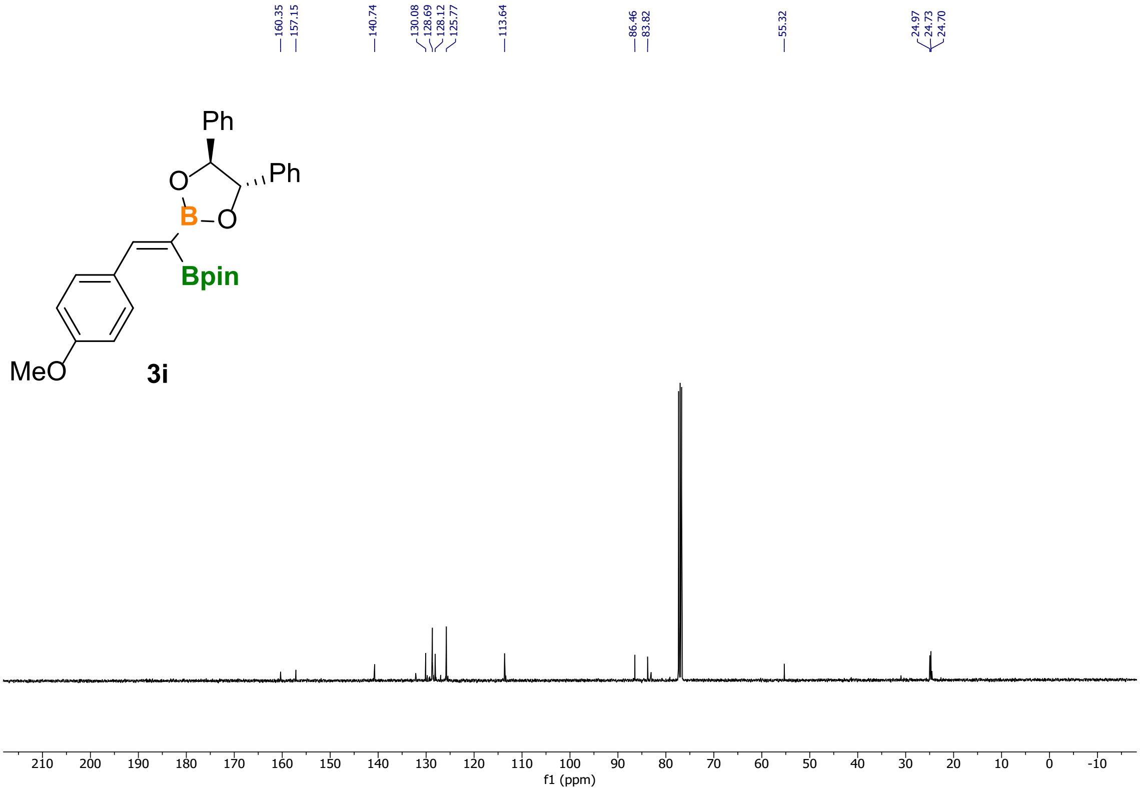

^{11}B NMR ($CDCl_3$, 128.3 MHz)

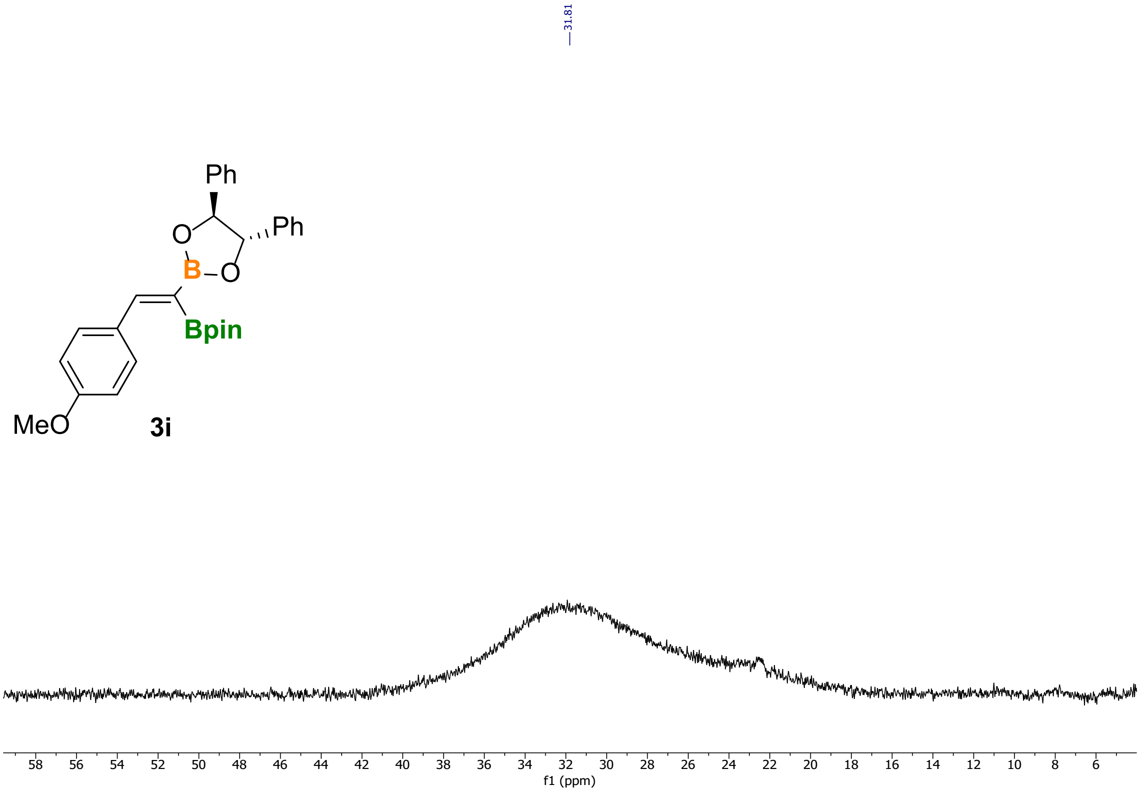

^{1}H NMR ($CDCl_3$, 400 MHz)

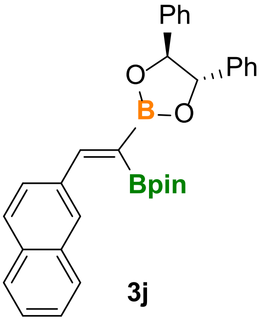

3j

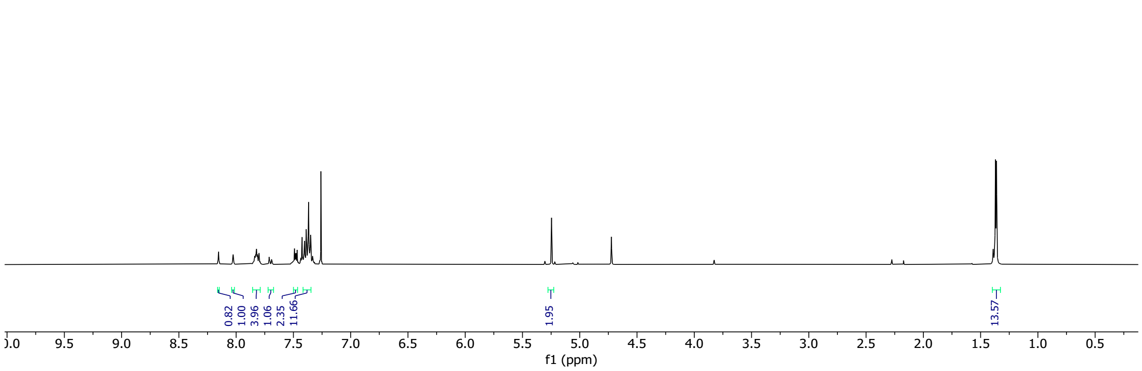

^{13}C NMR ($CDCl_3$, 100 MHz)

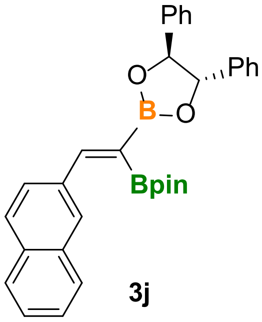

3j

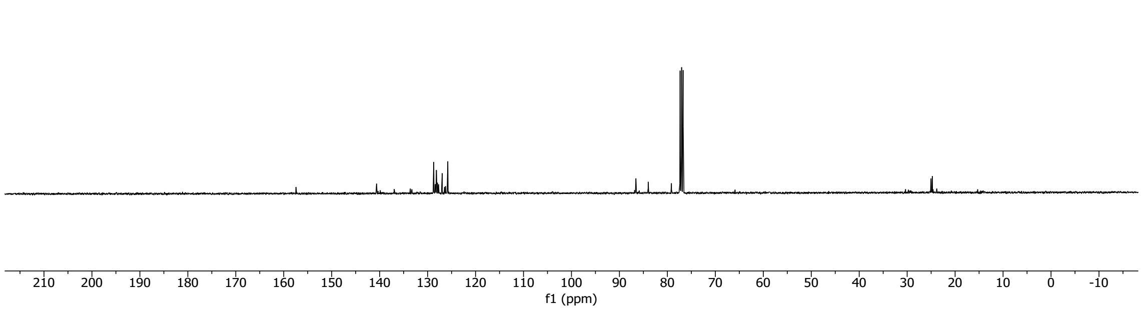

^{11}B NMR (CDCl$_3$, 128.3 MHz)

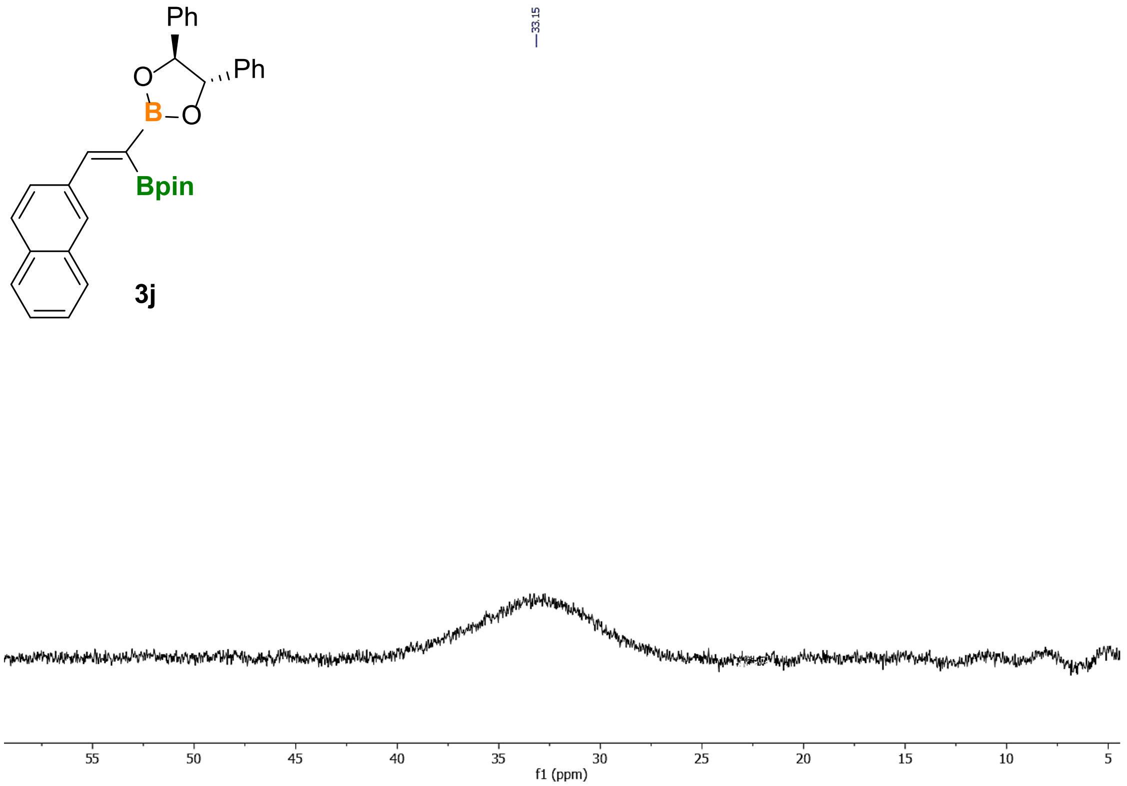

^{1}H NMR (CDCl$_3$, 400 MHz)

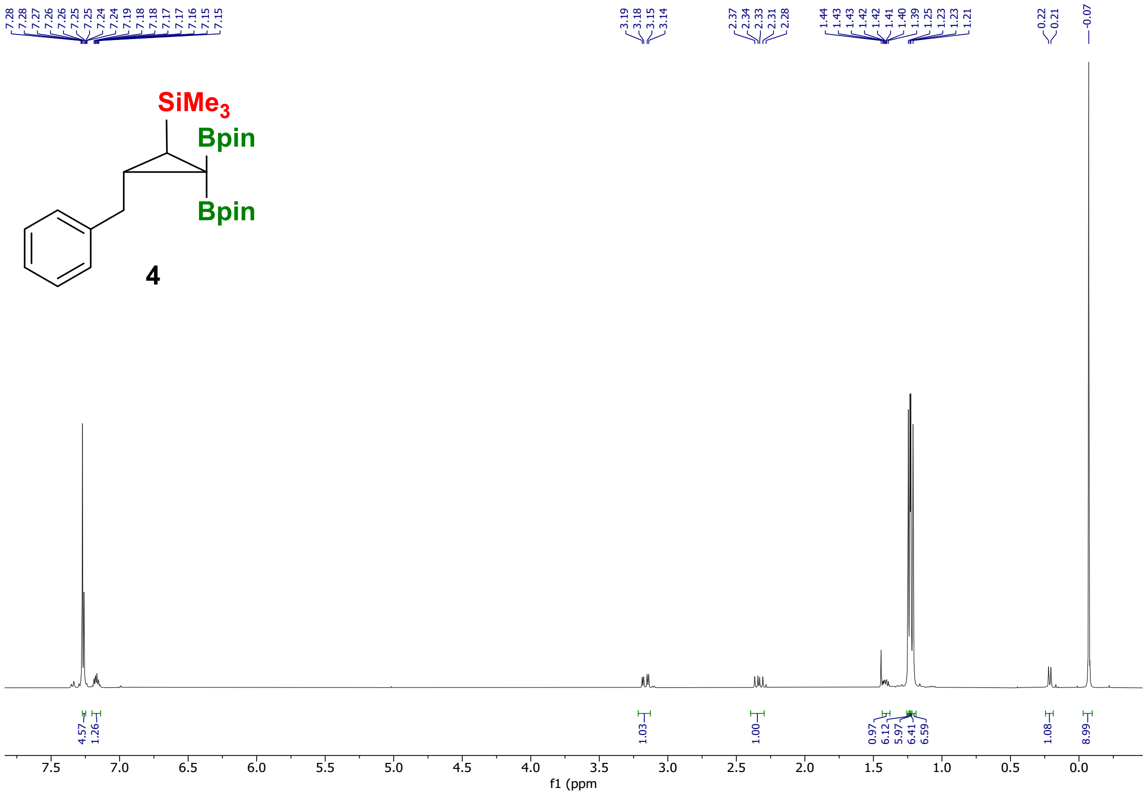

^{13}C NMR ($CDCl_3$, 100 MHz)

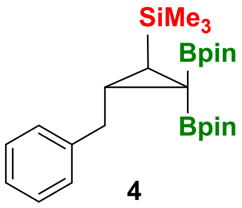

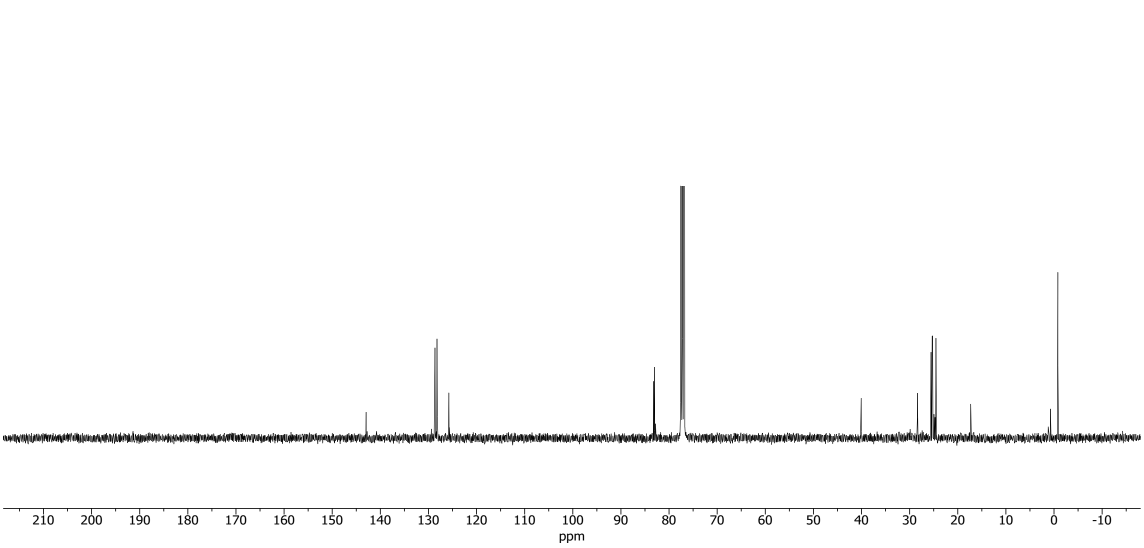

^{11}B NMR ($CDCl_3$, 128.3 MHz)

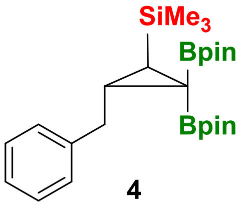

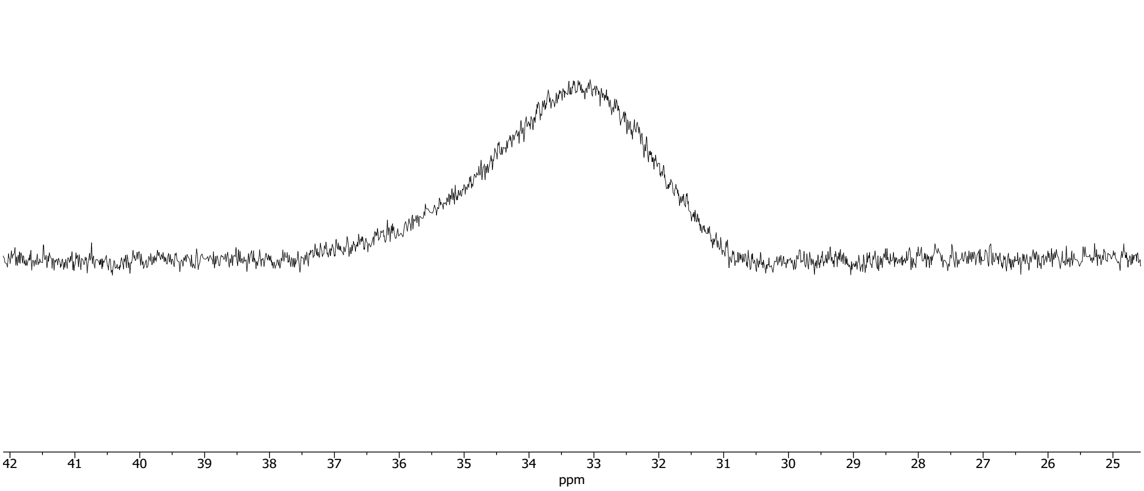

^1H NMR ($CDCl_3$, 400 MHz)

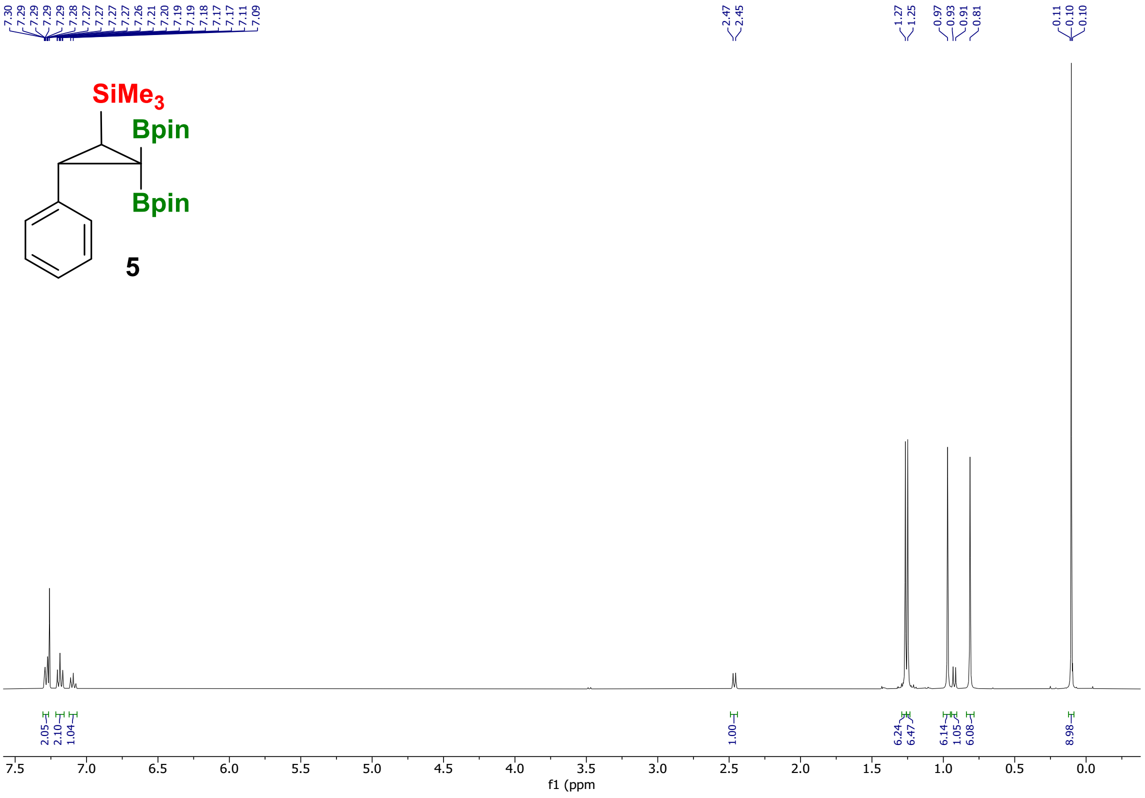

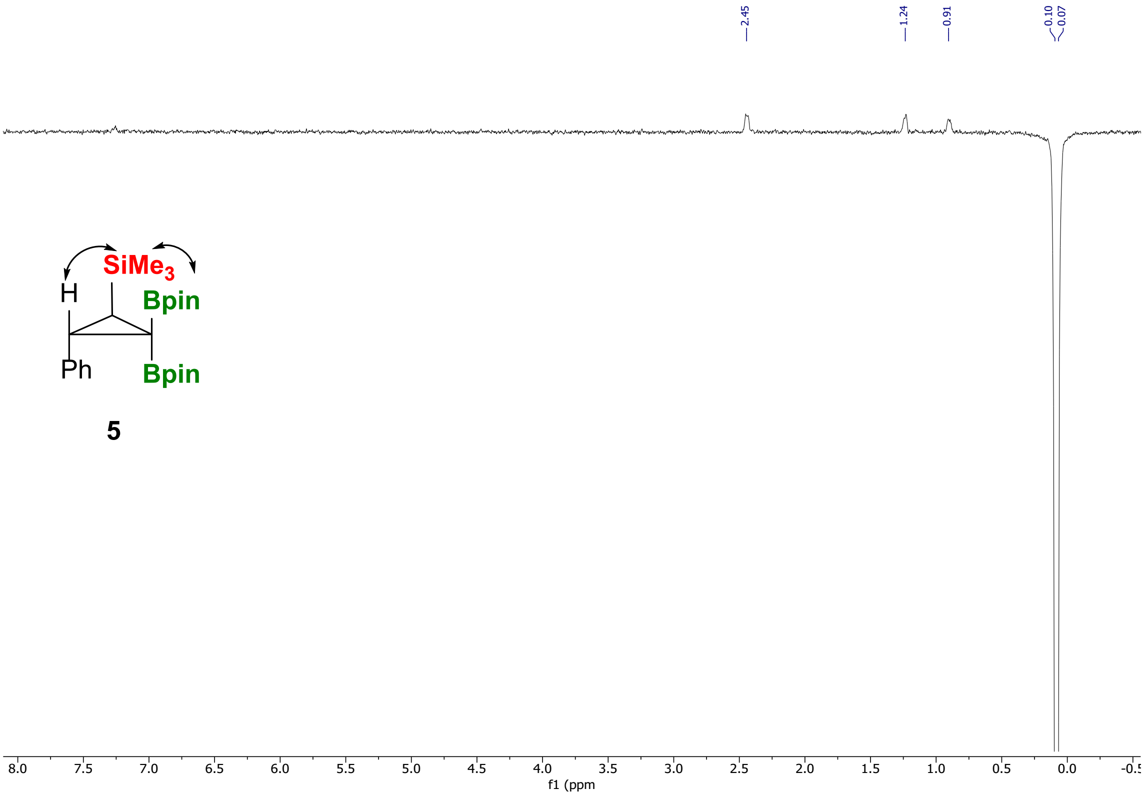

^{13}C NMR (CDCl$_3$, 100 MHz)

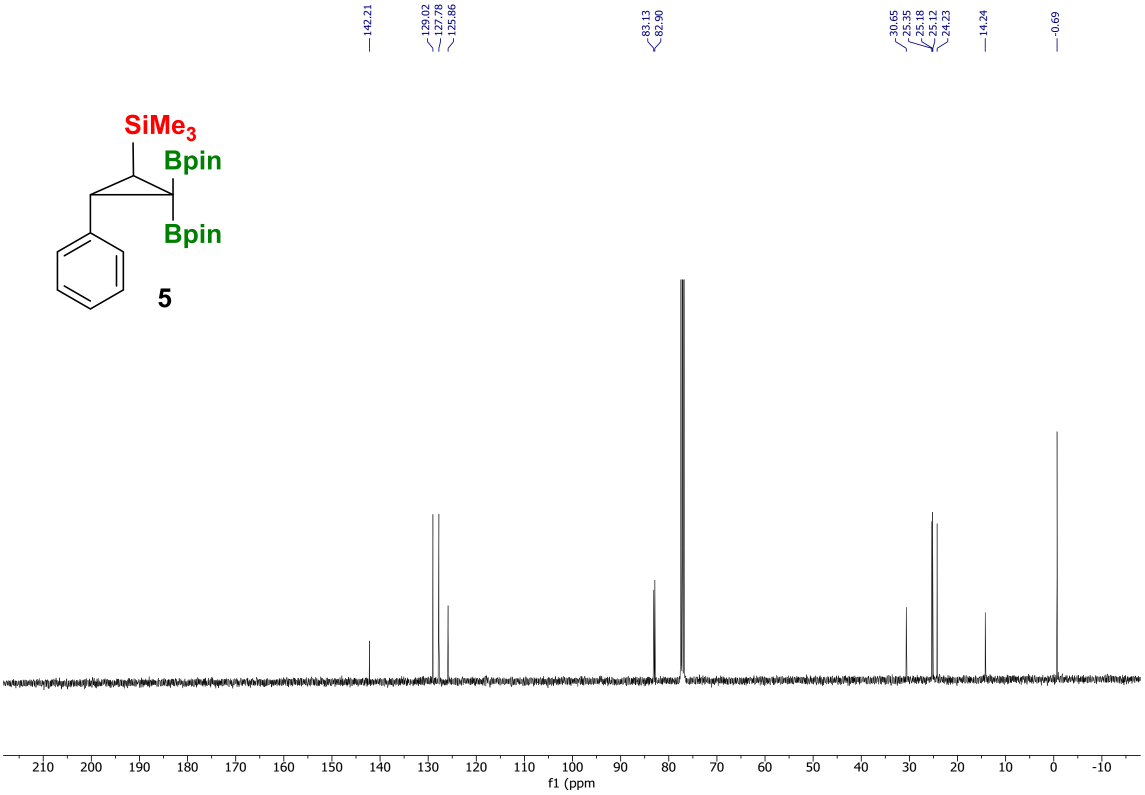

^{11}B NMR (CDCl$_3$, 128.3 MHz)

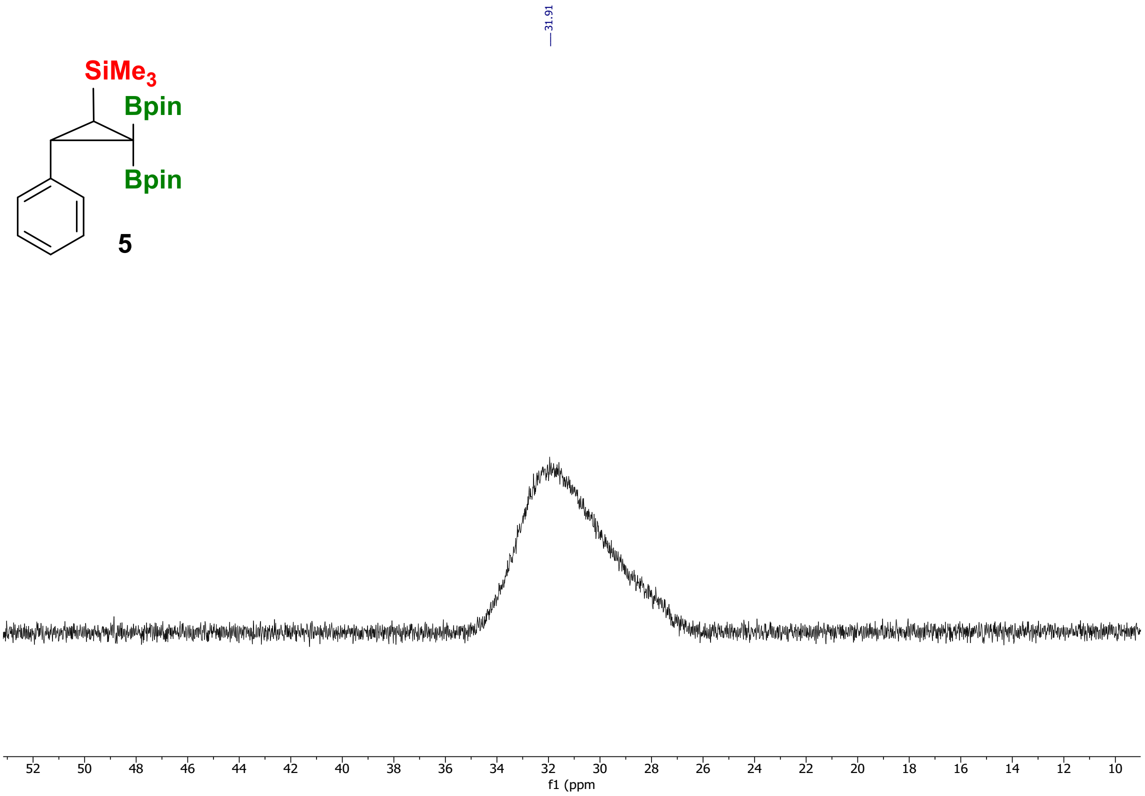

^{1}H NMR ($CDCl_3$, 400 MHz)

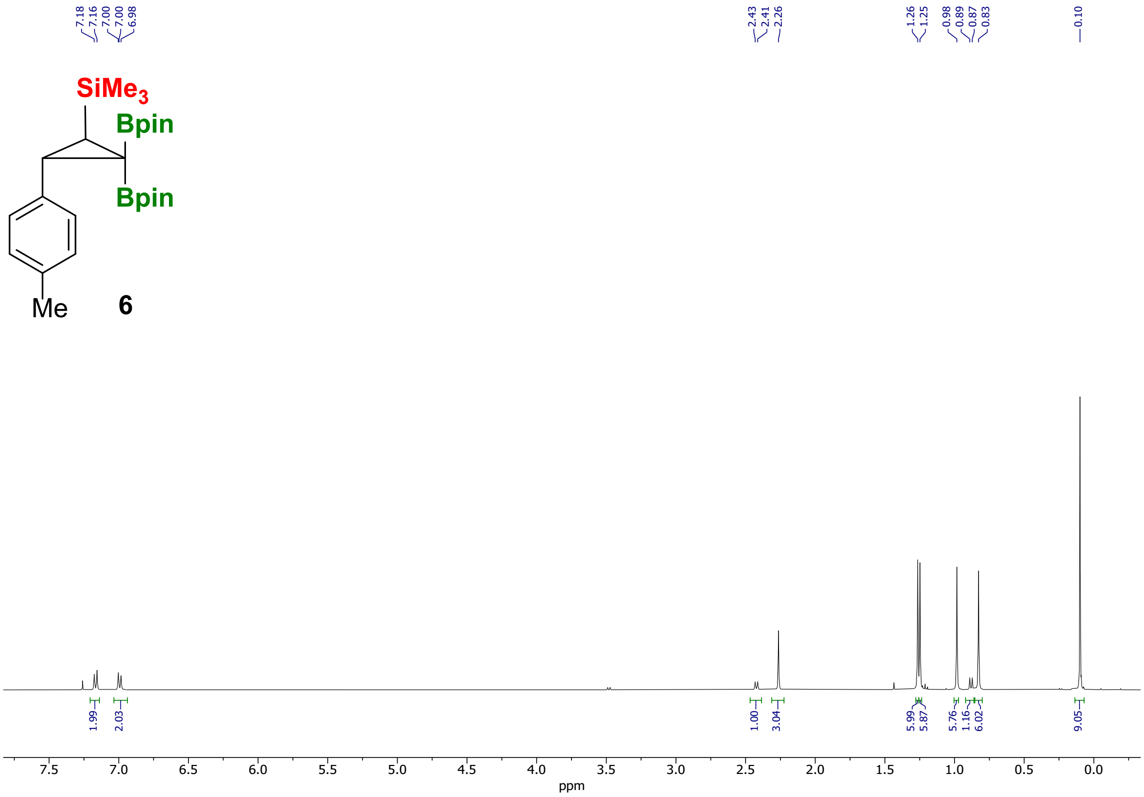

^{13}C NMR ($CDCl_3$, 100 MHz)

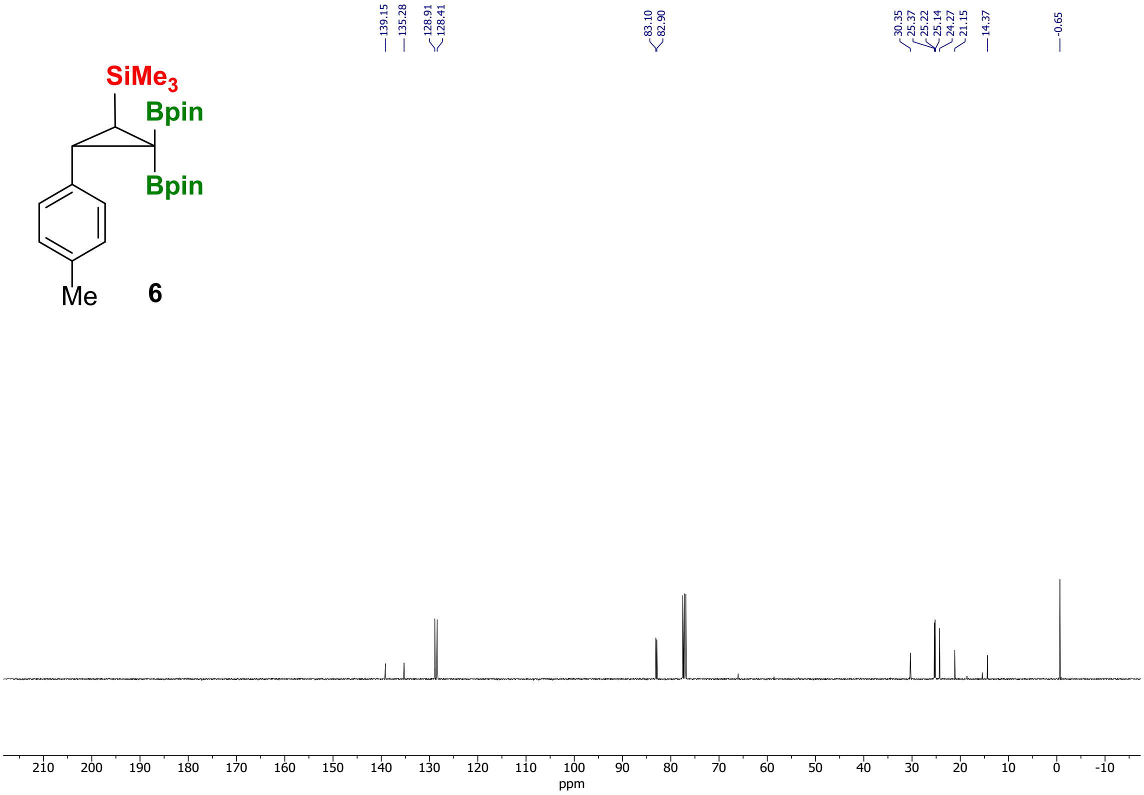

^{11}B NMR ($CDCl_3$, 128.3 MHz)

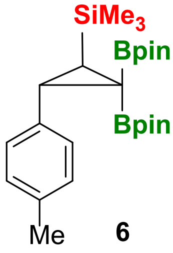

6

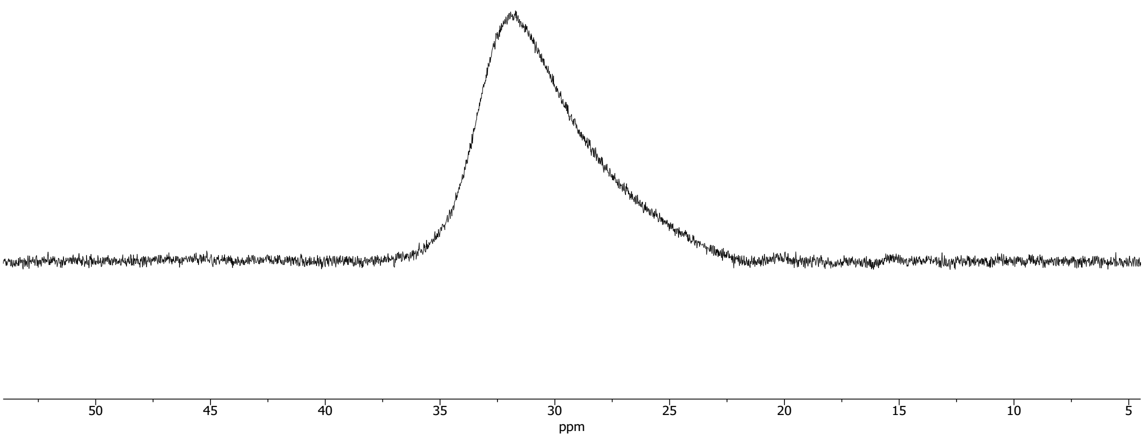

^{1}H NMR ($CDCl_3$, 400 MHz)

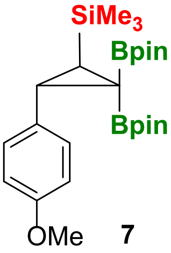

7

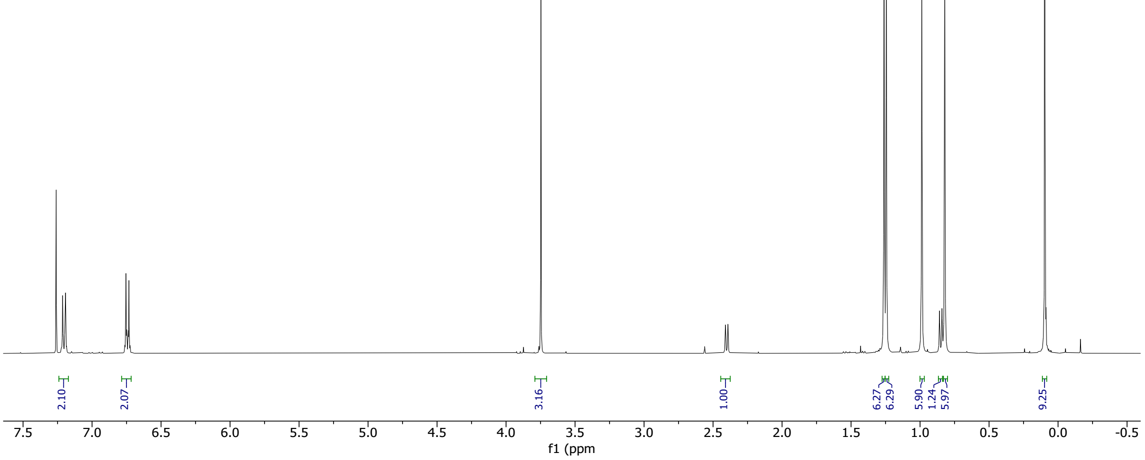

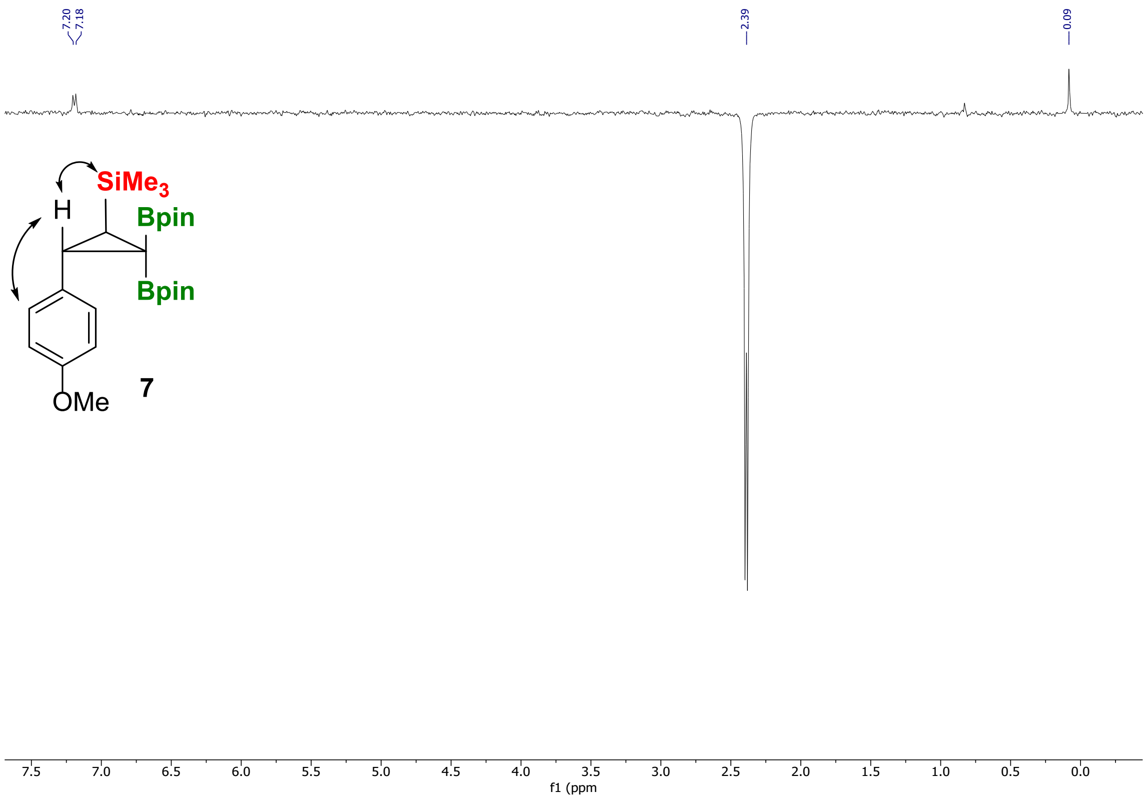

13C NMR (CDCl$_3$, 100 MHz)

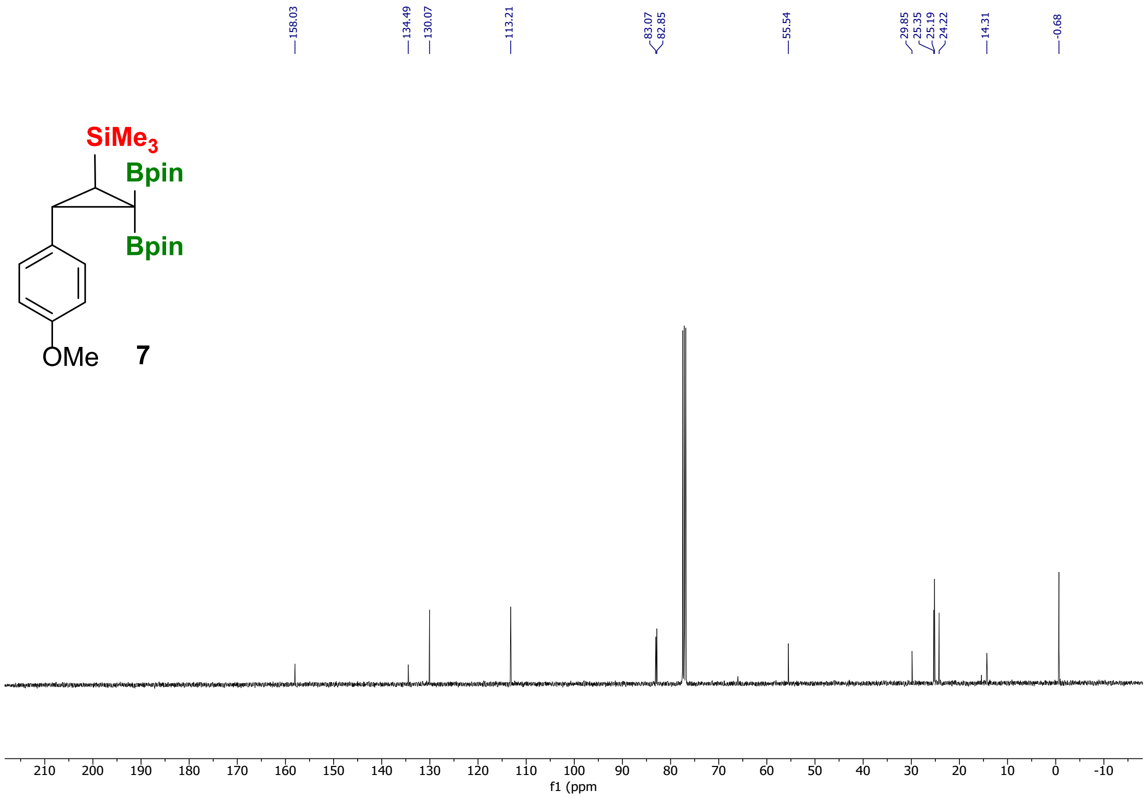

^{11}B NMR (CDCl$_3$, 128.3 MHz)

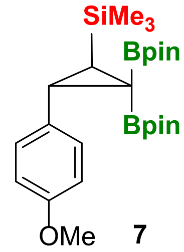

7

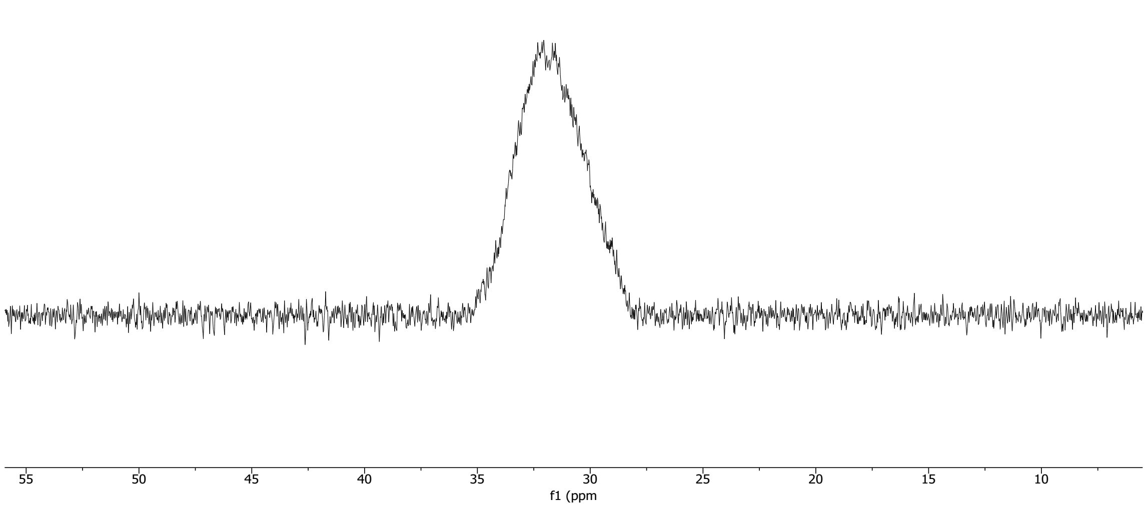

^{1}H NMR (CDCl$_3$, 400 MHz)

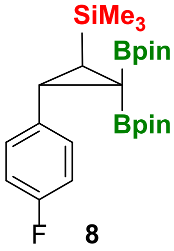

8

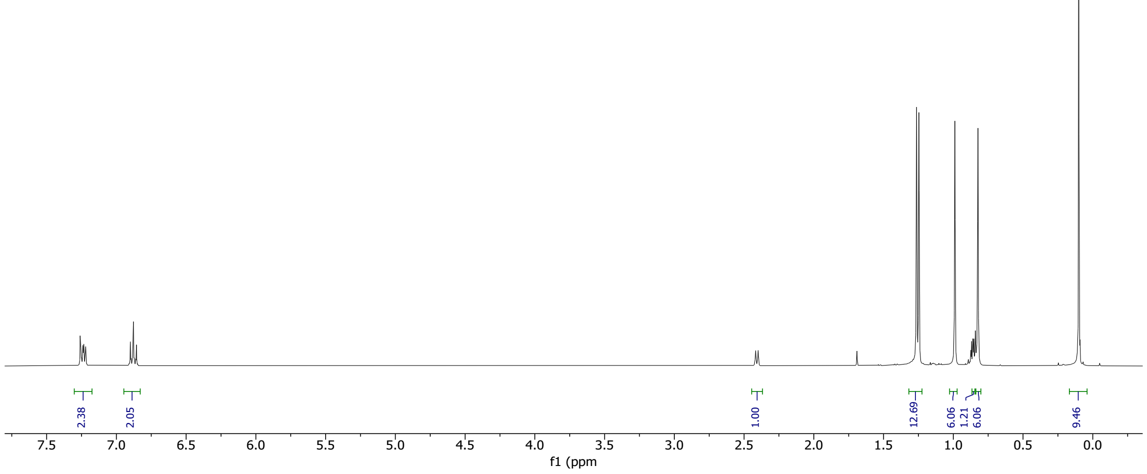

^{13}C NMR ($CDCl_3$, 100 MHz)

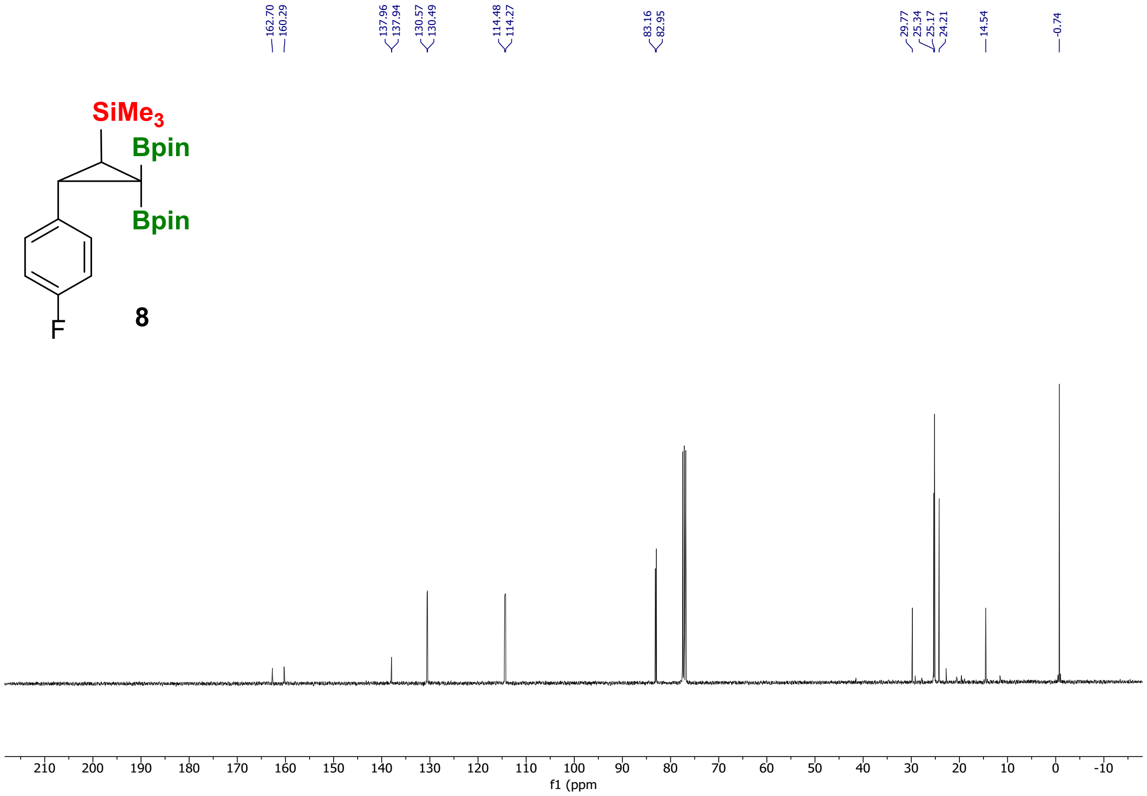

8

^{11}B NMR ($CDCl_3$, 128.3 MHz)

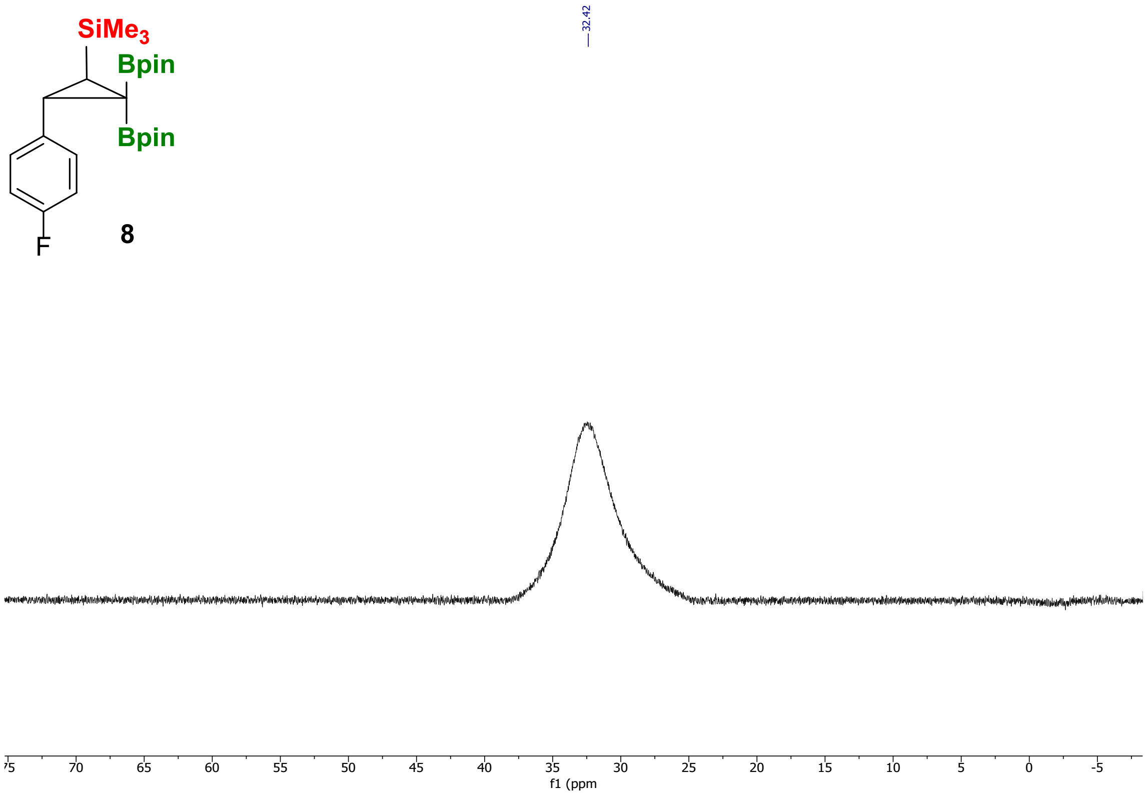

8

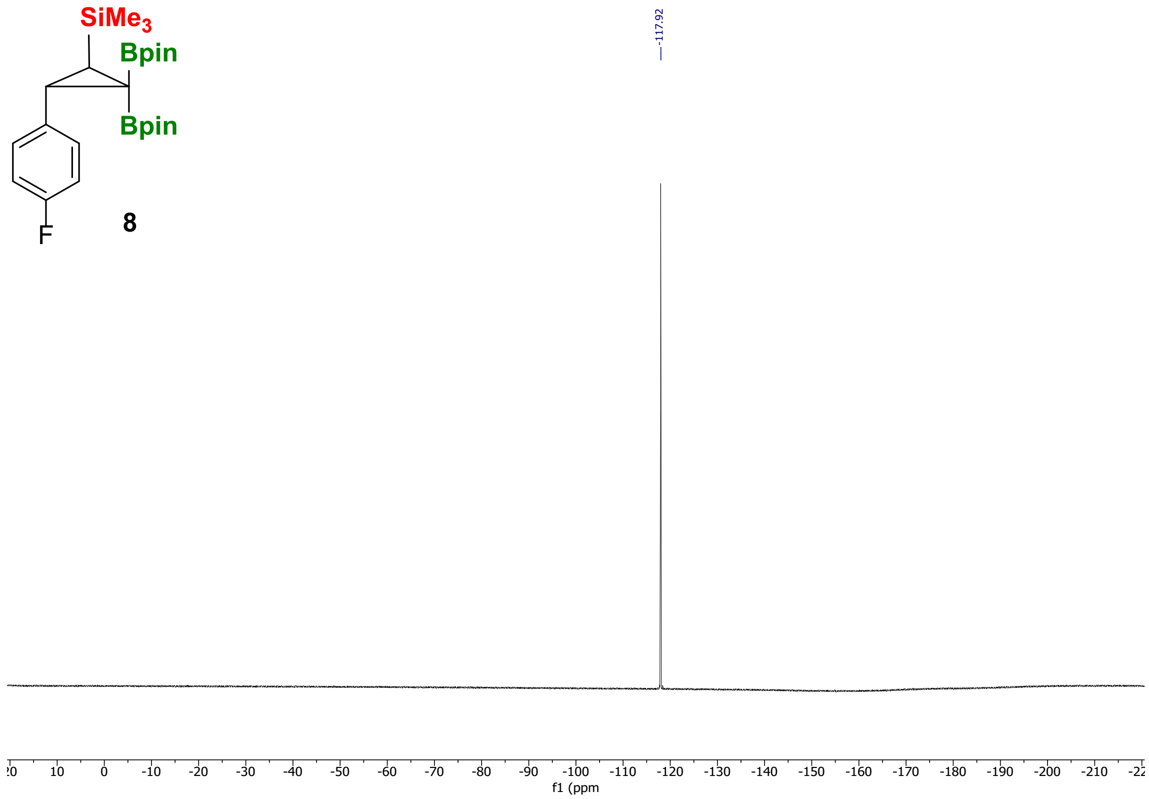

^{1}H NMR ($CDCl_3$, 400 MHz)

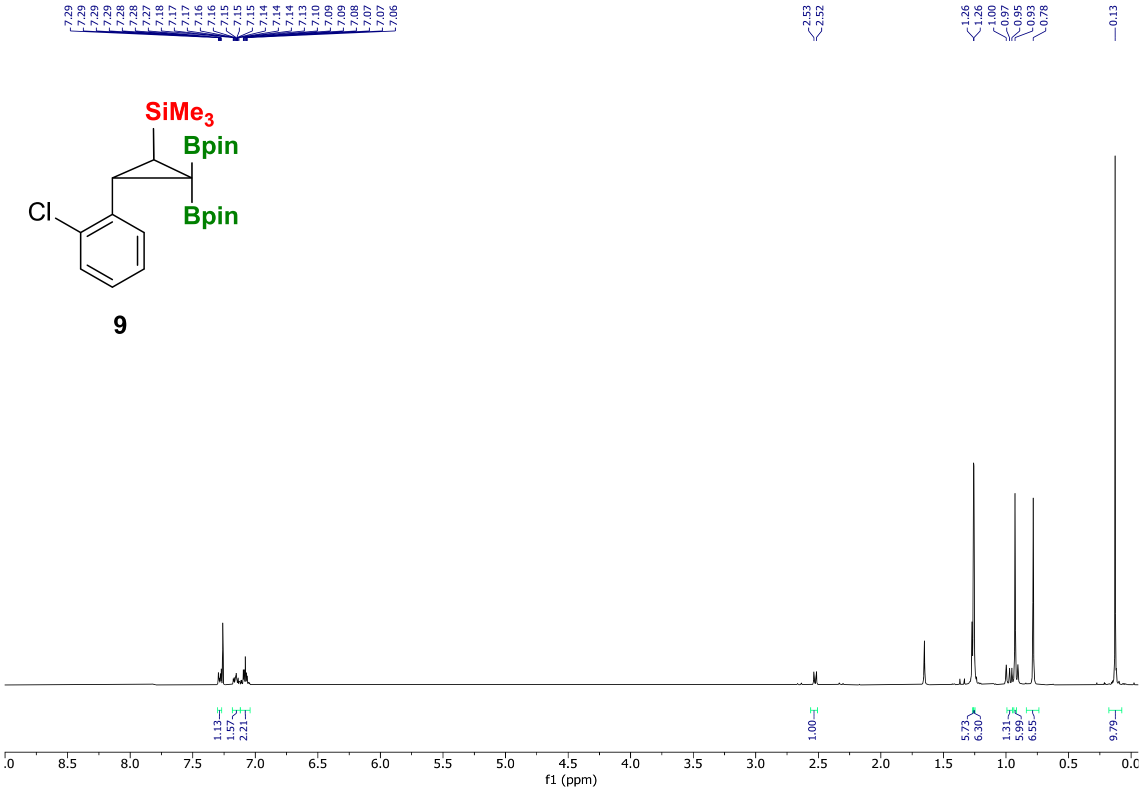

^{13}C NMR (CDCl$_3$, 100 MHz)

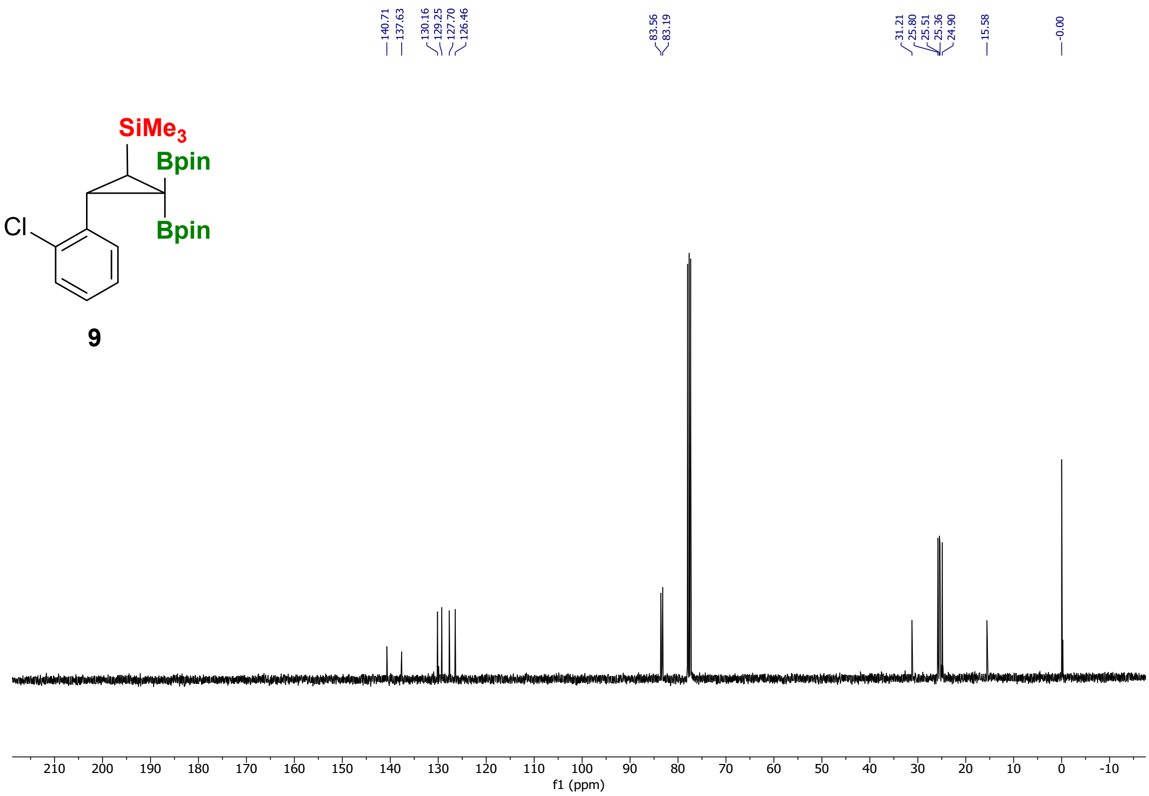

^{11}B NMR (CDCl$_3$, 128.3 MHz)

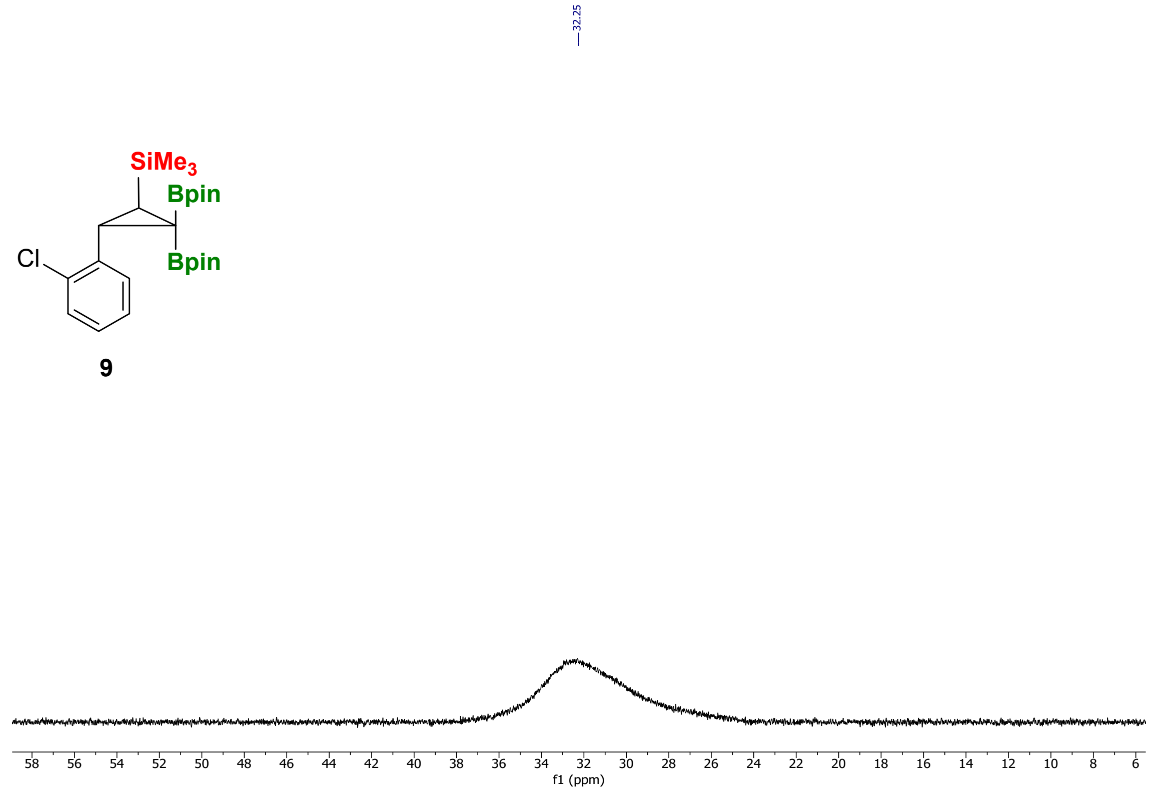

^{1}H NMR ($CDCl_3$, 400 MHz)

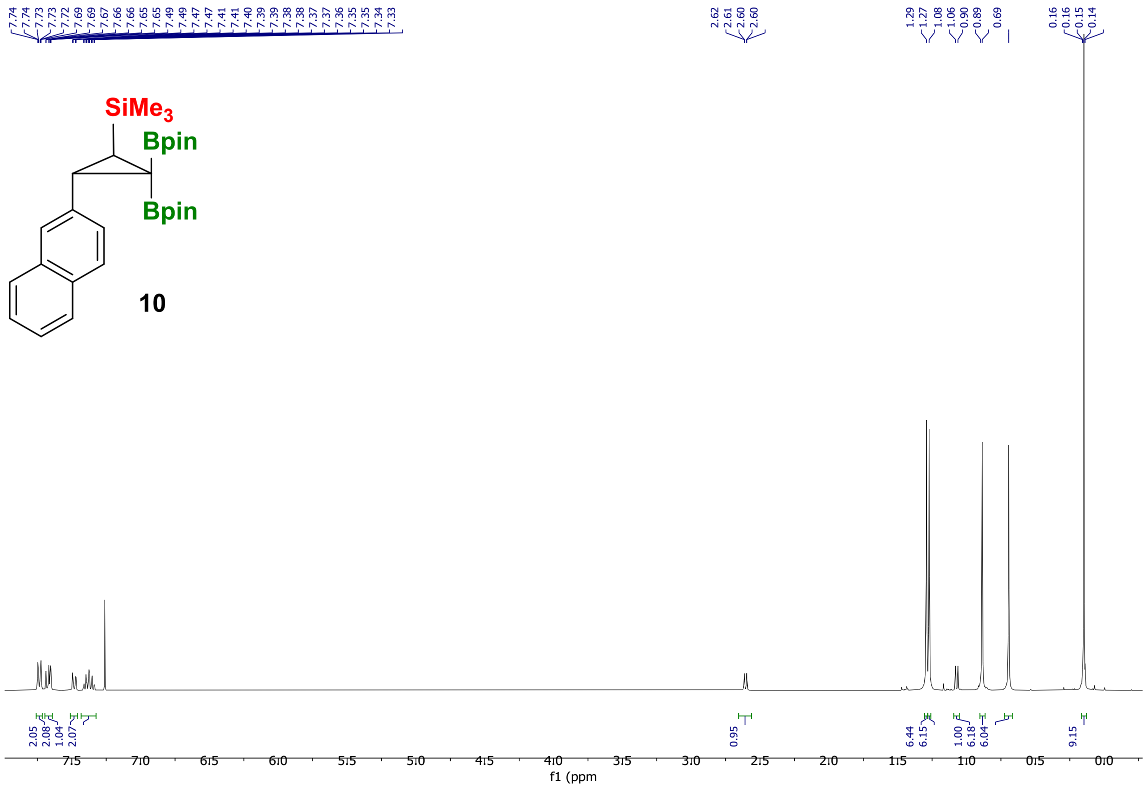

^{13}C NMR ($CDCl_3$, 100 MHz)

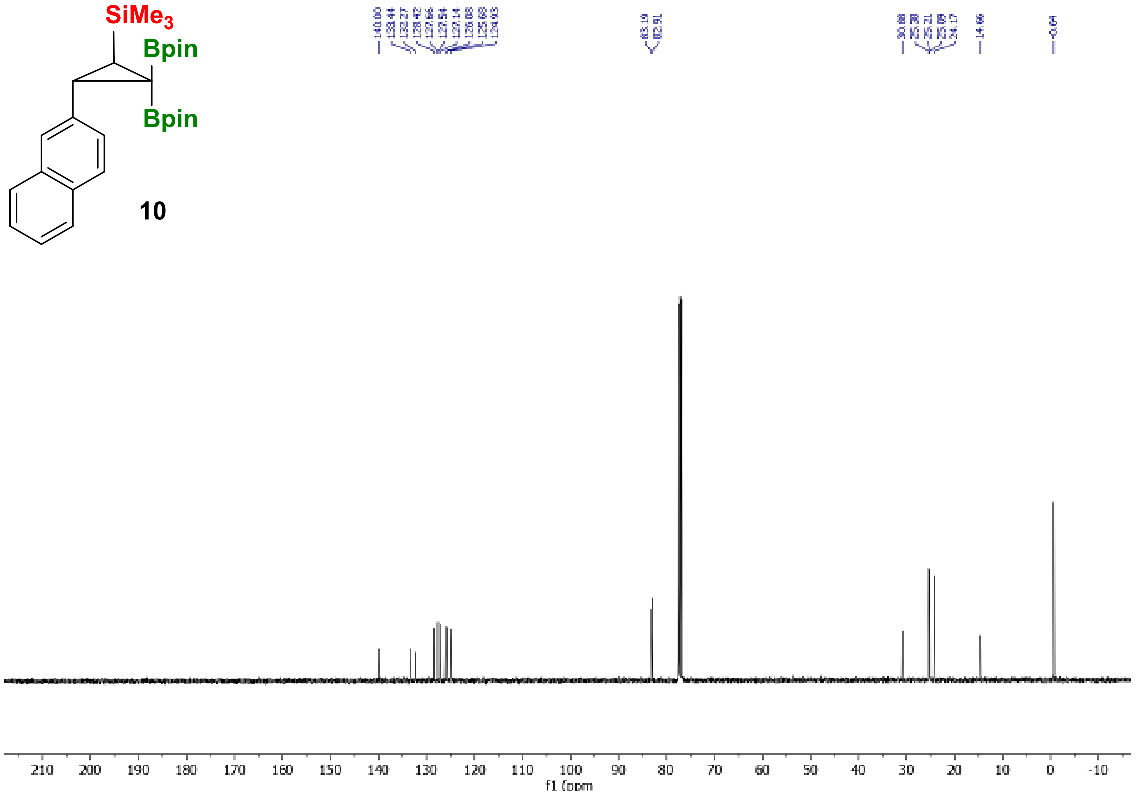

^{11}B NMR (CDCl$_3$, 128.3 MHz)

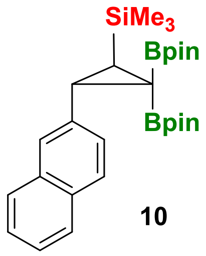

10

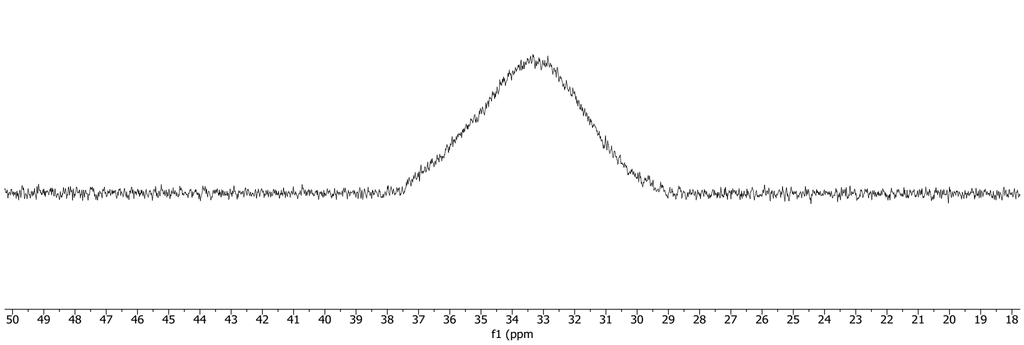

^{1}H NMR (CDCl$_3$, 400 MHz)

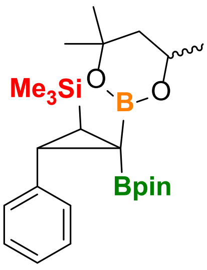

11a

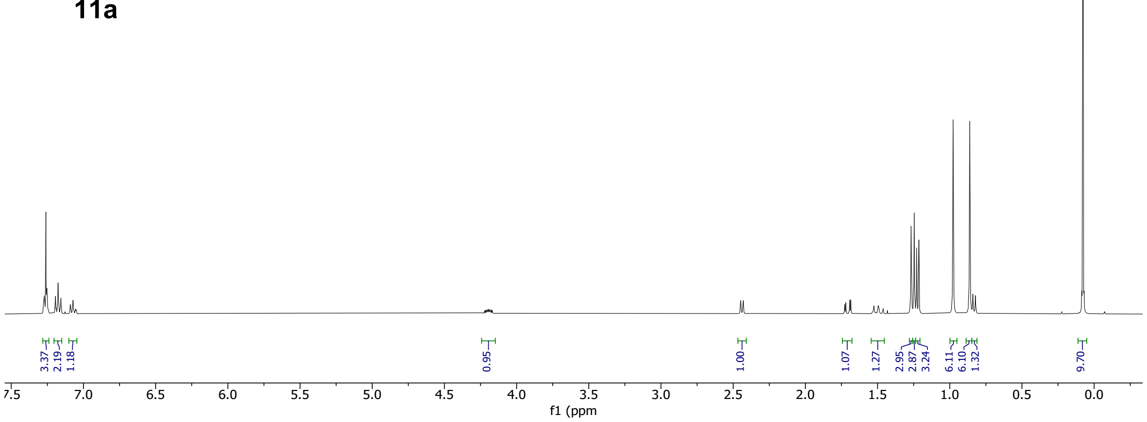

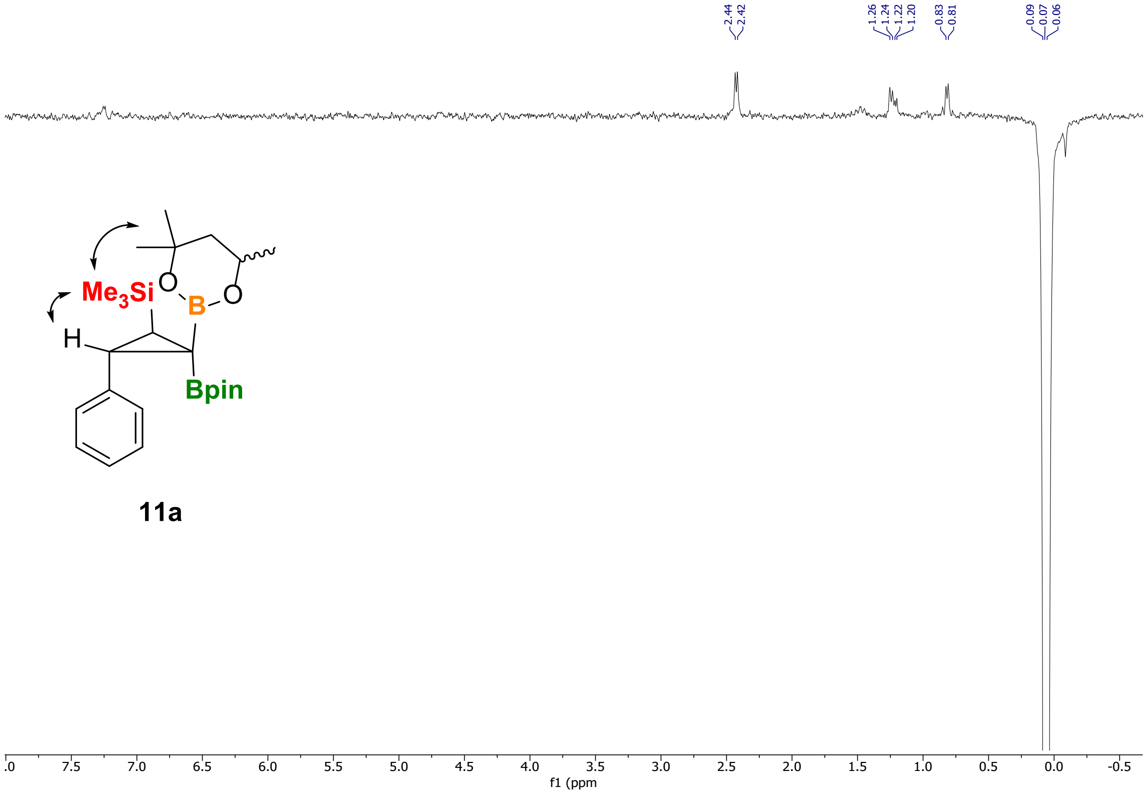

^{13}C NMR ($CDCl_3$, 100 MHz)

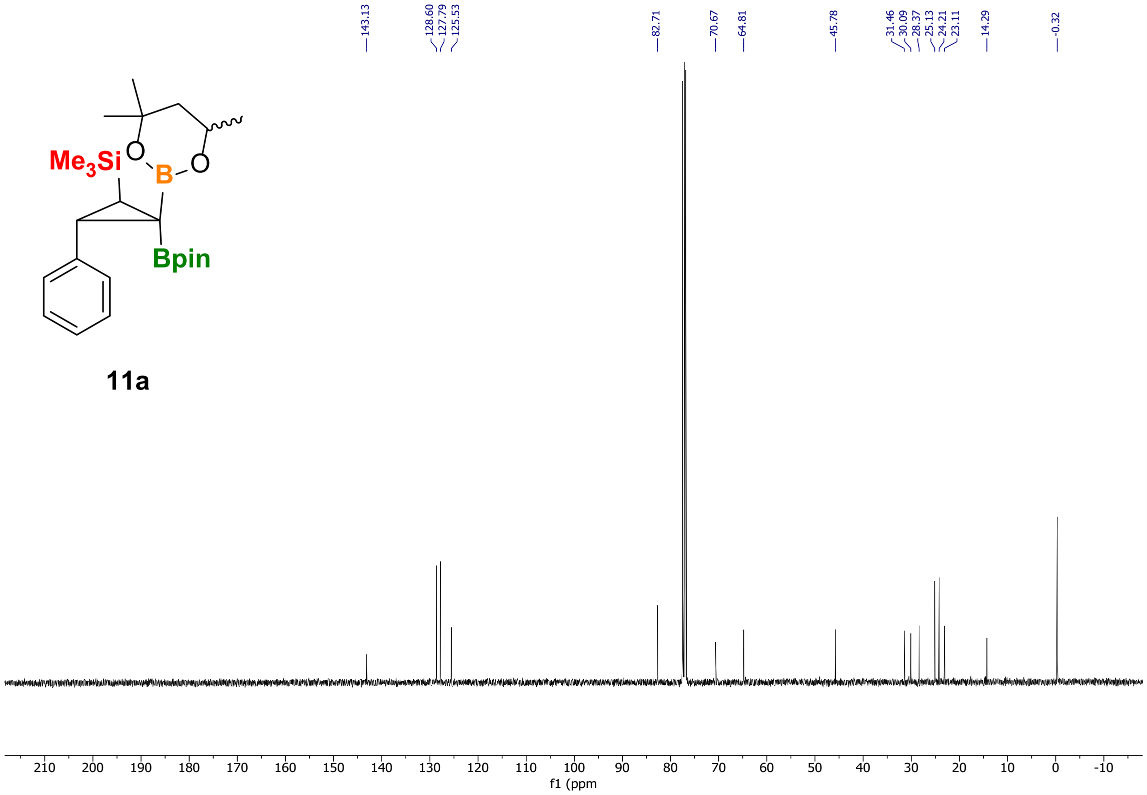

^{11}B NMR ($CDCl_3$, 128.3 MHz)

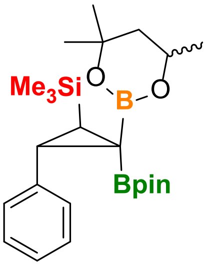

11a

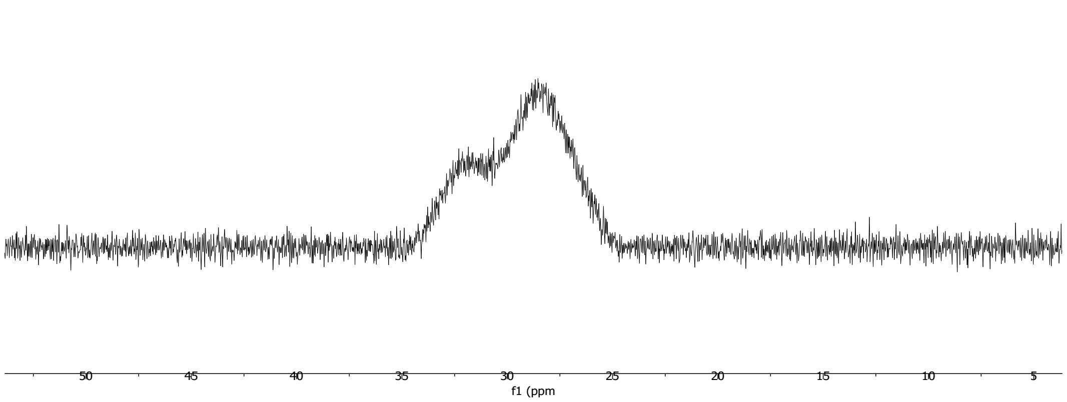

^1H NMR ($CDCl_3$, 400 MHz)

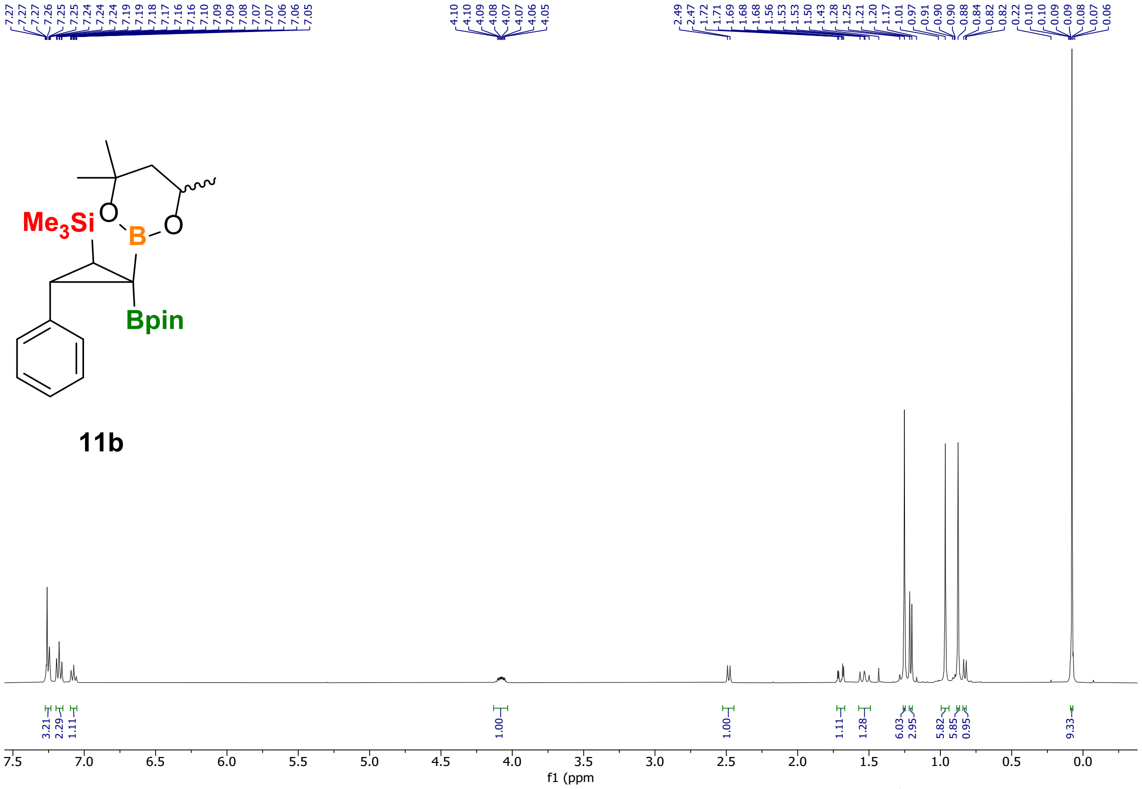

11b

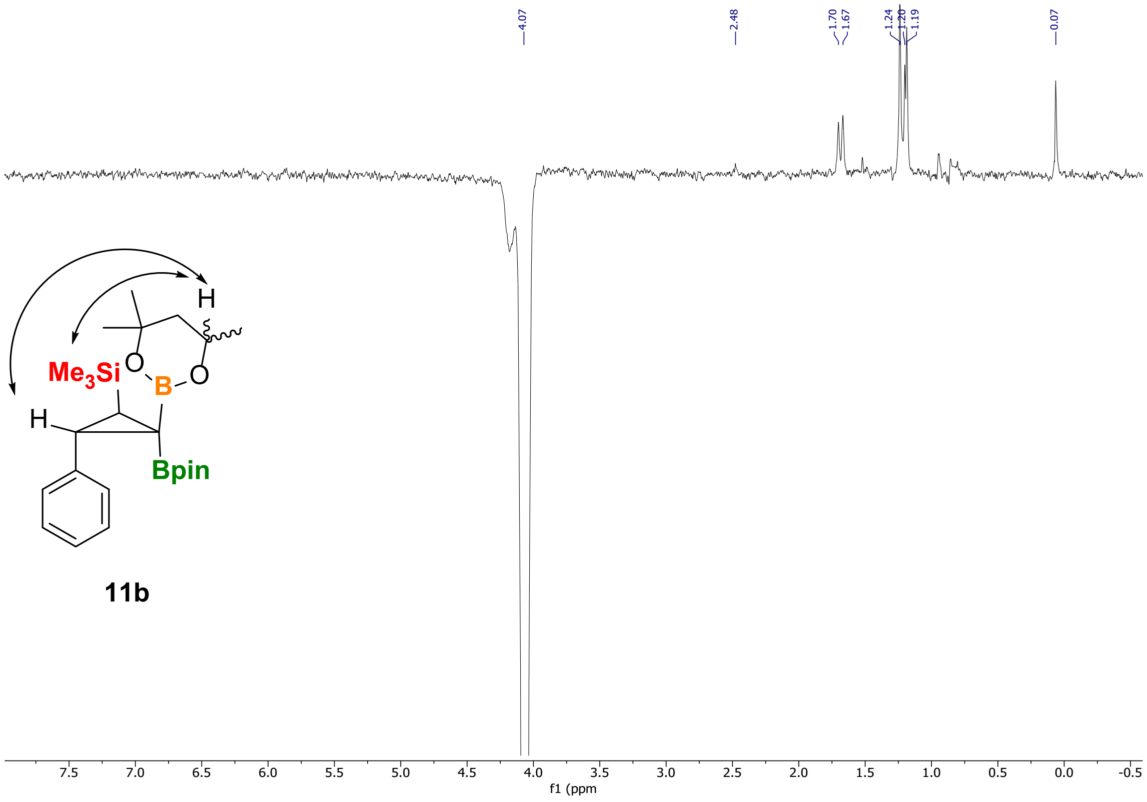

11b

^{13}C NMR (CDCl$_3$, 100 MHz)

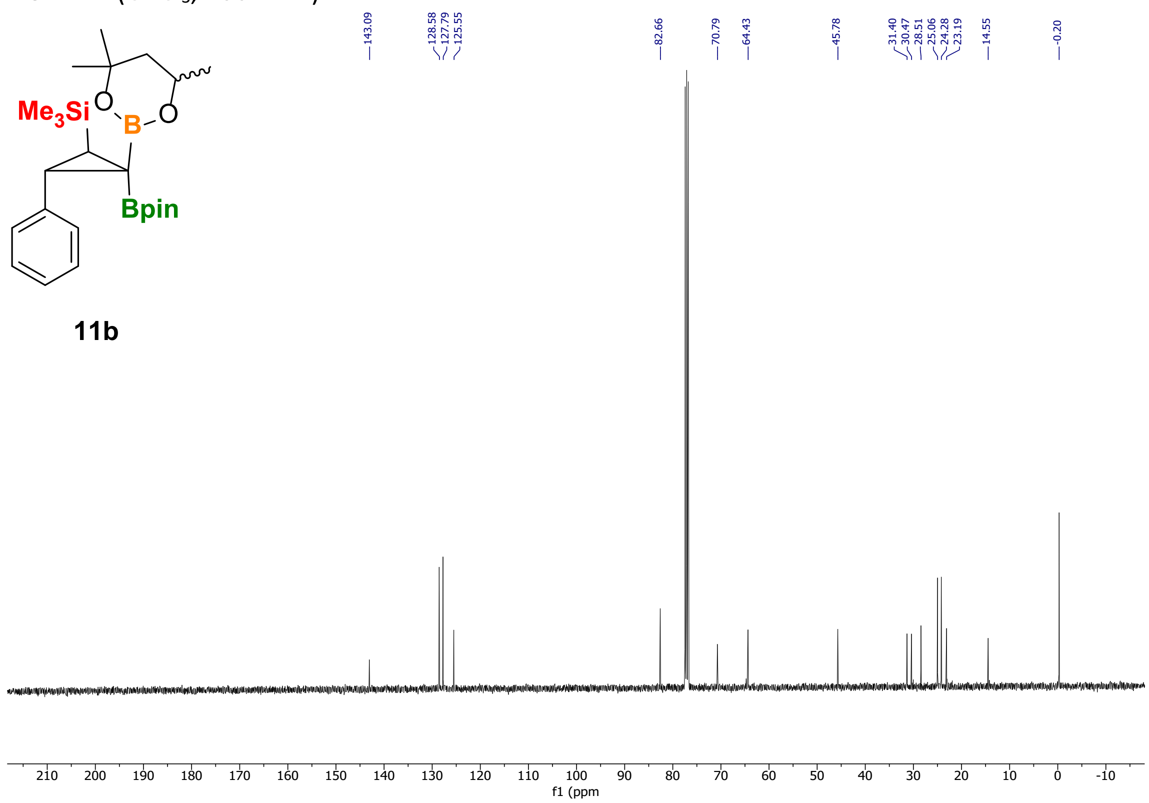

^{11}B NMR (CDCl$_3$, 128.3 MHz)

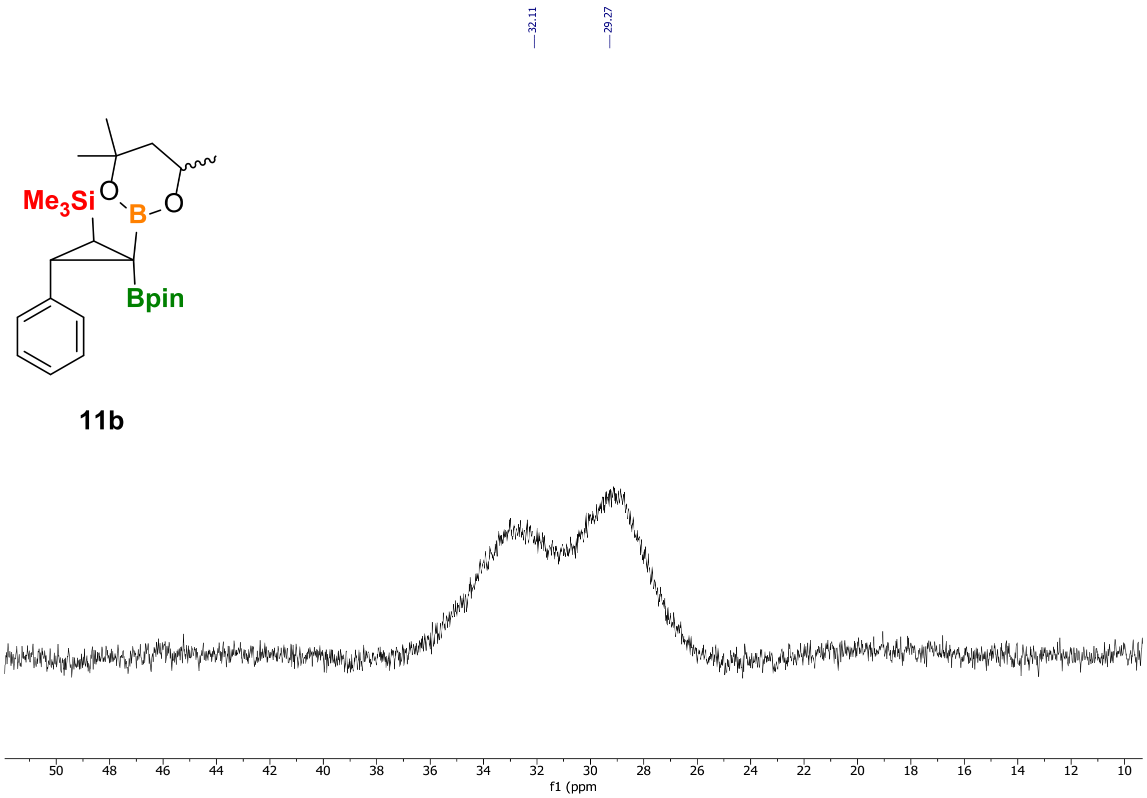

^{1}H NMR ($CDCl_3$, 400 MHz)

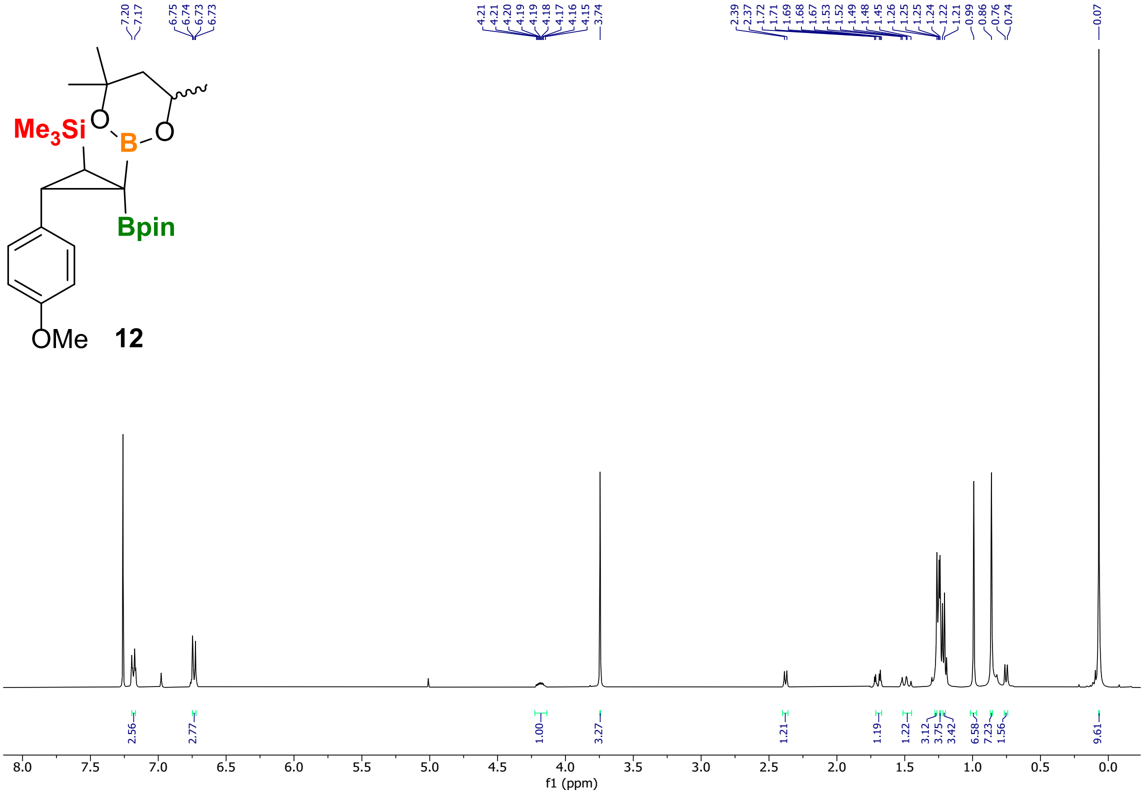

^{13}C NMR ($CDCl_3$, 100 MHz)

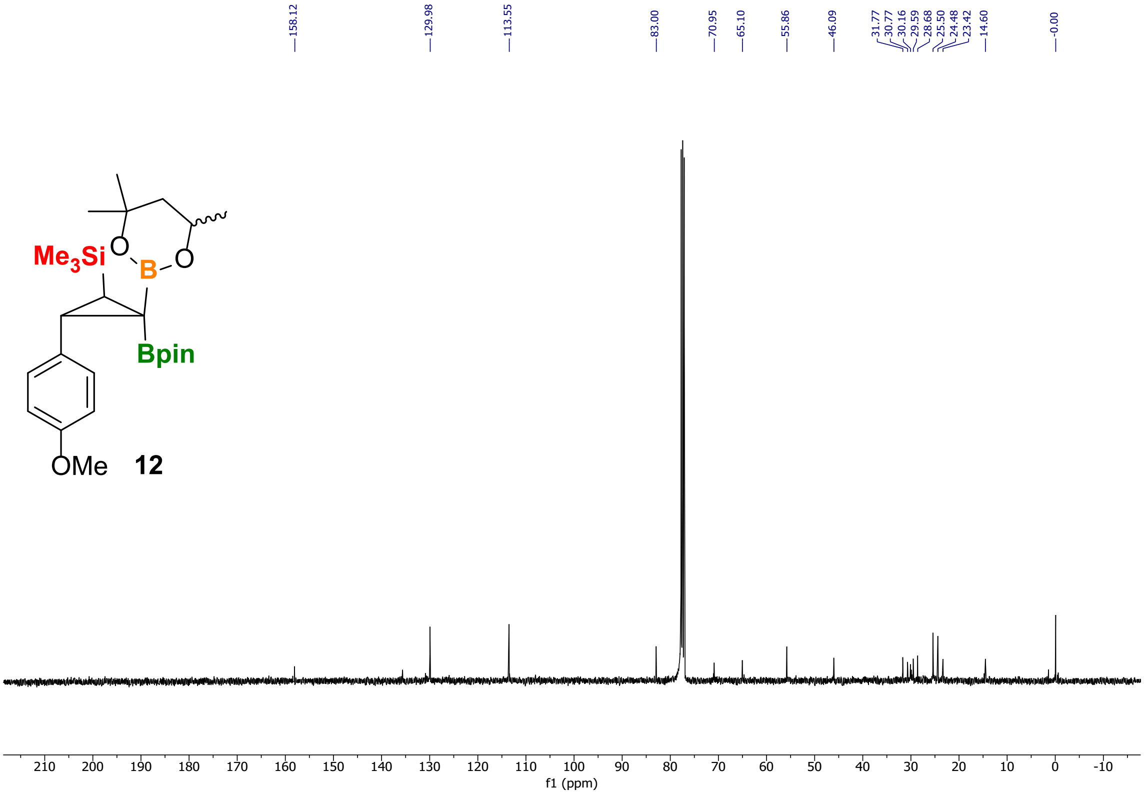

^{11}B NMR (CDCl$_3$, 128.3 MHz)

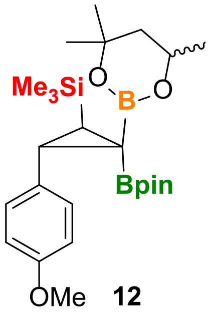

12

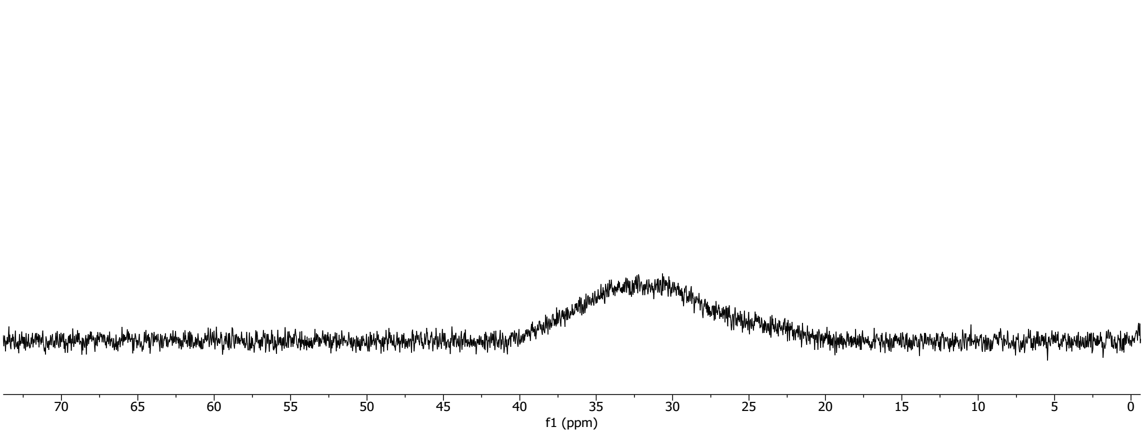

^{1}H NMR (CDCl$_3$, 400 MHz)

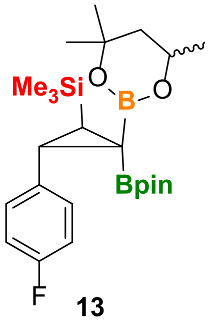

13

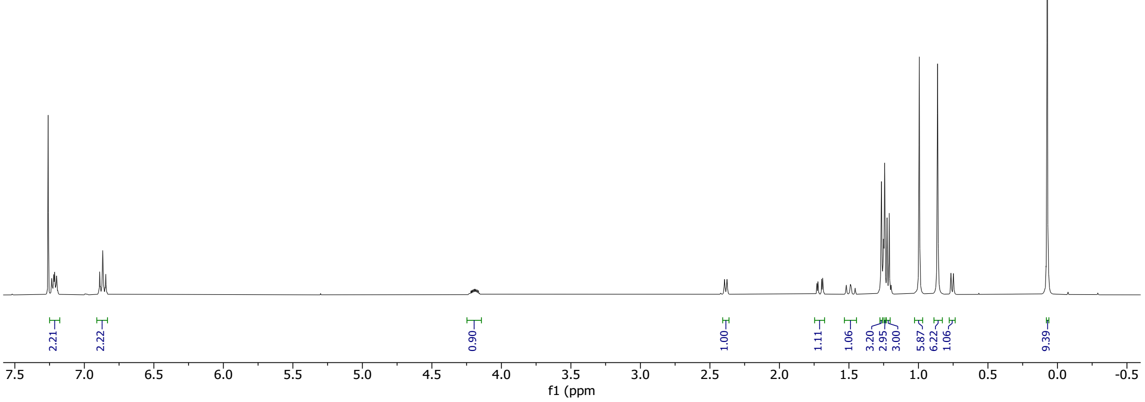

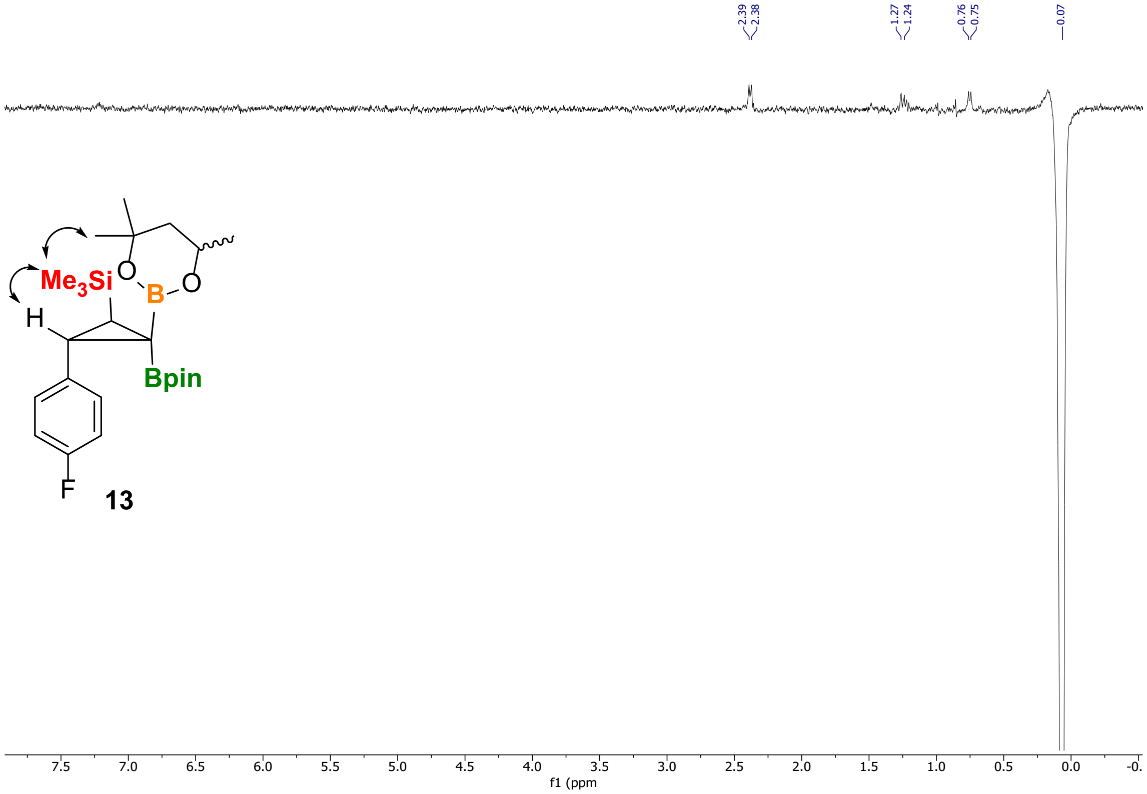

^{13}C NMR (CDCl$_3$, 100 MHz)

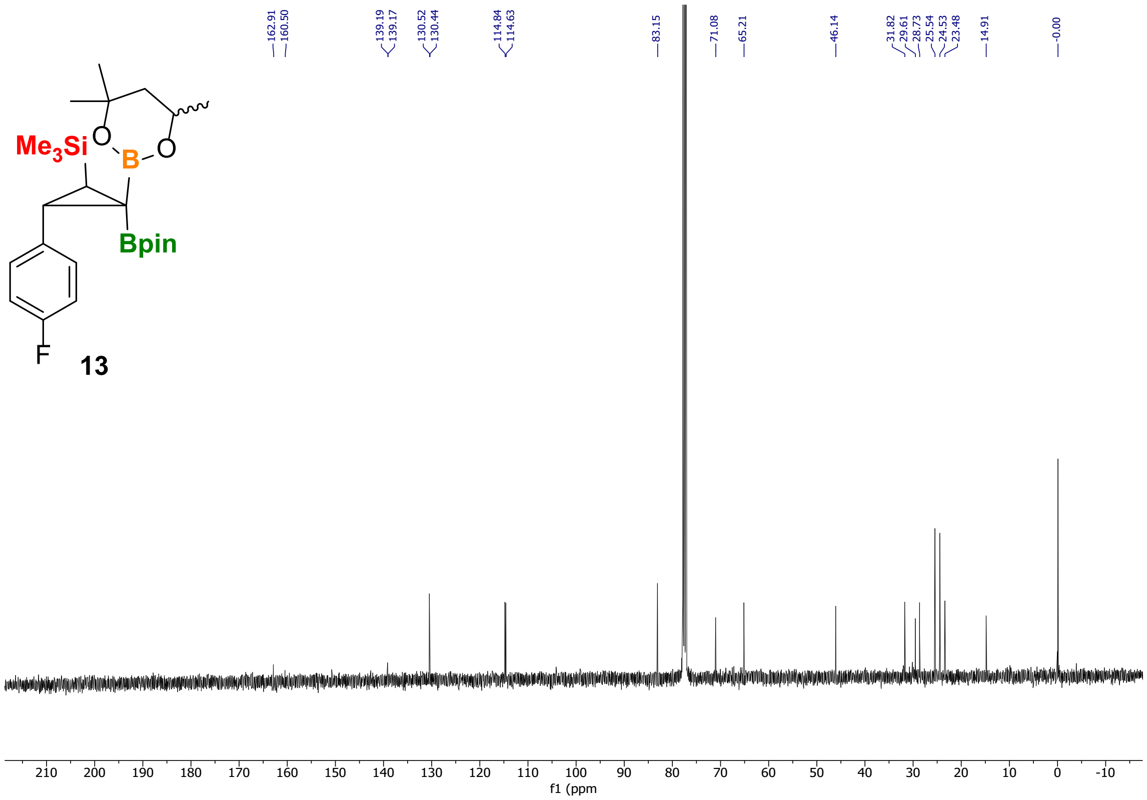

^{11}B NMR (CDCl$_3$, 128.3 MHz)

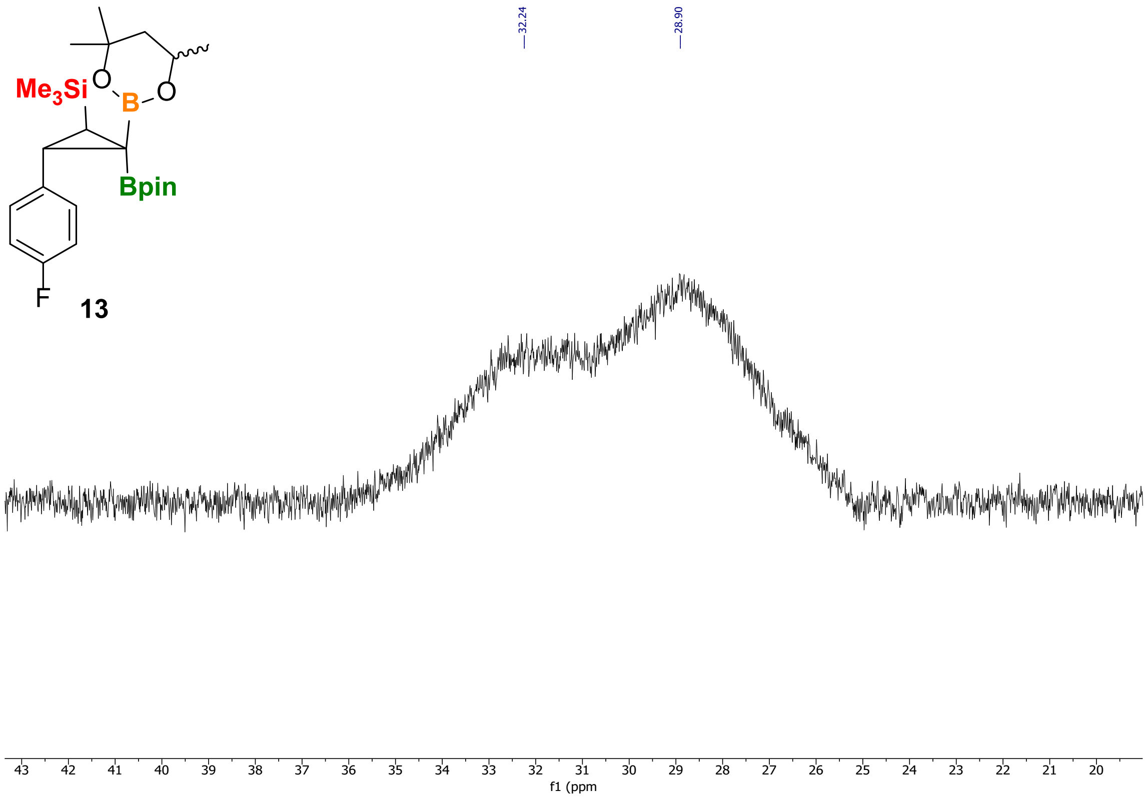

13

^{1}H NMR (CDCl$_3$, 400 MHz)

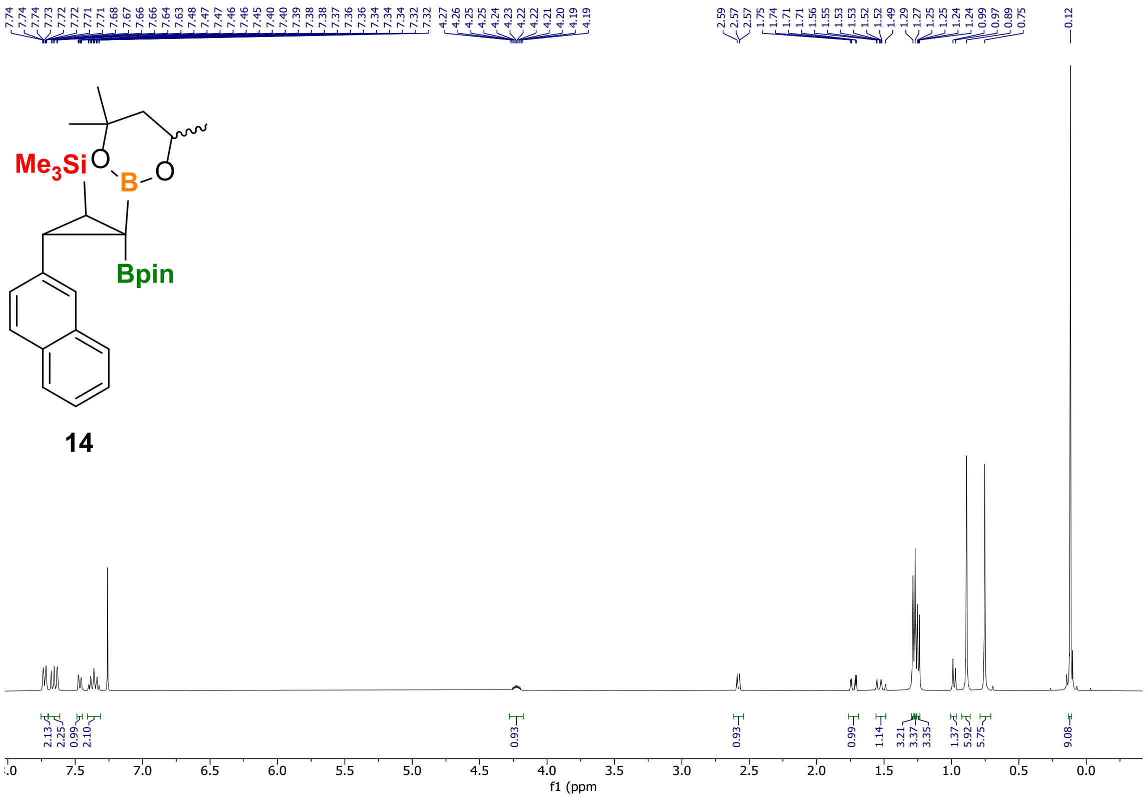

14

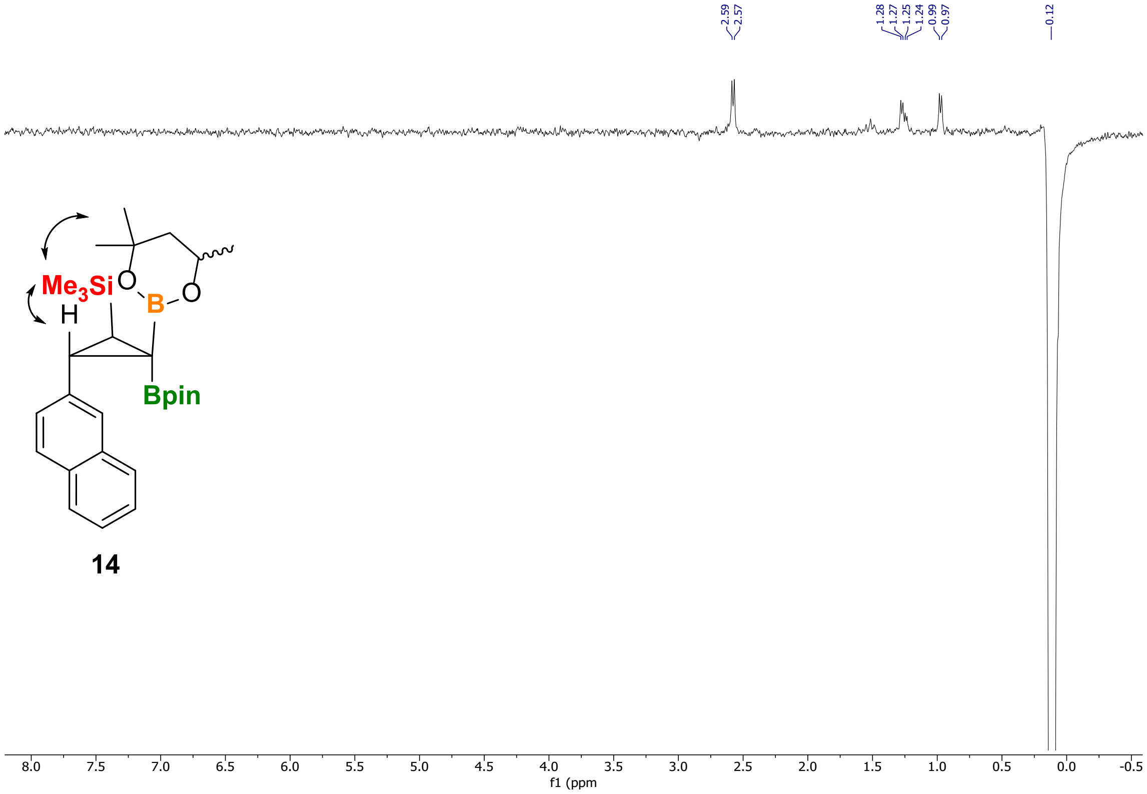

^{13}C NMR (CDCl$_3$, 100 MHz)

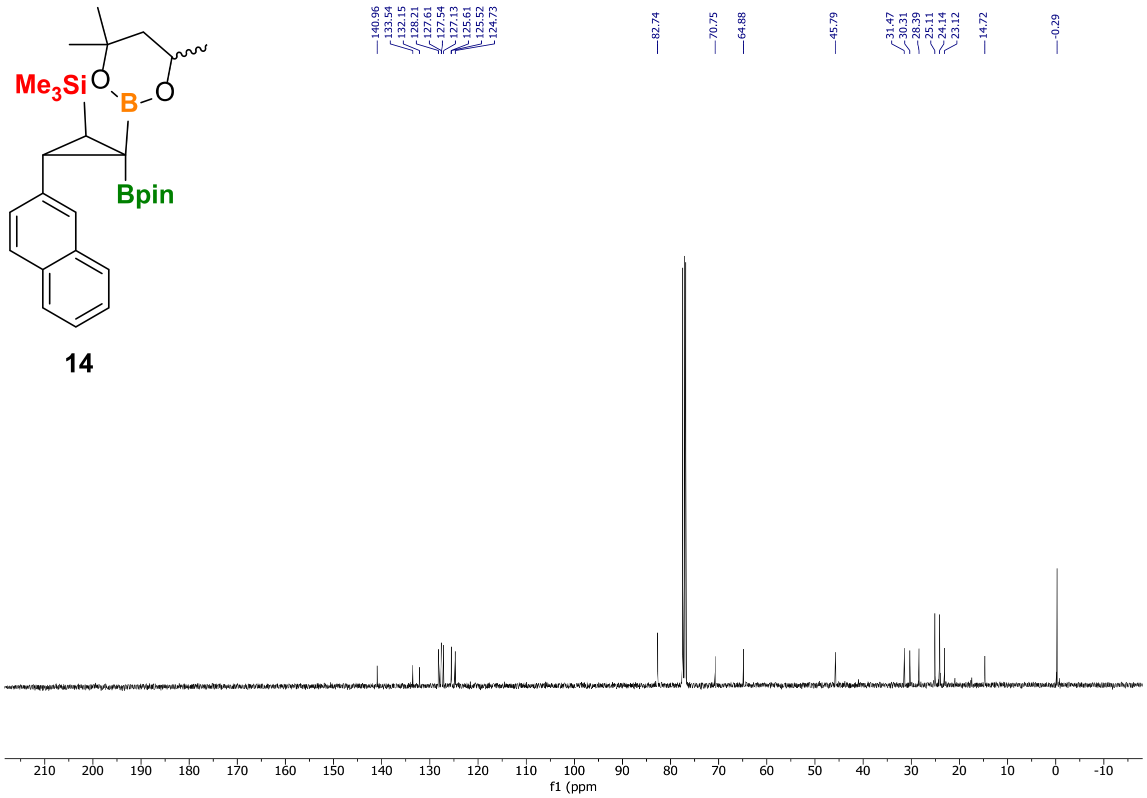

^{11}B NMR (CDCl$_3$, 128.3 MHz)

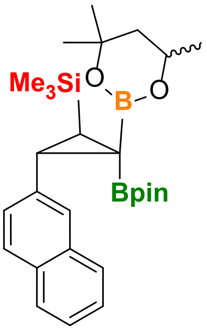

14

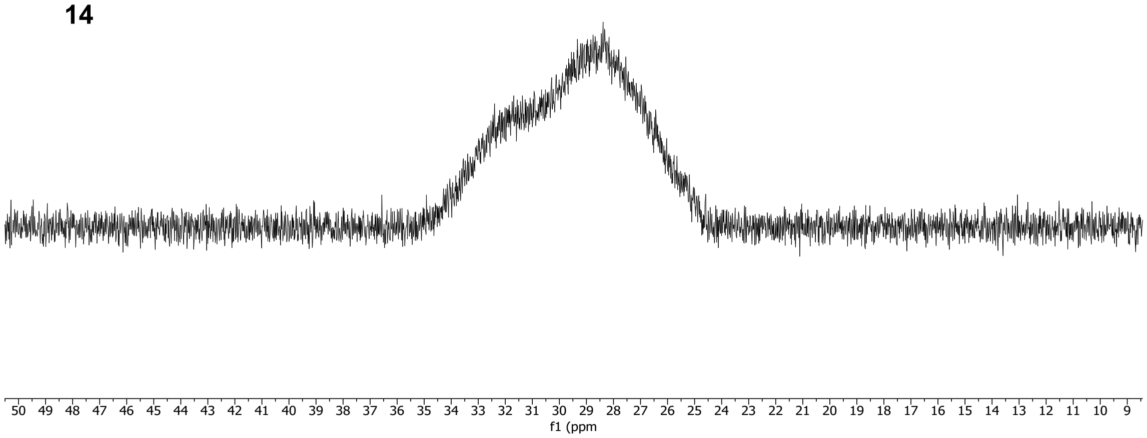

^{1}H NMR (CDCl$_3$, 400 MHz)

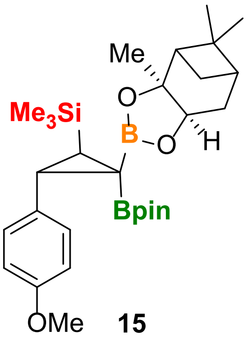

15

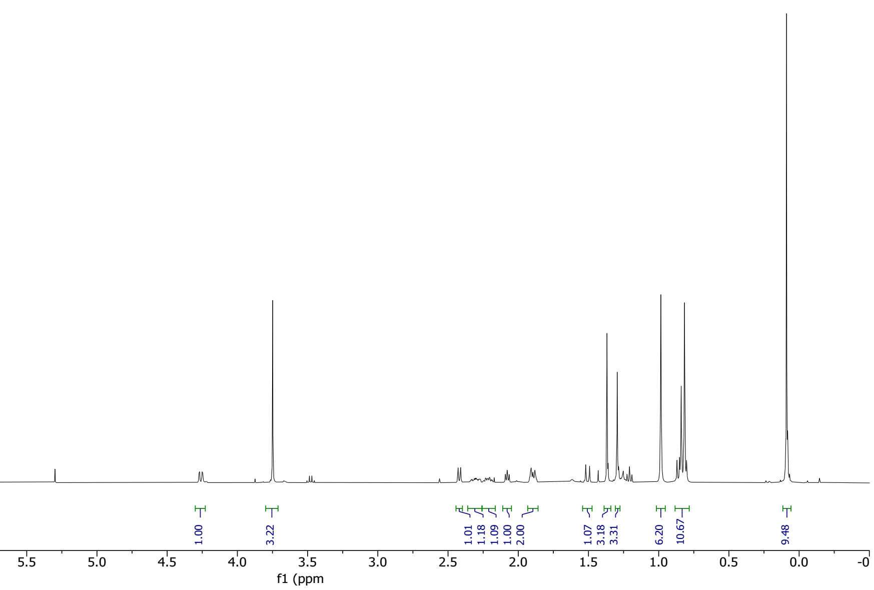

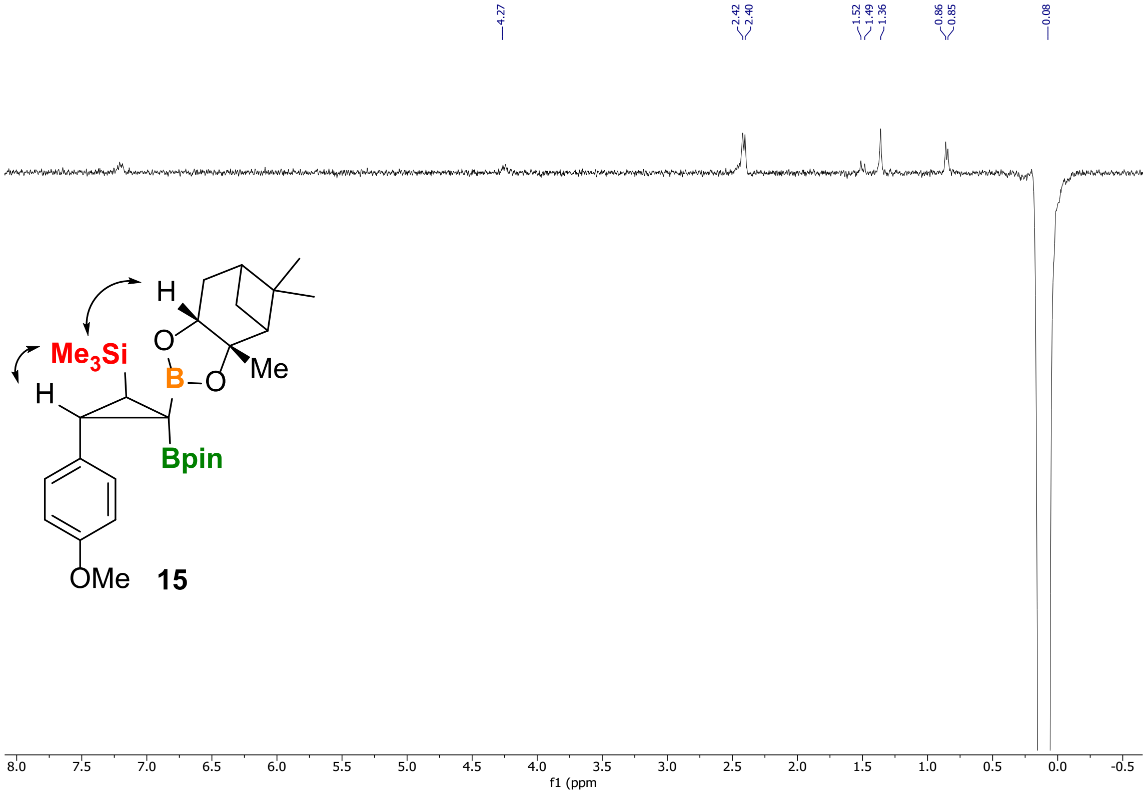

^{13}C NMR (CDCl$_3$, 100 MHz)

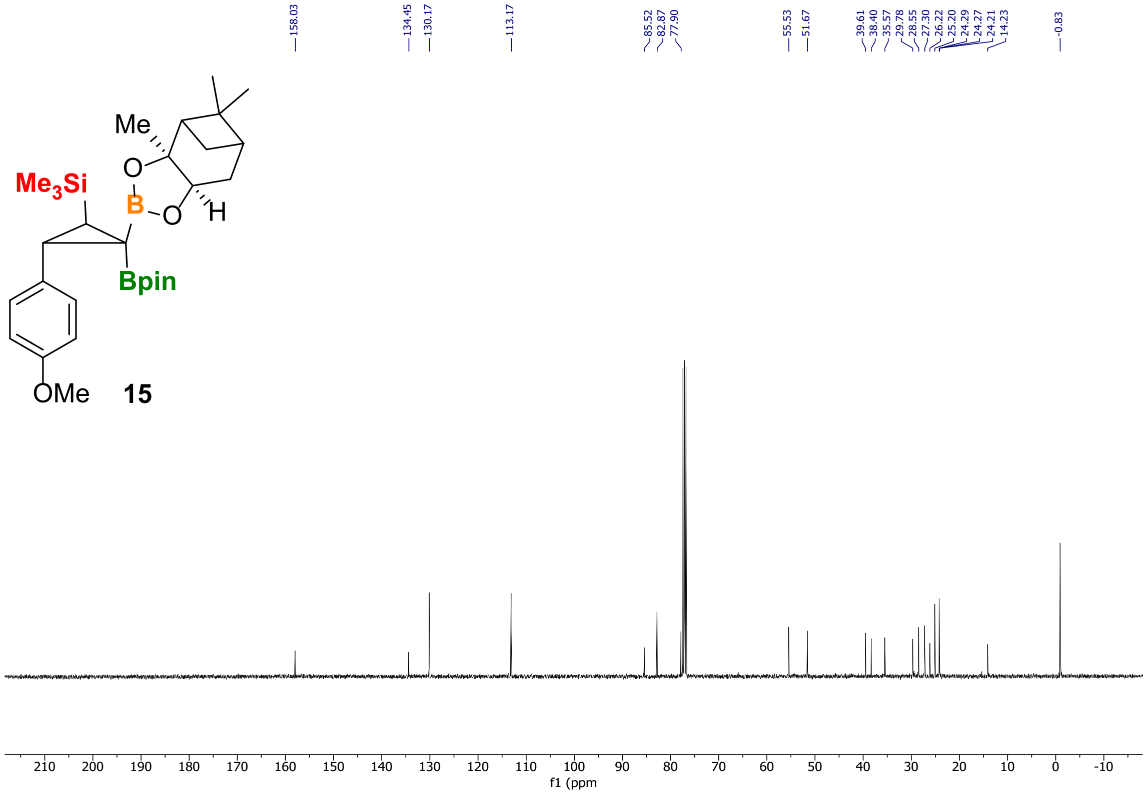

^{11}B NMR (CDCl$_3$, 128.3 MHz)

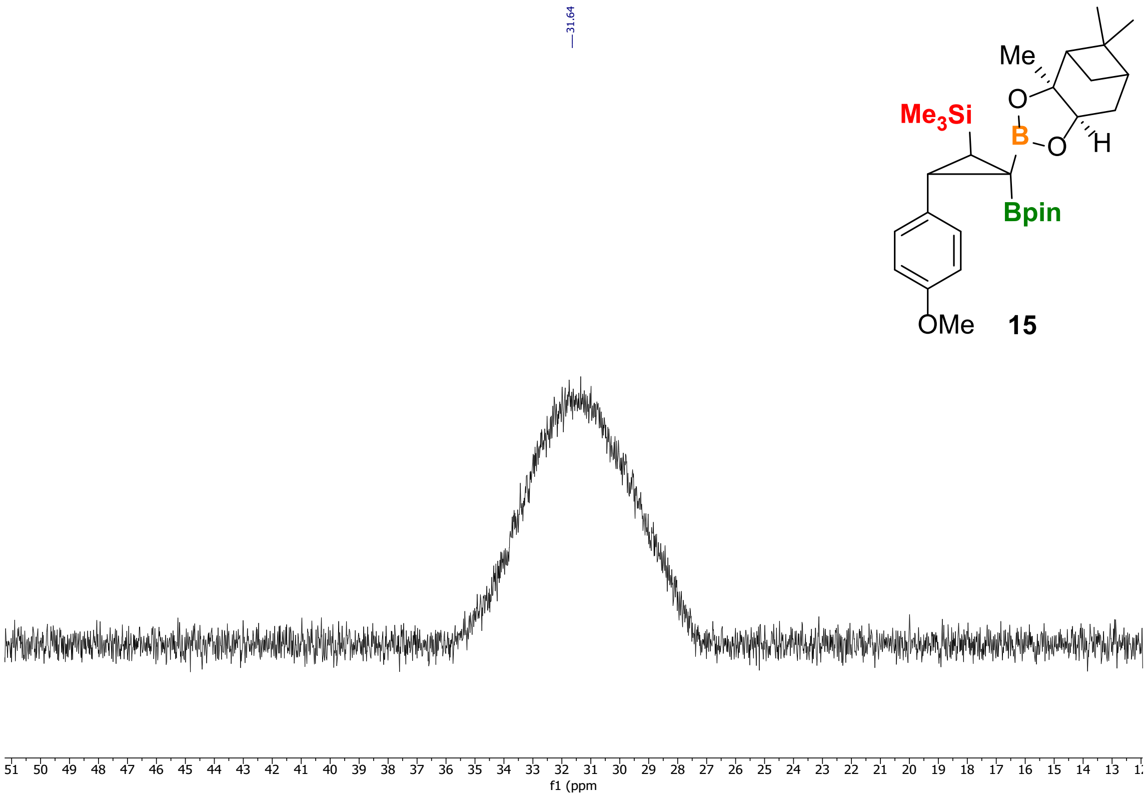

15

^{1}H NMR (CDCl$_3$, 400 MHz)

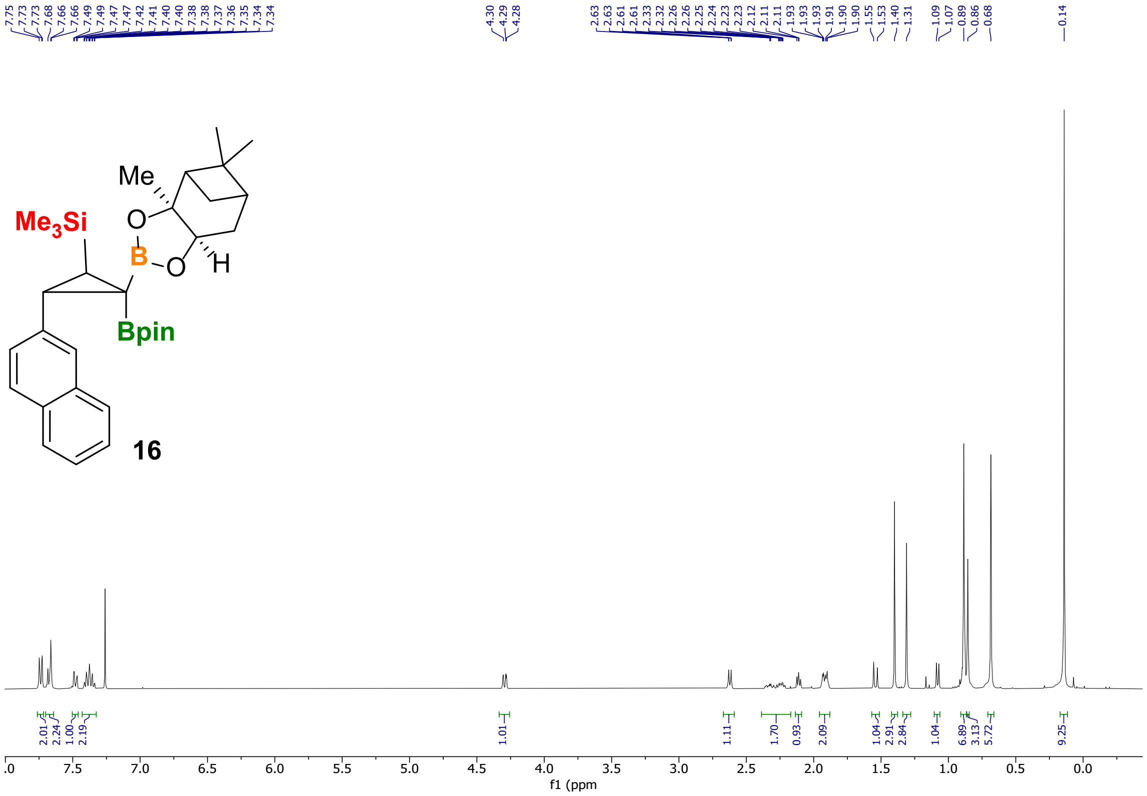

16

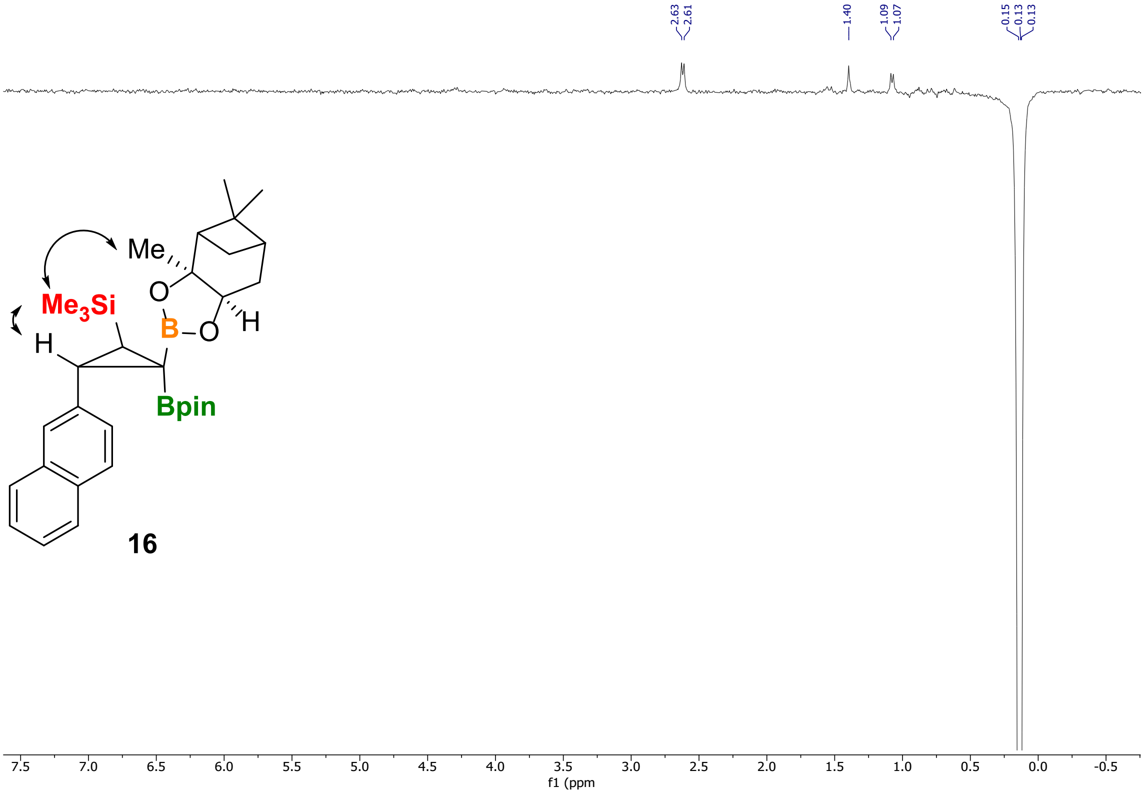

^{13}C NMR (CDCl$_3$, 100 MHz)

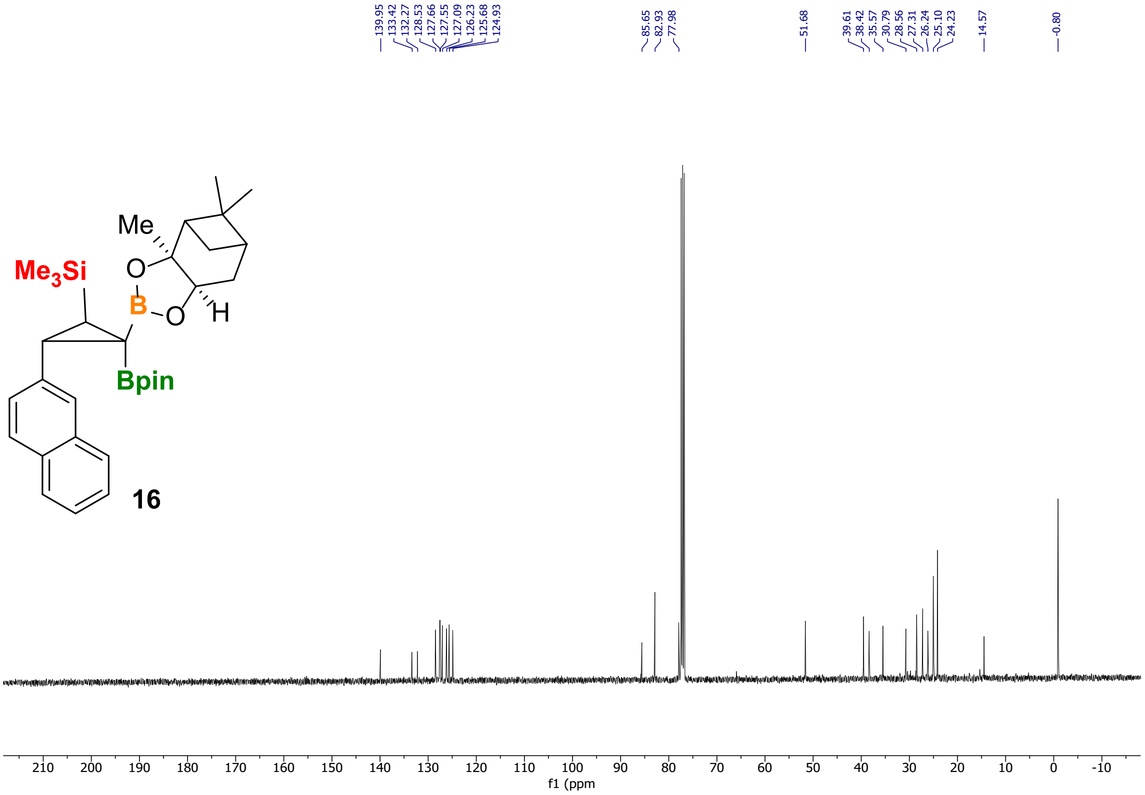

^{11}B NMR (CDCl$_3$, 128.3 MHz)

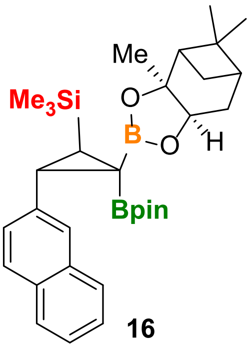

16

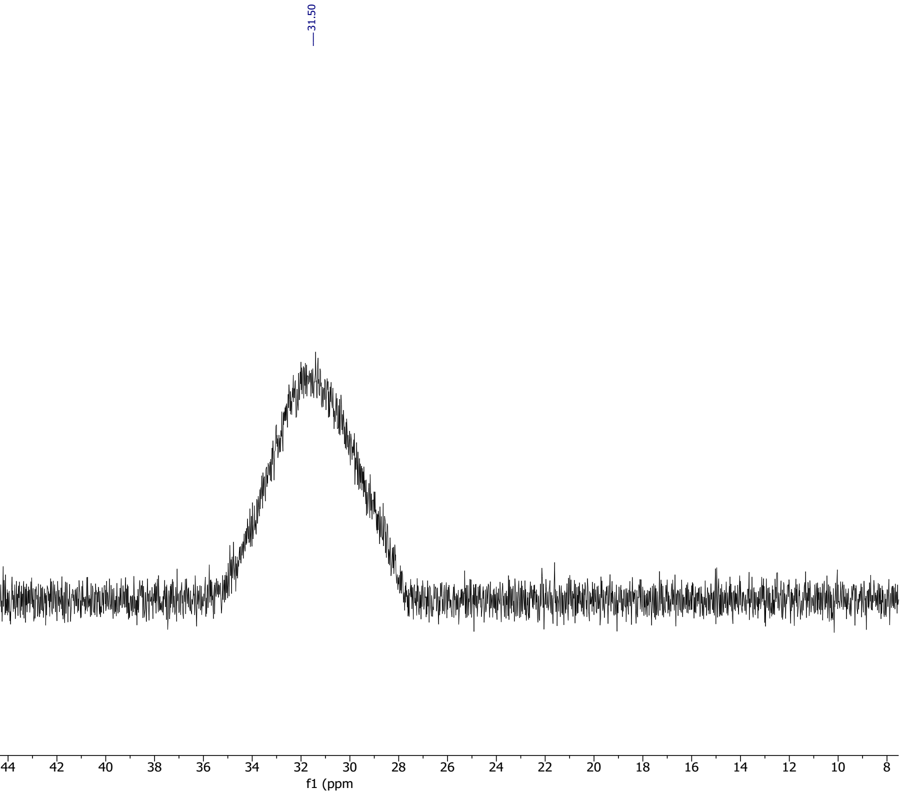

^{1}H NMR (CDCl$_3$, 400 MHz)

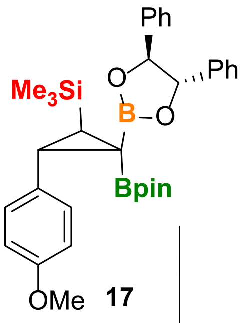

17

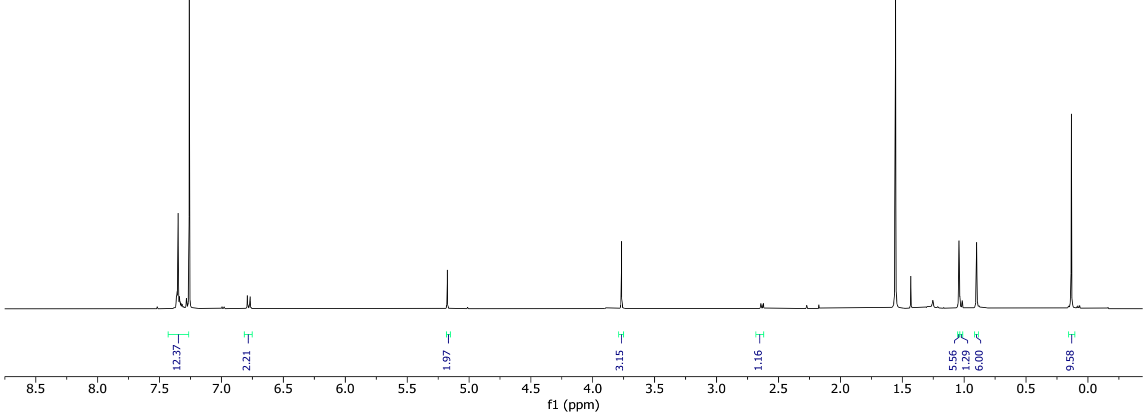

^{13}C NMR (CDCl$_3$, 100 MHz)

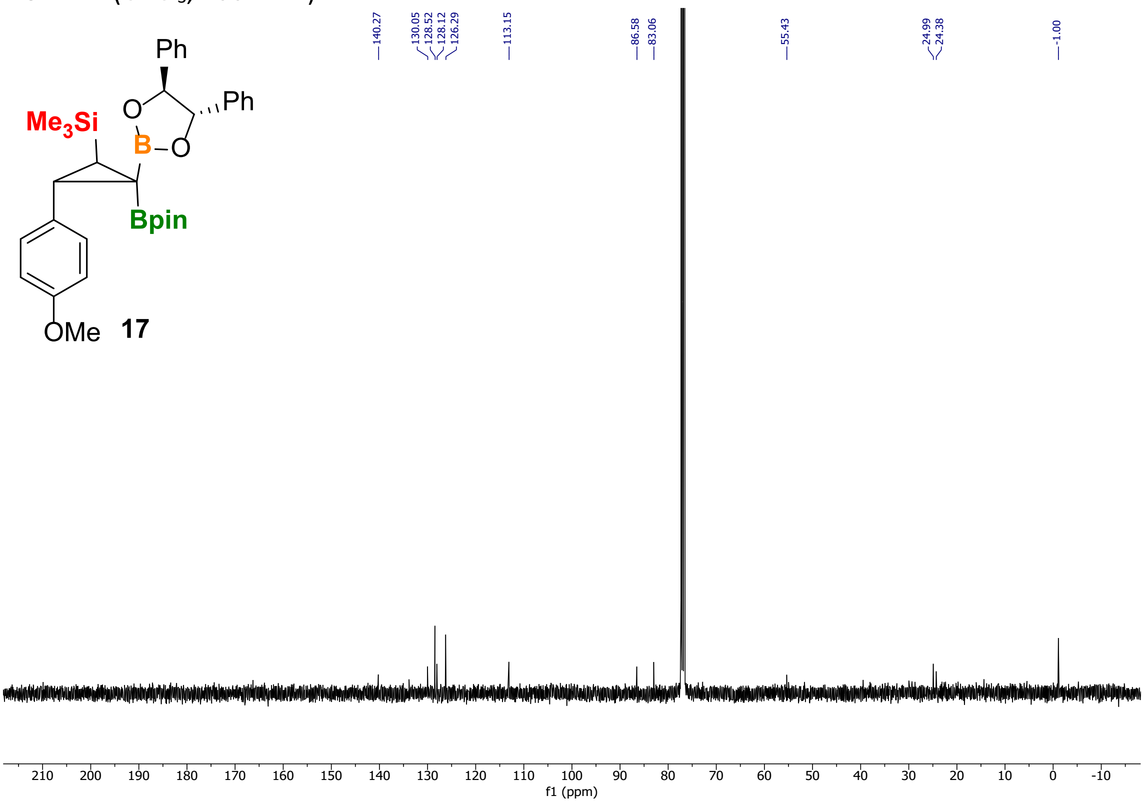

^{11}B NMR (CDCl$_3$, 128.3 MHz)

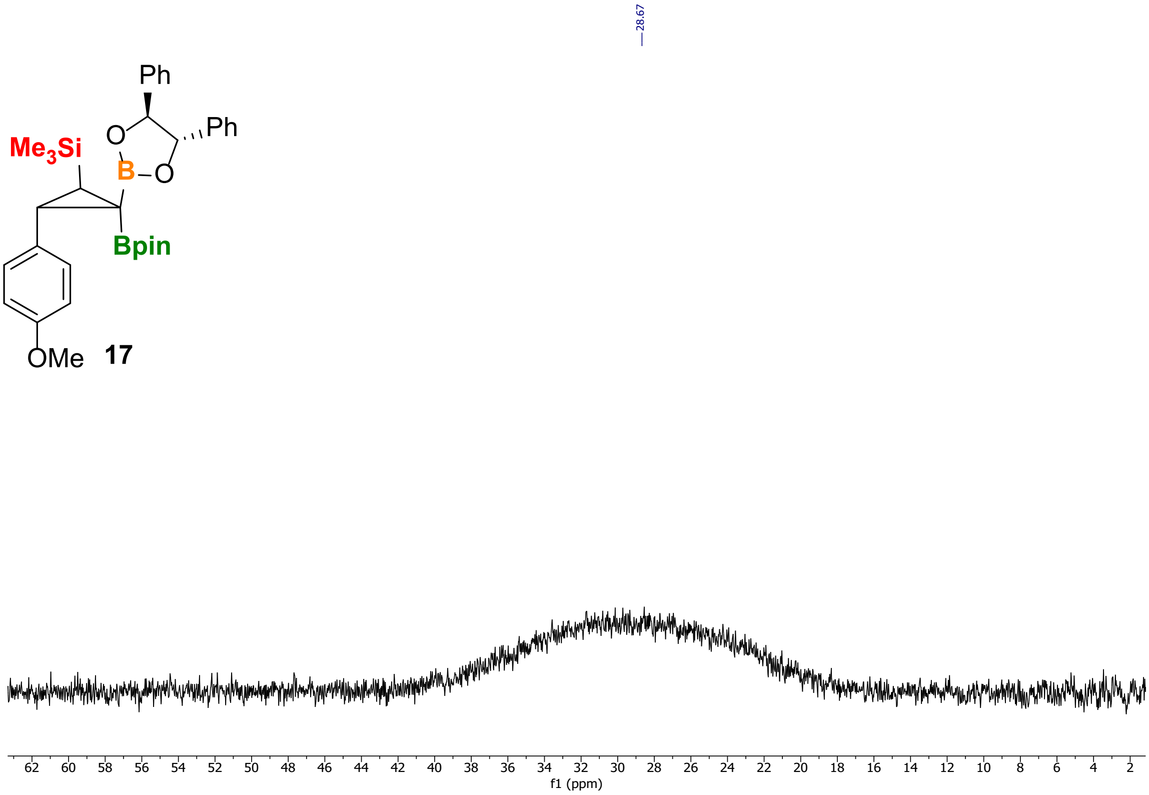

^{1}H NMR (CDCl$_3$, 400 MHz)

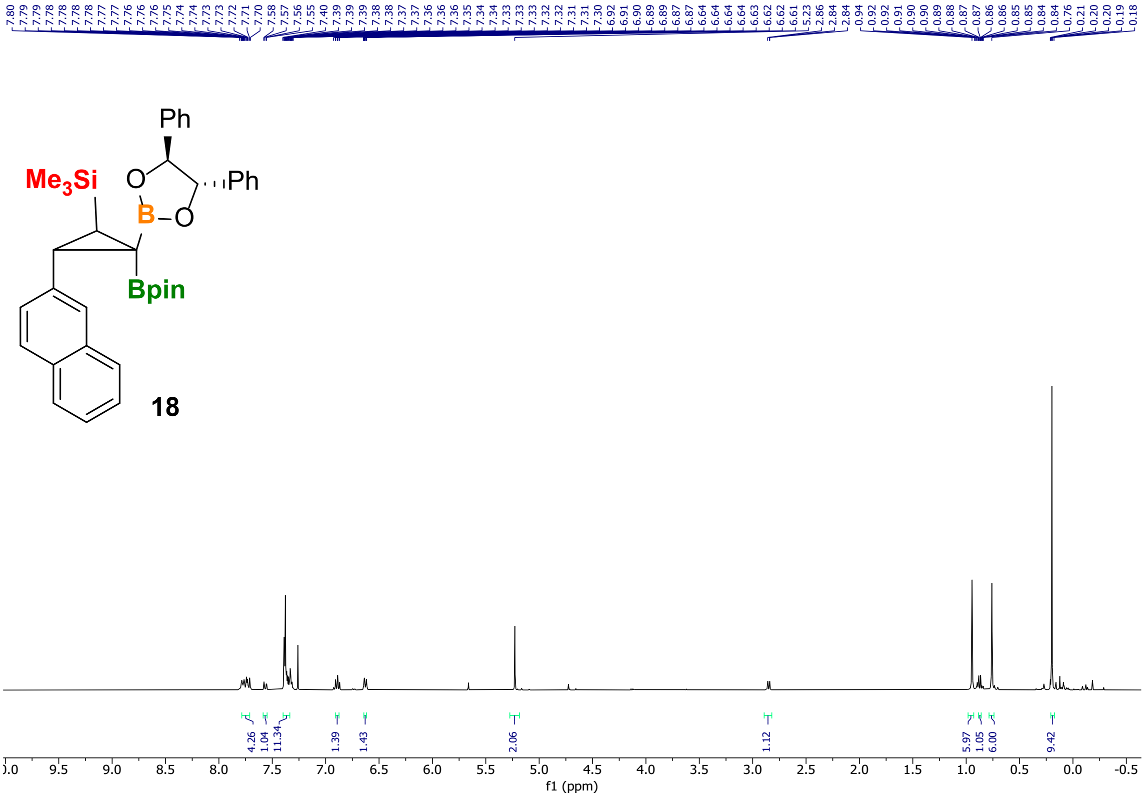

^{13}C NMR (CDCl$_3$, 100 MHz)

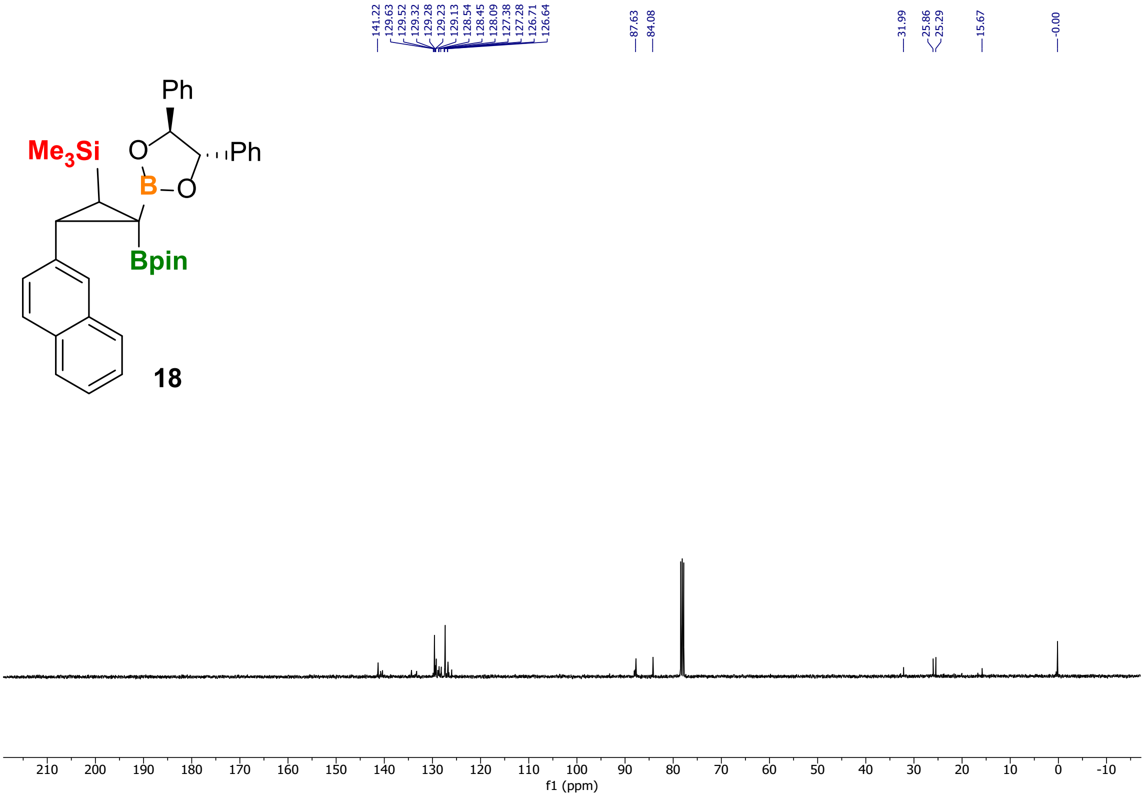

^{11}B NMR ($CDCl_3$, 128.3 MHz)

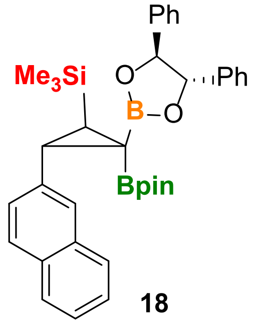

18

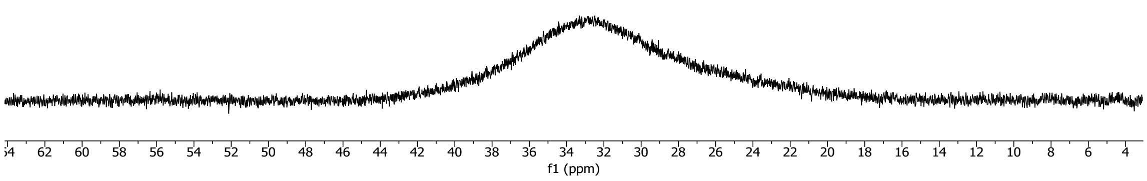

^{1}H NMR ($CDCl_3$, 400 MHz)

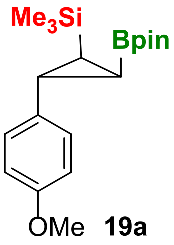

19a

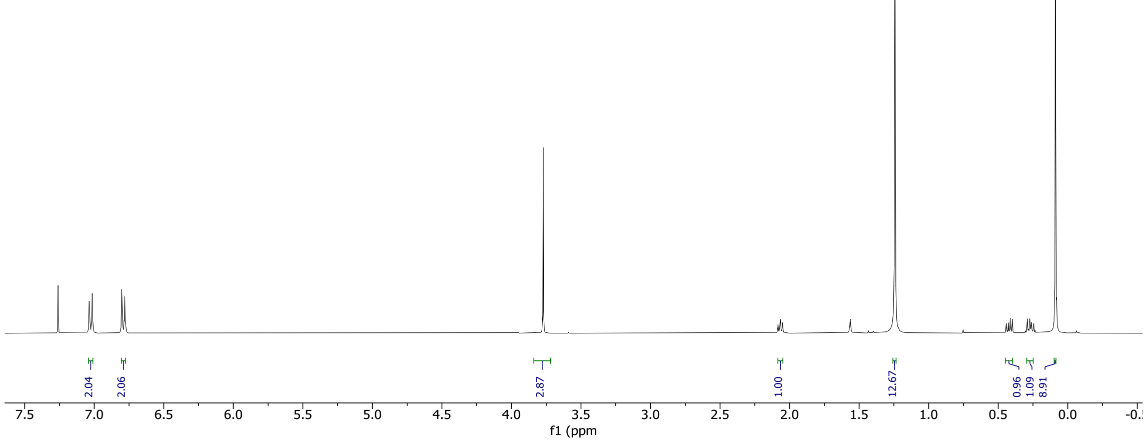

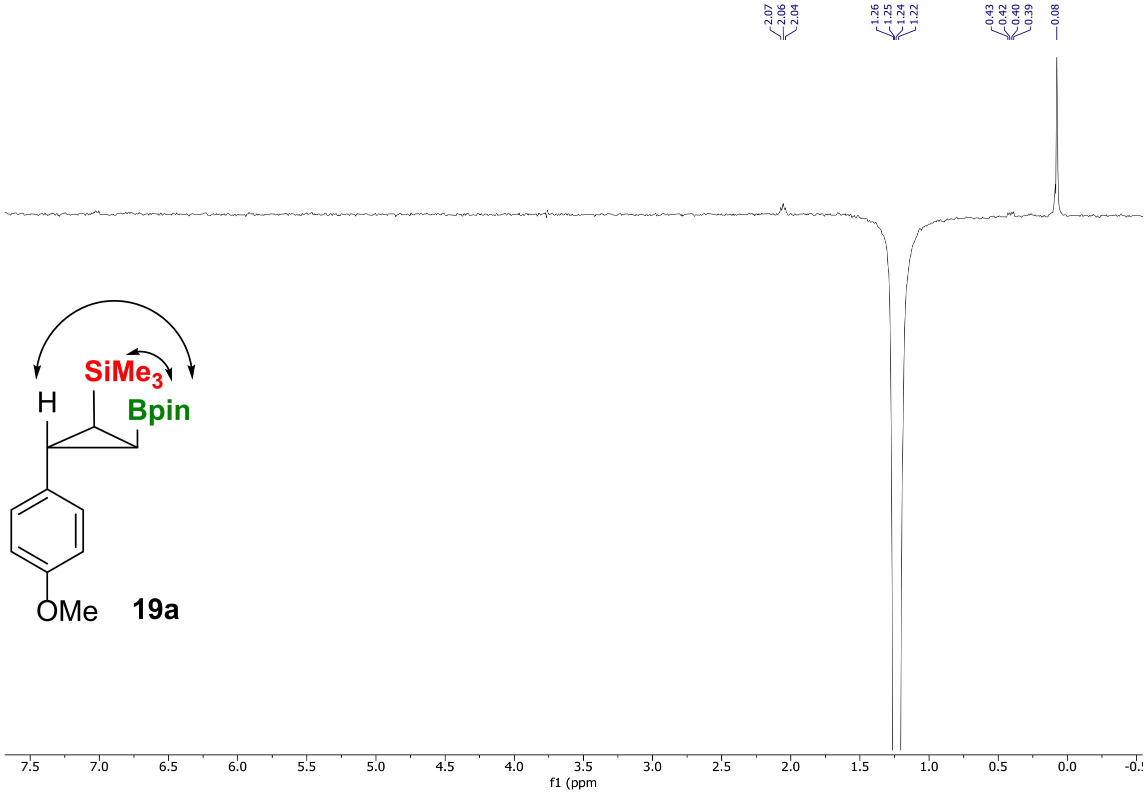

^{13}C NMR ($CDCl_3$, 100 MHz)

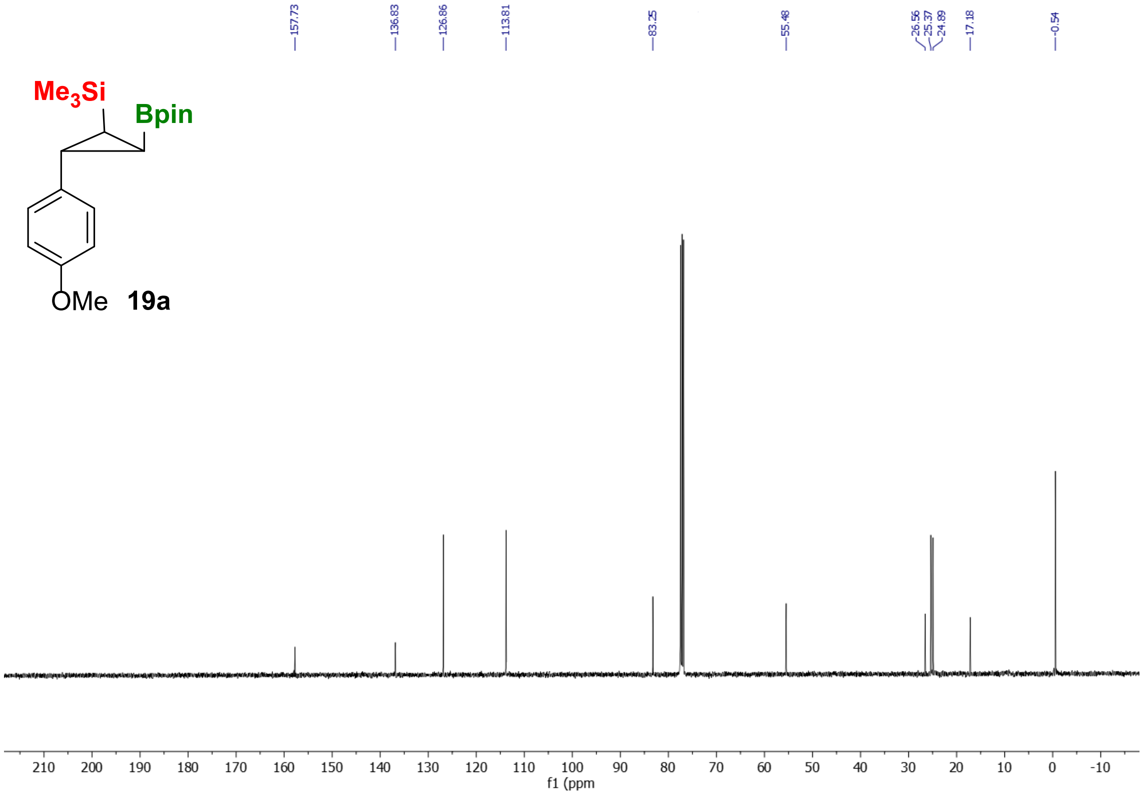

^{11}B NMR (CDCl$_3$, 128.3 MHz)

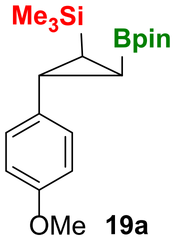

19a

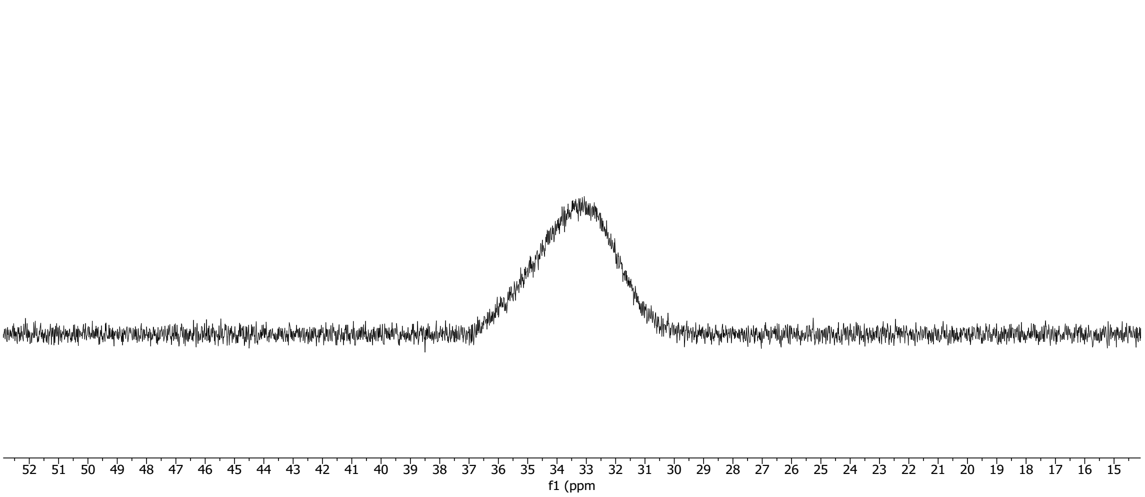

^{1}H NMR (CDCl$_3$, 400 MHz)

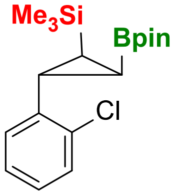

20a

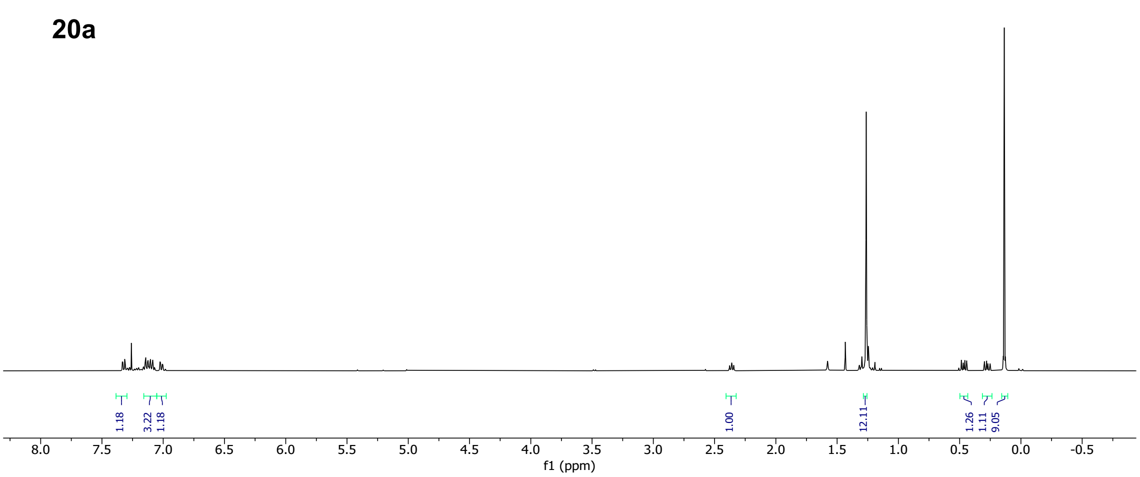

^{13}C NMR (CDCl$_3$, 100 MHz)

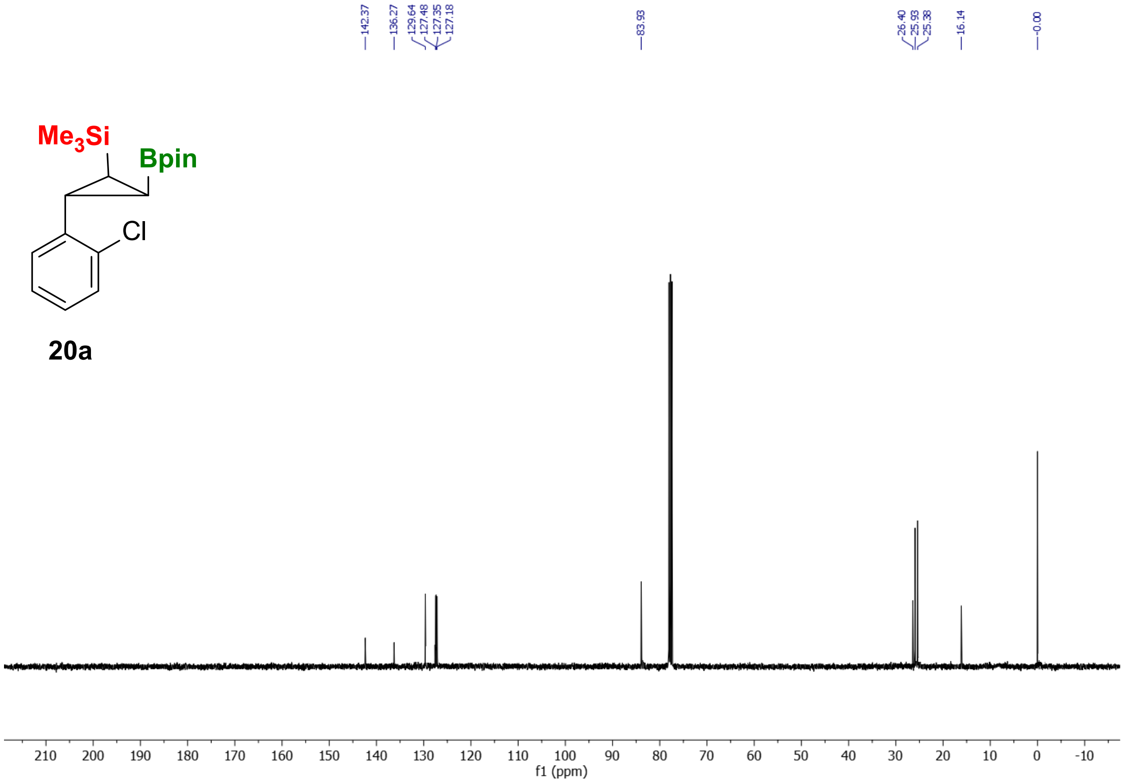

^{11}B NMR (CDCl$_3$, 128.3 MHz)

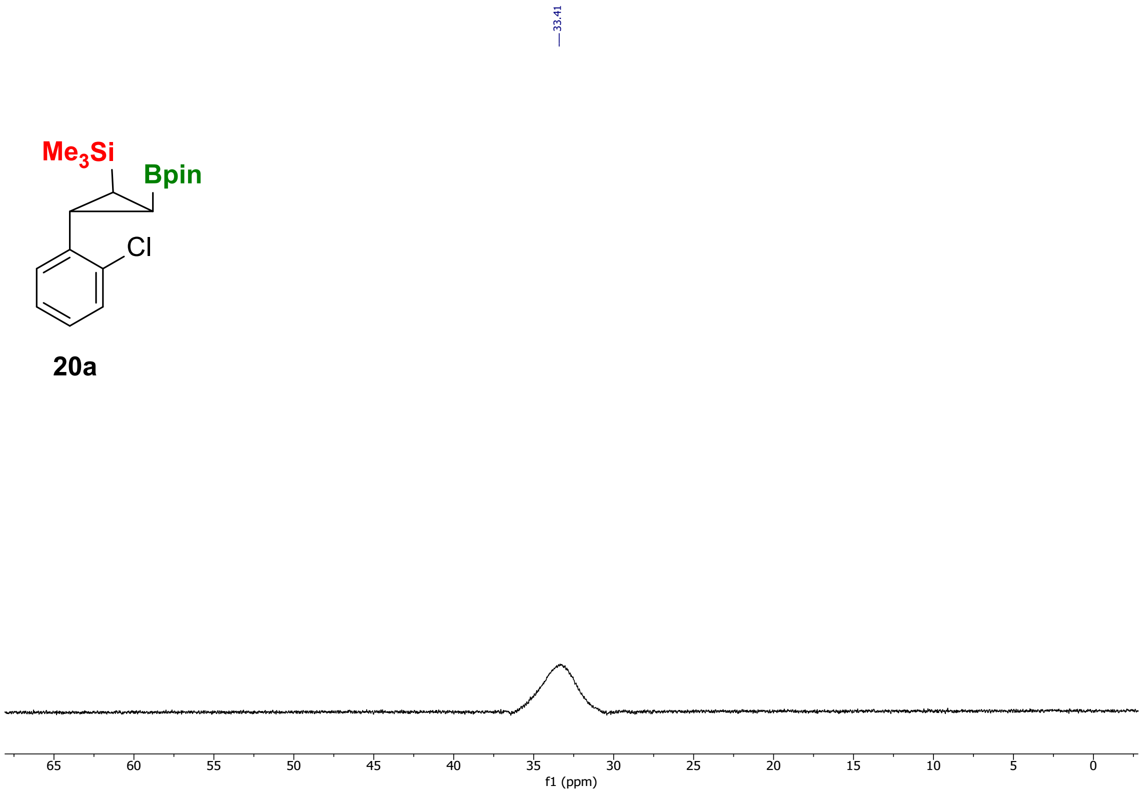

^{1}H NMR ($CDCl_3$, 400 MHz)

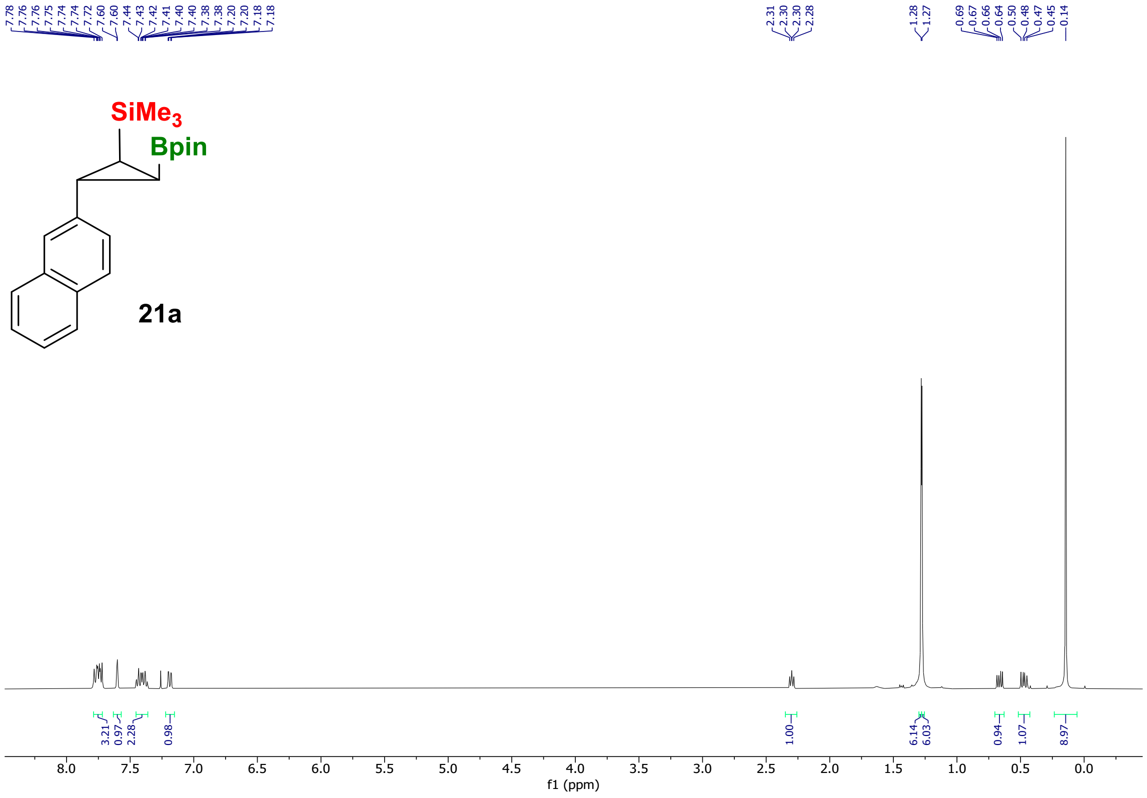

^{13}C NMR ($CDCl_3$, 100 MHz)

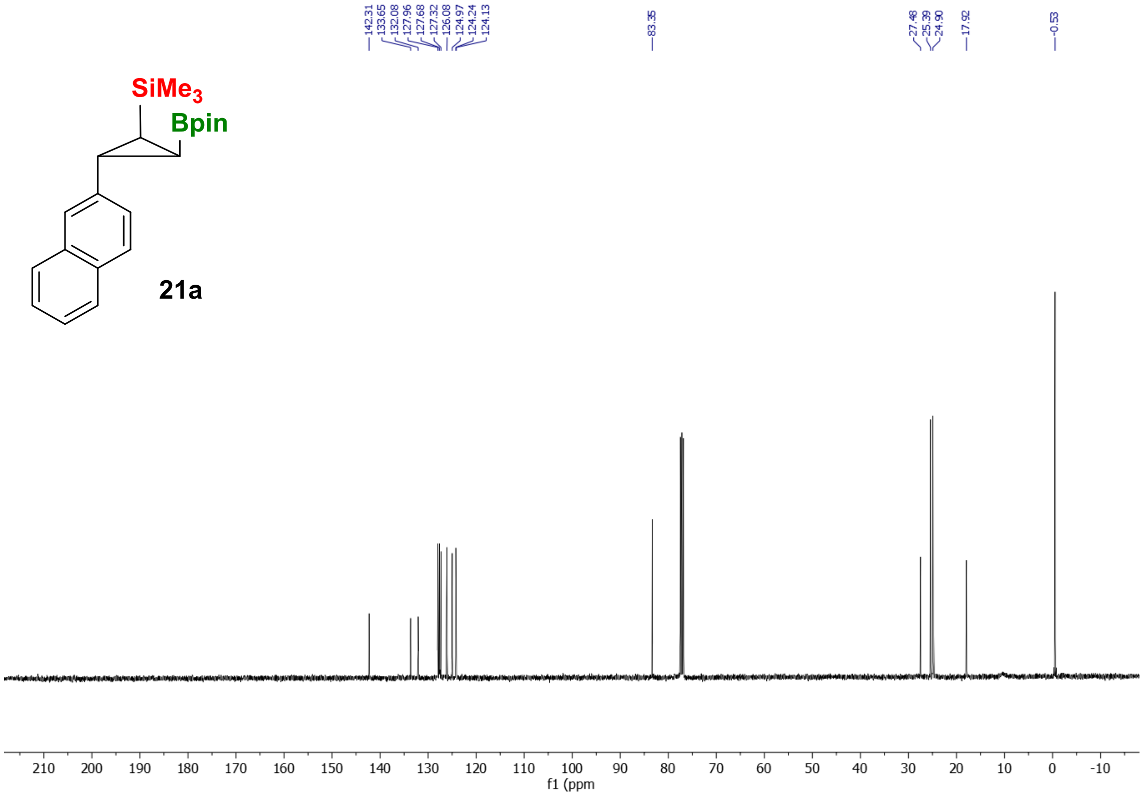

^{11}B NMR (CDCl$_3$, 128.3 MHz)

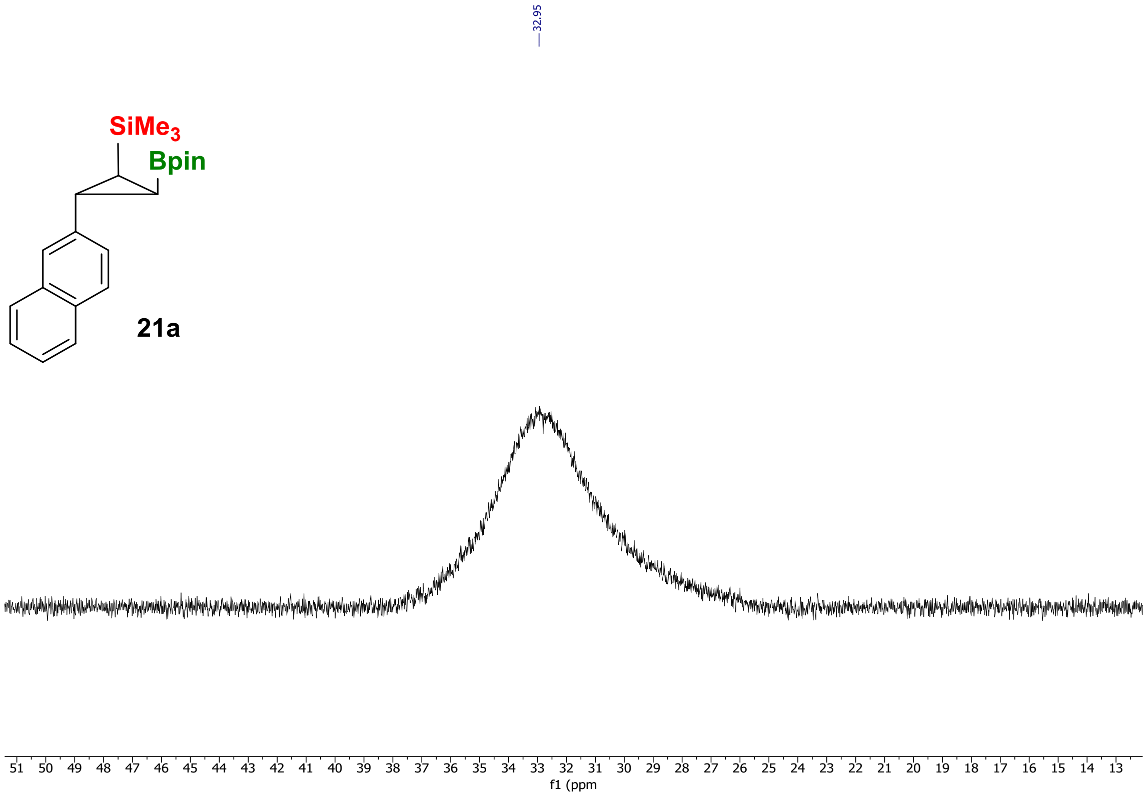

^{1}H NMR (CDCl$_3$, 400 MHz)

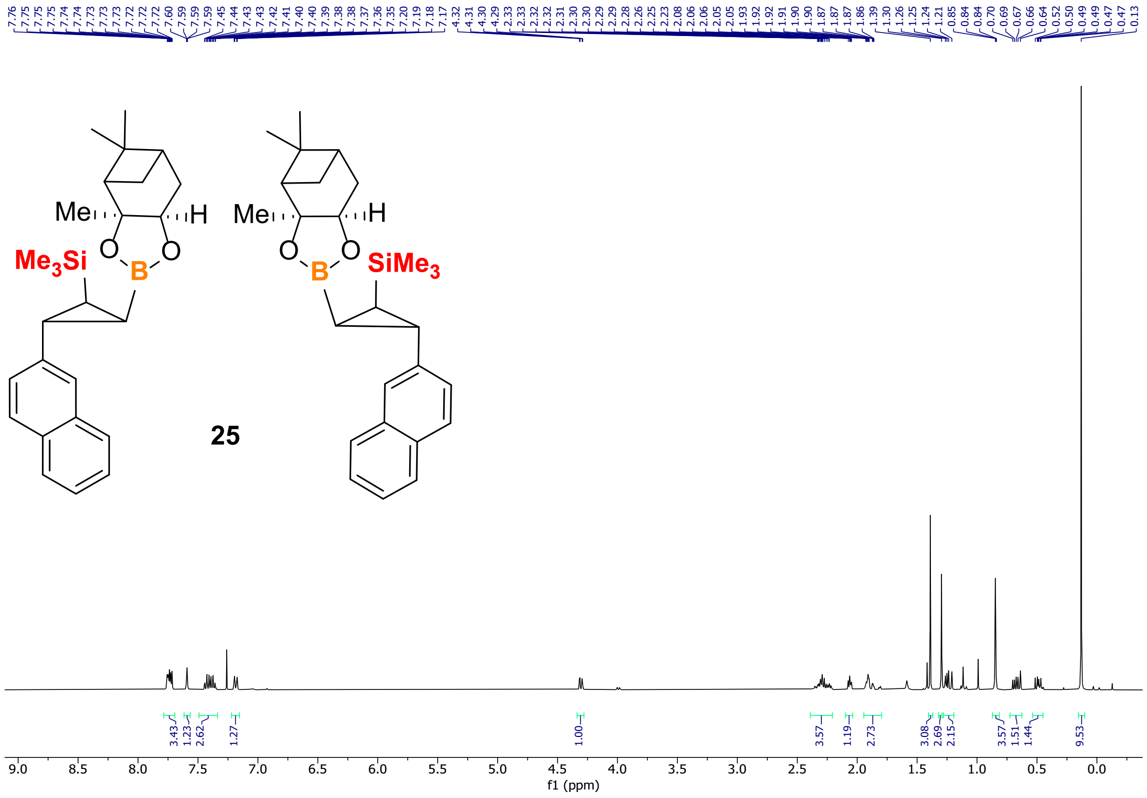

^{13}C NMR ($CDCl_3$, 100 MHz)

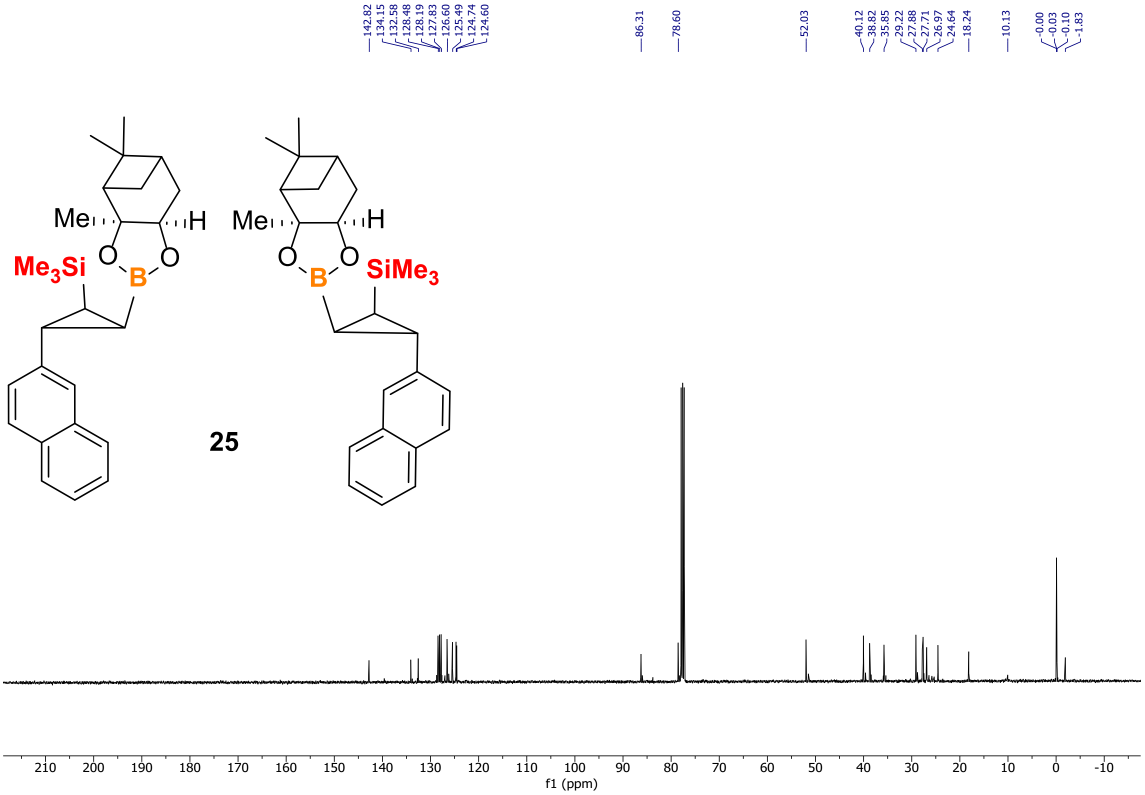

^{11}B NMR ($CDCl_3$, 128.3 MHz)

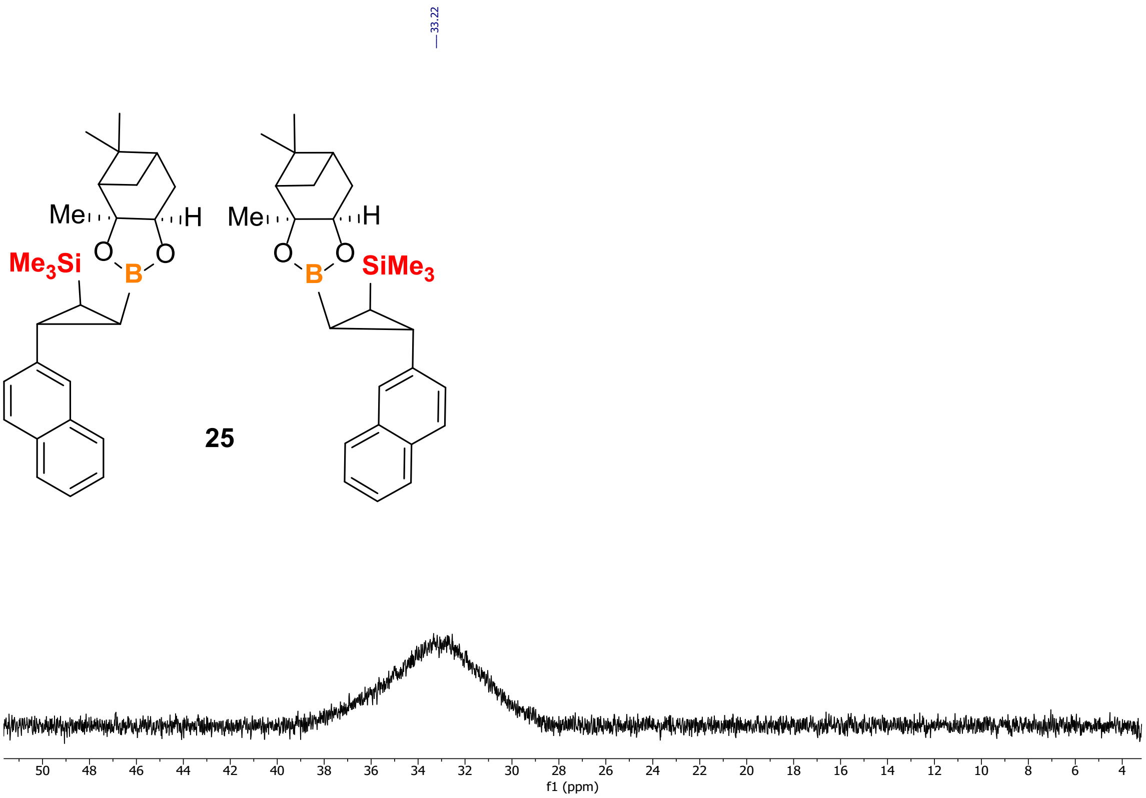

^{1}H NMR ($CDCl_3$, 400 MHz)

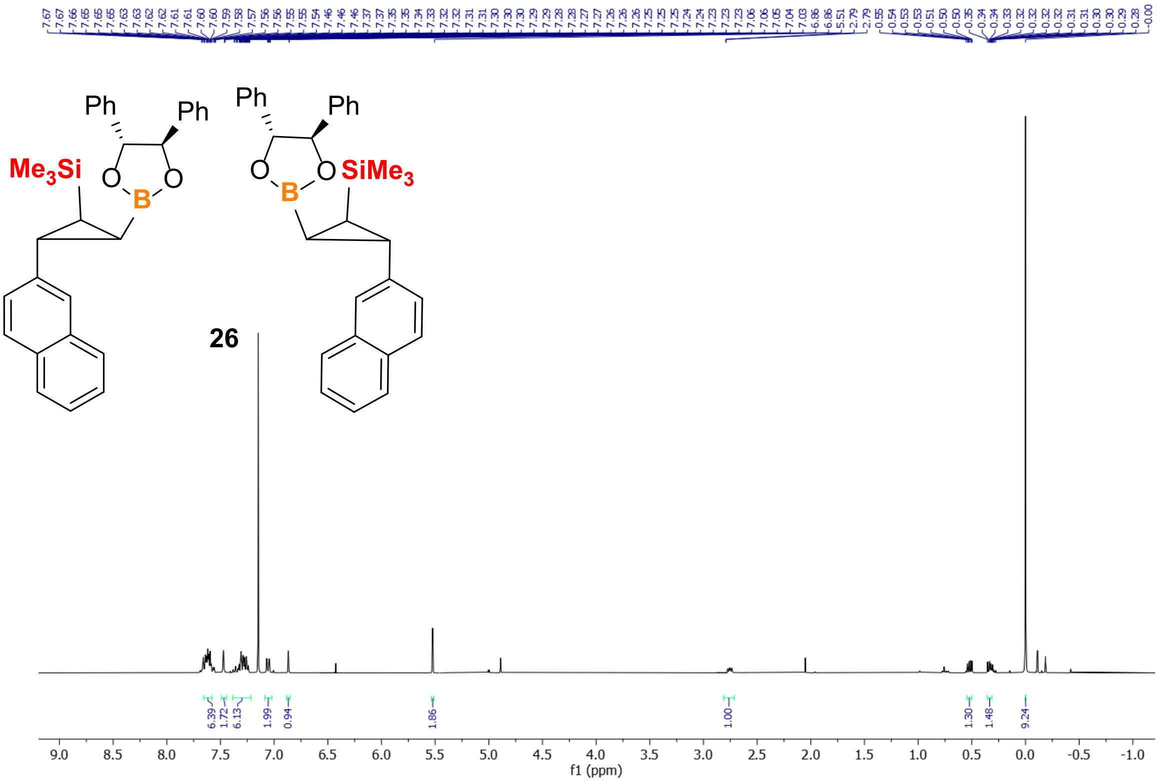

^{13}C NMR ($CDCl_3$, 100 MHz)

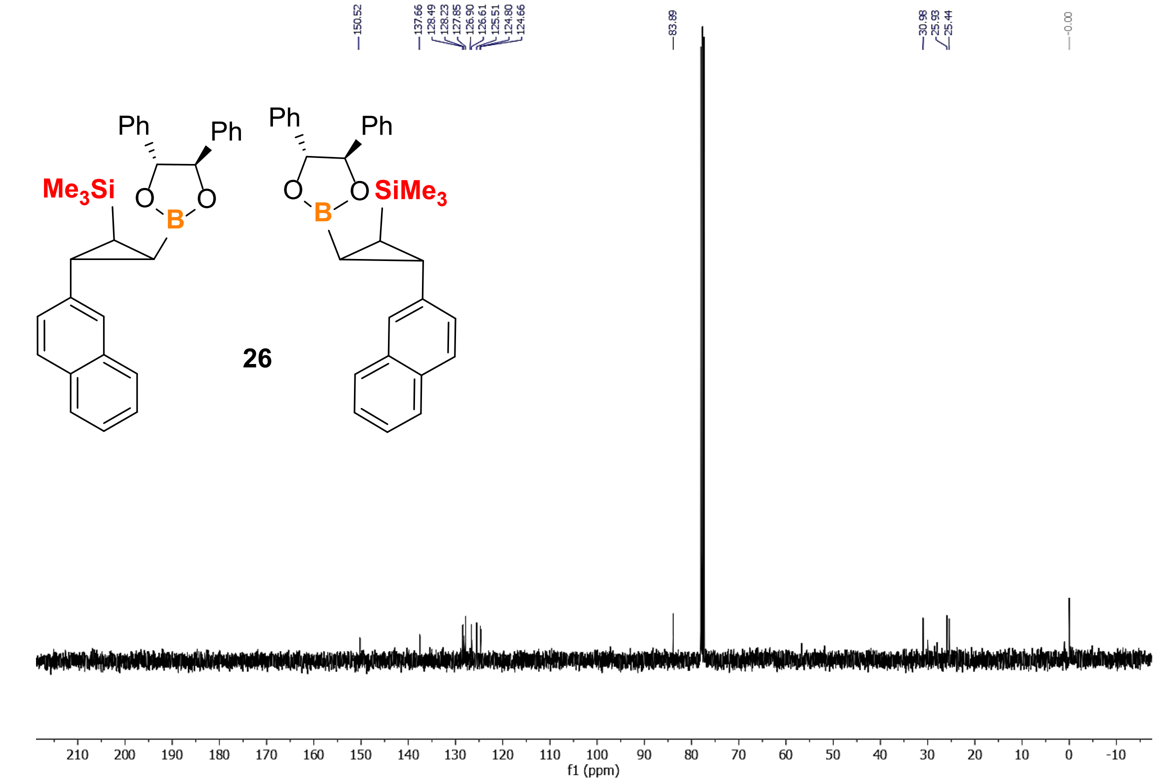

^{11}**B NMR** (CDCl$_3$, 128.3 MHz)

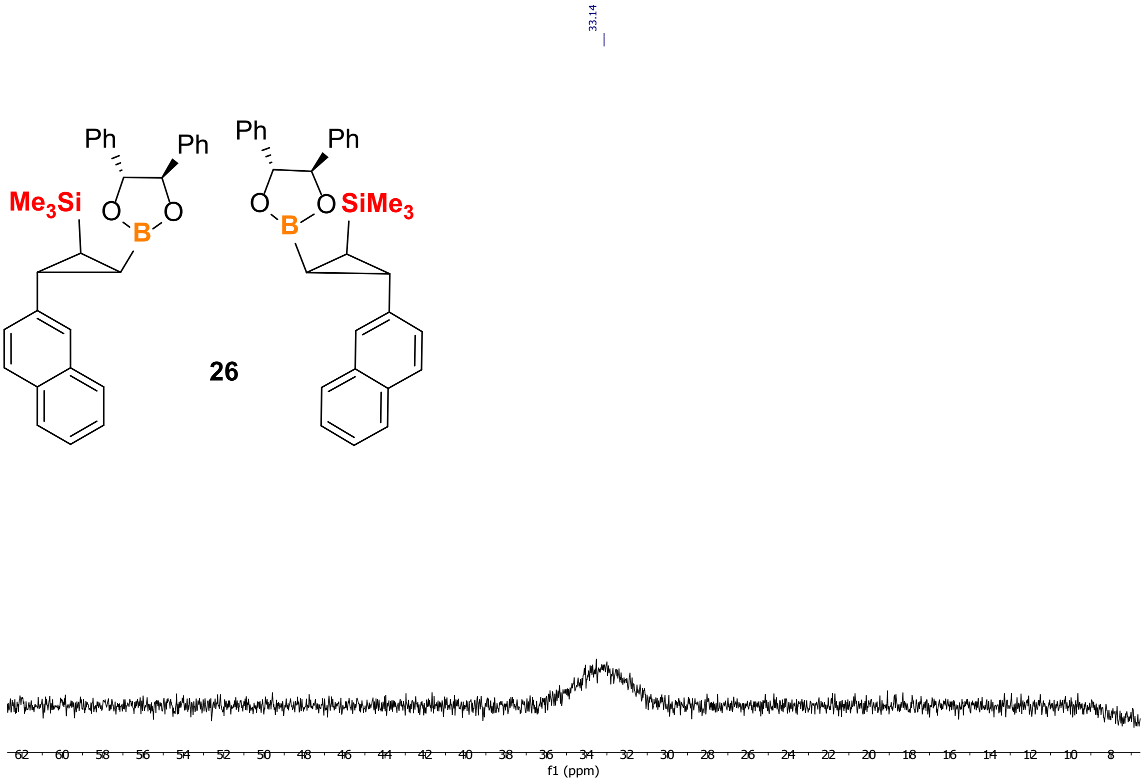

^{1}H NMR (CDCl$_3$, 400 MHz)

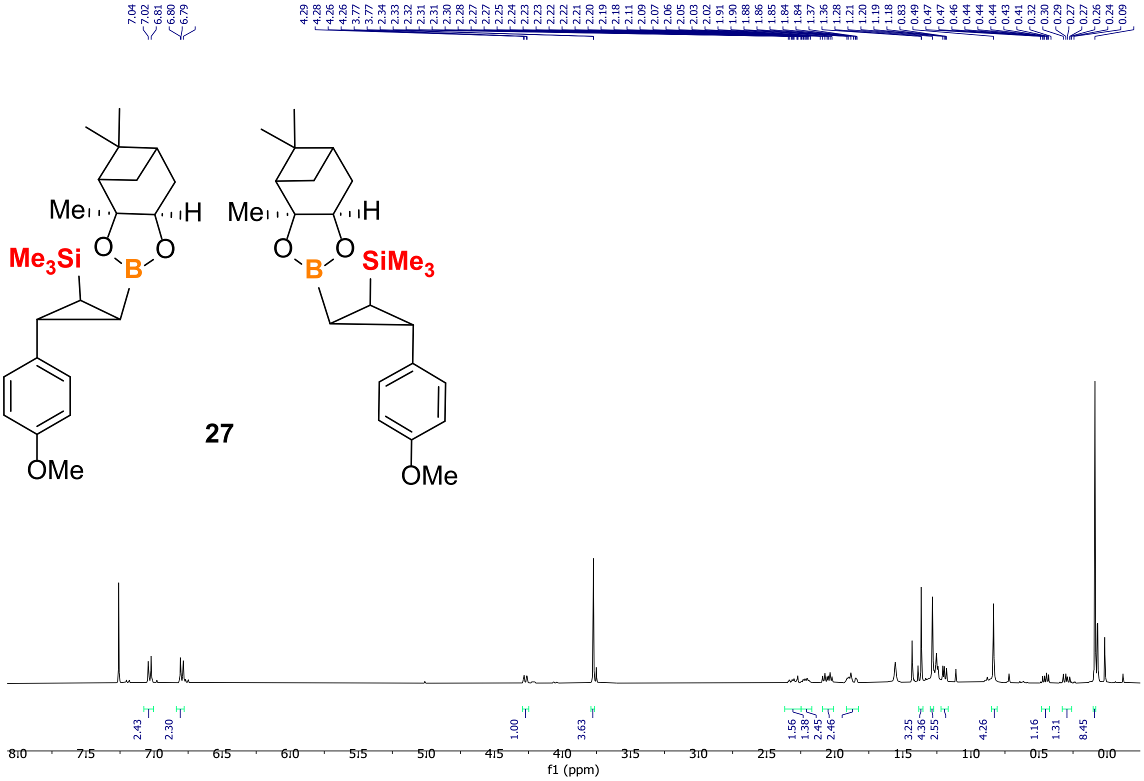

^{13}C NMR (CDCl$_3$, 100 MHz)

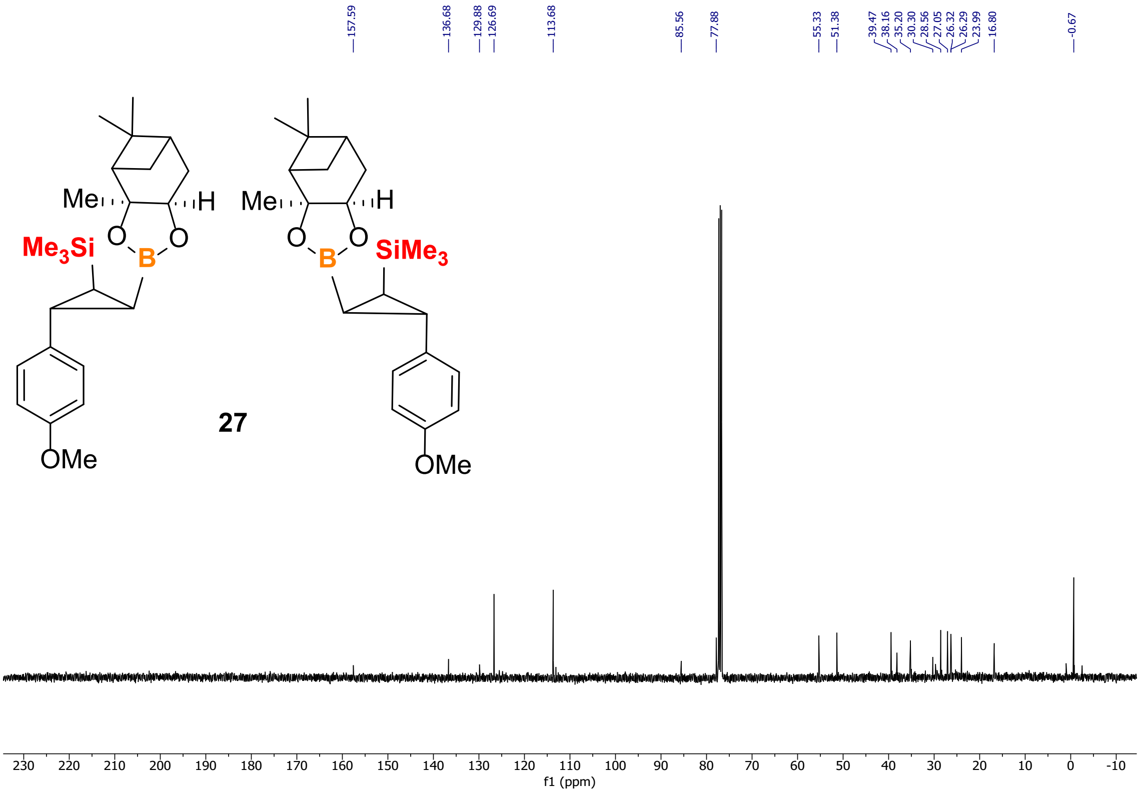

^{11}B NMR (CDCl$_3$, 128.3 MHz)

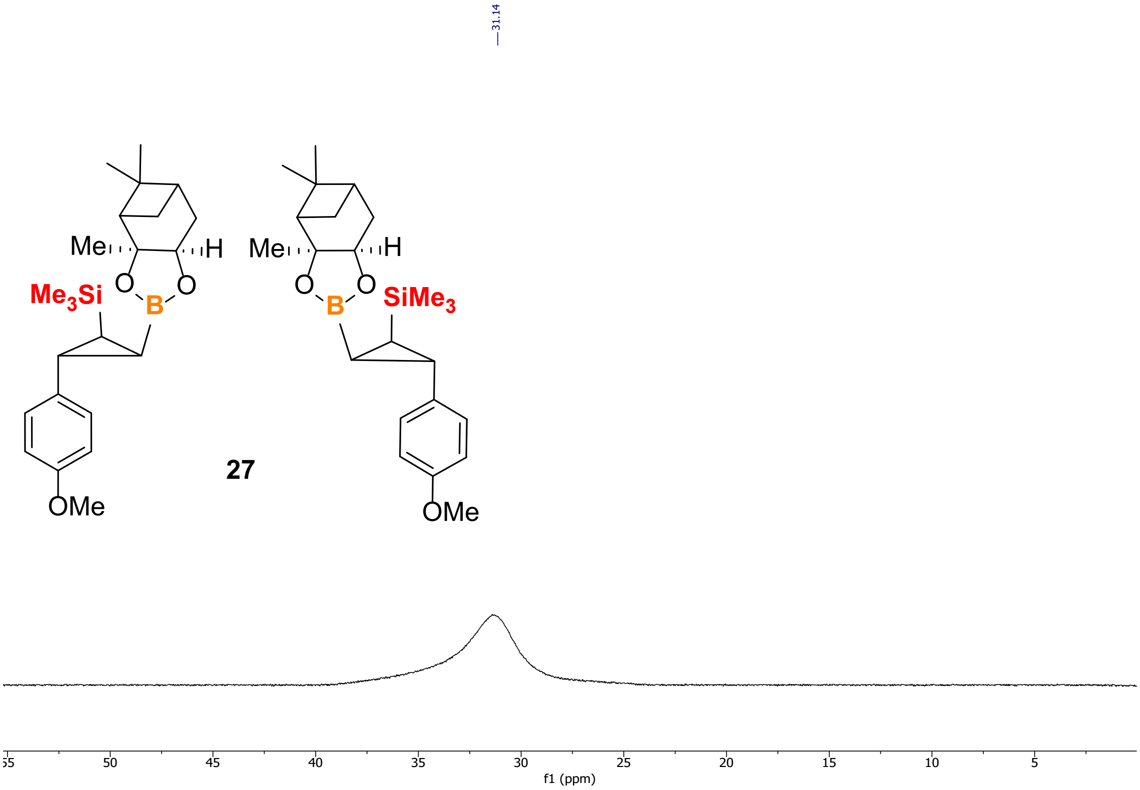

^{1}H NMR ($CDCl_3$, 400 MHz)

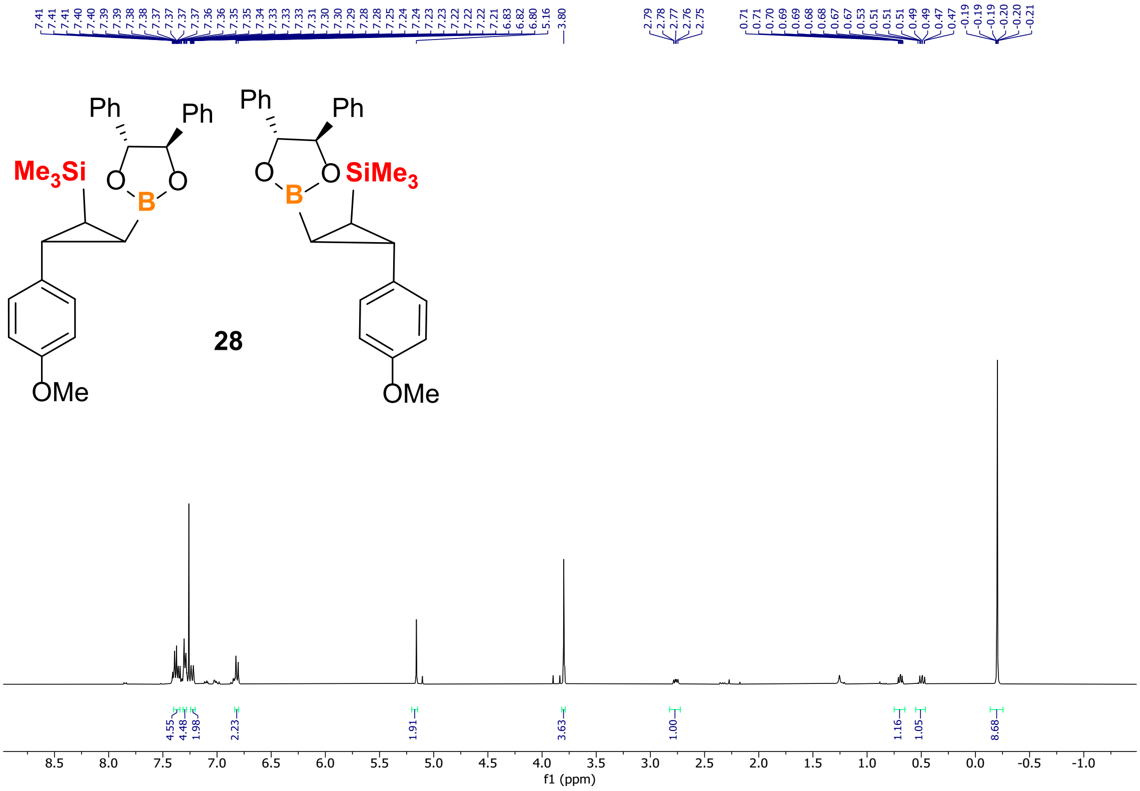

^{13}C **NMR** ($CDCl_3$, 100 MHz)

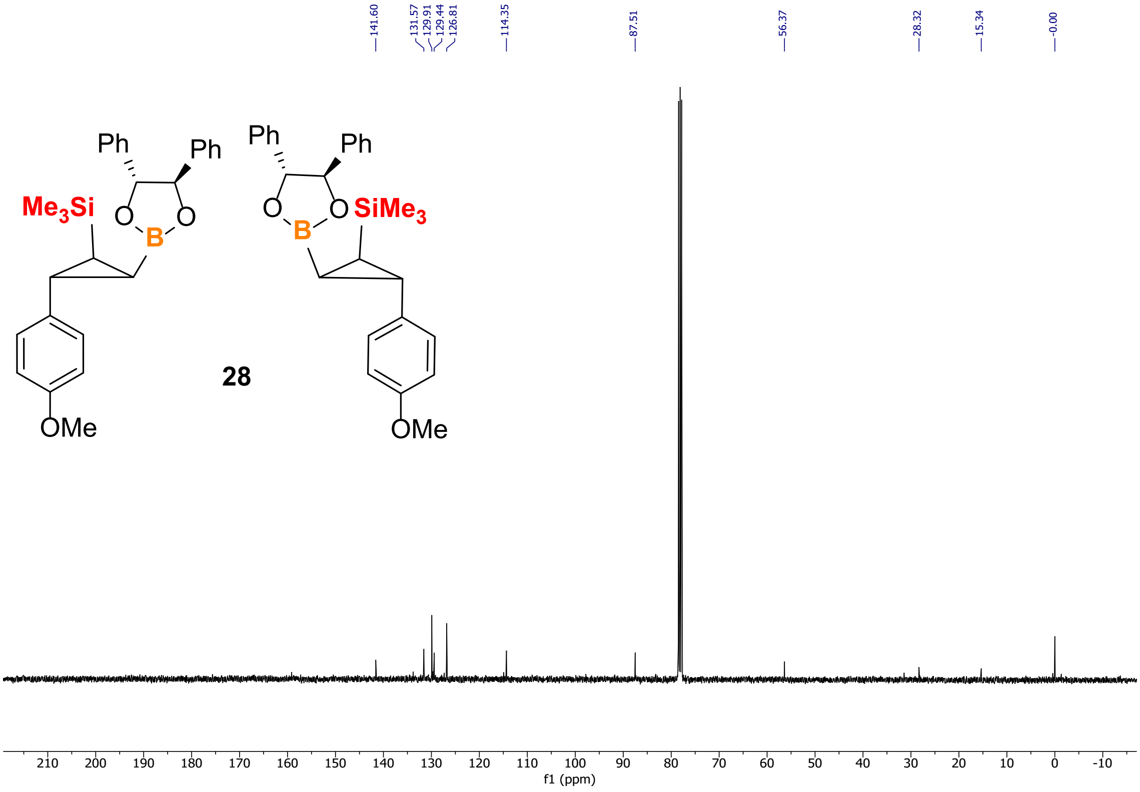

^{11}B NMR ($CDCl_3$, 128.3 MHz)

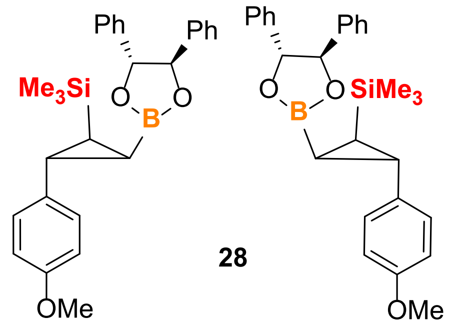

28

36.57

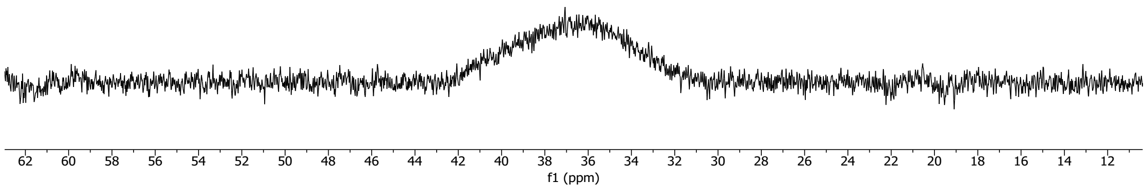

^{1}H NMR (CDCl$_3$, 400 MHz)

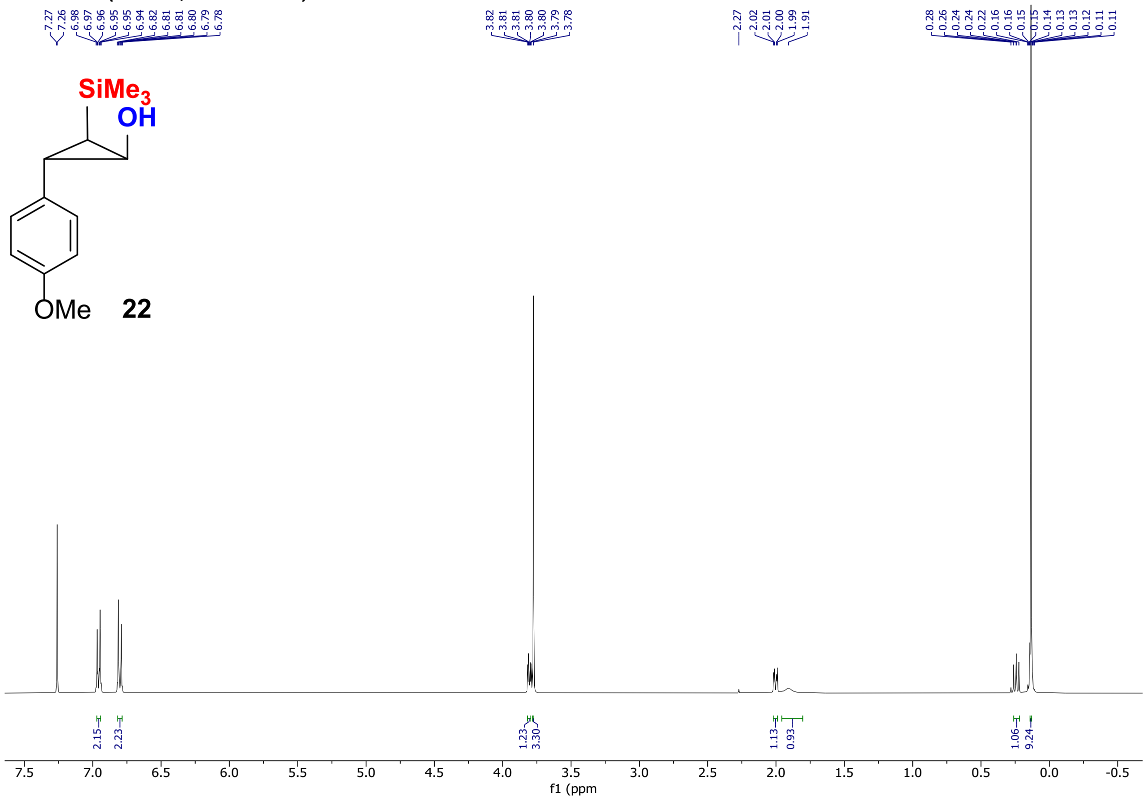

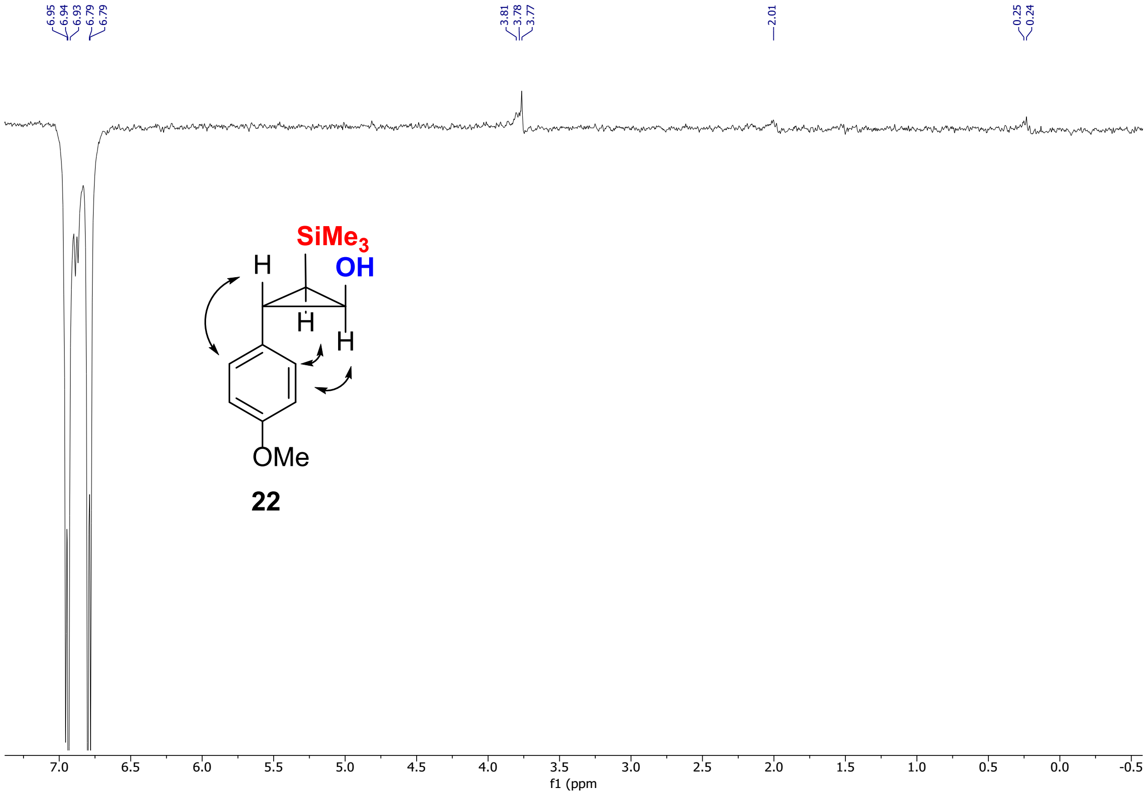

^{13}C NMR (CDCl$_3$, 100 MHz)

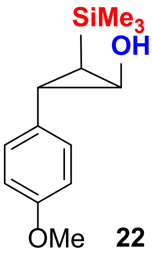

22

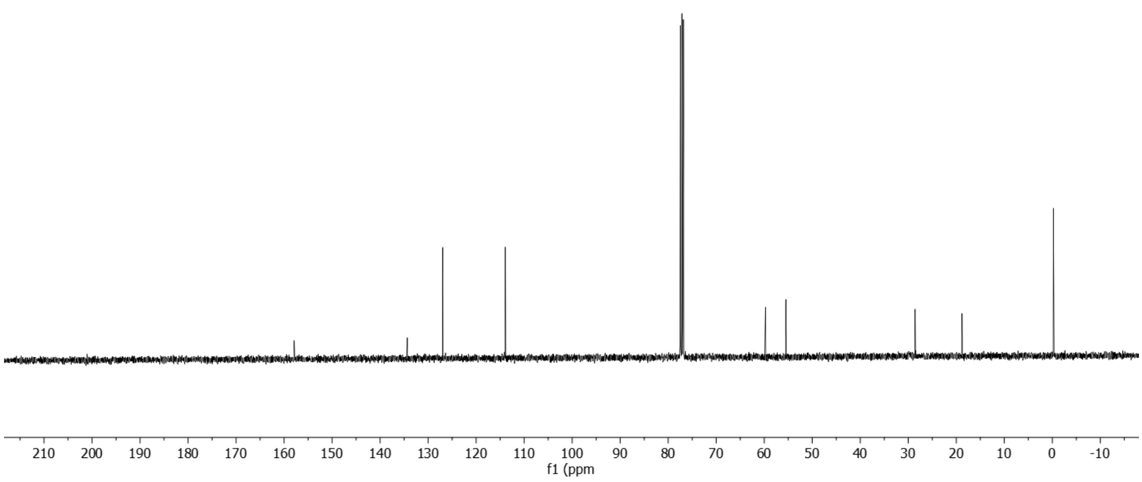

^{1}H NMR (CDCl$_3$, 400 MHz)

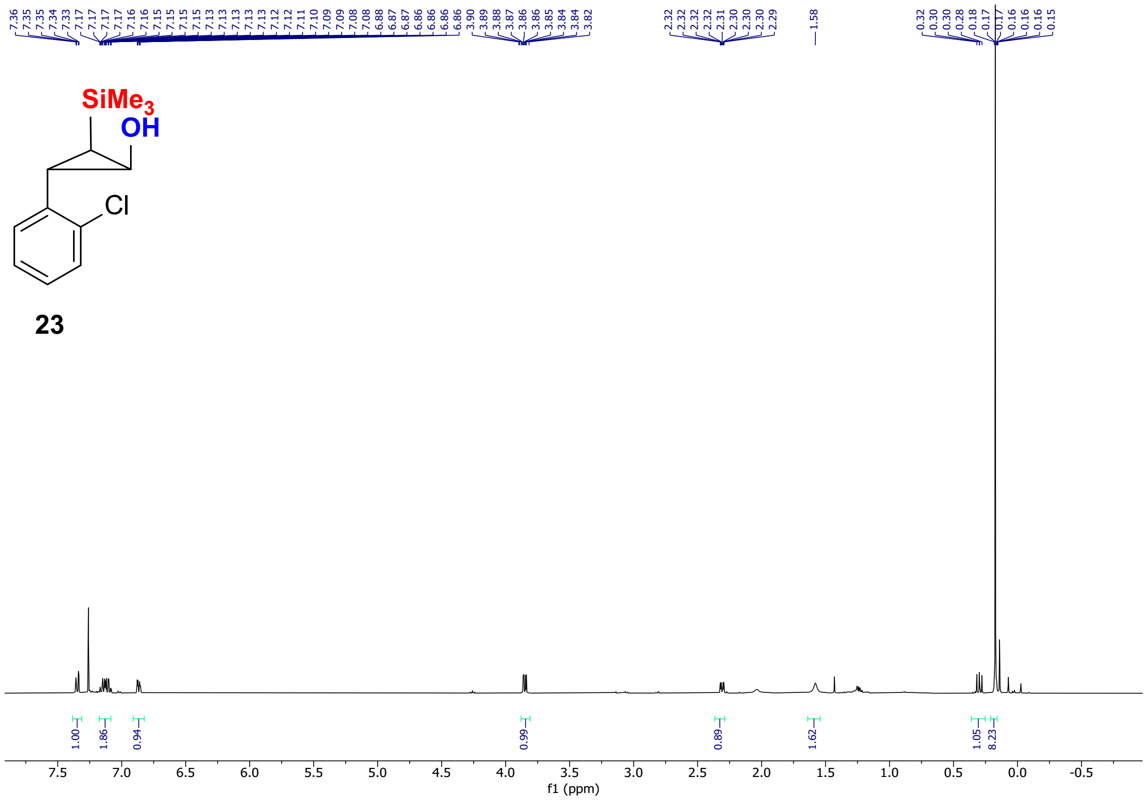

^{13}C NMR (CDCl3, 100 MHz)

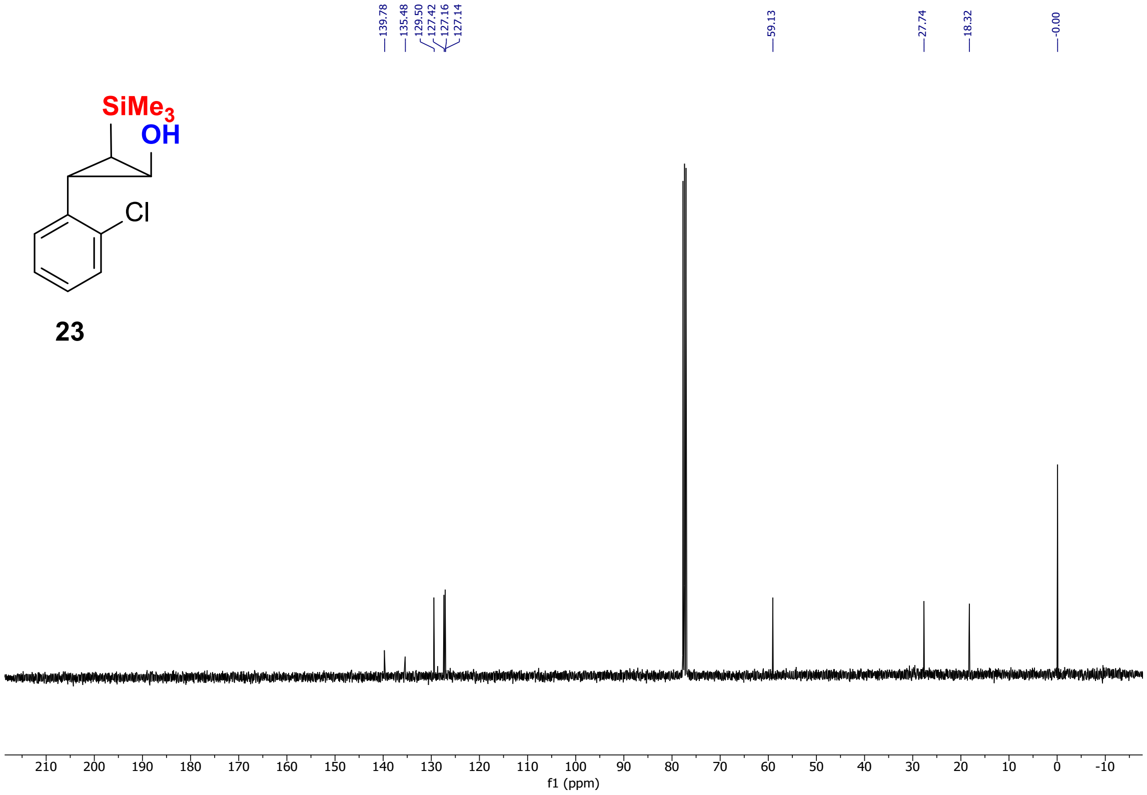

^{1}H NMR (CDCl3, 400 MHz)

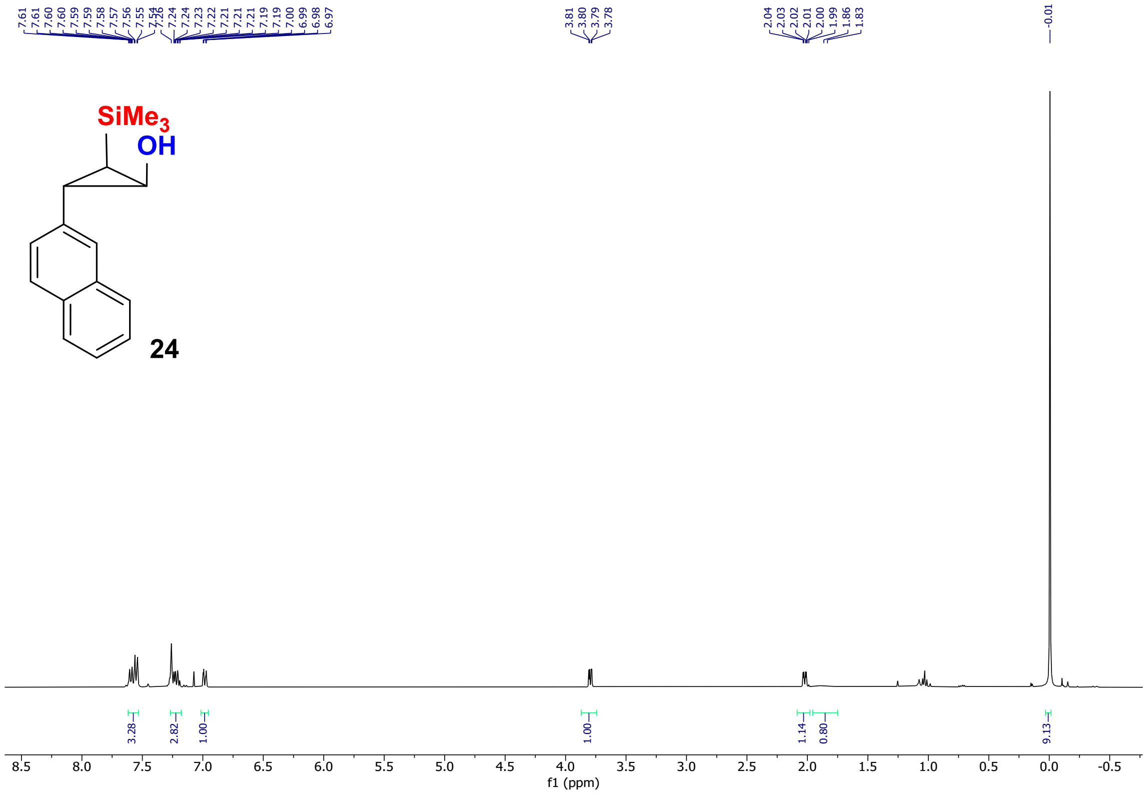

¹³C NMR ($CDCl_3$, 100 MHz)

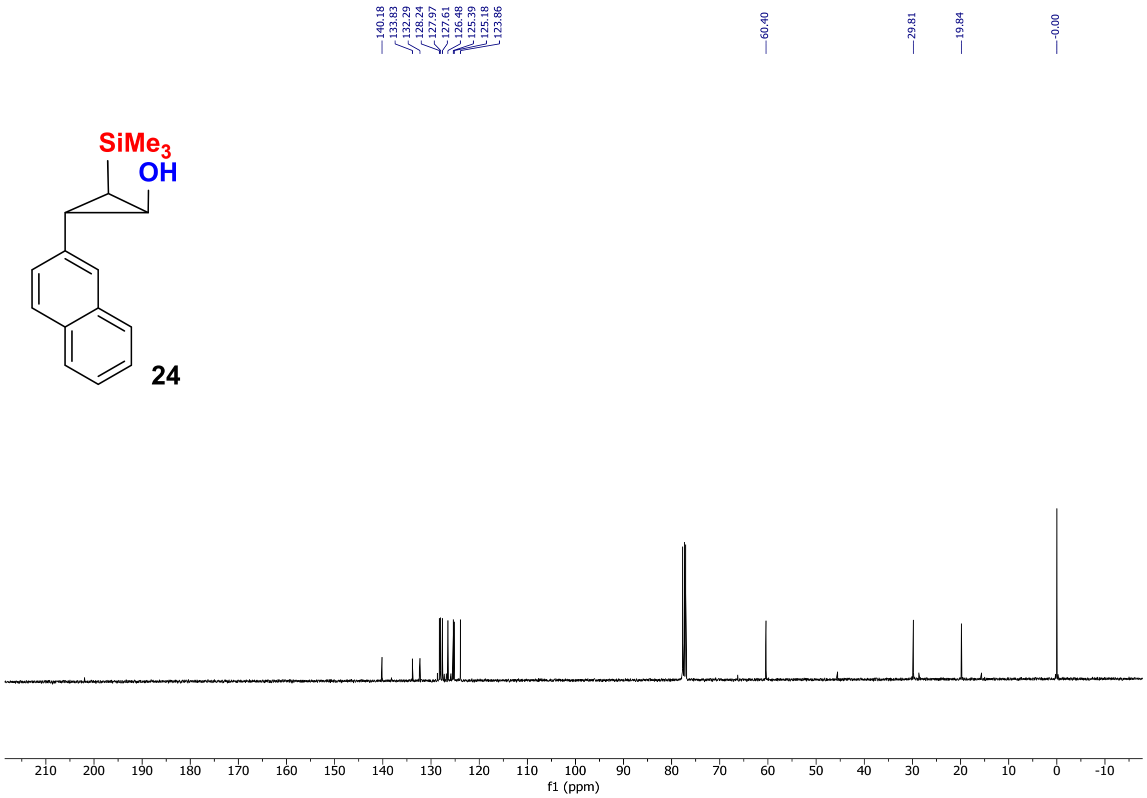

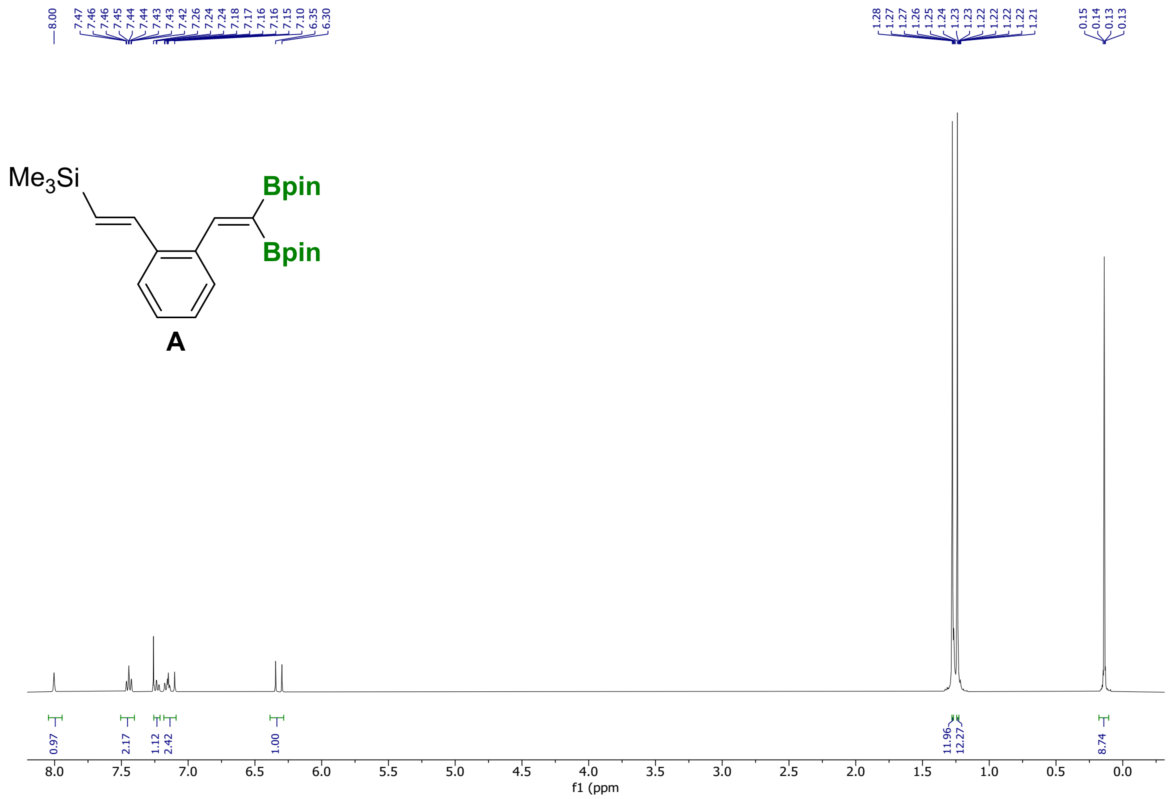

13C NMR (CDCl$_3$, 100 MHz)

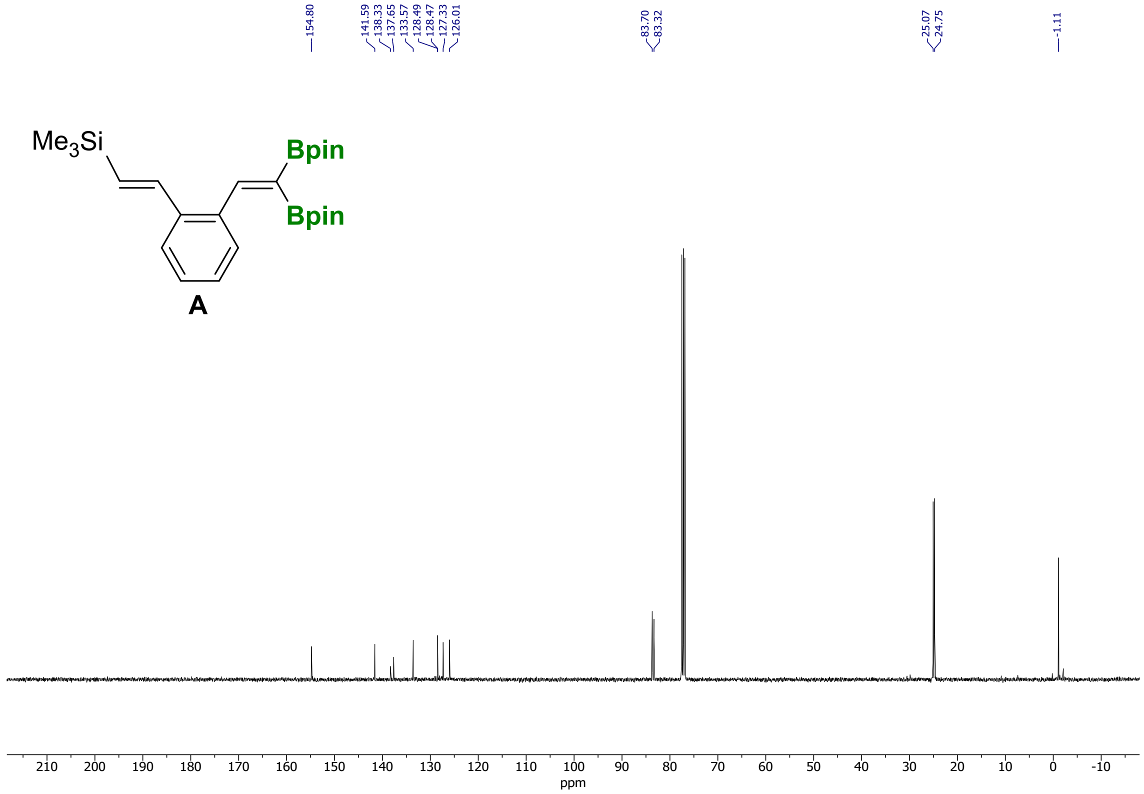

11B NMR (CDCl$_3$, 128.3 MHz)

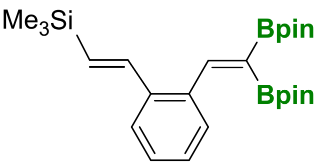

A

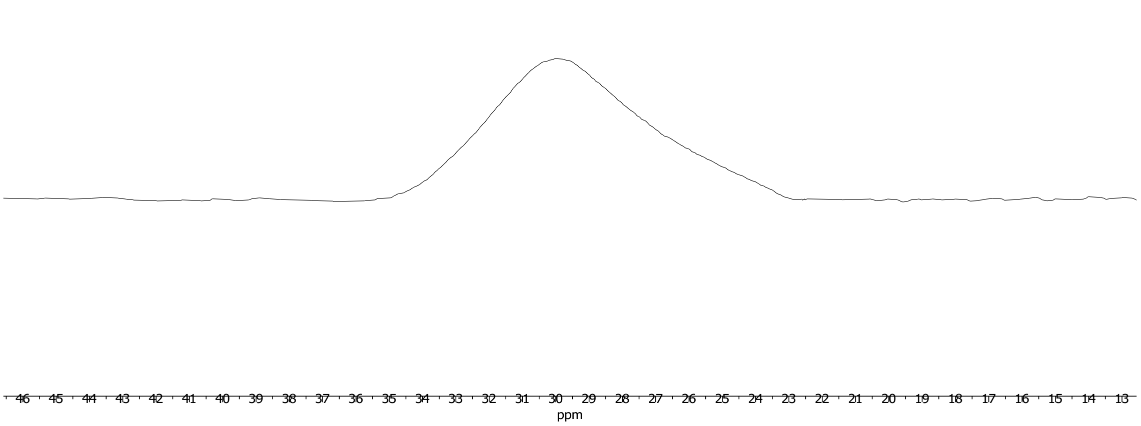

Enantiomeric ratio determined by HPLC

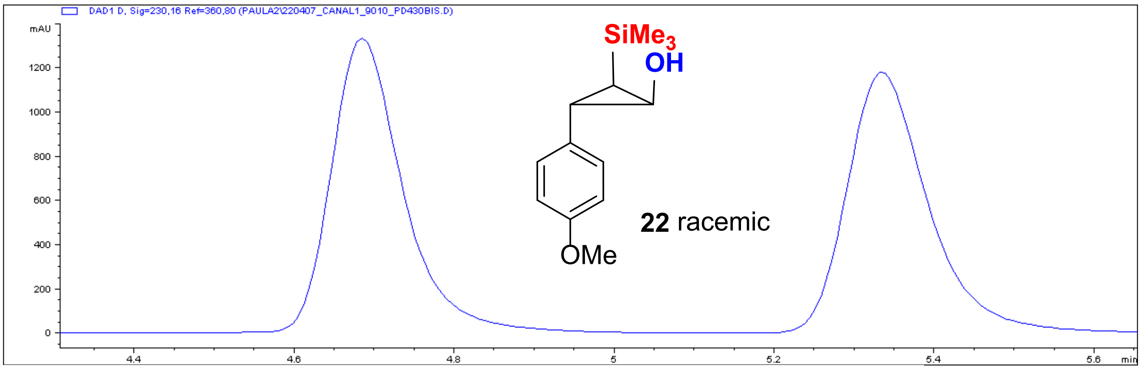

Signal 1: DAD1 D, Sig=230,16 Ref=360,80

Peak #	RetTime [min]	Type	Width [min]	Area [mAU*s]	Height [mAU]	Area %
1	4.685	MM	0.1010	8067.01270	1331.60876	50.0668
2	5.334	MM	0.1145	8045.49219	1171.60022	49.9332

Totals : 1.61125e4 2503.20898

===

*** End of Report ***

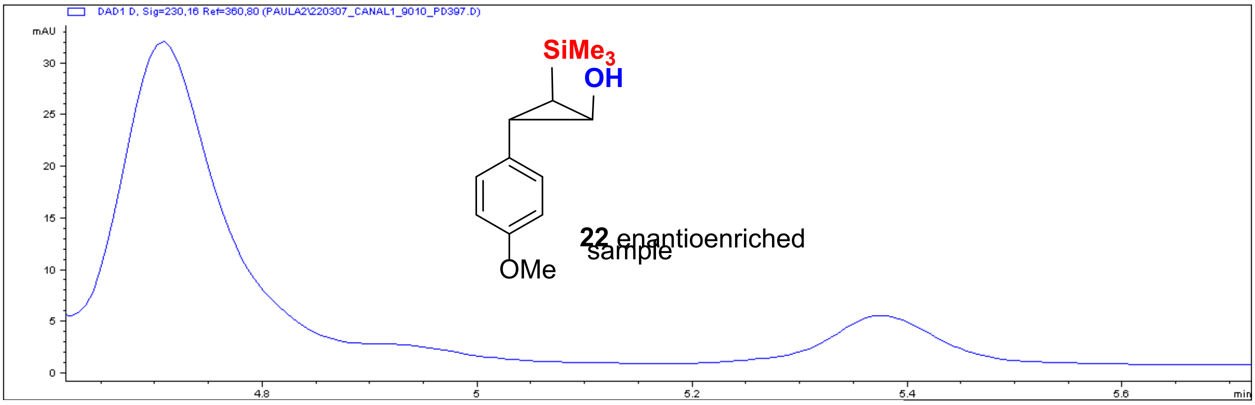

Signal 1: DAD1 D, Sig=230,16 Ref=360,80

Peak #	RetTime [min]	Type	Width [min]	Area [mAU*s]	Height [mAU]	Area %
1	4.707	MM	0.1017	179.47340	29.41964	89.1189
2	5.376	MM	0.0936	21.91317	3.90029	10.8811

Totals : 201.38657 33.31993

*** End of Report ***

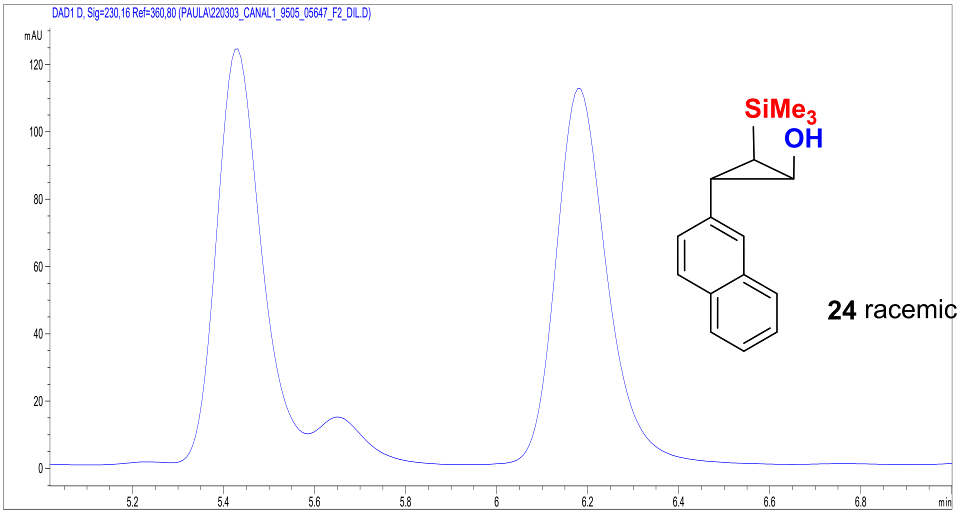

Signal 1: DAD1 D, Sig=230,16 Ref=360,80

Peak #	RetTime [min]	Type	Width [min]	Area [mAU*s]	Height [mAU]	Area %
1	5.431	MM	0.1101	802.87500	121.52465	50.1483
2	6.183	MM	0.1220	798.12793	109.07439	49.8517

Totals : 1601.00293 230.59904

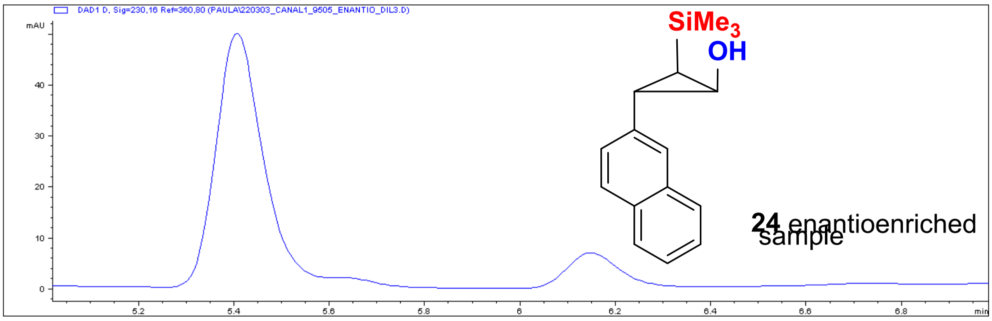

Signal 1: DAD1 D, Sig=230,16 Ref=360,80

Peak #	RetTime [min]	Type	Width [min]	Area [mAU*s]	Height [mAU]	Area %
1	5.406	MM	0.1119	332.06165	49.46257	89.6125
2	6.148	MM	0.1071	38.49122	5.98982	10.3875
Totals :				370.55287	55.45239	

X-Ray single-crystal diffraction analysis for 25

Method for crystal growth

A saturated solution of 5-10 mg of solid in 0.1 mL of dichloromethane was prepared in a 2mL GC-vial. 1 mL of pentane was then carefully added forming two layers. The system was then allowed to slowly evaporate with a needle in the cap until the crystals were obtained

Ortep-Drawing (thermal ellipsoids draw at 50% level) in two orientations.

Crystal preparation: The measured crystal of **PD392** was selected using a Zeiss stereomicroscope using polarized light and prepared under inert conditions immersed in perfluoropolyether as protecting oil for manipulation. Crystals of **PD392** were selected using a Zeiss stereomicroscope using polarized light and prepared under inert conditions immersed in perfluoropolyether as protecting oil for manipulation.

Data collection: Crystal structure determinations for sample **PD392** was carried out using a Apex DUO Kappa 4-axis goniometer equipped with an APPEX 2 4K CCD area detector, a Microfocus

Source E025 IuS using CuK_α radiation, Quazar MX multilayer Optics as monochromator and an Oxford Cryosystems low temperature device Cryostream 700 plus (*T* = -173 °C). Full-sphere data collection was used with ω and φ scans. *Programs used*: Data collection APEX-2[i], data reduction Bruker Saint[ii] V/.60A and absorption correction TWINABS[iii].

Structure Solution and Refinement: Crystal structure solution was achieved using the computer program SHELXT[iv]. Visualization was performed with the program SHELXle[v]. Missing atoms were subsequently located from difference Fourier synthesis and added to the atom list. Least-squares refinement on F^2 using all measured intensities was carried out using the program SHELXL 2015[vi]. All non-hydrogen atoms were refined including anisotropic displacement parameters. **Comments to the structure**: The absolute structure could be determined reliable with a Flack value of 0.017(61) and Flack value based on Parsons' quotients of 0.027(58) (Flack X determined using 1698 quotients [(I+)-(I-)]/[(I+)+(I-)]).[vii] The Flack (Parsons or Hooft) parameter value for the correct absolute structure determination should be 0; the inverted structure would give 1; always taking in account the standard deviation. The absolute configuration based on the absolute structure of the measured crystals was determined with: ***R*(C1), *S*(C3), S(C5), *S*(C6), S(C11), *R*(C12)** and ***S*(C13)**. No A- or B-alerts were observed.

This compound crystallizes in the monoclinic chiral space group $P2_1$. The asymmetric unit contains one molecule of the chiral compound. Although the crystal measured was a needle of small dimensions (0.30 x 0.03 x 0.02 $Å^3$), the structure was of enough quality for the absolute structure determination by measuring with copper radiation. The absolute structure could be determined reliable with a Flack value of 0.017(61) and Flack value based on Parsons' quotients of 0.027(58) (references: Flack H.D., Acta Cryst. A39 (1983) 876, Parsons S., Flack H., Acta Cryst. A39 (2004) S61, Parson, Flack and Wagner, Acta Cryst. B69 (2013) 249-259; Flack X determined using 1698 quotients [(I+)-(I-)]/[(I+)+(I-)]). The Flack (Parsons or Hooft) parameter value for the correct absolute structure determination should be 0; the inverted structure would give 1; always taking in account the standard deviation. The structure measured is of good quality (no A- or B-alerts) and publishable with R1 value of 6.27 %.

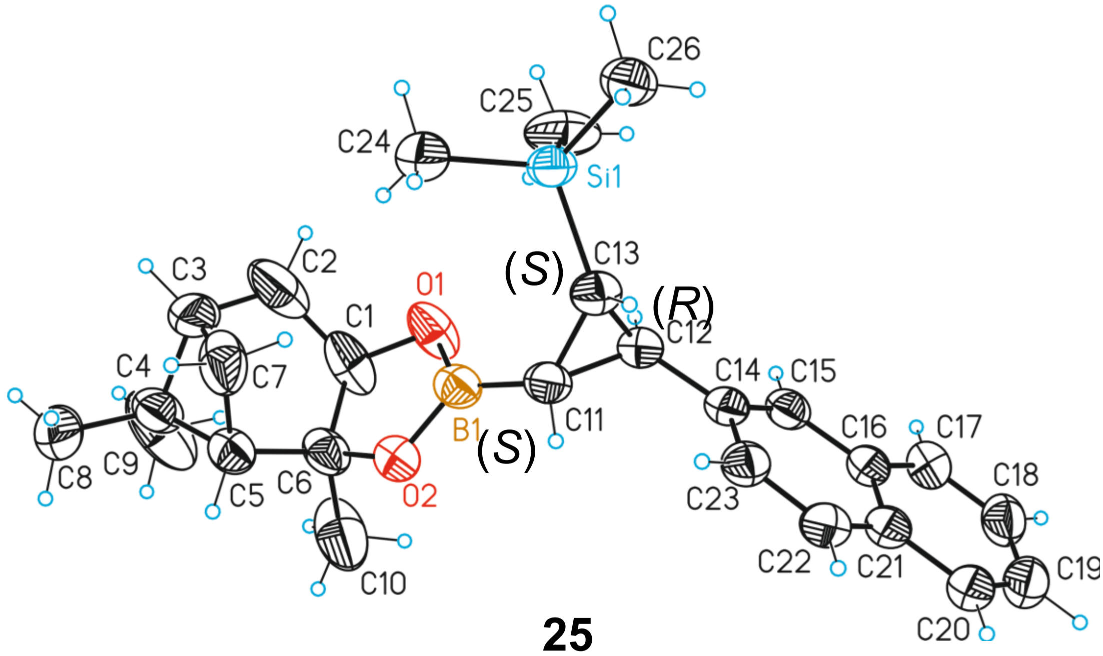

25

Table 1. Crystal data and structure refinement for cu_PD392_0m.

Identification code	cu_PD392_0m
Empirical formula	C26 H35 B O2 Si
Formula weight	418.44
Temperature	100(2)K
Wavelength	1.54178 Å
Crystal system	monoclinic
Space group	P 21

Unit cell dimensions	a = 13.0062(6)Å	α= 90°.
	b = 6.3738(3)Å	β = 99.168(3)°.
	c = 14.7534(7)Å	γ = 90°.
Volume	1207.42(10) $Å^3$	
Z	2	
Density (calculated)	1.151 Mg/m^3	
Absorption coefficient	0.991 mm^{-1}	
F(000)	452	
Crystal size	0.300 x 0.030 x 0.020 mm^3	
Theta range for data collection	3.034 to 68.180°.	
Index ranges	-15<=h<=13,-7<=k<=7,-17<=l<=17	
Reflections collected	8922	
Independent reflections	4214[R(int) = 0.0426]	
Completeness to theta =68.180°	98.0%	
Absorption correction	Multi-scan	
Max. and min. transmission	0.74 and 0.59	
Refinement method	Full-matrix least-squares on F^2	
Data / restraints / parameters	4214/ 1/ 278	
Goodness-of-fit on F^2	1.089	
Final R indices [I>2sigma(I)]	R1 = 0.0627, wR2 = 0.1657	
R indices (all data)	R1 = 0.0710, wR2 = 0.1715	
Flack parameter	x =0.03(6)	
Largest diff. peak and hole	0.273 and -0.249 e.$Å^{-3}$	

Table 2. Bond lengths [Å] and angles [°] for cu_PD392_0m.

Bond lengths----

Si1	C26	1.858(6)
Si1	C25	1.860(7)
Si1	C13	1.875(5)
Si1	C24	1.875(6)
B1	O1	1.345(7)
B1	O2	1.360(7)
B1	C11	1.539(8)
O1	C1	1.445(6)
O2	C6	1.454(7)
C1	C6	1.527(9)
C1	C2	1.539(12)
C1	H1	1.0000
C2	C3	1.521(13)
C2	H2A	0.9900
C2	H2B	0.9900
C3	C7	1.548(12)
C3	C4	1.558(9)
C3	H3	1.0000
C4	C9	1.516(11)
C4	C8	1.532(9)
C4	C5	1.552(8)
C5	C6	1.508(7)
C5	C7	1.515(8)
C5	H5	1.0000
C6	C10	1.507(8)
C7	H7A	0.9900
C7	H7B	0.9900
C8	H8A	0.9800
C8	H8B	0.9800
C8	H8C	0.9800
C9	H9A	0.9800
C9	H9B	0.9800
C9	H9C	0.9800
C10	H10A	0.9800
C10	H10B	0.9800

C10 H10C 0.9800
C11 C12 1.517(7)
C11 C13 1.529(7)
C11 H11 1.0000
C12 C14 1.494(6)
C12 C13 1.506(7)
C12 H12 1.0000
C13 H13 1.0000
C14 C15 1.366(7)
C14 C23 1.418(7)
C15 C16 1.422(7)
C15 H15 0.9500
C16 C21 1.414(7)
C16 C17 1.425(7)
C17 C18 1.361(8)
C17 H17 0.9500
C18 C19 1.407(10)
C18 H18 0.9500
C19 C20 1.356(8)
C19 H19 0.9500
C20 C21 1.426(7)
C20 H20 0.9500
C21 C22 1.417(7)
C22 C23 1.372(7)
C22 H22 0.9500
C23 H23 0.9500
C24 H24A 0.9800
C24 H24B 0.9800
C24 H24C 0.9800
C25 H25A 0.9800
C25 H25B 0.9800
C25 H25C 0.9800
C26 H26A 0.9800
C26 H26B 0.9800
C26 H26C 0.9800

Angles----------
C26 Si1 C25 109.4(3)

C26	Si1	C13	107.6(2)
C25	Si1	C13	111.1(3)
C26	Si1	C24	108.1(3)
C25	Si1	C24	111.3(4)
C13	Si1	C24	109.1(3)
O1	B1	O2	113.1(5)
O1	B1	C11	128.3(5)
O2	B1	C11	118.5(5)
B1	O1	C1	108.2(4)
B1	O2	C6	109.5(5)
O1	C1	C6	106.1(5)
O1	C1	C2	109.4(6)
C6	C1	C2	115.4(5)
O1	C1	H1	108.6
C6	C1	H1	108.6
C2	C1	H1	108.6
C3	C2	C1	112.4(6)
C3	C2	H2A	109.1
C1	C2	H2A	109.1
C3	C2	H2B	109.1
C1	C2	H2B	109.1
H2A	C2	H2B	107.8
C2	C3	C7	109.7(5)
C2	C3	C4	111.6(8)
C7	C3	C4	87.1(5)
C2	C3	H3	115.1
C7	C3	H3	115.1
C4	C3	H3	115.1
C9	C4	C8	108.0(6)
C9	C4	C5	121.2(6)
C8	C4	C5	110.3(5)
C9	C4	C3	118.5(7)
C8	C4	C3	112.8(6)
C5	C4	C3	84.4(5)
C6	C5	C7	108.0(4)
C6	C5	C4	113.8(5)
C7	C5	C4	88.5(5)
C6	C5	H5	114.6

C7	C5	H5	114.6
C4	C5	H5	114.6
O2	C6	C10	104.3(6)
O2	C6	C5	108.4(4)
C10	C6	C5	112.2(5)
O2	C6	C1	102.9(4)
C10	C6	C1	115.0(7)
C5	C6	C1	113.1(5)
C5	C7	C3	86.1(5)
C5	C7	H7A	114.3
C3	C7	H7A	114.3
C5	C7	H7B	114.3
C3	C7	H7B	114.3
H7A	C7	H7B	111.4
C4	C8	H8A	109.5
C4	C8	H8B	109.5
H8A	C8	H8B	109.5
C4	C8	H8C	109.5
H8A	C8	H8C	109.5
H8B	C8	H8C	109.5
C4	C9	H9A	109.5
C4	C9	H9B	109.5
H9A	C9	H9B	109.5
C4	C9	H9C	109.5
H9A	C9	H9C	109.5
H9B	C9	H9C	109.5
C6	C10	H10A	109.5
C6	C10	H10B	109.5
H10A	C10	H10B	109.5
C6	C10	H10C	109.5
H10A	C10	H10C	109.5
H10B	C10	H10C	109.5
C12	C11	C13	59.3(3)
C12	C11	B1	124.0(4)
C13	C11	B1	127.2(4)
C12	C11	H11	112.2
C13	C11	H11	112.2
B1	C11	H11	112.2

C14	C12	C13	121.3(4)
C14	C12	C11	118.5(4)
C13	C12	C11	60.8(3)
C14	C12	H12	115.1
C13	C12	H12	115.1
C11	C12	H12	115.1
C12	C13	C11	60.0(3)
C12	C13	Si1	123.1(4)
C11	C13	Si1	126.2(4)
C12	C13	H13	112.6
C11	C13	H13	112.6
Si1	C13	H13	112.6
C15	C14	C23	118.7(4)
C15	C14	C12	119.4(5)
C23	C14	C12	121.9(4)
C14	C15	C16	121.7(5)
C14	C15	H15	119.2
C16	C15	H15	119.2
C21	C16	C15	119.2(4)
C21	C16	C17	119.0(4)
C15	C16	C17	121.8(5)
C18	C17	C16	120.4(5)
C18	C17	H17	119.8
C16	C17	H17	119.8
C17	C18	C19	120.5(5)
C17	C18	H18	119.7
C19	C18	H18	119.7
C20	C19	C18	120.8(5)
C20	C19	H19	119.6
C18	C19	H19	119.6
C19	C20	C21	120.5(5)
C19	C20	H20	119.7
C21	C20	H20	119.7
C16	C21	C22	118.4(4)
C16	C21	C20	118.8(5)
C22	C21	C20	122.8(5)
C23	C22	C21	120.8(5)
C23	C22	H22	119.6

C21	C22	H22	119.6
C22	C23	C14	121.1(5)
C22	C23	H23	119.4
C14	C23	H23	119.4
Si1	C24	H24A	109.5
Si1	C24	H24B	109.5
H24A	C24	H24B	109.5
Si1	C24	H24C	109.5
H24A	C24	H24C	109.5
H24B	C24	H24C	109.5
Si1	C25	H25A	109.5
Si1	C25	H25B	109.5
H25A	C25	H25B	109.5
Si1	C25	H25C	109.5
H25A	C25	H25C	109.5
H25B	C25	H25C	109.5
Si1	C26	H26A	109.5
Si1	C26	H26B	109.5
H26A	C26	H26B	109.5
Si1	C26	H26C	109.5
H26A	C26	H26C	109.5
H26B	C26	H26C	109.5

Table 3. Torsion angles [°] for cu_PD392_0m.

O2	B1	O1	C1	1.9(6)
C11	B1	O1	C1	178.0(5)
O1	B1	O2	C6	1.9(6)
C11	B1	O2	C6	-174.6(4)
B1	O1	C1	C6	-4.8(6)
B1	O1	C1	C2	120.3(6)
O1	C1	C2	C3	-118.1(7)
C6	C1	C2	C3	1.4(10)
C1	C2	C3	C7	44.4(9)
C1	C2	C3	C4	-50.4(10)
C2	C3	C4	C9	-39.8(9)
C7	C3	C4	C9	-149.8(7)
C2	C3	C4	C8	-167.3(6)
C7	C3	C4	C8	82.6(6)
C2	C3	C4	C5	82.9(6)
C7	C3	C4	C5	-27.1(4)
C9	C4	C5	C6	38.8(8)
C8	C4	C5	C6	166.5(5)
C3	C4	C5	C6	-81.3(6)
C9	C4	C5	C7	147.8(6)
C8	C4	C5	C7	-84.5(6)
C3	C4	C5	C7	27.7(6)
B1	O2	C6	C10	115.7(6)
B1	O2	C6	C5	-124.6(4)
B1	O2	C6	C1	-4.6(5)
C7	C5	C6	O2	62.8(6)
C4	C5	C6	O2	159.3(4)
C7	C5	C6	C10	177.4(8)
C4	C5	C6	C10	-86.1(8)
C7	C5	C6	C1	-50.6(7)
C4	C5	C6	C1	45.9(6)
O1	C1	C6	O2	5.6(6)
C2	C1	C6	O2	-115.7(6)
O1	C1	C6	C10	-107.1(7)
C2	C1	C6	C10	131.6(7)
O1	C1	C6	C5	122.3(5)

C2	C1	C6	C5	1.0(8)
C6	C5	C7	C3	86.7(5)
C4	C5	C7	C3	-27.9(5)
C2	C3	C7	C5	-84.1(7)
C4	C3	C7	C5	27.8(5)
O1	B1	C11	C12	-31.3(8)
O2	B1	C11	C12	144.5(5)
O1	B1	C11	C13	43.5(8)
O2	B1	C11	C13	-140.7(5)
C13	C11	C12	C14	111.9(5)
B1	C11	C12	C14	-131.5(5)
B1	C11	C12	C13	116.6(5)
C14	C12	C13	C11	-107.5(5)
C14	C12	C13	Si1	136.5(4)
C11	C12	C13	Si1	-116.0(5)
B1	C11	C13	C12	-111.4(5)
C12	C11	C13	Si1	111.0(5)
B1	C11	C13	Si1	-0.4(7)
C26	Si1	C13	C12	-114.1(4)
C25	Si1	C13	C12	5.7(5)
C24	Si1	C13	C12	128.8(5)
C26	Si1	C13	C11	171.1(4)
C25	Si1	C13	C11	-69.1(5)
C24	Si1	C13	C11	54.0(5)
C13	C12	C14	C15	-144.5(5)
C11	C12	C14	C15	144.2(5)
C13	C12	C14	C23	37.7(7)
C11	C12	C14	C23	-33.6(7)
C23	C14	C15	C16	0.5(7)
C12	C14	C15	C16	-177.4(4)
C14	C15	C16	C21	2.5(7)
C14	C15	C16	C17	-177.9(5)
C21	C16	C17	C18	0.5(8)
C15	C16	C17	C18	-179.1(5)
C16	C17	C18	C19	0.4(8)
C17	C18	C19	C20	-0.6(9)
C18	C19	C20	C21	-0.2(8)
C15	C16	C21	C22	-3.4(7)

C17	C16	C21	C22	176.9(4)
C15	C16	C21	C20	178.3(4)
C17	C16	C21	C20	-1.4(7)
C19	C20	C21	C16	1.2(8)
C19	C20	C21	C22	-177.0(5)
C16	C21	C22	C23	1.5(7)
C20	C21	C22	C23	179.7(5)
C21	C22	C23	C14	1.6(7)
C15	C14	C23	C22	-2.6(7)
C12	C14	C23	C22	175.3(4)

Symetry operations

1	'x, y, z'
2	'-x, y+1/2, -z'

Experimental of cu_PD392_0m.

Full sphere single crystal data collection of cu_PD392_0m where performed at 100 K on a Bruker Kappa Apex II DUO diffractometer equiped with a Cryostream 700 plus low temperature device, a microsource anode with Mo *Kα* (λ= 0.71073 Å)

References Experimental Part

1. Yamamoto, T.; Ishibashi, A.; Suginome, M. *Org. Lett*. **2019**, *21*, 6235.
2. Idowu, O. O.;Hayes, J. C.; Reid, W. B.; Watson, D. A. *Org. Lett*. **2021**, *23*, 4838.
3. Krause, A. B.; McAtee, J. R.; Yap, G. P. A.; Watson, D. A. *Org. Lett*. **2017**, *19*, 5641.
4. Dominguez-Molano, P.; Bru, G.; Salvado, O.; Maza, R.J.; Carbó, J.; Fernández, E. *Chem. Commun*., **2021**, *57*, 13361.
5. Miura, T.; Oku, N.; Murakami, M. *Angew. Chem. Int. Ed.* **2019**, *58*, 14620.

References for X-Ray single-crystal diffraction analysis for 25

[i] Data collection with APEX II version v2013.4-1. Bruker (2007). Bruker AXS Inc., Madison, Wisconsin, USA.

[ii] Data reduction with Bruker SAINT version V8.30c. Bruker (2007). Bruker AXS Inc., Madison, Wisconsin, USA.

[iii] TWINABS: V2012/1 Bruker (2001). Bruker AXS Inc., Madison, Wisconsin, USA. Blessing, Acta Cryst. **1995**, A51, 33-38.

[iv] SHELXT; V2014/4 (Sheldrick 2014). Sheldrick, G.M. *Acta Cryst.* **2015**, A71, 3-8.

[v] SHELXle; C.B. Huebschle, G.M. Sheldrick & B. Dittrich; J. Appl. Cryst. **2011**, 44, 1281-1284.

[vi] SHELXL; SHELXL-2014/7 (Sheldrick 2014). Sheldrick, G.M. *Acta Cryst.* **2015**, C71, 3-8.

[vii] Flack H.D., *Acta Cryst*., **1983**, A39, 876, Parsons S., Flack H., Acta Cryst., **2004**, A39, S61, Parson, Flack and Wagner, Acta Cryst., **2013**, B69, 249-259.
